# Tests of the DFT Ladder for the Fulminic Acid Challenge

**DOI:** 10.1021/jacs.4c13823

**Published:** 2025-04-16

**Authors:** Ashley M. Allen, Laura N. Olive Dornshuld, Patricia A. Gonzalez Franco, Wesley D. Allen, Henry F. Schaefer

**Affiliations:** Center for Computational Quantum Chemistry, University of Georgia, Athens, Georgia 30602, United States

## Abstract

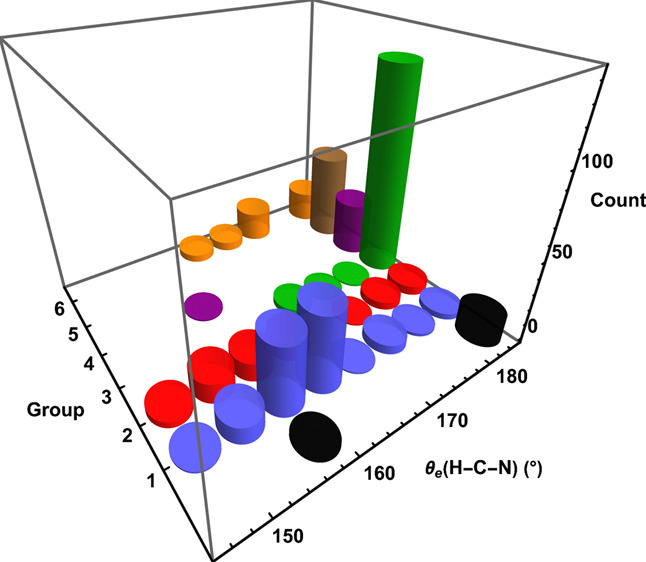

Properties of the historically pivotal fulminic acid (HCNO) molecule have been computed with a panoply of 473 density functionals of all varieties, providing a snapshot of the performance of contemporary density functional theory (DFT) for a challenging chemical system. Exhaustive tabulations and statistical analyses have been carried out for geometric parameters, vibrational frequencies, barriers to linearity, and the HCN–O dissociation energy. As the DFT ladder is climbed, confusion rather than consensus ensues regarding the details of the distinctive, extremely flat H–C–N bending potential of fulminic acid and whether the equilibrium structure is linear or bent. While high-ranking DFT functionals produce the smallest errors for the HCN + O(^3^*P*) → HCNO reaction energy, lower rungs emerge as the best performers for many of the bond distances and harmonic vibrational frequencies. This research shows that the current DFT zoo of approximations does not constitute a transparent ladder of increasingly accurate methods that consistently converges on definitive predictions for various properties of HCNO. Additional analyses are performed on the side effects of popular dispersion corrections on the covalently bonded properties and thermochemistry of HCNO.

## 1 Introduction

Fulminic acid (HCNO) has influenced the development of chemistry for over 200 years.^1^ Its explosive salts were first discovered in 1800 by Edward Howard, who appropriately called them “fulminating” after a Latin word meaning “to strike like lightning.”^1−4^ The chemical composition of these salts piqued the interest of Liebig, who discovered in 1824 that silver fulminate was identical in composition to the silver cyanate compound prepared by Wöhler.^1,5,6^ As such, fulminic and cyanic acids became preeminent early examples of the emerging concept of chemical isomerism.^6^ The elementary structure of fulminic acid was elusive for well over a century,^1,7−20^ as many false starts pursued a two-carbon species, but in 1966 an IR spectral analysis of gaseous fulminic acid finally proved it to be a tetra-atomic molecule with H–C–N–O connectivity.^17,18^ Over the next four decades, herculean efforts toward the accumulation and analysis of high-resolution rovibrational spectra revealed fascinating peculiarities in the quantum states of fulminic acid arising from large-amplitude H–C–N bending motion.^21−58^ The molecule was initially deemed quasilinear,^32^ a designation given to species with a nonlinear electronic minimum but a low barrier to linearity that gives rovibrational spectra characteristic of a linear framework.^41,59^ However, HCNO was later designated as quasibent, meaning that it possesses a vibrationless linear electronic minimum but has an *effective* one-dimensional, double-well H–C–N bending curve that yields rovibrational spectra bearing signatures of a bent structure.^38,39^ The mercurial quasilinear/quasibent nature of fulminic acid has thus become a testing ground for methods of modern chemical physics.

Electronic wave function computations over the years have given muddled results for HCNO, as the geometry and vibrational frequencies exhibit an unusually strong dependence on both the orbital basis set and electron correlation method.^59−73^ In 1982, an MP3/6-31G** study^66^ found a linear equilibrium structure for HCNO; in contrast, MP2/6-31G** computations^67^ in 1989 gave a bent geometry with a barrier to linearity of 0.64 kcal mol^–1^. A more comprehensive investigation^68^ in 1991 further revealed the difficulty of the problem. In particular, both the 6-31G** and 6-311G** basis sets produced linear structures with MP3 and CISD but bent geometries with MP2 and MP4.^68^ Moreover, MCSCF/DZP computations predicted a bent molecule, while MRCISD treatments built on these MCSCF wave functions spawned either linear or bent structures depending on the reference space employed.^68^ The gold-standard CCSD(T) method was applied to HCNO several times between 1992 and 2009, and the results varied dramatically with the basis set.^59−62^ A TZ2P basis gave a linear structure in 1992, while the cc-pVQZ choice produced a bent molecule in 1996.^60,61^ More recent computations^59,62^ applied both frozen-core and all-electron CCSD(T) with the cc-pV*X*Z (*X* = 2–6) and cc-pCV*X*Z (*X* = 2–5) series of basis sets. The optimized H–C–N bending angle varied nearly 30° over the series, but as the complete basis set (CBS) limit was approached, the molecule evolved to a linear structure.^59,62^

Our fresh 2024 investigation^74^ was the first to apply high-order coupled cluster theory beyond CCSD(T) to the fulminic acid problem. Wave function methods as extensive as CCSDT(Q), CCSDTQ(P), and even CCSDTQP(H) were employed in conjunction with the cc-pCV*X*Z (*X* = 4–6) basis sets and assorted CBS extrapolations to definitively ascertain the equilibrium structure, harmonic vibrational frequencies, and enthalpy of formation of HCNO via focal point analyses (FPA).^74^ Striking fluctuations of the ω_5_ H–C–N bending frequency were observed in the all-electron correlation series with CBS extrapolation: ω_5_(π) = (604, 260*i*, 334, 120, 146, 49*i*, 52, 45) cm^–1^ for [HF, AE-MP2, AE-CCSD, AE-CCSD(T), AE-CCSDT, AE-CCSDT(Q), AE-CCSDTQ, AE-CCSDTQ(P)].^74^ The inclusion of scalar relativistic effects (MVD1) further decreased ω_5_ such that the final Born-Oppenheimer AE-CCSDTQ(P)/CBS + MVD1 ω_5_ frequency was merely 19 cm^–1^.^74^ Although usually considered auxiliary effects, core electron correlation, the diagonal Born-Oppenheimer correction (DBOC), and MVD1 terms were all found to significantly influence whether vibrationless HCNO is linear or bent.^74^ Because our 2024 computations on HCNO were pushed to the *ab initio* limit, highly accurate benchmark data finally exist for this species.^74^ While notable deficiencies in some results were found even at the CCSDT(Q) level, the rigorous ladder of coupled-cluster methods was successful in ultimately reaching convergence. It is worth emphasizing that the question of multireference electronic character in HCNO was carefully investigated in our earlier work^74^ by means of both coupled-cluster diagnostics and CASSCF computations. The difficulty in treating fulminic acid is certainly *not* the presence of strong multireference character that most DFAs are not designed to handle. Instead, the molecule exhibits an exquisite balance of basis set effects, dynamical electron correlation, and other influences.

The vexing fulminic acid problem attracted attention 30 years ago from electronic structure method developers during the exuberant period in which density functional theory (DFT)^75^ first gained popularity in computational chemistry. In 1994, Handy et al.^76^ wrote a paper entitled “Does Fulminic Acid Have a Bent Equilibrium Structure?” and attempted to answer this question by applying the SVWN and newly minted BLYP functionals in conjunction with DZP, TZ2P, and TZ2Pf basis sets. With the best basis set (TZ2Pf), SVWN essentially gave a linear structure, while BLYP yielded a bent molecule with an H–C–N angle of 161.9° and a barrier to linearity of 16 cm^–1^.^76^ An analysis of basis set trends suggested that BLYP retains a bent equilibrium structure in the CBS limit.^76^ Under the mistaken belief that the BLYP ω_5_ = 237 cm^–1^ result is close to the true harmonic frequency for the H–C–N bending mode, the authors concluded that the BLYP prediction of a bent structure was correct and that “modern DFT has a role to play even in the study of the most delicate properties of small molecules.”^76^ What is most striking about the paper is its highly optimistic perspective about the capabilities of Kohn-Sham (KS)-DFT: “We recall that in principle the KS method includes all correlation effects (double, triple, quadruple replacements, and beyond), and there is every hope that, as functionals are improved, they will all be truly represented.”^76^ Even though KS-DFT has emerged as the most popular electronic structure method in computational chemistry,^77^ its viability for problematic molecules such as HCNO is still an open question. Therefore, in this paper, we consider the following essential question: can the ladder of current DFT methods converge on definitive, precise predictions for fulminic acid and its “delicate” bending potential, or was the optimism expressed by the Cambridge group three decades ago misplaced?

Fulminic acid DFT results published after 1994 are surprisingly sparse. A 2022 study by Planells and Ferao^78^ includes an optimized structure for HCNO at the B3LYP-D3/def2-TZVP level with a near-linear ∠(H–C–N) = 178.8°. In 2023, Bégué and coworkers^79^ provided HCNO optimized structures and harmonic vibrational frequencies for three methods: B3LYP/6-311G(*d*,*p*), B3LYP-D3BJ/6-311++G(*d*,*p*), and M06-2X/6-311G(*d*,*p*). In all three cases, HCNO is linear with ω_5_ H–C–N bending frequencies equal to 241, 268, and 398 cm^–1^ for the B3LYP, B3LYP-D3BJ, and M06-2X functionals, respectively.^79^ Fulminic acid is a member of the W4-11 benchmarking data set developed by Karton et al.,^80^ which has been employed in numerous DFT studies.^81−84^ However, individual data for HCNO or other molecules are not typically reported in benchmarking papers. Moreover, equilibrium structures and vibrational frequencies are given far less attention than energetic properties in the DFT benchmarking literature.^83^

Recent developments in DFT have been extensively chronicled.^77,81−89^ In principle, DFT is exact, but the true exchange-correlation functional remains elusive, requiring reliance on density functional approximations (DFAs).^81^ The degree to which DFT is systematically improvable has been hotly debated in the literature, and disagreements abound as to whether there is indeed a hierarchical “Jacob’s Ladder” leading to DFT heaven.^77,81,82^ Nonetheless, members in the zoo of existing DFAs are ubiquitously classified in terms of sequential rungs in a ladder.^81^ Large DFT benchmarking studies across thousands of main-group molecules generally confirm the improved performance of higher-rung methods on a statistical basis,^81,82^ and proponents contend winsomely that density functionals *are* systematically improvable *if* one selects best in class. However, as analyzed by Gould and Dale,^86^ large and diverse data sets can neglect instances where DFT approaches fail badly and can give a misleading sense of security.

Given the historical prominence of fulminic acid in the history of chemistry and the lack of comprehensive DFT computations on this species thus far, we present here a detailed analysis of the performance of current DFT methods as applied to this notorious molecule. In particular, we analyze HCNO equilibrium geometries, corresponding vibrational frequencies, and the HCN + O(^3^*P*) → HCNO reaction energy to determine whether 473 DFT methods in current use provide any consensus or convergence, particularly on the quasibent/quasilinear nature of fulminic acid. This approach provides a fresh perspective because DFT research typically tracks the statistical performance of functionals when applied to a multitude of chemical systems rather than investigating whether the collective DFT armamentarium can converge on precise answers for a single molecule, as the practicing chemist would desire. The essential question we ask here is not whether some DFAs perform adequately well by the standards of some users, but whether the methods in totality provide a ladder that can converge on definitive predictions for “delicate” properties. For some HCNO properties of this type, such convergence requires methods to reach or exceed “chemical accuracy” (1 kcal mol^–1^), which is a stronger standard than applied in most DFT applications.

## 2 Theoretical Methods

Geometry optimizations and corresponding harmonic vibrational frequency computations were performed on both bent and linear HCNO structures using 473 density functional theory (DFT) methods, implemented in the Psi4 software package.^90^ The large C, N, O [7*s* 4*p* 3*d* 2*f* 1*g*], and H [4*s* 3*p* 2*d* 1*f*] def2-QZVP^91^ basis set was utilized to eliminate any concerns over basis set deficiencies. Selection of this basis set was informed by recent and comprehensive DFT assessments by Goerigk and coworkers.^81^ All DFT methods employed are specified in the Supporting Information, where in each case references are provided and proper functional names are matched with keywords in the Psi4 manual.^92^ Electron repulsion integrals (ERIs) were evaluated using density fitting, which is the default for DFT computations in Psi4, as bolstered by systematic tests demonstrating the high accuracy of this approach. Preliminary computations were carried out on 26 functionals (denoted with an asterisk in the Supporting Information) that were selected based on performance recommendations^81^ or popularity.^93^ This preliminary set of functionals was assessed at three different (radial, spherical) integration grid settings: (75, 302), (99, 590), and (175, 974). The sensitivity of HCNO optimum geometries and harmonic vibrational frequencies to both functional selection and computational parameters dictated the use of the finest available grid size (175, 974), as well as an energy convergence for geometry optimizations of 10^–8^*E*_h_ and an even tighter underlying SCF convergence of at least 2 × 10^–10^*E*_h_. Structures and frequencies were also computed for HCN with all functionals to yield reference data. Finally, the DFT methods were rigorously tested by application to the reaction energy of HCN + O(^3^*P*) → HCNO, which is a convenient route for determining the enthalpy of formation of fulminic acid.

For each DFT functional, optimizations were run twice from both bent and linear starting geometries. Bent cases that reoptimized to a linear structure were discarded as duplicates. Optimizations that produced a linear transition state were retained so that the barrier to linearity could be determined. For all structures that were minima, reaction energies were computed, as well as shifts in C–H and C–N bond distances and corresponding harmonic stretching frequencies compared to HCN. All functionals were assigned a percentile to assess performance for reaction energies, bond distances, shifts in bond distances, harmonic vibrational frequencies, and shifts in harmonic vibrational frequencies. For a sizable subset of the Psi4 data, confirmatory results were also obtained with the ORCA^94,95^ or Q-Chem^96^ software packages; a summary of the comparisons between Psi4 and Q-Chem is provided in Supporting Information. To assess the trends in the DFT data, the functionals were grouped according to the following categories, as adopted from Morgante and Peverati:^97^ group 0 (LDA, HF, LC, SE): local spin density approximations, Hartree-Fock, low-cost methods, and semiempirical methods; group 1 (GGA): generalized gradient approximations and nonseparable gradient approximations; group 2 (mGGA): meta-GGA and meta-NGA functionals; group 3 (GH-GGA): global hybrid GGA and NGA functionals; group 4 (GH-mGGA): global hybrid meta-GGA and meta-NGA functionals; group 5 (RSH): long-range corrected hybrid functionals; group 6 (DH): double-hybrid functionals. These groupings are in general accord with commonly assigned rungs of the DFT ladder but do not necessitate that each functional belongs to only one category.

## 3 Results and Discussion

The AE-CCSDTQ(P)/CBS + MVD1 results now available^74^ for HCNO provide high-accuracy bond distances and harmonic vibrational frequencies as benchmark data for this vexing molecule with a linear equilibrium structure. Calibrations of this theoretical method against rigorous spectroscopic parameters for HCN indicate that our *r*_e_ and *ω*_*i*_ benchmarks for fulminic acid are accurate to 10^–4^ Å and 1.6 cm^–1^, respectively.^74^ Moreover, the HCN + O(^3^*P*) → HCNO reaction energy [Δ*E*_e_(rxn) = –*D*_e_(HCN–O)] converged to the AE-CCSDTQP(H)/CBS + MVD1 level via a composite focal-point analysis (FPA) scheme yields a thermochemical target accurate to about 0.1 kcal mol^–1^ for a challenging homolytic bond cleavage.^74^ Theoretical chemists would thus consider our HCNO benchmarks as having “spectroscopic accuracy.”

Our HCNO standards^74^ have special chemical significance beyond the quasilinear/quasibent issue. In particular, fulminic acid exhibits one of the shortest C–H bond lengths and highest C–H stretching frequencies known in organic chemistry, and *D*_e_(HCN–O) proves important in pinpointing the enthalpies of formation of the classic [H,C,N,O] isomers.^62,72,74^ Finally, the shifts in bond distances (Δ*r*_e_) and vibrational frequencies (Δω) in going from HCN to HCNO are useful probes of the chemical bonding environment in these compounds. Historically, such shifts in properties are understood to be less sensitive to level of theory than the values themselves; hence, an interesting test is whether quick convergence to our benchmark values occurs as the DFT ladder is climbed.

An exhaustive tabulation of results for all 473 DFAs investigated here is provided in the Supporting Information (Tables S2–S6) for the following properties: *r*_e_(H–C), *r*_e_(C–N), *r*_e_(N–O), ω_1_-ω_5_, Δ*r*_e_(H–C), Δ*r*_e_(C–N), Δω(H–C str.), Δω(C–N str.), and Δ*E*_e_(rxn), as well as ω_6_, θ_e_(H–C–N), θ_e_(C–N–O), and the barrier to linearity (*E*_B_) for bent structures. In addition, percentiles (%-ile) in accuracy within the collection of DFAs relative to our benchmarks are listed for each property except the last four. If a linear transition state is found for a given DFA, the corresponding bond distances and frequencies are also given.

**Figure 1:**
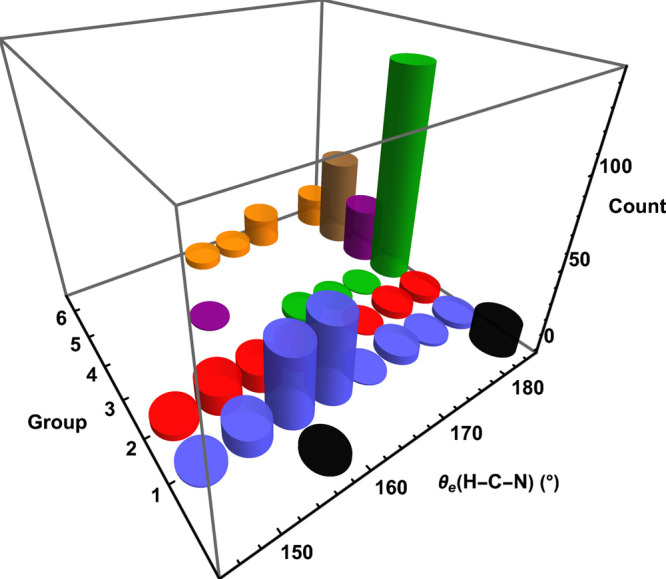
Histogram of fulminic acid θ_e_(H–C–N) values predicted by each group of DFAs.

**Figure 2:**
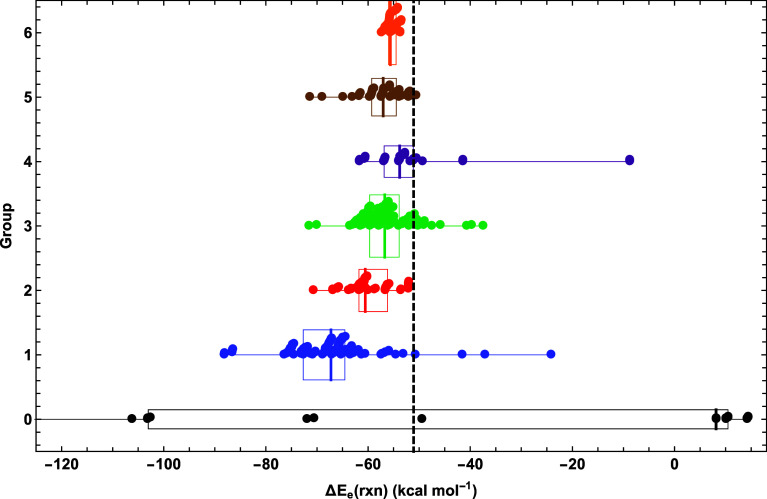
Scatter plot of DFA predictions for Δ*E*_e_ of the HCN + O(^3^*P*) → HCNO reaction sorted by group hierarchy. For each group, a box and whisker plot shows the maximum, minimum, quartiles, and median of the data set. The vertical dotted line is placed at the essentially exact Δ*E*_e_ value.^74^

**Figure 3:**
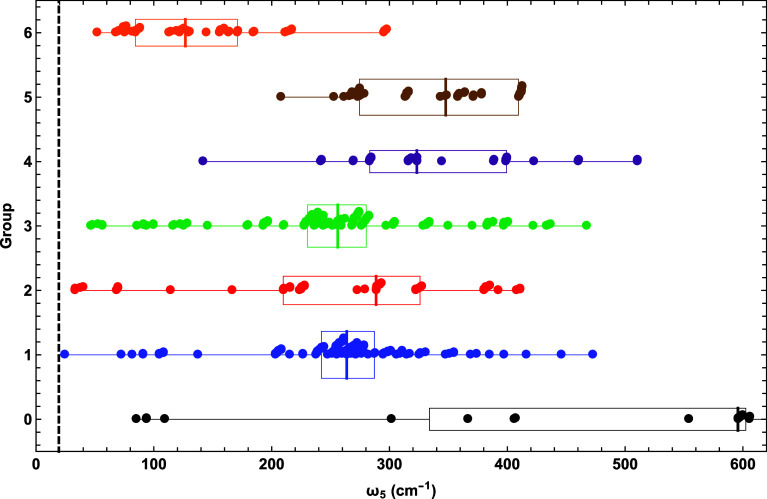
Scatter plot of fulminic acid ω_5_(H–C–N bend) predictions for each DFA group, overlaid with box and whisker representations of the data. See the caption of Figure 2 for further explanation.

**Figure 4:**
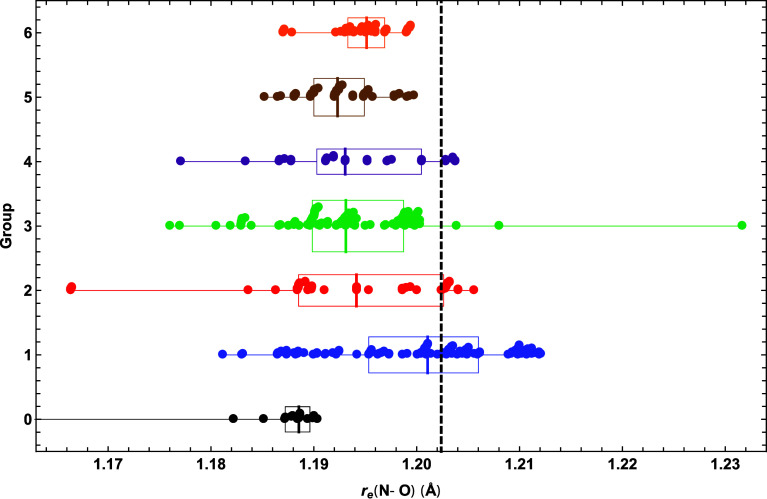
Scatter plot of fulminic acid *r*_e_(N–O) predictions for each DFA group, overlaid with box and whisker representations of the data. See the caption of Figure 2 for further explanation.

**Figure 5:**
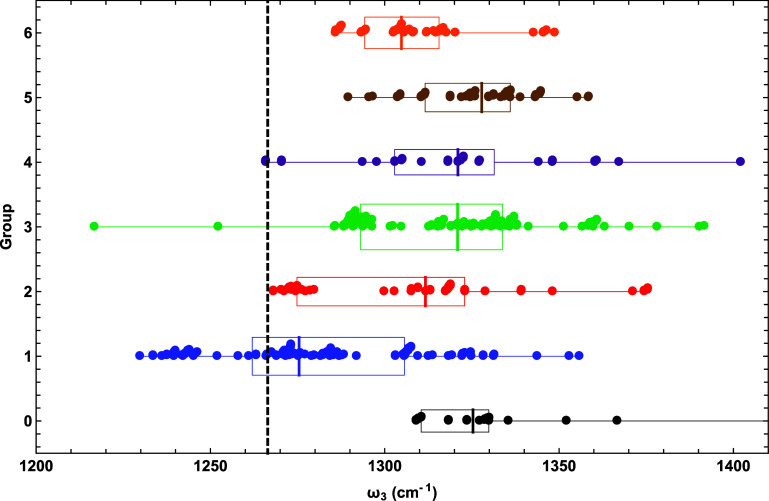
Scatter plot of fulminic acid ω_3_ predictions for each DFA group, overlaid with box and whisker representations of the data. See the caption of Figure 2 for further explanation.

**Figure 6:**
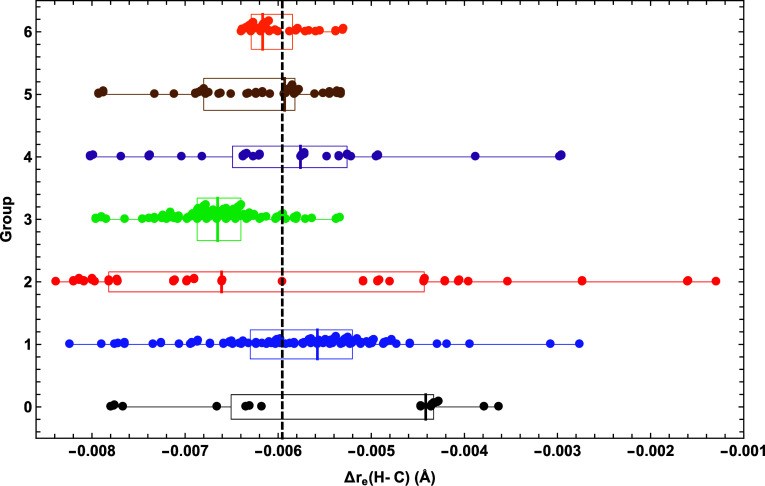
Scatter plot of Δ*r*_e_(H–C) predictions going from HCN to HCNO for each DFA group, overlaid with box and whisker representations of the data. See the caption of Figure 2 for further explanation.

**Figure 7:**
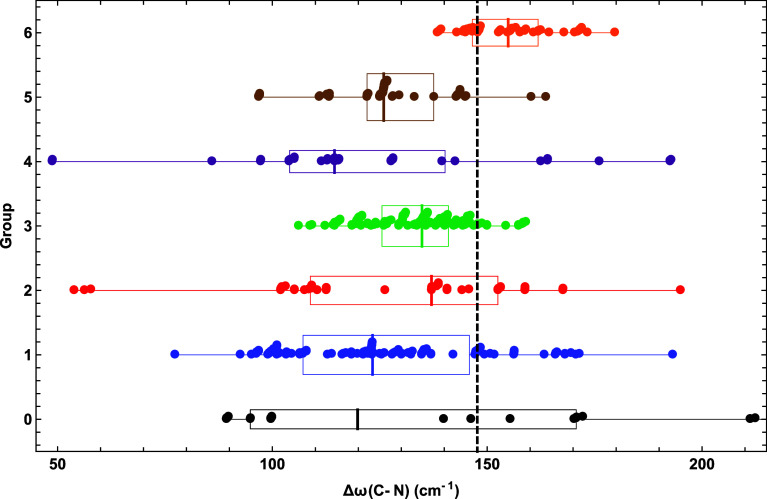
Scatter plot of Δω(C–N str.) predictions going from HCN to HCNO for each DFA group, overlaid with box and whisker representations of the data. See the caption of Figure 2 for further explanation.

For all of the aforementioned properties, data for all DFAs employed are displayed in scatter plots or histograms sorted by group hierarchy. For visualization of predicted HCNO equilibrium bond angles, a 3D histogram is presented in Figure 1 for θ_e_(H–C–N) and Figure S1 for θ_e_(C–N–O). For the scatter plots of each group, a box and whisker plot is overlaid showing the maximum, minimum, median, and quartiles for each group (five-number summary), and the corresponding benchmark result for each property is displayed with a dashed, vertical line. Scatter plots for Δ*E*_e_(rxn), *r*_e_(N–O), ω_3_, ω_5_, Δ*r*_e_(H–C), and Δω(C–N str.) are provided in Figures 2–7, whereas scatter plots for *r*_e_(H–C), *r*_e_(C–N), ω_1_, ω_2_, ω_4a_, ω_4b_, Δ*r*_e_(C–N), and Δω(H–C str.) appear in Figures S2–S9. Results from a pruned subset of 34 functionals have been selected for presentation and discussion in the main text. This subset comprises DFAs cited for robust performance by Goerigk and coworkers,^81^ Mardirossian and Head-Gordon,^82^ and Verma and Truhlar,^85^ as well as a majority of the most popular functionals per a 2023 DFT poll organized by Swart, Bickelhaupt, and Duran.^98^ For this pruned subset, we present Tables 1 and 2 detailing optimized bond distances and harmonic vibrational frequencies for linear and bent HCNO minima. For DFAs that produced a bent minimum, the barrier to linearity, optimized structure, and frequencies of the linear HCNO transition state are provided in Table 3. Table 4 lists values for Δ*E*_e_(rxn) along with percentiles for the Δ*E*_e_(rxn), *r*_e_, and Δ*r*_e_ quantities, while Table 5 gives analogous results for the vibrational frequencies.

**Table 1:** Linear HCNO Optimized Bond Distances (*r*_e_, Å) and Harmonic Vibrational Frequencies (ω_*i*_, cm^–1^)

Method	Group	*r*_e_(H–C)	*r*_e_(C–N)	*r*_e_(N–O)	ω_1_(σ)	ω_2_(σ)	ω_3_(σ)	ω_4_(π)	ω_5_(π)
LDA0	0	1.0661	1.1515	1.1851	3470	2374	1352	589	301
MN12-L	2	1.0565	1.1468	1.1885	3545	2375	1318	584	33
B3LYP-D3(BJ)2B	3	1.0590	1.1542	1.1989	3498	2322	1291	565	238
PBE0	3	1.0609	1.1528	1.1901	3513	2366	1336	582	269
SOGGA11-X-D3(BJ)2B	3	1.0597	1.1497	1.1931	3546	2386	1334	590	331
TPSSh	3	1.0600	1.1582	1.1998	3497	2312	1289	564	52
M05-2X-D3(0)2B	4	1.0582	1.1468	1.1931	3538	2375	1327	590	389
M06-2X-D3(0)2B	4	1.0600	1.1477	1.1952	3511	2369	1318	593	399
PW6B95-D3(BJ)2B	4	1.0558	1.1499	1.1911	3529	2358	1322	580	283
CAM-B3LYP	5	1.0597	1.1475	1.1952	3504	2360	1311	580	358
HSE06	5	1.0604	1.1525	1.1900	3512	2363	1333	580	266
M11	5	1.0645	1.1492	1.1924	3474	2354	1325	595	412
MN12-SX	5	1.0603	1.1471	1.1948	3523	2365	1304	578	275
MN15	5	1.0606	1.1530	1.1978	3520	2349	1319	580	378
ωB97M-V	5	1.0595	1.1497	1.1990	3505	2346	1297	572	347
ωB97X-D3	5	1.0606	1.1495	1.1928	3513	2367	1326	582	343
ωB97X-V	5	1.0629	1.1507	1.1982	3505	2359	1311	579	363
B2GP-PLYP-D3(BJ)2B	6	1.0557	1.1585	1.1957	3537	2303	1305	567	122
AE-CCSDTQ(P)/CBS + MVD1^74^		1.0590	1.1588	1.2024	3497	2275	1266	548	19

**Table 2:** *Trans*-Bent HCNO Optimized Bond Distances (*r*_e_, Å), Bond Angles (*θ*, °), and Harmonic Vibrational Frequencies (ω_*i*_, cm^–1^)

Method	Group	*r*_e_(H–C)	*r*_e_(C–N)	*r*_e_(N–O)	θ_HCN_	θ_CNO_	ω_1_(*a*′)	ω_2_(*a*′)	ω_3_(*a*′)	ω_4_(*a*′)	ω_5_(*a*′)	ω_6_(*a*″)
B97-D3(BJ)	1	1.0646	1.1694	1.1967	159.0	175.2	3424	2256	1285	546	273	547
B97M-V	1	1.0548	1.1566	1.1903	161.0	175.3	3540	2338	1322	572	238	572
BLYP-D3(BJ)2B	1	1.0663	1.1728	1.2100	159.6	175.4	3393	2213	1243	532	267	532
BP86	1	1.0693	1.1736	1.2032	158.8	175.2	3389	2238	1273	541	270	541
PBE	1	1.0689	1.1728	1.2010	160.2	175.6	3403	2255	1284	545	255	546
revPBE	1	1.0702	1.1773	1.2044	156.3	174.5	3388	2230	1272	540	306	541
revPBE-D3(BJ)2B	1	1.0700	1.1773	1.2042	156.0	174.5	3389	2230	1273	540	310	541
M06-L-D3(0)2B	2	1.0577	1.1608	1.1898	163.0	175.8	3511	2348	1339	583	210	584
MGGA_MS1	2	1.0630	1.1673	1.2000	150.0	172.4	3454	2247	1277	541	392	537
MGGA_MS2	2	1.0635	1.1680	1.1994	149.0	172.1	3445	2245	1280	539	408	535
MN12-L	2	1.0567	1.1471	1.1884	175.3	178.8	3543	2373	1319	583	69	583
MN15-L	2	1.0688	1.1660	1.1986	160.7	175.0	3494	2336	1323	576	225	576
revM06-L	2	1.0564	1.1490	1.1863	170.5	177.8	3534	2395	1348	592	114	592
revTPSS-D3(BJ)2B	2	1.0663	1.1722	1.2029	154.7	174.0	3413	2240	1275	549	327	550
SCAN-D3(BJ)2B	2	1.0628	1.1629	1.1941	157.8	174.3	3455	2293	1303	557	272	552
DSD-BLYP-D3(BJ)	6	1.0560	1.1610	1.1952	173.8	178.6	3534	2292	1307	565	84	568
DSD-PBEP86-D3(BJ)	6	1.0599	1.1645	1.1950	167.3	177.2	3507	2283	1308	563	171	562

**Table 3:** Linear HCNO Transition State Optimized Bond Distances (*r*_e_, Å), Harmonic Vibrational Frequencies (*ω*_*i*_, cm^–1^), and Barriers to Linearity (*E*_B_, cm^–1^)

Method	Group	*E*_B_	*r*_e_(H–C)	*r*_e_(C–N)	*r*_e_(N–O)	ω_1_(σ)	ω_2_(σ)	ω_3_(σ)	ω_4_(π)	ω_5_(π)
B97-D3(BJ)	1	32.18	1.0622	1.1645	1.1987	3461	2280	1281	547	197*i*
B97M-V	1	19.21	1.0527	1.1523	1.1923	3574	2360	1316	572	169*i*
BLYP-D3(BJ)2B	1	28.84	1.0640	1.1682	1.2120	3428	2235	1237	532	192*i*
BP86	1	33.44	1.0668	1.1687	1.2052	3426	2260	1268	543	192*i*
PBE	1	25.43	1.0668	1.1686	1.2027	3434	2274	1280	546	184*i*
revPBE	1	52.18	1.0671	1.1711	1.2069	3434	2259	1266	542	222*i*
revPBE-D3(BJ)2B	1	55.31	1.0668	1.1709	1.2067	3436	2260	1267	541	225*i*
M06-L-D3(0)2B	2	11.88	1.0560	1.1573	1.1912	3537	2365	1336	584	147*i*
MGGA_MS1	2	141.38	1.0576	1.1558	1.2057	3534	2306	1257	540	291*i*
MGGA_MS2	2	165.44	1.0576	1.1558	1.2053	3530	2307	1259	538	307*i*
MN15-L	2	18.77	1.0665	1.1613	1.2008	3528	2361	1316	577	163*i*
revM06-L	2	1.05	1.0559	1.1480	1.1868	3542	2401	1346	592	79*i*
revTPSS-D3(BJ)2B	2	67.66	1.0628	1.1646	1.2062	3466	2277	1266	550	237*i*
SCAN-D3(BJ)2B	2	51.93	1.0601	1.1571	1.1965	3497	2324	1297	561	225*i*
DSD-BLYP-D3(BJ)	6	0.23	1.0558	1.1606	1.1954	3536	2298	1306	565	57*i*
DSD-PBEP86-D3(BJ)	6	4.32	1.0591	1.1625	1.1959	3518	2291	1306	564	116*i*

**Table 4:** Reaction Energies [Δ*E*_e_(rxn), kcal mol^–1^], HCNO Bond Distance Shifts Relative to HCN (Δ*r*_e_, Å), and Corresponding Accuracy Percentiles (%-ile)

			%-ile	%-ile	%-ile	%-ile		%-ile		%-ile
Method	Group	Δ*E*_e_(rxn)	Δ*E*_e_(rxn)	*r*_e_(H–C)	*r*_e_(C–N)	*r*_e_(N–O)	Δ*r*_e_(H–C)	Δ*r*_e_(H–C)	Δ*r*_e_(C–N)	Δ*r*_e_(C–N)
LDA0	0	–72.0	11.3	21.5	54.0	6.3	–0.0067	45.3	0.0095	45.9
B97-D3(BJ)	1	–63.8	26.9	30.6	38.5	66.3	–0.0057	82.0	0.0153	20.5
B97M-V	1	–57.5	54.3	37.3	89.8	30.2	–0.0054	61.0	0.0118	35.5
BLYP-D3(BJ)2B	1	–65.7	22.5	20.2	20.2	55.6	–0.0052	41.3	0.0163	11.5
BP86	1	–72.5	10.9	8.1	16.7	95.4	–0.0054	58.9	0.0162	15.7
PBE	1	–74.8	8.1	9.8	20.0	91.9	–0.0060	98.3	0.0154	19.4
revPBE	1	–66.4	20.3	5.0	9.6	87.3	–0.0052	43.2	0.0169	7.9
revPBE-D3(BJ)2B	1	–67.5	17.5	6.0	9.2	88.8	–0.0052	43.6	0.0169	6.9
M06-L-D3(0)2B	2	–61.8	32.4	75.6	91.5	24.4	–0.0077	11.3	0.0116	36.3
MGGA_MS1	2	–53.7	85.6	37.9	50.4	84.6	–0.0016	0.2	0.0189	1.3
MGGA_MS2	2	–55.9	70.1	34.8	45.8	80.6	–0.0013	0.0	0.0196	0.8
MN12-L^a^	2	–52.1	94.2	54.2	27.7	17.7	–0.0081	3.3	0.0059	99.0
MN15-L	2	–56.3	66.4	11.7	54.4	72.7	–0.0078	9.6	0.0096	45.3
revM06-L	2	–58.8	49.7	52.9	41.7	6.7	–0.0080	6.1	0.0096	45.7
revTPSS-D3(BJ)2B	2	–60.5	42.2	19.8	24.2	97.1	–0.0041	9.2	0.0175	3.8
SCAN-D3(BJ)2B	2	–60.8	39.2	40.2	79.0	51.5	–0.0044	15.9	0.0163	12.1
B3LYP-D3(BJ)2B	3	–56.5	64.9	99.6	73.8	75.4	–0.0065	60.1	0.0091	50.1
PBE0	3	–59.6	46.6	62.9	64.2	29.2	–0.0068	35.7	0.0082	74.9
SOGGA11-X-D3(BJ)2B	3	–52.4	91.2	89.2	45.4	44.2	–0.0068	37.0	0.0050	91.4
TPSSh	3	–56.1	68.7	80.2	95.2	83.3	–0.0072	20.5	0.0090	52.0
M05-2X-D3(0)2B	4	–51.1	99.4	86.7	26.7	43.8	–0.0049	26.5	0.0077	82.0
M06-2X-D3(0)2B	4	–52.8	90.0	79.2	37.5	59.0	–0.0057	85.0	0.0066	90.0
PW6B95-D3(BJ)2B	4	–56.9	60.1	47.1	47.1	32.5	–0.0064	68.7	0.0086	63.9
CAM-B3LYP	5	–54.3	81.4	87.1	34.8	58.8	–0.0058	87.9	0.0079	80.0
HSE06	5	–59.1	48.9	71.5	61.5	27.7	–0.0068	38.6	0.0083	68.9
M11	5	–55.7	73.7	31.3	42.3	38.5	–0.0053	53.0	0.0101	40.7
MN12-SX	5	–52.1	94.8	74.6	29.8	55.0	–0.0079	8.1	0.0053	94.2
MN15	5	–57.0	59.3	67.7	66.5	69.6	–0.0054	62.0	0.0080	77.2
ωB97M-V	5	–51.8	96.5	90.8	46.3	77.1	–0.0055	66.6	0.0063	93.3
ωB97X-D3	5	–54.8	78.5	66.0	44.4	41.5	–0.0063	71.8	0.0069	88.1
ωB97X-V	5	–53.6	86.0	39.2	51.7	71.0	–0.0061	91.0	0.0060	98.1
B2GP-PLYP-D3(BJ)2B	6	–53.7	85.4	45.6	97.3	63.5	–0.0062	84.1	0.0087	59.1
DSD-BLYP-D3(BJ)	6	–54.8	79.1	50.0	90.0	60.0	–0.0060	93.9	0.0093	48.0
DSD-PBEP86-D3(BJ)	6	–55.4	76.0	84.0	67.9	57.9	–0.0057	80.0	0.0106	37.6
AE-CCSDTQ(P)/CBS + MVD1^74^		–51.1^b^	100	100	100	100	–0.0059	100	0.0057	100

a Linear minimum.

b Includes a CCSDTQ(P) → CCSDTQP(H) increment and a spin–orbit correction (SOC).

**Table 5:** Percentiles for HCNO Vibrational Frequencies and Shifts (Δω, cm^–1^) Relative to HCN

Method	Group	%-ile ω_1_	%-ile ω_2_	%-ile ω_3_	%-ile ω_4_	Δω(C–H)	%-ile Δω(C–H)	Δω(C–N)	%-ile Δω(C–N)
LDA0	0	52.3	16.0	7.1	17.7	54.3	90.8	146.2	95.0
B97-D3(BJ)	1	25.2	88.8	84.4	95.8	38.0	30.1	127.8	48.9
B97M-V	1	40.6	51.0	34.4	45.6	42.5	37.2	128.3	50.3
BLYP-D3(BJ)2B	1	12.7	51.3	74.6	61.9	33.5	19.6	101.0	9.6
BP86	1	9.0	72.7	93.1	86.7	34.9	21.9	122.9	33.6
PBE	1	17.5	87.5	85.6	95.2	43.1	38.6	134.8	62.6
revPBE	1	7.5	65.4	94.2	85.0	31.7	15.4	123.2	36.1
revPBE-D3(BJ)2B	1	8.5	66.3	92.9	83.8	31.2	13.4	123.1	34.9
M06-L-D3(0)2B	2	75.4	43.1	12.5	22.5	70.1	25.7	167.6	48.2
MGGA_MS1	2	40.4	81.5	88.3	85.8	–14.7	0.2	57.7	1.0
MGGA_MS2	2	34.2	79.2	87.7	80.6	–19.2	0.0	56.2	0.8
MN12-L^a^	2	35.4	14.0	39.6	21.5	81.8	7.3	138.6	73.7
MN15-L	2	91.3	52.5	32.3	41.9	56.9	73.5	140.6	81.0
revM06-L	2	46.0	7.5	8.1	13.1	79.9	8.8	144.1	89.1
revTPSS-D3(BJ)2B	2	22.7	76.5	90.0	98.5	18.0	3.1	102.0	11.3
SCAN-D3(BJ)2B	2	41.0	89.0	61.7	82.3	25.2	7.7	108.4	16.9
B3LYP-D3(BJ)2B	3	98.8	64.0	73.3	60.2	55.9	79.3	120.6	29.6
PBE0	3	71.9	25.2	16.0	27.7	59.1	59.5	145.1	92.1
SOGGA11-X-D3(BJ)2B	3	34.8	10.4	19.2	17.1	57.9	66.8	139.0	75.6
TPSSh	3	98.5	72.9	77.5	65.8	65.2	34.4	135.7	65.1
M05-2X-D3(0)2B	4	42.3	14.6	27.3	16.0	34.5	20.9	104.1	13.8
M06-2X-D3(0)2B	4	75.6	21.5	40.8	12.9	44.4	43.8	112.7	20.9
PW6B95-D3(BJ)2B	4	48.5	35.0	35.6	35.0	55.1	85.2	128.1	49.9
CAM-B3LYP	5	86.3	33.1	48.8	36.5	46.3	50.7	113.1	21.7
HSE06	5	72.7	29.2	19.4	34.0	57.5	69.7	142.7	84.8
M11	5	58.3	37.3	30.2	9.2	32.7	17.5	97.0	5.4
MN12-SX	5	54.4	25.8	59.0	40.2	68.6	29.9	126.1	44.5
MN15	5	59.8	42.5	38.8	35.4	41.8	35.9	122.0	31.5
ωB97M-V	5	84.4	44.6	64.0	46.3	47.8	62.0	110.9	18.8
ωB97X-D3	5	72.1	23.5	29.0	26.7	53.0	99.0	129.5	52.6
ωB97X-V	5	83.5	33.5	49.0	37.5	50.6	79.7	122.0	31.3
B2GP-PLYP-D3(BJ)2B	6	43.5	81.7	58.3	54.6	51.8	88.3	155.4	79.1
DSD-BLYP-D3(BJ)	6	45.8	90.0	53.5	59.4	51.0	80.8	161.9	60.1
DSD-PBEP86-D3(BJ)	6	79.6	95.8	51.9	70.0	49.9	75.8	171.6	37.6

a Linear minimum.

### 3.1 Bond Angles

The most salient issue in assessing DFA performance for fulminic acid is whether the predicted equilibrium geometry is linear or bent, or more broadly what values are given for θ_e_(H–C–N) and θ_e_(C–N–O). Here, we discuss in detail the data for θ_e_(H–C–N), which are portrayed in Figure 1. Companion statistics are the percentage of DFAs within each group that produce a linear structure: (group, % linear) = (0, 90.0), (1, 3.3), (2, 11.3), (3, 95.0), (4, 97.3), (5, 100), and (6, 40.4). Thus, the hybrid DFAs (groups 3–5) correctly yield linear minima for all but a handful of functionals, and group 5 produces linear structures exclusively. The smallest θ_e_(H–C–N) values witnessed for groups (3, 4) are (169, 158)° by (MPWLYP1M, MGGA_MS2h). On the contrary, the group 1 GGAs and group 2 meta-GGAs give much broader distributions of θ_e_(H–C–N). Group 1 has a median θ_e_(H–C–N) = 160° with an interquartile range (IQR) = 2.9°, while its minimum angle is 148° from MOHLYP2. The distribution of group 1 shows a clustering of θ_e_(H–C–N) results around 160°, which is not observed in the more scattered profile of group 2. For group 2, the median θ_e_(H–C–N) = 158° with an IQR = 8.7°, and the smallest θ_e_(H–C–N) across all DFAs is 146° from PKZB-D3(0)2B of this group. Less than half of group 6 functionals correctly produce linear minima. The group 6 minimum value for θ_e_(H–C–N) is 163° from PTPSS-D3(BJ)2B, and several DFAs are clustered around 175°. The essential observations from Figure 1 are that groups 1 and 2 have an erroneous propensity for bent minima, groups 3–5 rectify this failure until no bent structures occur for group 5, but the ladder returns at group 6 back to a preponderance of bent minima.

When assessing θ_e_(H–C–N) within our pruned lists in Tables 1 and 2, the same trends emerge. Excluding MN12-L, all 14 DFAs in groups 1 and 2, namely the GGAs and meta-GGAs, produce bent minima while all 15 hybrid functionals (groups 3–5) produce linear minima. Among the three group 6 double-hybrids, B2GP-PLYP-D3(BJ)2B produces a linear minimum, while DSD-BLYP-D3(BJ) and DSD-PBEP86-D3(BJ) produce bent structures with tiny *E*_B_ values of 0.23 and 4.32 cm^–1^, respectively (Table 3). For groups 1–5, the percentage of DFAs producing linear minima shows a continuous increase as the DFT ladder is climbed; however, at the top of the ladder, the group 6 double-hybrids only produce a linear structure in 33% of cases in the pruned list. It is thus substantiated that these DFT methods do not yield any consensus or convergence on the “delicate” ω_5_ bending frequency and thus do not provide insight into the intricacies of the fulminic acid quasibent/quasilinear problem.

### 3.2 Reaction Energy

With a mean error in Δ*E*_e_(rxn) of −7.5 kcal mol^–1^ over all functionals, the DFAs show a propensity to predict a reaction energy that is too exergonic, as seen in Figure 2. Within our pruned sample set (Table 4), all DFAs but one overestimate the exergonic nature of this reaction, some by merely 0.7 kcal mol^–1^ but others by as much as 23 kcal mol^–1^. Figure 2 reveals that the DFT ladder is rather successful in converging on a reasonably precise Δ*E*_e_(rxn) value, but the final group result displays a systematic error of almost 5 kcal mol^–1^. In particular, the group 6 double-hybrid functionals are clearly the best performing group, as all results differ from the benchmark by at most 6.3 kcal mol^–1^ [DSD-PBEB95] and at least 2.4 kcal mol^–1^ [B2GP-PLYP]. With a median of –53.8 kcal mol^–1^, the global hybrid meta-GGA (group 4) functionals come closest to the Δ*E*_e_(rxn) benchmark, but poor results (−9 kcal mol^–1^) from DLDF, DLDF+D09, and DLDF+D10 contribute to a large standard deviation of 13.5 kcal mol^–1^. Aside from this irregularity, the data spread shows remarkable improvement as rungs on the DFT ladder are climbed. Group 1 has an IQR of 8.2 kcal mol^–1^, which is improved to (5.6, 5.8, 5.7, 4.8) kcal mol^–1^ for groups 2–5 and finally decreased to merely 1.5 kcal mol^–1^ for group 6.

The three double-hybrid functionals [B2GP-PLYP-D3(BJ)2B, DSD-BLYP-D3(BJ), DSD-PBEP86-D3(BJ)] included in the pruned sample set (Table 4) are in the (85.4, 79.1, 76.0)%-iles for Δ*E*_e_(rxn). In comparison, among the three group 4 DFAs included in Table 4, M05-2X-D3(0)2B is a star performer with 99.4%-ile for Δ*E*_e_(rxn), the best in the pruned sample set; M06-2X-D3(0)2B also performs quite well (90.0%-ile) followed by a reasonable prediction from PW6B95-D3(BJ)2B (60.1%-ile). The group 5 long-range corrected hybrids and group 2 meta-GGAs yield comparable data clustering, exhibiting the smallest ranges and no egregious outliers. For groups 2 and 5, the medians are −60.5 and −57.0 kcal mol^–1^ respectively, showing that group 5 is significantly more accurate. The group 3 hybrid GGAs demonstrate comparable statistical performance (median = −56.7 kcal mol^–1^) but have more DFAs predicting Δ*E*_e_(rxn) to be too endergonic, a trend unique to this group. For group 2, the (min, max) values are (−70.7, −52.1) kcal mol^–1^ with no DFAs exceeding the true value. In comparison, for group 3 (min, max) = (−71.6, −37.5) kcal mol^–1^, and for group 5 (min, max) = (−71.4, −50.6) kcal mol^–1^. Of the eight group 2 DFAs in Table 4, MN12-L predicts Δ*E*_e_(rxn) especially well, making it to 94.2%-ile. From the four group 3 DFAs in Table 4, only SOGGA-11-X-D3(BJ)2B is above 90%-ile, while two of the eight group 5 DFAs surpass this threshold, namely MN12-SX (94.8%-ile) and ωB97M-V (96.5%-ile). Across groups 3–6, only 2 of the 18 results for DFAs in Table 4 are below 50%-ile, in sharp contrast to group 1. In summary, the overall DFT performance highlighted in Figure 2 shows mixed success of the DFT ladder, as the precision of the Δ*E*_e_(rxn) results improves markedly but the medians of groups (4, 5, 6) remain systematically inaccurate by (2.7, 5.9, 4.5) kcal mol^–1^.

### 3.3 Bending Frequencies

The hallmark of fulminic acid is its extremely flat H–C–N bending potential with a nearly vanishing harmonic vibrational frequency at linearity. Fluctuations in the H–C–N bending frequency^99^ are so sensitive that even robust CCSDT(Q)/CBS computations fail to produce a linear minimum for HCNO. Nonetheless, given the aforementioned optimism in using modern DFT methods to probe the properties of fulminic acid,^76^ it is critical to assess performance on the H–C–N bending vibration.

Figure 3 depicts a scatter plot of the associated ω_5_, which is the lowest frequency given by each DFA regardless of whether the predicted minimum is linear or bent. Clearly, no group produces a ω_5_ median within even 100 cm^–1^ of the 19 cm^–1^ benchmark. The medians of groups 1–3 are (264, 289, 256) cm^–1^, whereas the medians of groups 4 and 5 backtrack to (323, 348) cm^– 1^. However, group 6 substantially improves the ω_5_ median to 127 cm^–1^, an instance where the top rung of the DFT ladder generally does yield the best results, albeit still far above the true value. The group 1 functional TH-FL gives a frequency closest to the benchmark (ω_5_ = 24 cm^–1^). The smallest ω_5_ results for groups 2–6 are as follows: [MN12-L-D3(BJ)ATM, 33 cm^–1^], [TPSSh-D3(BJ)2B, 46 cm^–1^], [τ-HCTHh, 142 cm^–1^], [tuned-CAM-B3LYP, 208 cm^–1^], and [DSD-PBEB95, 52 cm^–1^]. All groups have a maximum ω_5_ over 297 cm^–1^ [group 6, PBE0-DH-D3(0)2B], and the most egregious value within groups 1–6 is ω_5_ = 510 cm^–1^ [group 4, M06-HF-D3(0)ATM]. For groups 1–6, IQR = (45, 116, 50, 116, 135, 87) cm^–1^, showing that groups 1 and 3 have considerably less spread by this measure than the other four groups and that group improvement fails to tighten the distribution.

From the pruned subset of DFAs that produce a linear minimum (Table 1), only two result in ω_5_ less than 100 cm^–1^. The group 3 meta-GGA TPSSh predicts ω_5_(π) = 52 cm^–1^, whereas the ω_5_(π) = 33 cm^–1^ result from MN12-L (group 3, meta-GGA) is closest to the benchmark. However, MN12-L also produces a bent minimum with a total energy essentially indistinguishable from that of its linear structure, and ω_5_(*a*′) = 69 cm^–1^ for this bent form. The only other DFA in Table 2 that produces an H–C–N bending frequency below 100 cm^–1^ is the group 6 double-hybrid DSD-BLYP-D3(BJ), for which ω_5_(*a*′) = 84 cm^–1^ and the barrier to linearity (*E*_B_) is merely 0.23 cm^–1^ (Table 3). The overall picture of the data afforded by Figure 3 is that the DFAs fail almost universally to predict a sufficiently small ω_5_ and to properly describe the flatness of the bottom of the H–C–N bending potential.

### 3.4 Bond Distances

The scatter plot (Figure 4) for *r*_e_(N–O) has been chosen for selective discussion of HCNO optimized bond distances. All DFA groups have a median *r*_e_(N–O) shorter than the benchmark, and only a handful of DFAs in groups 3–6 (hybrid and double-hybrid functionals) predict an *r*_e_(N–O) longer than its true value. Only the group 1 GGAs have a significant number of members exceeding the benchmark *r*_e_(N–O). Surprisingly, group 1 produces the most accurate median (1.2011 Å), in error by only −0.0013 Å. The medians for groups 2–5 are (1.1942, 1.1931, 1.1930, 1.1923) Å, which successively regress from the target. However, the group 6 double-hybrids push the median forward to 1.1951 Å, the second most accurate value but still −0.0073 Å away from the true distance. For groups 1–6, the IQRs are (0.0107, 0.0141, 0.0089, 0.0102, 0.0049, 0.0036) Å, showing a general decrease in the breadth of the distribution except for a blip in this trend by group 4. In group 3, XB1K dramatically overshoots the benchmark by 0.03 Å, presumably because this DFA is calibrated for thermochemical kinetics and weak interactions and is not sufficiently robust. Similarly, group 2 contains a clustering of bad outliers with errors of −0.04 Å arising from M11-L and its associated dispersion variants. Aside from the outliers in groups 2 and 3, all other DFA results excluding group 0 lie in the 1.1760–1.2121 Å range. While physically reasonable, such bond distances are too inaccurate to solve problems in chemical bonding theory or spectroscopy.

Within the pruned subset of DFAs (Table 4), only three surpass 90%-ile for *r*_e_(N–O). Two are group 1 GGAs [(BP86, 95.4%-ile), (PBE, 91.9%-ile)] and one is a group 2 meta-GGA [revTPSS-D3(BJ)2B, 97.1%-ile]. However, these three DFAs yield poor predictions for *r*_e_(H–C) and *r*_e_(C–N), not exceeding 25%-ile in any instance. From Table 4, the two DFAs with the highest %-ile average across all bond distances were both in group 3: [B3LYP-D3(BJ)2B, 82.9%-ile], (TPSSh, 86.2%-ile). The essential point is that the correspondence between rung of DFT ladder and the accuracy of *r*_e_(N–O) predictions is poor. Indeed, the medians and IQRs in Figure 4 demonstrate that group 1 is the preferred choice for this quantity.

### 3.5 Stretching Frequencies

Our choice among HCNO stretching frequencies for specific discussion is ω_3_, whose normal mode mostly involves the N–O bond. Consistent with the expected relationship between bond distances and stretching frequencies, Figure 5 generally looks like a mirror image of Figure 4. The median ω_3_ for all groups is larger than the benchmark, and the group 1 median of 1275 cm^–1^ is closest to the true value of 1266 cm^–1^. The medians of groups 2–5 are (1312, 1321, 1321, 1328) cm^–1^, moving successively away from the benchmark. Group 6 yields a median of 1305 cm^–1^, which is a shift in the correct direction but is still nearly 40 cm^–1^ too large. The IQRs for groups 1–6 are (44, 48, 41, 29, 24, 21) cm^–1^, showing that groups 4–6 have significantly less spread than groups 1–3. Interestingly, the marked IQR reduction of the group 4 ω_3_ values compared to groups 1–3 is not found for *r*_e_(N–O). Likewise, group 2 demonstrates an isolated clustering of data closer to the benchmark that is not witnessed in the *r*_e_(N–O) plot, where the data are distributed more evenly. The maxima for groups 3 and 4 are 1392 and 1402 cm^–1^, considerable overestimations of 10 and 11% given by KMLYP and MGGA_MVSh, respectively. The group 3 outlier with an ω_3_ minimum value (1217 cm^–1^) much lower than the benchmark belongs to XB1K, which is consistent with the corresponding outlier in Figure 4.

In the pruned list of DFAs in Table 5, four functionals reach 90%-ile or above for ω_3_, two of which also achieved this performance threshold for *r*_e_(N–O). The group 1 (GGA) BP86 and group 2 (meta-GGA) revTPSS-D3(BJ)2B land at 93.1%-ile and 90.0%-ile for ω_3_, respectively, while also surpassing 95%-ile for *r*_e_(N–O). The group 1 functional revPBE and its dispersion variant revPBE-D3(BJ)2B give 94.2%-ile and 92.9%-ile predictions for ω_3_, whereas their *r*_e_(N–O) results land in the upper 80%-ile. The PBE functional (group 1) gave an *r*_e_(N–O) prediction at 91.9%-ile, but its performance is somewhat diminished for ω_3_ (85.6%-ile). As is the case for *r*_e_(N–O), the DFAs that work best for ω_3_ are not typically the best for ω_1_ and ω_2_. For all the aforementioned DFAs with high performances for ω_3_, their predictions for ω_1_ do not exceed 25%-ile, with three even below 10%-ile. These same DFAs give mediocre results for ω_2_, typically in the %-ile range of 65–76, although PBE does appear at 87.5%-ile for this property. In summary, ω_3_ is another property for which accuracy does not systematically improve as the DFT ladder is climbed. Instead, the first rung (group 1) showcases the most accurate median with subsequent steps only deteriorating ω_3_ in a statistical sense.

### 3.6 Bond Distance Shifts

As a clear demonstration of the bonding information that can be garnered from HCN → HCNO shifts in equilibrium distances, we single out Δ*r*_e_(H–C) for detailed analysis here. The dashed, vertical line in the scatter plot of Figure 6 shows that the exact value is Δ*r*_e_(H–C) = −0.00596 Å. This sizable negative value is rather surprising because the carbon atom in HCN is formally *sp* hybridized, and greater carbon *s*-character for a shorter σ bond is not possible in HCNO, at least according to the usual valence-bond paradigms. Therefore, Δ*r*_e_(H–C) exposes some complexities in covalent bonding that constitute an interesting test for DFAs. Visually, the median Δ*r*_e_(H–C) values for groups 1–4 oscillate around the benchmark. The group (1, 4) median shifts are (−0.0056, –0.0058) Å whereas those of groups (2, 3) are (−0.0066, −0.0067) Å. Group 5 predicts Δ*r*_e_(H–C) the best with a median of −0.0059 Å on top of the benchmark. Showing that quick convergence is not reached, group 6 predicts a median shift of −0.0062 Å, regressing back to the accuracy of group 4. Although the group 6 median does worsen compared to group 5, there is a clear reduction of stray results. The group 5 point farthest from the exact value is Δ*r*_e_ = −0.0079 Å [MN12-SX-D3(0)2B], while that of group 6 is Δ*r*_e_ = −0.0053 Å (PTPSS). The Δ*r*_e_(H–C) medians are closer to the benchmark than the *r*_e_(H–C) results (Figure S2) by approximately an order of magnitude for all groups but one. This affirms the pervasive understanding that shifts in properties are less affected by method than the properties themselves. Although group 0 is the most errant and unpredictable class for all the aforementioned properties, it actually produces fewer Δ*r*_e_(H–C) outliers than groups 1, 2, and 4, albeit with worse median performance. Moreover, for groups 1–6, the Δ*r*_e_(H–C) IQR = (0.0011, 0.0034, 0.0005, 0.0012, 0.0010, 0.0004) Å, whereas *r*_e_(H–C) IQR = (0.0051, 0.0074, 0.0026, 0.0048, 0.0032, 0.0017) Å. A key general feature of Figure 6 is the large reduction in spread as the DFT ladder is climbed. Groups 1, 2, and 4 have (min, max) = (−0.0082, –0.0028) Å, (−0.0084, −0.0013) Å, and (−0.0080, −0.0030) Å, respectively. In contrast, group 5 has (min, max) = (−0.0079, −0.0053) Å, and group 6 produces an even larger contraction in spread with (min, max) = (−0.0064, −0.0053) Å. With progressive improvements in both median and reduction of spread, Δ*r*_e_(H–C) is a case where the DFT ladder is largely successful.

Three DFAs in the pruned subset surpass 90%-ile for Δ*r*_e_(H–C) (Table 4). The group 1 PBE functional performs very well with 98.3%-ile for Δ*r*_e_(H–C), the best in the pruned list; however, the corresponding 19.4%-ile for Δ*r*_e_(C–N) is poor. The group 5 ωB97X-V functional boasts (91.0, 98.1)%-ile for [Δ*r*_e_(H–C), Δ*r*_e_(C–N)], by far the best overall performance in Table 4 across both bond distance shifts. For group 6, DSD-BLYP-D3(BJ) gives a [Δ*r*_e_(H–C), Δ*r*_e_(C–N)] performance of (93.9, 48.0)%-ile. Clearly, as found for *r*_e_(N–O), top performers for Δ*r*_e_(H–C) do not typically repeat a strong performance for Δ*r*_e_(C–N).

### 3.7 Stretching Frequency Shifts

Similarly to Δ*r*_e_, shifts in stretching frequencies from HCN to HCNO can also provide insight into chemical bonding. Figure 7 shows a scatter plot of Δω(C–N str.) predictions. The median Δω(C–N str.) results for groups 1–6 are (−25, −11, −13, −33, −22, +7) cm^–1^ away from the benchmark Δω(C–N str.) = 148 cm^–1^. Although groups 4 and 5 worsen the gains made by groups 2 and 3, group 6 substantially improves the median Δω(C–N str.) and is the most accurate group for this shift. As found for Δ*r*_e_(H–C), Δω(C–N str.) is less sensitive to method than the parent property. For groups 1–6, IQR = (39, 44, 15, 36, 16, 15) cm^–1^ for Δω(C–N str.), demonstrating vacillations in the spread of the distribution as the DFT rung increases. In fact, group 4 has the largest range in Δω(C–N str.), extending from 49 cm^–1^ [M06-HF-D3(0)ATM] to 193 cm^–1^ (M05) and exhibiting errors that are extreme for a frequency shift. In contrast, the group 6 Δω(C–N str.) values fall within 138–180 cm^–1^, the lower end given by PTPSS-D3(0)2B and the upper end by PBE0-2. Remarkably, the best performing functional in our pruned list in Table 5 for Δω(C–N str.) is LDA0 in Group 0! In fact, LDA0 predicts the [Δω(C–N str.), Δω(H–C str.)] pair of shifts to (95.0, 90.8)%-ile, whereas the corresponding performance for the group 3 functional PBE0 is (92.1, 59.5)%-ile. The only other DFA in Table 5 with a Δω(C–N str.) performance close to 90%-ile is revM06-L, but in this case the associated Δω(H–C str.) is only 8.8%-ile. A summary observation regarding the Δω(C–N str.) data in Figure 7 and Table 5 is that the DFT ladder achieves limited success after the group 4 letdown, but the overall performance along the way is fraught with inconsistency.

### 3.8 Performance Consistency

An analysis of consistency was conducted by first singling out the DFAs that were the best performers for each fulminic acid property discussed here. Because no functional was the top performer in more than one category, this pruning generated 13 different DFAs for 13 properties. Our analysis then asked the revealing question of how well the winner in one category performed for the other 12 properties. The accuracy percentiles from our consistency analysis are curated in Table 6. The group 3 global hybrid DFAs produce the highest number of category winners (6), followed by groups 1 and 2 with three apiece. Only one group 6 double-hybrid functional is a category winner. The most accurate Δ*E*_e_(rxn) prediction is by MPWB1K (group 3). This functional exhibits very poor performance across the *r*_e_ and ω_*i*_ quantities with a mean percentile of only 17.7, but the associated Δ*r*_e_ and Δω shifts are surprisingly good. Although not a particularly robust method, MPWB1K was optimized against a kinetics database for thermochemical applications, so its performance is consistent with expectations.^101^ Developed in tandem with MPWB1K was the group 3 functional XB1K,^101^ which appears in Table 6 as the top performer for ω_5_, with comparably good results for ω_4_ (96.3%-ile). However, XB1K yields abysmal results for [*r*_e_(H–C), *r*_e_(C–N), *r*_e_(N–O), Δ*r*_e_(H–C), ω_1_] with the percentiles (0.0, 3.8, 1.3, 13.4, 0.2).

**Table 6:** Percentiles Showing How the Best DFAs (out of 473) in One Category Perform Otherwise within a Collection of 13 Properties of HCNO^a^

		%-ile												
Functional	Group	Δ*E*_e_(rxn)	*r*_e_(H–C)	*r*_e_(C–N)	*r*_e_(N–O)	Δ*r*_e_(H–C)	Δ*r*_e_(C–N)	ω_1_	ω_2_	ω_3_	ω_4_	ω_5_	Δω(C–H)	Δω(C–N)
MPWB1K	3	best	35.6	10.8	3.3	99.6	89.1	27.7	5.2	3.8	6.3	48.8	72.9	55.7
B3LYP-D3(BJ)2B	3	64.9	best	73.8	75.4	60.1	50.1	98.8	64.0	73.3	60.2	94.8	79.3	29.6
hPBEint	3	13.6	21.0	best	33.3	16.5	49.1	54.6	45.0	20.0	43.1	82.1	35.1	66.8
TPSS-NL	2	26.7	25.0	32.3	best	23.8	15.2	25.0	81.3	90.4	99.6	33.1	10.4	18.6
VSXC	2	51.1	59.0	44.2	60.8	best	21.9	61.9	96.5	62.9	72.9	30.2	34.0	45.7
ωB97	1	87.9	36.7	51.0	69.0	67.8	best	71.3	17.7	30.0	19.8	62.1	83.5	51.4
TPSSh-D3(BJ)2B	3	63.9	82.3	94.8	82.3	19.6	52.8	best	72.5	76.0	66.5	98.1	34.2	65.6
TH4	1	29.4	78.8	55.4	61.0	71.0	22.3	39.0	best	71.5	97.3	23.8	30.5	50.5
oPBE-D	1	16.7	6.3	12.1	83.1	48.4	10.9	7.3	65.8	best	82.1	30.4	20.0	31.1
revTPSS-NL	2	31.3	15.8	23.1	99.2	6.9	3.3	19.6	77.1	88.8	best	37.9	2.5	12.7
XB1K	3	36.1	0.0	3.8	1.3	13.4	93.1	0.2	38.8	43.8	96.3	best	55.9	17.1
B2GP-PLYP-NL	6	82.3	48.3	98.5	63.1	86.6	58.9	44.4	81.9	58.1	55.8	92.1	best	77.9
B97-2-D3(0)2B	3	45.1	95.2	69.2	28.8	20.0	76.4	55.6	35.8	26.7	38.5	78.1	38.2	best

a best ≥ 99.6%-ile.

The top performers for [*r*_e_(H–C), *r*_e_(C–N), *r*_e_(N–O)] are [(B3LYP-D3(BJ)2B, group 3), (hPBEint, group 3), (TPSS-NL, group 2)]. Of these three DFAs, B3LYP-D3(BJ)2B is the only case where all three *r*_e_ predictions are above 70%-ile, as hPBEint and TPSS-NL yield 21.0–33.3%-iles for the other *r*_e_ values. The mean percentile of the other properties for B3LYP-D3(BJ)2B is 68.7, second to only that of B2GP-PLYP-NL with a value of 70.7. Despite giving the best *r*_e_(N–O), TPSS-NL incorrectly predicts HCNO to have a bent minimum. Likewise, the bent minimum given by VSXC (group 2) actually shows the best Δ*r*_e_(H–C) %-ile but inconsistently displays (59.0, 21.9)%-ile for [*r*_e_(H–C), Δ*r*_e_(C–N)]. The group 1 ωB97 functional yields the highest Δ*r*_e_(C–N) %-ile, and those for [*r*_e_(C–N), Δ*r*_e_(H–C)] are (51.0, 67.8).

TPSSh-D3(BJ)2B (group 3) produces the best ω_1_, and its mean percentile for the other properties is 67.4. TH4 and oPBE-D (group 1) both give erroneous bent HCNO minima but have the best performance for ω_2_ and ω_3_, respectively. The best ω_4_ is given by revTPSS-NL (group 2, linear minimum). Although acceptable ω_2_-ω_4_ results are found for (TH4, oPBE-D, revTPSS-NL), these three DFAs give %-iles for (ω_1_, ω_5_) = [(39.0, 23.8), (7.3, 30.4), (19.6, 37.9)]. B2GP-PLYP-NL (group 6) predicts Δω(H–C) with the most accuracy, albeit with a 44.4%-ile result for ω_1_. In fact, B2GP-PLYP-NL is the only DFA in Table 6 where the lowest %-ile across all properties is above 44. Similarly, B97-2-D3(0)2B yields the highest Δω(C–N) %-ile but performs with 26.7%-ile for ω_3_. The essential observation from Table 6 is that the top DFAs in a particular category are relatively poor performers in various other areas, and no clear overall champion emerges.

### 3.9 Dispersion Corrections

As a long-range electron correlation effect, intramolecular dispersion should have little influence on the bond distances, bond angles, vibrational frequencies, and bond energies of a small molecule such as HCNO that exhibits strong covalent bonding. A stated objective in developing DFAs is that “the dispersion correction does not interfere with covalent bond lengths.”^102^ Nonetheless, a question rarely discussed is whether dispersion corrections designed to rectify DFA deficiencies for long-range interactions are indeed properly damped at short-range to prevent undesirable shifts in essential molecular properties distinctive of chemical bonds. We researched this question by analyzing changes in the predictions given by myriad parent DFAs upon application of 11 semiclassical dispersion corrections: D, D3, D3(BJ), D3(BJ)2B, D3(BJ)ATM, D3(0)2B, D3(0)ATM, D3M(BJ)2B, D3M(BJ)ATM, D3M(0)2B, and D3M(0)ATM. Specific information regarding these dispersion schemes can be found in refs (81), (102−109). Comprehensive statistical tables detailing the effects of each dispersion correction on HCNO bond distances, bond angles, harmonic vibrational frequencies, and dissociation energies are presented in Tables S7–S11. For the sake of brevity, we only discuss highlights of the data here.

Generally, dispersion corrections do noticeably impact the covalently bonded properties of HCNO but do not provide a consensus as to which direction the shift occurs. Across all bond distances and all functionals, the mean absolute shift (MAS) was only 0.0002 Å with a standard deviation (σ_D_) of 0.0004 Å. However, examples of much more sizable bond distance shifts are encountered, such as Δ*r*_e_(C–N) = −0.0027 Å for PBE-D3M(0)ATM and Δ*r*_e_(N–O) = 0.0045 Å for ωB97X-D3(BJ). For the 125 data with a Becke-Johnson (BJ) damping scheme conjoined with two-body (2B) or three-body (ATM) terms, including the modified (M) variations, each scheme yields [*r*_e_(H–C), *r*_e_(C–N), *r*_e_(N–O)] contractions for at least (93, 60, 93)% of the parent DFAs. In stark contrast, mostly positive shifts are observed for the -D3(BJ) correction without consideration of many-body effects. An entirely different story is told by the 124 instances in which a “zero-damping” (0) scheme was employed. Negative shifts in bond distances are observed for less than 5% of parent DFAs with D3(0)2B and D3(0)ATM, whereas D3M(0)2B and D3M(0)ATM give contractions in 38% of cases. Among the four corrections implemented with the most parent DFAs [D3(BJ)2B, D3(BJ)ATM, D3(0)2B, and D3(0)ATM], the MAS did not exceed 0.00017 Å, with the two (0) schemes showing larger shifts than the (BJ) varieties. Standard deviations in bond-distance shifts were noticeably greater for the D3M schemes, the largest being (0.0008, 0.0011, 0.0009) Å for [*r*_e_(H–C), *r*_e_(C–N), *r*_e_(N–O)].

Dispersion corrections with (BJ) damping distort both θ_e_(H–C–N) and θ_e_(C–N–O) away from linearity in all cases, whereas the (0) damping schemes show a strong propensity (>87%) in the opposite direction. For all methods, the MAS values for θ_e_(H–C–N) and θ_e_(C–N–O) lie within the [0.26°, 1.47°] and [0.06°, 0.34°] intervals, respectively. While θ_e_(C–N–O) shifts less than 1.2°, the changes in θ_e_(H–C–N) exceed 1.5° in four instances. In a striking example, TPSSh-D3(BJ)2B produces a θ_e_(H–C–N) decrease of nearly 5°. The parent functional produces an unambiguous linear minimum, but inclusion of the D3(BJ)2B correction curiously produces both bent and linear minima differing in energy by merely 0.22 cm^–1^. The bent structure has [θ_e_(H–C–N), θ_e_(C–N–O)] = (175.1, 178.9)°, thus producing bond angle shifts of (−4.9, 1.1)° from the reference linear structure. The largest positive bond-angle shift [Δθ_e_(H–C–N) = +1.3°] belongs to PBE-D3M(0)2B, analogous to the sizable bond-distance shifts for PBE-D3M(0)ATM.

Over the set of all harmonic frequencies and all dispersion corrections to parent DFAs, the MAS and σ_D_ are 1.7 and 4.1 cm^–1^, respectively. In accord with Badger’s Rule,^110^ dispersion corrections that give bond distance contractions yield increases in ω_1_-ω_3_ values and *vice versa*. Among the four most widely implemented corrections (*vide supra*) and their modified (M) counterparts, shifts with (BJ) damping have consistently smaller means, MASs, and standard deviations than those with (0) damping; however, the (BJ) varieties exhibit broader ranges than their (0) counterparts. Notably, the minimum and maximum observed shifts both involved ω_5_(H–C–N bend). With ωB97X-D, ω_5_ decreased by a striking 57 cm^–1^, whereas the DSD-PBEB95-D3(BJ) increased ω_5_ by 62 cm^–1^. Presumably, these large shifts are a direct consequence of the extremely flat H–C–N bending potential of fulminic acid.

The dispersion corrections investigated here most substantially impact the HCN + O(^3^*P*) → HCNO reaction energy [Δ*E*_e_(rxn) = −*D*_e_(HCN–O)]. Over the entire data set, the mean shift in the reaction energy is −0.43 kcal mol^–1^ with an attendant standard deviation of 0.84 kcal mol^– 1^, but these statistics are misleading. As expected, the dispersion corrections decrease Δ*E*_e_(rxn) and hence increase *D*_e_(HCN–O) with the exception of D3(BJ), which yields shifts of the opposite sign in all cases. The (BJ) damping schemes more strongly influence the dissociation energy than their (0) counterparts, and the (M) variations influence results even greater still. Six dispersion schemes shift Δ*E*_e_(rxn) in the negative direction by more than 3.9 kcal mol^–1^, as highlighted by the conspicuous –6.3 kcal mol^–1^ change in the case of HF-D3M(BJ)ATM. On the other hand, DSD-PBEB95-D3(BJ) shifts Δ*E*_e_(rxn) in the positive direction by 2.6 kcal mol^–1^. Our recent benchmark research^74^ with definitive wave function methods gives a Δ*E*_e_(rxn) = −51.08 kcal mol^– 1^. Because Δ*E*_e_(rxn) = (14.4, −57.4) kcal mol^–1^ for (HF, DSD-PBEB95), the [D3M(BJ)ATM, D3(BJ)] corrections do move the reaction energy in a favorable direction, but the magnitude of the change is too large to be a consequence of intramolecular dispersion.

## 4 Summary and Perspectives

Our analysis on the performance of 473 DFAs on fulminic acid demonstrates that the top-performing functionals for one property typically do not repeat strong performances for other properties. Moreover, over the entire set the rung of a functional on the DFT ladder is not well correlated with its performance ranking. Overall winners for the HCNO challenge are not evident by inspection, so we have resorted to averaging individual performance rankings over the 473 DFAs for 15 separate properties: Δ*E*_e_(rxn), *r*_e_(H–C), *r*_e_(C–N), *r*_e_(N–O), θ_e_(H–C–N), θ_e_(C–N–O), Δ*r*_e_(H–C), Δ*r*_e_(C–N), ω_1_-ω_5_, Δω(H–C str.), and Δω(C–N str.). For θ_e_(H–C–N) and θ_e_(C–N–O), functionals received a first-place ranking if they produced a linear minimum; DFAs that gave a bent minimum were then ranked from 277 to 473 according to how far the angles were removed from 180°. By our overall metric, no DFA achieves an average ranking better than 111 out of 473, and the top four performers all belong to the B2GP-PLYP family: B2GP-PLYP-NL, B2GP-PLYP-D3(0)ATM, B2GP-PLYP, B2GP-PLYP-D3(BJ)ATM. In fact, of the top 30 DFAs, 23 employ the Lee-Yang-Parr correlation functional, the exceptions being TPSSh and PWPB95 with its six associated dispersion and nonlocal correlation variants. Of the top 40 DFAs, 70% belong to group 3 with the remaining 30% belonging to group 6. These findings are contrary to many of the recommendations of DFT benchmarking studies.^81^ A complete ranking of all DFAs from top to bottom is included in Table S12.

No convergence is reached for the equilibrium structure of the vexing fulminic acid molecule as the DFT ladder is climbed. Qualitatively, DFAs in groups 1 and 2 overwhelmingly produce incorrect bent minima. This deficiency is incrementally rectified by groups 3, 4, and 5 until no bent minima remain, but the DFT ladder ultimately relapses at group 6 to a majority of bent minima. Analysis of Δ*E*_e_(rxn) reveals that the DFT ladder is quite successful in achieving reasonably precise results, albeit with a systematic median error of almost 5 kcal mol^–1^ at the top of the ladder (group 6). Although the group 6 DFAs do yield the best values for ω_5_(H–C–N bend), the size of this frequency is exaggerated throughout the DFT ladder, and thus the critical feature of the quasilinear/quasibent conundrum is misrepresented.

Contrary to expectations, group 1 is statistically the best performer for both *r*_e_(N–O) and ω_3_, but the results are too scattered for applications that demand high accuracy, such as spectroscopy and chemical bonding theory. The DFT ladder is largely successful for Δ*r*_e_(H–C), with large reductions in spread and generally more accurate medians with group improvements. Similarly, group 6 produces the best results for Δω(C–N str.), but an unexpectedly broad distribution for this property within group 4 renders the success of the DFT ladder dubious. We also observe that long-range dispersion corrections noticeably impact the covalently bonded properties of fulminic acid and substantially shift Δ*E*_e_(rxn), in some instances by more than 4 kcal mol^–1^.

Now 30 years from the investigation of Handy et al.,^76^ DFT methods are still incapable of firmly determining whether fulminic acid has a bent or linear equilibrium structure. The modern DFT hierarchy is too erratic to truly ascertain “the most delicate properties of small molecules,” as the esteemed Cambridge group initially had hoped.^76^ While the DFT *ladder* might thus be considered a collective failure for the fulminic acid challenge, various functionals within the panoply still deliver HCNO results with accuracy acceptable for many applications. Therefore, our research should not be considered a false alarm against the use of DFT in general. Moreover, the scatter plots introduced here should not be construed as a misguided scheme to improve or extrapolate results based on performance statistics. These plots are merely intended to concisely display voluminous facts, including the disappointing outliers. To employ such plots in an algorithm to reach final answers, the collection of DFAs at each rung would need to be pruned to a modest set of best-in-class performers, although reaching a consensus on which functionals should be included as best-in-class would likely be difficult.

To gain some final perspective on the status of DFT performance for fulminic acid, it is worthwhile to take a step backward and consider def2-QZVP results from specific high-rung functionals such as ωB97M-V (group 5, Table 1) and the relatively new variant ωB97M(2) (group 6).^111^ The latter DFA is not available in Psi4 but is implemented in Q-Chem 6.2. A linear HCNO equilibrium structure is given by ωB97M-V, and the attendant errors with respect to AE-CCSDTQ(P)/CBS + MVD1 for [Δ*E*_e_(rxn), *r*_e_(H–C), *r*_e_(C–N), *r*_e_(N–O), ω_1_, ω_2_, ω_3_, ω_4_, ω_5_] are (−0.7 kcal mol^–1^, 0.0005 Å, −0.0091 Å, −0.0034 Å, 8 cm^–1^, 71 cm^–1^, 31 cm^–1^, 24 cm^–1^, 328 cm^–1^). Likewise, ωB97M(2) gives a linear minimum, and the same list of errors is (−2.1 kcal mol^–1^, −0.0029 Å, −0.0028 Å, −0.0012 Å, 36 cm^–1^, 31 cm^–1^, 16 cm^–1^, 17 cm^–1^, 219 cm^–1^). With the exception of the “delicate” ω_5_ frequency, many users would be satisfied with the performance of either ωB97M-V or ωB97M(2), but high-resolution spectroscopists and theorists committed to rigorous electronic wave function methods are among those who generally would not be so inclined. Some DFT supporters might argue that the ω_5_ disparity is immaterial because this quantity only measures quadratic force constants on a highly anharmonic potential energy surface. This objection is dispelled by a mathematical analysis we have presented in Section 7 of the Supporting Information.

For the 5000 data points in the MGDCB84 set, ωB97M(2) does provide clear statistical improvements in accuracy over ωB97M-V.^111^ However, this status does not guarantee that ωB97M(2) is the better functional for fulminic acid, and indeed its performance is noticeably worse for Δ*E*_e_(rxn), *r*_e_(H–C), and ω_1_. In the absence of even higher DFT rungs to ascend, a scientist seeking precise results on fulminic acid would thus be left in a quandary. In brief, the best current DFAs have not yet fully reached “chemical accuracy” and for that reason resolving the H–C–N bending potential of HCNO may logically lie beyond their present resolving power. In contrast, by pushing the level of theory to AE-CCSDTQ(P)/CBS + MVD1, electronic wave function methods yield unambiguously converged results for HCNO.^74^ Of course, such methods are more than an order of magnitude more costly than all the DFAs tested here, as getting the right answer for the right reason does not come cheap and may be intractable for larger systems. The late Professor Handy might still hope for the day that DFT could provide a far less expensive route to the same final answers.

## Data Availability

The data supporting this article have been included as a part of the Supporting Information.

## Supporting Information Available

A list of all DFAs employed with corresponding references (Table S1); tabulation of all results for fulminic acid properties (Tables S2–S6); additional histogram and scatter plots for HCNO properties not discussed in the text (Figures S1–S9); statistical tables detailing dispersion correction side effects (Tables S7–S11); a final ranking of all DFAs based on overall HCNO performance (Table S12); statistical comparisons of properties computed with Psi4 vs Q-Chem (Section 6, Table S13); and a mathematical analysis of the importance of the quadratic bending force constant on the potential curves for H–C–N bending (Section 7, Figures S10–S11) (PDF)
