# Supplementary material for: Tests of the DFT Ladder for the Fulminic Acid Challenge
Source: J Am Chem Soc. 2025 Apr 16;147(17):14088–104. doi: 10.1021/jacs.4c13823 (PMC12046562; doi:10.1021/jacs.4c13823)
Supplement: Supplementary file 1 — ja4c13823_si_001.pdf [file ja4c13823_si_001.pdf]

Supplementary Information
Tests of the DFT Ladder for the Fulminic Acid Challenge

Ashley M. Allen, Laura N. Olive Dornshuld, Patricia A. Gonzalez Franco, Wesley D. Allen, and Henry F. Schaefer III*

Center for Computational Quantum Chemistry, University of Georgia, Athens, GA 30602 USA

E-mail: ccq@uga.edu

Table of Contents

1. DFAs employed with corresponding Psi4 keywords, types, and literature sources (Table S1) p. S2
2. Tabulation of all DFA results (Tables S2-S6) p. S14
3. Histogram and scatter plots for other HCNO properties (Figures S1-S9) p. S69
4. Statistics for dispersion correction effects (Tables S7-S11) p. S78
5. Comprehensive ranking of all DFAs (Table S12) p. S83
6. Property comparison: Q-Chem vs. Psi4 p. S89
7. Influence of the quadratic bending force constant on the potential curves along the minimum-energy path for H–C–N bending in fulminic acid p. S93
8. Complete DFA literature sources (References) p. S95

Table S1. DFT functionals and types with corresponding Psi4 keywords and literature sources

XC = exchange correlation	Hyb = hybrid	+D = plus dispersion	m = meta
SCS = spin component scaled	dHyb = double hybrid	(K) = for kinetics	(S) = screened
SOS = scaled opposite spin	NS = nonseparable	rev = revised	SR = short ranged

Functional	Psi4 Keyword	Type	Reference
B1LYP	B1LYP	Hyb-GGA XC	1
B1LYP-D3(BJ)2B	B1LYP-D3BJ2B	Hyb-GGA XC +D	1–3
B1LYP-D3(BJ)ATM	B1LYP-D3BJATM	Hyb-GGA XC +D	1,2,4,5
B1LYP-D3(0)2B	B1LYP-D3ZERO2B	Hyb-GGA XC +D	1,3,6
B1LYP-D3(0)ATM	B1LYP-D3ZEROATM	Hyb-GGA XC +D	1,4–6
B1PW91	B1PW91	Hyb-GGA XC	1
B1WC	B1WC	Hyb-GGA XC	7
B2GP-PLYP	B2GPPLYP	dHyb-GGA XC	8
B2GP-PLYP-D3(BJ)2B*	B2GPPLYP-D3BJ2B	dHyb-GGA XC +D	2,8,9
B2GP-PLYP-D3(BJ)ATM	B2GPPLYP-D3BJATM	dHyb-GGA XC +D	2,4,5,8
B2GP-PLYP-D3(0)2B	B2GPPLYP-D3ZERO2B	dHyb-GGA XC +D	6,8,10
B2GP-PLYP-D3(0)ATM	B2GPPLYP-D3ZEROATM	dHyb-GGA XC +D	4–6,8
B2GP-PLYP-NL	B2GPPLYP-NL	dHyb-GGA XC	8,11,12
B2PLYP*	B2PLYP	dHyb-GGA XC	13
B2PLYP-D3(BJ)2B	B2PLYP-D3BJ2B	dHyb-GGA XC +D	2,9,13
B2PLYP-D3(BJ)ATM	B2PLYP-D3BJATM	dHyb-GGA XC +D	2,4,5,13
B2PLYP-D3M(BJ)2B	B2PLYP-D3MBJ2B	dHyb-GGA XC +D	2,13,14
B2PLYP-D3M(BJ)ATM	B2PLYP-D3MBJATM	dHyb-GGA XC +D	2,4,5,13,14
B2PLYP-D3M(0)2B	B2PLYP-D3MZERO2B	dHyb-GGA XC +D	6,13,14
B2PLYP-D3M(0)ATM	B2PLYP-D3MZEROATM	dHyb-GGA XC +D	4–6,13,14
B2PLYP-D3(0)2B	B2PLYP-D3ZERO2B	dHyb-GGA XC +D	6,10,13
B2PLYP-D3(0)ATM	B2PLYP-D3ZEROATM	dHyb-GGA XC +D	4–6,13
B2PLYP-NL	B2PLYP-NL	dHyb-GGA XC	11,13,15
B3LYP*	B3LYP	Hyb-GGA XC	16,17
B3LYP-D3(BJ)2B	B3LYP-D3BJ2B	Hyb-GGA XC +D	2,16,17
B3LYP-D3(BJ)ATM	B3LYP-D3BJATM	Hyb-GGA XC +D	2,4,5,16,17
B3LYP-D3M(BJ)2B	B3LYP-D3MBJ2B	Hyb-GGA XC +D	2,14,16,17
B3LYP-D3M(BJ)ATM	B3LYP-D3MBJATM	Hyb-GGA XC +D	2,4,5,14,16,17
B3LYP-D3M(0)2B	B3LYP-D3MZERO2B	Hyb-GGA XC +D	6,14,16,17
B3LYP-D3M(0)ATM	B3LYP-D3MZEROATM	Hyb-GGA XC +D	4–6,14,16,17
B3LYP-D3(0)2B	B3LYP-D3ZERO2B	Hyb-GGA XC +D	6,16,17
B3LYP-D3(0)ATM	B3LYP-D3ZEROATM	Hyb-GGA XC +D	4–6,16,17
B3LYP-NL	B3LYP-NL	Hyb-GGA XC	11,16,17
B3LYP5	B3LYP5	Hyb-GGA XC	16,18,19
B3LYPS	B3LYPS	Hyb-GGA XC	16,17,20
B3P86	B3P86	Hyb-GGA XC	17,21
B3P86-D3(BJ)2B	B3P86-D3BJ2B	Hyb-GGA XC +D	2,3,17,21
B3P86-D3(BJ)ATM	B3P86-D3BJATM	Hyb-GGA XC +D	2–5,17,21
B3P86-D3(0)2B	B3P86-D3ZERO2B	Hyb-GGA XC +D	3,6,17,21
B3P86-D3(0)ATM	B3P86-D3ZEROATM	Hyb-GGA XC +D	3–6,17,21
B3PW91	B3PW91	Hyb-GGA XC	17,22

Table S1 (continued)

Functional	Psi4 Keyword	Type	Reference
B3PW91-D3(BJ)2B	B3PW91-D3BJ2B	Hyb-GGA XC +D	2,9,17,22
B3PW91-D3(BJ)ATM	B3PW91-D3BJATM	Hyb-GGA XC +D	2,4,5,17,22
B3PW91-D3(0)2B	B3PW91-D3ZERO2B	Hyb-GGA XC +D	6,9,17,22
B3PW91-D3(0)ATM	B3PW91-D3ZEROATM	Hyb-GGA XC +D	4–6,17,22
B3PW91-NL	B3PW91-NL	Hyb-GGA XC	11,17,22
B5050LYP	B5050LYP	Hyb-GGA XC	23
B86B95	B86B95	Hyb-GGA XC	24
B86bPBE	B86BPBE	GGA XC	25
B88B95	B88B95	Hyb-GGA XC	24
B88B95-D3(BJ)2B	B88B95-D3BJ2B	Hyb-GGA XC +D	2,9,24
B88B95-D3(BJ)ATM	B88B95-D3BJATM	Hyb-GGA XC +D	2,4,5,9,24
B88B95-D3(0)2B	B88B95-D3ZERO2B	Hyb-GGA XC +D	6,9,24
B88B95-D3(0)ATM	B88B95-D3ZEROATM	Hyb-GGA XC +D	4–6,9,24
B97-0	B97-0	Hyb-GGA XC	26
B97-1	B97-1	Hyb-GGA XC	27
B97-1-D3(BJ)2B	B97-1-D3BJ2B	Hyb-GGA XC +D	2,3,27
B97-1-D3(BJ)ATM	B97-1-D3BJATM	Hyb-GGA XC +D	2–5,27
B97-1-D3(0)2B	B97-1-D3ZERO2B	Hyb-GGA XC +D	3,6,27
B97-1-D3(0)ATM	B97-1-D3ZEROATM	Hyb-GGA XC +D	3–6,27
B97-1P	B97-1P	Hyb-GGA XC	28
B97-2	B97-2	Hyb-GGA XC	29
B97-2-D3(BJ)2B	B97-2-D3BJ2B	Hyb-GGA XC +D	2,3,29
B97-2-D3(BJ)ATM	B97-2-D3BJATM	Hyb-GGA XC +D	2–5,29
B97-2-D3(0)2B	B97-2-D3ZERO2B	Hyb-GGA XC +D	3,6,29
B97-2-D3(0)ATM	B97-2-D3ZEROATM	Hyb-GGA XC +D	3–6,29
B97-3	B97-3	Hyb-GGA XC	30
B97-D*	B97-D	GGA XC	31
B97-D3(BJ)*	B97-D3BJ	GGA XC +D	2,31
B97-D3M(BJ)	B97-D3MBJ	GGA XC +D	2,14,31
$B97^{GGA}$-1	B97-GGA1	GGA XC	28
B97-K	B97-K	Hyb-GGA XC	32
B97M-D3(BJ)	B97M-D3BJ	GGA XC +D	2,33,34
B97M-V	B97M-V	GGA XC +D	34
BB1K	BB1K	Hyb-GGA XC	35
BHandH	BHANDH	Hyb-GGA XC	36
BHandHLYP	BHANDHLYP	Hyb-GGA XC	36
BLYP	BLYP	GGA XC	16,18
BLYP-D3(BJ)2B	BLYP-D3BJ2B	GGA XC +D	2,16,18
BLYP-D3(BJ)ATM	BLYP-D3BJATM	GGA XC +D	2,4,5,16,18
BLYP-D3M(BJ)2B	BLYP-D3MBJ2B	GGA XC +D	2,14,16,18
BLYP-D3M(BJ)ATM	BLYP-D3MBJATM	GGA XC +D	2,4,5,14,16,18

Table S1 (continued)

Functional	Psi4 Keyword	Type	Reference
BLYP-D3M(0)2B	BLYP-D3MZERO2B	GGA XC +D	6,14,16,18
BLYP-D3M(0)ATM	BLYP-D3MZEROATM	GGA XC +D	4–6,14,16,18
BLYP-D3(0)2B	BLYP-D3ZERO2B	GGA XC +D	6,16,18
BLYP-D3(0)ATM	BLYP-D3ZEROATM	GGA XC +D	4–6,16,18
BLYP-NL	BLYP-NL	GGA XC	11,16,18
BMK	BMK	Hyb-m-GGA XC (K)	32
BMK-D3(BJ)2B	BMK-D3BJ2B	Hyb-m-GGA XC (K) +D	2,9,32
BMK-D3(BJ)ATM	BMK-D3BJATM	Hyb-m-GGA XC (K) +D	2,4,5,32
BMK-D3(0)2B	BMK-D3ZERO2B	Hyb-m-GGA XC (K) +D	6,9,32
BMK-D3(0)ATM	BMK-D3ZEROATM	Hyb-m-GGA XC (K) +D	4–6,32
BOP	BOP	GGA XC	37
BOP-D3(BJ)2B	BOP-D3BJ2B	GGA XC +D	2,37
BOP-D3(BJ)ATM	BOP-D3BJATM	GGA XC +D	2,4,5,37
BOP-D3(0)2B	BOP-D3ZERO2B	GGA XC +D	6,37
BOP-D3(0)ATM	BOP-D3ZEROATM	GGA XC +D	4–6,37
BP86	BP86	GGA XC	18,21
BP86-D3(BJ)2B	BP86-D3BJ2B	GGA XC +D	2,18,21
BP86-D3(BJ)ATM	BP86-D3BJATM	GGA XC +D	2,4,5,18,21
BP86-D3M(BJ)2B	BP86-D3MBJ2B	GGA XC +D	2,14,18,21
BP86-D3M(BJ)ATM	BP86-D3MBJATM	GGA XC +D	2,4,5,14,18,21
BP86-D3M(0)2B	BP86-D3MZERO2B	GGA XC +D	6,14,18,21
BP86-D3M(0)ATM	BP86-D3MZEROATM	GGA XC +D	4–6,14,18,21
BP86-D3(0)2B	BP86-D3ZERO2B	GGA XC +D	6,18,21
BP86-D3(0)ATM	BP86-D3ZEROATM	GGA XC +D	4–6,18,21
BP86-NL	BP86-NL	GGA XC	11,12,18,21
BP86-VWN	BP86-VWN	GGA XC	18,19,21
CAM-B3LYP*	CAM-B3LYP	Hyb-GGA XC	38
CAM-B3LYP-D3(BJ)2B	CAM-B3LYP-D3BJ2B	Hyb-GGA XC +D	2,38
CAM-B3LYP-D3(BJ)ATM	CAM-B3LYP-D3BJATM	Hyb-GGA XC +D	2,4,5,38
CAM-B3LYP-D3(0)2B	CAM-B3LYP-D3ZERO2B	Hyb-GGA XC +D	6,38
CAM-B3LYP-D3(0)ATM	CAM-B3LYP-D3ZEROATM	Hyb-GGA XC +D	4–6,38
CAM-LDA0	CAM-LDA0	XC	39
CAP0	CAP0	Hyb-GGA XC	40
core-DSD-BLYP	CORE-DSD-BLYP	SCS dHyb XC	41,42
core-DSD-BLYP-D3(BJ)2B	CORE-DSD-BLYP-D3BJ2B	SCS dHyb XC +D	2,41,42
core-DSD-BLYP-D3(BJ)ATM	CORE-DSD-BLYP-D3BJATM	SCS dHyb XC +D	2,4,5,41,42
DLDF	DLDF	Hyb-m-GGA XC	43
DLDF+D09	DLDF+D09	Hyb-m-GGA XC	43
DLDF+D10	DLDF+D10	Hyb-m-GGA XC	43,44
DSD-BLYP	DSD-BLYP	SCS dHyb XC	41
DSD-BLYP-D3(BJ)	DSD-BLYP-D3BJ	SCS dHyb XC +D	2,41

Table S1 (continued)

Functional	Psi4 Keyword	Type	Reference
DSD-BLYP-D3(BJ)2B*	DSD-BLYP-D3BJ2B	SCS dHyb XC +D	2,9,41
DSD-BLYP-D3(BJ)ATM	DSD-BLYP-D3BJATM	SCS dHyb XC +D	2,4,5,9,41
DSD-BLYP-D3(0)2B	DSD-BLYP-D3ZERO2B	SCS dHyb XC +D	6,9,41
DSD-BLYP-D3(0)ATM	DSD-BLYP-D3ZEROATM	SCS dHyb XC +D	4–6,9,41
DSD-BLYP-NL	DSD-BLYP-NL	SCS dHyb XC	11,41,42
DSD-PBEB95	DSD-PBEB95	SCS dHyb-m-GGA XC	45
DSD-PBEB95-D3(BJ)	DSD-PBEB95-D3BJ	SCS dHyb-m-GGA XC +D	2,45
DSD-PBEB95-NL	DSD-PBEB95-NL	SCS dHyb-m-GGA XC	11,12,45
DSD-PBEP86*	DSD-PBEP86	SCS dHyb XC	45,46
DSD-PBEP86-D3(BJ)*	DSD-PBEP86-D3BJ	SCS dHyb XC +D	2,45,46
DSD-PBEP86-NL	DSD-PBEP86-NL	SCS dHyb XC	11,12,45,46
DSD-PBEPBE	DSD-PBEPBE	SCS dHyb XC	45
DSD-PBEPBE-D3(BJ)	DSD-PBEPBE-D3BJ	SCS dHyb XC +D	2,45
DSD-PBEPBE-NL	DSD-PBEPBE-NL	SCS dHyb XC	11,12,45
EDF1	EDF1	GGA XC	47
EDF2	EDF2	Hyb-GGA XC	48
FT97	FT97	GGA XC	49
GAM	GAM	GGA XC	50
HCTH/120	HCTH120	GGA XC	51
HCTH/120-D3(BJ)2B	HCTH120-D3BJ2B	GGA XC +D	2,51
HCTH/120-D3(BJ)ATM	HCTH120-D3BJATM	GGA XC +D	2,4,5,51
HCTH/120-D3(0)2B	HCTH120-D3ZERO2B	GGA XC +D	6,51
HCTH/120-D3(0)ATM	HCTH120-D3ZEROATM	GGA XC +D	4–6,51
HCTH/147	HCTH147	GGA XC	51
HCTH/407	HCTH407	GGA XC	52
HCTH/407-D3(BJ)2B	HCTH407-D3BJ2B	GGA XC +D	2,3,52
HCTH/407-D3(BJ)ATM	HCTH407-D3BJATM	GGA XC +D	2,4,5,52
HCTH/407-D3(0)2B	HCTH407-D3ZERO2B	GGA XC +D	3,6,52
HCTH/407-D3(0)ATM	HCTH407-D3ZEROATM	GGA XC +D	4–6,52
HCTH/407+	HCTH407P	GGA XC	53
HCTH/93	HCTH93	GGA XC	54
HCTH-p(1/4)	HCTHP14	GGA XC	55
HCTH-p(7/6)	HCTHP76	GGA XC	55
HF	HF	Hartree–Fock	56
HF+D	HF+D	Hartree–Fock +D	44,56
HF-D3(BJ)2B	HF-D3BJ2B	Hartree–Fock +D	2,56
HF-D3(BJ)ATM	HF-D3BJATM	Hartree–Fock +D	2,4,5,56
HF-D3M(BJ)2B	HF-D3MBJ2B	Hartree–Fock +D	2,14,56
HF-D3M(BJ)ATM	HF-D3MBJATM	Hartree–Fock +D	2,4,5,14,56
HF-D3M(0)2B	HF-D3MZERO2B	Hartree–Fock +D	6,14,56
HF-D3M(0)ATM	HF-D3MZEROATM	Hartree–Fock +D	4–6,14,56

Table S1 (continued)

Functional	Psi4 Keyword	Type	Reference
HF-D3(0)2B	HF-D3ZERO2B	Hartree–Fock +D	6,56
HF-D3(0)ATM	HF-D3ZEROATM	Hartree–Fock +D	4–6,56
HF3C	HF3C	Hartree–Fock (3C composite method)	2
HJS-B97x	HJS-B97X	Hyb-GGA XC	57
HJS-PBE	HJS-PBE	Hyb-GGA XC	57
HJS-PBEsol	HJS-PBE-SOL	Hyb-GGA XC	57,58
hPBEint	HPBEINT	Hyb-GGA XC	59
HSE03	HSE03	Hyb-GGA XC	60
HSE03-D3(BJ)2B	HSE03-D3BJ2B	Hyb-GGA XC +D	2,3,60
HSE03-D3(BJ)ATM	HSE03-D3BJATM	Hyb-GGA XC +D	2,4,5,60
HSE03-D3(0)2B	HSE03-D3ZERO2B	Hyb-GGA XC +D	3,6,60
HSE03-D3(0)ATM	HSE03-D3ZEROATM	Hyb-GGA XC +D	4–6,60
HSE06*	HSE06	Hyb-GGA XC	60,61
HSE06-D3(BJ)2B	HSE06-D3BJ2B	Hyb-GGA XC +D	2,60–62
HSE06-D3(BJ)ATM	HSE06-D3BJATM	Hyb-GGA XC +D	2,4,5,60,61
HSE06-D3(0)2B	HSE06-D3ZERO2B	Hyb-GGA XC +D	6,60–62
HSE06-D3(0)ATM	HSE06-D3ZEROATM	Hyb-GGA XC +D	4–6,60,61
KMLYP	KMLYP	Hyb-GGA XC	63
KSDT	KSDT	XC	64
KT2	KT2	GGA XC	65
LC-BOP	LC-BOP	GGA XC	66–68
LC-VV10	LC-VV10	GGA XC	69
LDA0	LDA0	XC	70
LRC-ωPBE	LRC-WPBE	GGA XC	71
LRC-ωPBEh	LRC-WPBEH	Hyb-GGA XC	72
M05	M05	m-GGA XC	73
M05-2X	M05-2X	Hyb-m-GGA XC	74
M05-2X-D3(0)2B*	M05-2X-D3ZERO2B	Hyb-m-GGA XC +D	6,9,74
M05-2X-D3(0)ATM	M05-2X-D3ZEROATM	Hyb-m-GGA XC +D	4–6,9,74
M05-D3(0)2B	M05-D3ZERO2B	m-GGA XC +D	6,9,73
M05-D3(0)ATM	M05-D3ZEROATM	m-GGA XC +D	4–6,73
M06*	M06	m-GGA XC	75
M06-2X*	M06-2X	Hyb-m-GGA XC	75
M06-2X-D3(0)2B	M06-2X-D3ZERO2B	Hyb-m-GGA XC +D	6,9,75
M06-2X-D3(0)ATM	M06-2X-D3ZEROATM	Hyb-m-GGA XC +D	4–6,9,75
M06-D3(0)2B	M06-D3ZERO2B	m-GGA XC +D	6,9,75
M06-D3(0)ATM	M06-D3ZEROATM	m-GGA XC +D	4–6,75
M06-HF	M06-HF	Hyb XC	76
M06-HF-D3(0)2B	M06-HF-D3ZERO2B	Hyb XC +D	6,76
M06-HF-D3(0)ATM	M06-HF-D3ZEROATM	Hyb XC +D	4–6,76

Table S1 (continued)

Functional	Psi4 Keyword	Type	Reference
M06-L*	M06-L	m-GGA XC	77
M06-L-D3(0)2B	M06-L-D3ZERO2B	m-GGA XC +D	6,9,77
M06-L-D3(0)ATM	M06-L-D3ZEROATM	m-GGA XC +D	4–6,9,77
M08-HX	M08-HX	Hyb XC	78
M08-HX-D3(0)2B	M08-HX-D3ZERO2B	Hyb XC +D	3,6,78
M08-HX-D3(0)ATM	M08-HX-D3ZEROATM	Hyb XC +D	3–6,78
M08-SO	M08-SO	Hyb XC	78
M11	M11	m-GGA XC	79
M11-D3(BJ)2B	M11-D3BJ2B	m-GGA XC +D	2,79,80
M11-D3(BJ)ATM	M11-D3BJATM	m-GGA XC +D	2,4,5,79
M11-D3(0)2B	M11-D3ZERO2B	m-GGA XC +D	6,79,80
M11-D3(0)ATM	M11-D3ZEROATM	m-GGA XC +D	4–6,79,80
M11-L	M11-L	m-GGA XC	81
M11-L-D3(BJ)2B	M11-L-D3BJ2B	m-GGA XC +D	2,80,81
M11-L-D3(BJ)ATM	M11-L-D3BJATM	m-GGA XC +D	2,4,5,80,81
M11-L-D3(0)2B	M11-L-D3ZERO2B	m-GGA XC +D	6,80,81
M11-L-D3(0)ATM	M11-L-D3ZEROATM	m-GGA XC +D	4–6,80,81
mB3LYP-RC04	MB3LYP-RC04	Hyb-GGA XC	82
MGGA_MS0	MGGA_MS0	m-GGA XC	83
MGGA_MS1	MGGA_MS1	m-GGA XC	84
MGGA_MS2	MGGA_MS2	m-GGA XC	84
MGGA_MS2h	MGGA_MS2H	Hyb-m-GGA XC	84
MGGA-MVS	MGGA_MVS	m-GGA XC	85
MGGA-MVSh	MGGA_MVSH	Hyb-m-GGA XC	85
MN12-L	MN12-L	m-GGA XC	86
MN12-L-D3(BJ)2B	MN12-L-D3BJ2B	m-GGA XC +D	2,80,86
MN12-L-D3(BJ)ATM	MN12-L-D3BJATM	m-GGA XC +D	2,4,5,80,86
MN12-L-D3(0)2B	MN12-L-D3ZERO2B	m-GGA XC +D	6,80,86
MN12-L-D3(0)ATM	MN12-L-D3ZEROATM	m-GGA XC +D	4–6,80,86
MN12-SX	MN12-SX	Hyb-m-GGA (S) XC	87
MN12-SX-D3(BJ)2B	MN12-SX-D3BJ2B	Hyb-m-GGA (S) XC +D	2,80,87
MN12-SX-D3(BJ)ATM	MN12-SX-D3BJATM	Hyb-m-GGA (S) XC +D	2,4,5,80,87
MN12-SX-D3(0)2B	MN12-SX-D3ZERO2B	Hyb-m-GGA (S) XC +D	6,80,87
MN12-SX-D3(0)ATM	MN12-SX-D3ZEROATM	Hyb-m-GGA (S) XC +D	4–6,80,87
MN15	MN15	Hyb-m-GGA XC	88
MN15-D3(BJ)2B	MN15-D3BJ2B	Hyb-m-GGA XC +D	2,3,88
MN15-D3(BJ)ATM	MN15-D3BJATM	Hyb-m-GGA XC +D	2,4,5,88
MN15-L	MN15-L	m-GGA XC	89
MN15-L-D3(0)2B	MN15-L-D3ZERO2B	m-GGA XC +D	3,6,89
MN15-L-D3(0)ATM	MN15-L-D3ZEROATM	m-GGA XC +D	3–6,89
MOHLYP	MOHLYP	GGA XC	90

Table S1 (continued)

Functional	Psi4 Keyword	Type	Reference
MOHLYP2	MOHLYP2	GGA XC	91
MP2D	MP2D	MP2 +D	92
MP2	MP2	MP2	93
MPW1B95	MPW1B95	Hyb-GGA XC	94
MPW1B95-D3(BJ)2B	MPW1B95-D3BJ2B	Hyb-GGA XC +D	2,9,94
MPW1B95-D3(BJ)ATM	MPW1B95-D3BJATM	Hyb-GGA XC +D	2,4,5,94
MPW1B95-D3(0)2B	MPW1B95-D3ZERO2B	Hyb-GGA XC +D	6,9,94
MPW1B95-D3(0)ATM	MPW1B95-D3ZEROATM	Hyb-GGA XC +D	4–6,9,94
MPW1K	MPW1K	Hyb-GGA XC	95
mPW1LYP	MPW1LYP	Hyb-GGA XC	16,96
mPW1LYP-D3(0)2B	MPW1LYP-D3ZERO2B	Hyb-GGA XC +D	3,6,16,96
mPW1LYP-D3(0)ATM	MPW1LYP-D3ZEROATM	Hyb-GGA XC +D	4–6,16,96
mPW1PBE	MPW1PBE	Hyb-GGA XC	96,97
mPW1PW	MPW1PW	Hyb-GGA XC	96
mPW1PW-D3(BJ)2B	MPW1PW-D3BJ2B	Hyb-GGA XC +D	2,3,96
mPW1PW-D3(BJ)ATM	MPW1PW-D3BJATM	Hyb-GGA XC +D	2,4,5,96
mPW1PW-D3(0)2B	MPW1PW-D3ZERO2B	Hyb-GGA XC +D	3,6,96
mPW1PW-D3(0)ATM	MPW1PW-D3ZEROATM	Hyb-GGA XC +D	4–6,96
MPW3LYP	MPW3LYP	Hyb-GGA XC	94
mPW3PW	MPW3PW	Hyb-GGA XC	96
MPWB1K	MPWB1K	Hyb-GGA XC	94
MPWB1K-D3(BJ)2B	MPWB1K-D3BJ2B	Hyb-GGA XC +D	2,9,94
MPWB1K-D3(BJ)ATM	MPWB1K-D3BJATM	Hyb-GGA XC +D	2,4,5,94
MPWB1K-D3(0)2B	MPWB1K-D3ZERO2B	Hyb-GGA XC +D	6,9,94
MPWB1K-D3(0)ATM	MPWB1K-D3ZEROATM	Hyb-GGA XC +D	4–6,94
MPWLYP1M	MPWLYP1M	Hyb-GGA XC	90
MPWLYP1W	MPWLYP1W	GGA XC	98
mPWPW	MPWPW	GGA XC	96
N12	N12	NS GGA XC	99
N12-D3(BJ)2B	N12-D3BJ2B	NS GGA XC +D	2,80,99
N12-D3(BJ)ATM	N12-D3BJATM	NS GGA XC +D	2,4,5,80,99
N12-D3(0)2B	N12-D3ZERO2B	NS GGA XC +D	6,80,99
N12-D3(0)ATM	N12-D3ZEROATM	NS GGA XC +D	4–6,99
N12-SX	N12-SX	Hyb NS GGA XC	87
N12-SX-D3(BJ)2B	N12-SX-D3BJ2B	Hyb NS GGA XC +D	2,80,87
N12-SX-D3(BJ)ATM	N12-SX-D3BJATM	Hyb NS GGA XC +D	2,4,5,80,87
N12-SX-D3(0)2B	N12-SX-D3ZERO2B	Hyb NS GGA XC +D	6,80,87
N12-SX-D3(0)ATM	N12-SX-D3ZEROATM	Hyb NS GGA XC +D	4–6,80,87
O3LYP	O3LYP	Hyb-GGA XC	100,101
O3LYP-D3(BJ)2B	O3LYP-D3BJ2B	Hyb-GGA XC +D	2,3,100,101
O3LYP-D3(BJ)ATM	O3LYP-D3BJATM	Hyb-GGA XC +D	2,4,5,100,101

Table S1 (continued)

Functional	Psi4 Keyword	Type	Reference
O3LYP-D3(0)2B	O3LYP-D3ZERO2B	Hyb-GGA XC +D	3,6,100,101
O3LYP-D3(0)ATM	O3LYP-D3ZEROATM	Hyb-GGA XC +D	4–6,100,101
oBLYP-D	OBLYP-D	GGA XC	31,102
oPBE-D	OPBE-D	GGA XC	31,102
oPWLYP-D	OPWLYP-D	GGA XC	31,102
oTPSS-D	OTPSS-D	GGA XC	31,102
PBE*	PBE	GGA XC	97
PBE-D3(BJ)2B	PBE-D3BJ2B	GGA XC +D	2,97
PBE-D3(BJ)ATM	PBE-D3BJATM	GGA XC +D	2,4,5,97
PBE-D3M(BJ)2B	PBE-D3MBJ2B	GGA XC +D	2,14,97
PBE-D3M(BJ)ATM	PBE-D3MBJATM	GGA XC +D	2,4,5,14,97
PBE-D3M(0)2B	PBE-D3MZERO2B	GGA XC +D	6,14,97
PBE-D3M(0)ATM	PBE-D3MZEROATM	GGA XC +D	4–6,14,97
PBE-D3(0)2B	PBE-D3ZERO2B	GGA XC +D	6,97
PBE-D3(0)ATM	PBE-D3ZEROATM	GGA XC +D	4–6,97
PBE-NL	PBE-NL	GGA XC	11,12,97
PBEOP	OP-PBE	GGA XC	103,104
PBEsol	PBE-SOL	GGA XC	105
PBEsol-D3(BJ)2B	PBE-SOL-D3BJ2B	GGA XC +D	2,105
PBEsol-D3(BJ)ATM	PBE-SOL-D3BJATM	GGA XC +D	2,4,5,105
PBEsol-D3(0)2B	PBE-SOL-D3ZERO2B	GGA XC +D	6,105
PBEsol-D3(0)ATM	PBE-SOL-D3ZEROATM	GGA XC +D	4–6,105
PBE0*	PBE0	Hyb-GGA XC	106,107
PBE0-1/3	PBE0-13	Hyb-GGA XC	108
PBE0-2	PBE0-2	dHyb-GGA XC	109
PBE0-D3(BJ)2B	PBE0-D3BJ2B	Hyb-GGA XC +D	2,106,107
PBE0-D3(BJ)ATM	PBE0-D3BJATM	Hyb-GGA XC +D	2,4,5,106,107
PBE0-D3M(BJ)2B	PBE0-D3MBJ2B	Hyb-GGA XC +D	2,14,106,107
PBE0-D3M(BJ)ATM	PBE0-D3MBJATM	Hyb-GGA XC +D	2,4,5,14,106,107
PBE0-D3M(0)2B	PBE0-D3MZERO2B	Hyb-GGA XC +D	6,14,106,107
PBE0-D3M(0)ATM	PBE0-D3MZEROATM	Hyb-GGA XC +D	4–6,14,106,107
PBE0-D3(0)2B	PBE0-D3ZERO2B	Hyb-GGA XC +D	6,106,107
PBE0-D3(0)ATM	PBE0-D3ZEROATM	Hyb-GGA XC +D	4–6,106,107
PBE0-DH	PBE0-DH	dHyb XC	110
PBE0-DH-D3(BJ)2B	PBE0-DH-D3BJ2B	dHyb XC +D	2,110,111
PBE0-DH-D3(BJ)ATM	PBE0-DH-D3BJATM	dHyb XC +D	2,4,5,110,111
PBE0-DH-D3(0)2B	PBE0-DH-D3ZERO2B	dHyb XC +D	6,110,111
PBE0-DH-D3(0)ATM	PBE0-DH-D3ZEROATM	dHyb XC +D	4–6,110,111
PBE0-NL	PBE0-NL	Hyb-GGA XC	11,12,106,107
PBE1W	PBE1W	GGA XC	98
PBE50	PBE50	Hyb-GGA XC	97

Table S1 (continued)

Functional	Psi4 Keyword	Type	Reference
PBEh-3c	PBEH3C	PBE Hyb (3C composite method)	112
PBELYP1W	PBELYP1W	GGA XC	98
PKZB	PKZB	m-GGA XC	113
PKZB-D3(0)2B	PKZB-D3ZERO2B	m-GGA XC +D	3,6,113
PKZB-D3(0)ATM	PKZB-D3ZEROATM	m-GGA XC +D	4–6,113
PTPSS	PTPSS	SOS dHyb XC	114
PTPSS-D3(BJ)2B	PTPSS-D3BJ2B	SOS dHyb XC +D	2,114
PTPSS-D3(BJ)ATM	PTPSS-D3BJATM	SOS dHyb XC +D	2,4,5,114
PTPSS-D3(0)2B	PTPSS-D3ZERO2B	SOS dHyb XC +D	6,114
PTPSS-D3(0)ATM	PTPSS-D3ZEROATM	SOS dHyb XC +D	4–6,114
PW6B95	PW6B95	Hyb-m-GGA XC	115
PW6B95-D3(BJ)2B	PW6B95-D3BJ2B	Hyb-m-GGA XC +D	2,115
PW6B95-D3(BJ)ATM	PW6B95-D3BJATM	Hyb-m-GGA XC +D	2,4,5,115
PW6B95-D3(0)2B	PW6B95-D3ZERO2B	Hyb-m-GGA XC +D	6,115
PW6B95-D3(0)ATM	PW6B95-D3ZEROATM	Hyb-m-GGA XC +D	4–6,115
PW86B95	PW86B95	Hyb-GGA XC	24
PW86PBE	PW86PBE	GGA XC	97,116
PW91*	PW91	GGA XC	117
PW91-D3(BJ)2B	PW91-D3BJ2B	GGA XC +D	2,117,118
PW91-D3(BJ)ATM	PW91-D3BJATM	GGA XC +D	2,4,5,117,118
PWB6K	PWB6K	Hyb-GGA XC	115
PWB6K-D3(BJ)2B	PWB6K-D3BJ2B	Hyb-GGA XC +D	2,115
PWB6K-D3(BJ)ATM	PWB6K-D3BJATM	Hyb-GGA XC +D	2,4,5,115
PWB6K-D3(0)2B	PWB6K-D3ZERO2B	Hyb-GGA XC +D	6,115
PWB6K-D3(0)ATM	PWB6K-D3ZEROATM	Hyb-GGA XC +D	4–6,115
PWPB95	PWPB95	SOS dHyb XC	114
PWPB95-D3(BJ)2B	PWPB95-D3BJ2B	SOS dHyb XC +D	2,114
PWPB95-D3(BJ)ATM	PWPB95-D3BJATM	SOS dHyb XC +D	2,4,5,114
PWPB95-D3(0)2B	PWPB95-D3ZERO2B	SOS dHyb XC +D	6,114
PWPB95-D3(0)ATM	PWPB95-D3ZEROATM	SOS dHyb XC +D	4–6,114
PWPB95-NL	PWPB95-NL	SOS dHyb XC	11,114,119
revB3LYP	REVB3LYP	Hyb-GGA XC	120
revM06-L	REVM06-L	m-GGA XC	121
revPBE*	REVPBE	GGA XC	122
revPBE-D3(BJ)2B*	REVPBE-D3BJ2B	GGA XC +D	2,122
revPBE-D3(BJ)ATM	REVPBE-D3BJATM	GGA XC +D	2,4,5,122
revPBE-D3(0)2B	REVPBE-D3ZERO2B	GGA XC +D	6,122
revPBE-D3(0)ATM	REVPBE-D3ZEROATM	GGA XC +D	4–6,122
revPBE-NL	REVPBE-NL	GGA XC	11,122
revPBE0	REVPBE0	Hyb-GGA XC	106,122
revPBE0-D3(BJ)2B	REVPBE0-D3BJ2B	Hyb-GGA XC +D	2,106,122

Table S1 (continued)

Functional	Psi4 Keyword	Type	Reference
revPBE0-D3(BJ)ATM	REVPBE0-D3BJATM	Hyb-GGA XC +D	2,4,5,106,122
revPBE0-D3(0)2B	REVPBE0-D3ZERO2B	Hyb-GGA XC +D	6,106,122
revPBE0-D3(0)ATM	REVPBE0-D3ZEROATM	Hyb-GGA XC +D	4–6,106,122
revPBE0-NL	REVPBE0-NL	Hyb-GGA XC	11,106,122
revSCAN	REVSCAN	m-GGA XC	123
revSCAN0	REVSCAN0	Hyb-m-GGA XC	123
revTPSS	REVTPSS	m-GGA XC	124
revTPSS-D3(BJ)2B*	REVTPSS-D3BJ2B	m-GGA XC +D	2,3,124
revTPSS-D3(BJ)ATM	REVTPSS-D3BJATM	m-GGA XC +D	2,4,5,124
revTPSS-D3(0)2B	REVTPSS-D3ZERO2B	m-GGA XC +D	3,6,124
revTPSS-D3(0)ATM	REVTPSS-D3ZEROATM	m-GGA XC +D	4–6,124
revTPSS-NL	REVTPSS-NL	m-GGA XC	11,124,125
revTPSSh	REVTPSSH	Hyb-GGA XC	126
revTPSSh-D3(BJ)2B	REVTPSSH-D3BJ2B	Hyb-GGA XC +D	2,3,126
revTPSSh-D3(BJ)ATM	REVTPSSH-D3BJATM	Hyb-GGA XC +D	2,4,5,126
revTPSSh-D3(0)2B	REVTPSSH-D3ZERO2B	Hyb-GGA XC +D	3,6,126
revTPSSh-D3(0)ATM	REVTPSSH-D3ZEROATM	Hyb-GGA XC +D	4–6,126
RPBE	RPBE	GGA XC	127
RPBE-D3(BJ)2B	RPBE-D3BJ2B	GGA XC +D	2,3,127
RPBE-D3(BJ)ATM	RPBE-D3BJATM	GGA XC +D	2,4,5,127
RPBE-D3(0)2B	RPBE-D3ZERO2B	GGA XC +D	3,6,127
RPBE-D3(0)ATM	RPBE-D3ZEROATM	GGA XC +D	4–6,127
SB98-1a	SB98-1A	Hyb-GGA XC	128
SB98-1b	SB98-1B	Hyb-GGA XC	128
SB98-1c	SB98-1C	Hyb-GGA XC	128
SB98-2a	SB98-2A	Hyb-GGA XC	128
SB98-2b	SB98-2B	Hyb-GGA XC	128
SB98-2c	SB98-2C	Hyb-GGA XC	128
SCAN*	SCAN	m-GGA XC	129
SCAN-D3(BJ)2B*	SCAN-D3BJ2B	m-GGA XC +D	2,129,130
SCAN-D3(BJ)ATM	SCAN-D3BJATM	m-GGA XC +D	2,4,5,129
SCAN-D3(0)2B	SCAN-D3ZERO2B	m-GGA XC +D	6,129,130
SCAN-D3(0)ATM	SCAN-D3ZEROATM	m-GGA XC +D	4–6,129
SCAN0	SCAN0	Hyb-m-GGA XC	131
SOGGA	SOGGA	SOGGA X + PBE C	132
SOGGA11	SOGGA11	XC	133
SOGGA11-X	SOGGA11-X	Hyb XC	134
SOGGA11-X-D3(BJ)2B	SOGGA11-X-D3BJ2B	Hyb XC +D	2,80,134
SOGGA11-X-D3(BJ)ATM	SOGGA11-X-D3BJATM	Hyb XC +D	2,4,5,80,134
SOGGA11-X-D3(0)2B	SOGGA11-X-D3ZERO2B	Hyb XC +D	6,80,134
SOGGA11-X-D3(0)ATM	SOGGA11-X-D3ZEROATM	Hyb XC +D	4–6,80,134

Table S1 (continued)

Functional	Psi4 Keyword	Type	Reference
SPW92	SPW92	Slater Exchange	22,135,136
SVWN	SVWN	Slater Exchange	19,135,136
τ-HCTH	T-HCTH	m-GGA XC	137
τ-HCTH-D3(BJ)2B	T-HCTH-D3BJ2B	m-GGA XC +D	2,3,137
τ-HCTH-D3(BJ)ATM	T-HCTH-D3BJATM	m-GGA XC +D	2–5,137
τ-HCTH-D3(0)2B	T-HCTH-D3ZERO2B	m-GGA XC +D	3,6,137
τ-HCTH-D3(0)ATM	T-HCTH-D3ZEROATM	m-GGA XC +D	3–6,137
τ-HCTHh	T-HCTHH	Hyb-m-GGA XC	137
Teter93	TETER93	XC	138
TH-FC	TH-FC	GGA XC	139
TH-FC+FO	TH-FCFO	GGA XC	139
TH-FCO	TH-FCO	GGA XC	139
TH-FL	TH-FL	GGA XC	139
TH1	TH1	GGA XC	140
TH2	TH2	GGA XC	141
TH3	TH3	GGA XC	142
TH4	TH4	GGA XC	142
TPSS	TPSS	m-GGA XC	143
TPSS-D3(BJ)2B	TPSS-D3BJ2B	m-GGA XC +D	2,143
TPSS-D3(BJ)ATM	TPSS-D3BJATM	m-GGA XC +D	2,4,5,143
TPSS-D3(0)2B	TPSS-D3ZERO2B	m-GGA XC +D	6,143
TPSS-D3(0)ATM	TPSS-D3ZEROATM	m-GGA XC +D	4–6,143
TPSS-NL	TPSS-NL	m-GGA XC	11,143,144
TPSSh*	TPSSH	Hyb-GGA XC	145
TPSSh-D3(BJ)2B	TPSSH-D3BJ2B	Hyb-GGA XC +D	2,9,145
TPSSh-D3(BJ)ATM	TPSSH-D3BJATM	Hyb-GGA XC +D	2,4,5,145
TPSSh-D3(0)2B	TPSSH-D3ZERO2B	Hyb-GGA XC +D	6,9,145
TPSSh-D3(0)ATM	TPSSH-D3ZEROATM	Hyb-GGA XC +D	4–6,145
TPSSLYP1W	TPSSLYP1W	GGA XC	98
tuned-CAM-B3LYP	TUNED-CAM-B3LYP	Hyb-GGA XC	146
VSXC	VSXC	m-GGA XC	147
VV10	VV10	GGA XC	69
ωB97	WB97	GGA XC	148
ωB97M-D3(BJ)	WB97M-D3BJ	Hyb-GGA XC +D	2,149,150
ωB97M-V	WB97M-V	Hyb-GGA XC +D	150
ωB97X	WB97X	Hyb-GGA XC	148
ωB97X-D*	WB97X-D	Hyb-GGA XC	151
ωB97X-D3*	WB97X-D3	Hyb-GGA XC	6,151,152
ωB97X-D3(BJ)	WB97X-D3BJ	Hyb-GGA XC +D	2,149,153
ωB97X-D3(0)2B	WB97X-D3ZERO2B	Hyb-GGA XC +D	6,148,152
ωB97X-D3(0)ATM	WB97X-D3ZEROATM	Hyb-GGA XC +D	4–6,148

Table S1 (continued)

Functional	Psi4 Keyword	Type	Reference
ωB97X-V*	WB97X-V	Hyb-GGA XC +D	153
ωPBE	WPBE	SR-XC	57,154
ωPBE-D3(BJ)2B	WPBE-D3BJ2B	SR-XC +D	2,57,154
ωPBE-D3(BJ)ATM	WPBE-D3BJATM	SR-XC +D	2,4,5,57,154
ωPBE-D3M(BJ)2B	WPBE-D3MBJ2B	SR-XC +D	2,14,57,154
ωPBE-D3M(BJ)ATM	WPBE-D3MBJATM	SR-XC +D	2,4,5,14,57,154
ωPBE-D3M(0)2B	WPBE-D3MZERO2B	SR-XC +D	6,14,57,154
ωPBE-D3M(0)ATM	WPBE-D3MZEROATM	SR-XC +D	4–6,14,57,154
ωPBE-D3(0)2B	WPBE-D3ZERO2B	SR-XC +D	6,57,154
ωPBE-D3(0)ATM	WPBE-D3ZEROATM	SR-XC +D	4–6,57,154
ωPBE0	WPBE0	SR-XC	57,154
X1B95	X1B95	Hyb-GGA XC	94
X3LYP	X3LYP	Hyb-GGA XC	155
X3LYP-D3(BJ)2B	X3LYP-D3BJ2B	Hyb-GGA XC +D	2,3,155
X3LYP-D3(BJ)ATM	X3LYP-D3BJATM	Hyb-GGA XC +D	2,4,5,155
X3LYP-D3(0)2B	X3LYP-D3ZERO2B	Hyb-GGA XC +D	3,6,155
X3LYP-D3(0)ATM	X3LYP-D3ZEROATM	Hyb-GGA XC +D	4–6,155
XB1K	XB1K	Hyb-GGA XC	94
XLYP	XLYP	GGA XC	155
XLYP-D3(BJ)2B	XLYP-D3BJ2B	GGA XC +D	2,3,155
XLYP-D3(BJ)ATM	XLYP-D3BJATM	GGA XC +D	2,4,5,155
XLYP-D3(0)2B	XLYP-D3ZERO2B	GGA XC +D	3,6,155
XLYP-D3(0)ATM	XLYP-D3ZEROATM	GGA XC +D	4–6,155
ZLP	ZLP	GGA XC	156

* denotes functionals used for preliminary testing at three different integration grid settings

Table S2. DFT bent minima for HCNO: electronic energies (E_h), geometric structures (Å, °), and harmonic vibrational frequencies (cm^{-1})

Functional	Group	Energy	r_e(H–C)	r_e(C–N)	r_e(N–O)	θ_e(H–C–N)	θ_e(C–N–O)	$\omega_1(a')$	$\omega_2(a')$	$\omega_3(a')$	$\omega_4(a')$	$\omega_5(a')$	$\omega_6(a'')$
B2PLYP	6	−168.573258	1.0573	1.1615	1.1992	174.0	178.6	3513.8	2280.7	1286.7	557.1	73.9	556.7
B2PLYP−D3(BJ)2B	6	−168.574401	1.0573	1.1616	1.1991	173.7	178.6	3513.7	2280.8	1287.1	557.3	81.2	559.4
B2PLYP−D3(BJ)ATM	6	−168.574401	1.0573	1.1616	1.1991	173.6	178.6	3513.9	2280.8	1287.0	557.2	85.6	557.8
B2PLYP−D3M(BJ)2B	6	−168.575062	1.0573	1.1616	1.1990	173.3	178.5	3513.8	2280.8	1287.3	557.2	86.7	558.5
B2PLYP−D3M(BJ)ATM	6	−168.575062	1.0573	1.1616	1.1990	173.3	178.5	3514.2	2281.0	1287.2	557.5	88.0	557.2
B2PLYP−D3M(0)2B	6	−168.573784	1.0575	1.1616	1.1994	174.8	178.8	3513.4	2280.1	1285.9	557.6	67.3	557.4
B2PLYP−D3M(0)ATM	6	−168.573784	1.0575	1.1616	1.1994	174.8	178.8	3513.0	2279.8	1285.9	557.4	69.0	557.7
B2PLYP−D3(0)2B	6	−168.573510	1.0574	1.1616	1.1993	174.6	178.8	3513.4	2280.1	1285.8	557.6	72.1	558.4
B2PLYP−D3(0)ATM	6	−168.573510	1.0574	1.1616	1.1993	174.5	178.8	3512.6	2280.1	1285.8	558.1	75.0	557.4
B2PLYP−NL	6	−168.510354	1.0576	1.1617	1.1990	173.3	178.5	3511.7	2280.8	1287.6	556.7	87.7	557.0
B86bPBE	1	−168.634152	1.0678	1.1725	1.2020	160.1	175.5	3407.0	2250.5	1278.9	544.0	255.4	544.4
B97−D	1	−168.561081	1.0653	1.1693	1.1973	160.6	175.5	3416.7	2254.3	1281.5	546.4	252.3	546.7
B97−D3(BJ)	1	−168.565227	1.0646	1.1694	1.1967	159.0	175.2	3423.5	2256.1	1285.2	546.1	273.4	546.5
B97−D3M(BJ)	1	−168.569728	1.0644	1.1692	1.1962	158.6	175.1	3425.4	2258.7	1287.8	545.6	277.6	546.7
$B97^{GGA}$−1	1	−168.607577	1.0633	1.1673	1.1885	159.5	175.3	3454.7	2297.5	1319.2	558.5	265.2	559.2
B97M−D3(BJ)	1	−168.718034	1.0543	1.1564	1.1903	161.0	175.3	3544.2	2338.2	1322.1	571.8	237.8	571.7
B97M−V	1	−168.623549	1.0548	1.1566	1.1903	161.0	175.3	3540.3	2337.6	1322.3	571.8	238.0	572.0
BLYP	1	−168.643628	1.0663	1.1727	1.2102	160.1	175.5	3393.5	2213.3	1241.7	531.9	259.9	531.9
BLYP−D3(BJ)2B	1	−168.646833	1.0663	1.1728	1.2100	159.6	175.4	3393.2	2213.2	1242.6	531.6	266.7	531.7
BLYP−D3(BJ)ATM	1	−168.646833	1.0663	1.1728	1.2100	159.6	175.4	3393.2	2213.2	1242.6	531.6	266.7	531.7
BLYP−D3M(BJ)2B	1	−168.648401	1.0662	1.1728	1.2098	159.4	175.3	3393.8	2213.7	1243.3	531.5	269.1	531.6
BLYP−D3M(BJ)ATM	1	−168.648401	1.0662	1.1728	1.2098	159.4	175.3	3393.8	2213.7	1243.3	531.5	269.1	531.6
BLYP−D3M(0)2B	1	−168.644830	1.0667	1.1731	1.2107	160.2	175.5	3389.4	2209.8	1239.4	532.2	260.9	532.3
BLYP−D3M(0)ATM	1	−168.644830	1.0667	1.1731	1.2107	160.2	175.5	3389.4	2209.8	1239.5	532.2	260.9	532.3
BLYP−D3(0)2B	1	−168.644161	1.0665	1.1729	1.2104	160.1	175.5	3390.5	2211.2	1239.9	531.9	259.5	532.0
BLYP−D3(0)ATM	1	−168.644161	1.0665	1.1729	1.2104	160.1	175.5	3390.5	2211.2	1239.8	531.9	259.5	532.0
BLYP−NL	1	−168.486765	1.0672	1.1730	1.2093	159.5	175.3	3387.4	2215.2	1245.9	531.6	267.3	531.2
BOP	1	−168.630963	1.0669	1.1745	1.2100	158.1	174.9	3392.0	2208.7	1243.3	532.3	287.5	532.4
BOP−D3(BJ)2B	1	−168.637181	1.0666	1.1745	1.2096	157.5	174.8	3393.1	2209.6	1245.1	531.8	294.4	532.0
BOP−D3(BJ)ATM	1	−168.637181	1.0666	1.1745	1.2096	157.5	174.8	3393.1	2209.6	1245.1	531.8	294.4	532.0
BOP−D3(0)2B	1	−168.632042	1.0672	1.1747	1.2106	158.8	175.1	3387.3	2205.5	1239.5	532.6	278.2	532.8

Table S2 (continued)

Functional	Group	Energy	r_e(H–C)	r_e(C–N)	r_e(N–O)	θ_e(H–C–N)	θ_e(C–N–O)	$\omega_1(a')$	$\omega_2(a')$	$\omega_3(a')$	$\omega_4(a')$	$\omega_5(a')$	$\omega_6(a'')$
BOP–D3(0)ATM	1	−168.632042	1.0672	1.1747	1.2106	158.8	175.1	3387.3	2205.5	1239.5	532.6	278.2	532.8
BP86	1	−168.673363	1.0693	1.1736	1.2032	158.8	175.2	3389.4	2238.0	1272.5	540.7	270.4	540.9
BP86–D3(BJ)2B	1	−168.675594	1.0692	1.1737	1.2031	158.6	175.2	3390.3	2238.6	1272.9	541.3	276.4	541.7
BP86–D3(BJ)ATM	1	−168.675594	1.0692	1.1737	1.2031	158.6	175.2	3390.3	2238.6	1272.9	541.3	276.4	541.7
BP86–D3M(BJ)2B	1	−168.675866	1.0692	1.1737	1.2030	158.5	175.2	3390.5	2238.6	1273.0	541.3	277.8	541.7
BP86–D3M(BJ)ATM	1	−168.675866	1.0692	1.1737	1.2030	158.5	175.2	3390.5	2238.6	1273.0	541.3	277.8	541.7
BP86–D3M(0)2B	1	−168.673892	1.0694	1.1738	1.2034	159.0	175.3	3387.9	2236.7	1270.7	541.5	271.2	541.9
BP86–D3M(0)ATM	1	−168.673892	1.0694	1.1738	1.2034	159.0	175.3	3387.9	2236.7	1270.7	541.5	271.2	541.9
BP86–D3(0)2B	1	−168.673865	1.0694	1.1738	1.2034	158.9	175.3	3387.9	2236.8	1270.8	541.5	271.8	541.9
BP86–D3(0)ATM	1	−168.673865	1.0694	1.1738	1.2034	158.9	175.3	3387.9	2236.8	1270.8	541.5	271.8	541.9
BP86–NL	1	−168.535446	1.0700	1.1739	1.2025	158.4	175.1	3382.5	2239.7	1275.2	540.4	276.0	535.2
BP86–VWN	1	−168.683223	1.0685	1.1728	1.2025	159.2	175.3	3396.5	2243.6	1275.1	542.4	269.1	542.7
core–DSD–BLYP	6	−168.522200	1.0568	1.1625	1.1960	170.5	177.9	3523.9	2282.6	1302.4	562.7	129.3	562.9
core–DSD–BLYP–D3(BJ)2B	6	−168.523161	1.0567	1.1625	1.1960	170.3	177.8	3524.1	2282.9	1302.7	562.1	128.3	564.0
core–DSD–BLYP–D3(BJ)ATM	6	−168.523161	1.0567	1.1625	1.1960	170.3	177.8	3524.0	2282.7	1302.6	562.3	130.1	562.5
DSD–BLYP	6	−168.542338	1.0551	1.1611	1.1930	174.8	178.9	3543.6	2299.2	1316.4	568.5	70.1	568.8
DSD–BLYP–D3(BJ)	6	−168.529974	1.0560	1.1610	1.1952	173.8	178.6	3534.1	2291.9	1307.0	564.8	84.2	568.3
DSD–BLYP–D3(BJ)2B	6	−168.543299	1.0551	1.1611	1.1930	174.7	178.8	3544.0	2299.5	1316.7	568.6	73.3	574.3
DSD–BLYP–D3(BJ)ATM	6	−168.543299	1.0551	1.1611	1.1930	174.6	178.8	3543.8	2299.3	1316.7	568.4	76.3	569.3
DSD–BLYP–D3(0)2B	6	−168.542501	1.0552	1.1612	1.1931	174.9	178.9	3542.6	2298.6	1315.9	568.8	73.6	574.0
DSD–BLYP–D3(0)ATM	6	−168.542501	1.0552	1.1612	1.1931	174.8	178.9	3542.5	2298.4	1315.8	568.7	73.9	574.3
DSD–BLYP–NL	6	−168.493958	1.0562	1.1611	1.1953	173.9	178.6	3532.1	2291.2	1306.6	564.9	84.3	564.9
DSD–PBEP86	6	−168.446728	1.0589	1.1638	1.1935	168.9	177.5	3516.6	2290.2	1314.6	565.6	144.3	554.6
DSD–PBEP86–D3(BJ)	6	−168.431255	1.0599	1.1645	1.1950	167.3	177.2	3507.2	2282.6	1308.3	562.7	171.2	562.3
DSD–PBEP86–NL	6	−168.398755	1.0600	1.1646	1.1951	167.2	177.1	3505.2	2282.0	1308.0	562.5	170.6	560.0
DSD–PBEPBE	6	−168.452896	1.0585	1.1638	1.1921	167.8	177.3	3521.2	2296.2	1320.1	567.4	163.8	568.3
DSD–PBEPBE–D3(BJ)	6	−168.438515	1.0597	1.1646	1.1939	166.1	176.9	3508.1	2286.0	1311.9	562.8	184.1	565.1
DSD–PBEPBE–NL	6	−168.390296	1.0599	1.1647	1.1939	166.0	176.9	3505.8	2285.7	1312.0	562.5	184.9	563.7
EDF1	1	−168.703941	1.0644	1.1698	1.1991	159.8	175.4	3429.6	2264.7	1286.9	546.7	264.2	547.1
FT97	1	−168.586926	1.0711	1.1784	1.2039	156.3	174.5	3384.4	2230.2	1274.0	540.8	300.9	541.7
GAM	1	−168.669906	1.0564	1.1605	1.1811	169.5	177.6	3524.0	2358.3	1352.8	573.3	137.2	573.6

Table S2 (continued)

Functional	Group	Energy	r_e(H–C)	r_e(C–N)	r_e(N–O)	θ_e(H–C–N)	θ_e(C–N–O)	$\omega_1(a')$	$\omega_2(a')$	$\omega_3(a')$	$\omega_4(a')$	$\omega_5(a')$	$\omega_6(a'')$
HCTH/120	1	−168.636866	1.0636	1.1674	1.1923	160.7	175.6	3443.7	2287.9	1306.8	553.7	252.3	554.2
HCTH/120−D3(BJ)2B	1	−168.639514	1.0636	1.1675	1.1922	160.2	175.5	3443.3	2287.6	1307.5	553.5	259.5	554.0
HCTH/120−D3(BJ)ATM	1	−168.639514	1.0636	1.1675	1.1922	160.2	175.5	3443.3	2287.6	1307.5	553.5	259.5	554.0
HCTH/120−D3(0)2B	1	−168.637221	1.0637	1.1675	1.1924	160.7	175.6	3442.3	2286.8	1305.8	553.7	250.8	554.2
HCTH/120−D3(0)ATM	1	−168.637221	1.0637	1.1675	1.1924	160.7	175.6	3442.3	2286.8	1305.8	553.7	250.8	554.2
HCTH/147	1	−168.641754	1.0635	1.1670	1.1918	161.0	175.7	3447.2	2291.9	1309.4	554.9	247.2	555.4
HCTH/407	1	−168.630611	1.0624	1.1650	1.1881	163.5	176.3	3467.9	2313.5	1324.7	560.9	215.1	561.4
HCTH/407−D3(BJ)2B	1	−168.639365	1.0621	1.1648	1.1873	162.6	176.1	3470.1	2317.0	1328.2	559.9	226.3	560.5
HCTH/407−D3(BJ)ATM	1	−168.639365	1.0621	1.1648	1.1873	162.6	176.1	3470.1	2317.0	1328.2	559.9	226.3	560.5
HCTH/407−D3(0)2B	1	−168.631226	1.0626	1.1652	1.1884	164.0	176.4	3464.6	2311.3	1322.1	561.1	208.1	561.6
HCTH/407−D3(0)ATM	1	−168.631226	1.0626	1.1652	1.1884	164.0	176.4	3464.6	2311.3	1322.1	561.1	208.0	561.6
HCTH/407+	1	−168.642130	1.0641	1.1685	1.1890	159.8	175.4	3454.1	2303.4	1322.9	558.6	263.7	559.3
HCTH/93	1	−168.615710	1.0641	1.1680	1.1911	159.4	175.3	3442.4	2291.4	1312.4	555.8	269.1	556.4
HCTH−p(1/4)	1	−168.807434	1.0606	1.1606	1.1882	172.0	178.3	3487.6	2338.7	1331.2	564.3	104.5	564.4
HCTH−p(7/6)	1	−165.729799	1.0897	1.1896	1.1963	150.4	173.0	3251.1	2199.3	1286.3	546.1	368.6	547.6
KT2	1	−171.309978	1.0580	1.1730	1.2000	150.3	172.7	3425.1	2226.7	1272.9	538.4	373.7	538.0
M06−L	2	−168.642898	1.0577	1.1608	1.1898	162.9	175.8	3510.9	2347.5	1339.1	583.3	210.3	584.1
M06−L−D3(0)2B	2	−168.642909	1.0577	1.1608	1.1898	163.0	175.8	3510.8	2347.5	1339.1	583.4	209.8	584.1
M06−L−D3(0)ATM	2	−168.642909	1.0577	1.1608	1.1898	163.0	175.8	3510.8	2347.5	1339.1	583.4	209.8	584.1
M11−L	2	−168.628421	1.0649	1.1538	1.1664	150.6	172.8	3439.5	2352.5	1375.3	580.8	381.3	581.2
M11−L−D3(BJ)2B	2	−168.629067	1.0649	1.1538	1.1663	150.6	172.8	3439.5	2352.5	1375.4	580.8	381.6	581.2
M11−L−D3(BJ)ATM	2	−168.629068	1.0649	1.1538	1.1663	150.6	172.8	3439.5	2352.5	1375.4	580.8	381.6	581.2
M11−L−D3(0)2B	2	−168.628647	1.0650	1.1539	1.1665	150.7	172.8	3438.2	2351.7	1374.4	580.8	380.0	581.2
M11−L−D3(0)ATM	2	−168.628648	1.0650	1.1539	1.1665	150.7	172.8	3438.2	2351.7	1374.4	580.8	380.0	581.2
MGGA_MS0	2	−168.695839	1.0609	1.1640	1.1990	150.6	172.5	3473.6	2258.4	1278.5	541.2	384.0	537.0
MGGA_MS1	2	−168.669606	1.0630	1.1673	1.2000	150.0	172.4	3454.2	2247.3	1277.2	540.5	392.1	536.8
MGGA_MS2	2	−168.696008	1.0635	1.1680	1.1994	149.0	172.1	3445.2	2244.8	1279.6	539.1	407.6	534.9
MGGA_MS2h	4	−168.672422	1.0594	1.1576	1.1971	157.6	174.3	3504.8	2304.1	1293.5	552.5	282.8	550.2
MN12−L	2	−168.503507	1.0567	1.1471	1.1884	175.3	178.8	3543.1	2373.4	1318.8	583.3	69.3	583.2
MN12−L−D3(BJ)2B	2	−168.503727	1.0567	1.1471	1.1884	175.3	178.8	3543.1	2373.4	1318.8	583.3	69.3	583.2
MN12−L−D3(BJ)ATM	2	−168.503727	1.0567	1.1471	1.1884	175.3	178.8	3543.1	2373.4	1318.8	583.3	69.3	583.2

Table S2 (continued)

Functional	Group	Energy	r_e(H–C)	r_e(C–N)	r_e(N–O)	θ_e(H–C–N)	θ_e(C–N–O)	$\omega_1(a')$	$\omega_2(a')$	$\omega_3(a')$	$\omega_4(a')$	$\omega_5(a')$	$\omega_6(a'')$
MN12–L–D3(0)2B	2	−168.503714	1.0568	1.1472	1.1885	175.7	178.9	3541.7	2372.7	1317.9	583.6	68.1	583.2
MN12–L–D3(0)ATM	2	−168.503714	1.0568	1.1472	1.1885	175.7	178.9	3541.9	2372.7	1317.9	583.7	68.7	585.4
MN15–L	2	−168.561444	1.0688	1.1660	1.1986	160.7	175.0	3493.8	2335.6	1322.9	575.6	224.9	576.0
MN15–L–D3(0)2B	2	−168.561444	1.0688	1.1660	1.1986	160.7	175.0	3493.8	2335.6	1322.9	575.6	224.9	576.0
MN15–L–D3(0)ATM	2	−168.561444	1.0688	1.1660	1.1986	160.7	175.0	3493.8	2335.6	1322.9	575.6	224.9	576.0
MOHLYP	1	−167.934934	1.0778	1.1861	1.2092	152.5	173.5	3334.3	2193.5	1257.9	533.5	350.6	534.2
MOHLYP2	1	−168.551661	1.0750	1.1899	1.2108	147.8	171.9	3335.2	2160.8	1243.4	529.3	415.9	529.6
MP2D	0	−168.426157	1.0587	1.1792	1.1879	155.7	174.4	3501.1	2259.7	1328.5	557.9	405.7	572.1
MP2	0	−168.428885	1.0587	1.1791	1.1878	155.6	174.4	3501.5	2260.5	1328.7	558.0	406.8	571.4
MPWLYP1M	3	−168.631535	1.0634	1.1659	1.2080	168.6	177.4	3433.5	2249.4	1252.1	540.2	145.3	540.2
MPWLYP1W	1	−168.727692	1.0655	1.1719	1.2099	160.5	175.6	3399.3	2215.9	1242.1	532.1	255.1	532.2
mPWPW	1	−168.656102	1.0671	1.1716	1.2014	160.2	175.6	3409.8	2252.5	1279.7	544.5	257.2	544.9
N12	1	−168.626890	1.0528	1.1549	1.1865	172.1	178.3	3496.1	2312.7	1305.2	556.8	104.4	556.8
N12–D3(BJ)2B	1	−168.628700	1.0528	1.1549	1.1864	171.8	178.3	3496.3	2312.7	1305.5	556.7	108.0	556.8
N12–D3(BJ)ATM	1	−168.628700	1.0528	1.1549	1.1864	171.8	178.3	3496.3	2312.8	1305.5	556.7	108.0	556.8
N12–D3(0)2B	1	−168.627463	1.0530	1.1550	1.1868	173.1	178.5	3493.9	2311.0	1303.0	557.0	90.6	557.2
N12–D3(0)ATM	1	−168.627463	1.0530	1.1550	1.1868	173.1	178.5	3493.5	2310.8	1303.0	557.0	90.6	557.0
oBLYP–D	1	−168.597086	1.0664	1.1717	1.2089	161.8	175.9	3394.2	2221.2	1246.1	533.9	237.3	534.1
oPBE–D	1	−168.670878	1.0695	1.1756	1.2050	158.2	175.0	3387.7	2229.8	1266.7	539.6	281.7	540.0
PBEOP	1	−168.516422	1.0676	1.1746	1.2098	158.9	175.1	3390.1	2211.9	1245.6	532.8	276.5	533.0
oPWLYP–D	1	−168.598483	1.0666	1.1719	1.2092	161.7	175.8	3392.0	2219.3	1245.1	533.5	239.6	533.6
oTPSS–D	1	−168.593380	1.0676	1.1712	1.1968	153.4	173.4	3401.1	2256.7	1288.2	550.9	384.7	551.6
PBE	1	−168.486994	1.0689	1.1728	1.2010	160.2	175.6	3402.7	2254.8	1284.0	545.4	254.5	545.9
PBE1W	1	−168.682822	1.0673	1.1724	1.2033	160.2	175.5	3407.2	2244.8	1272.9	542.0	256.5	542.4
PBE–D3(BJ)2B	1	−168.488500	1.0688	1.1728	1.2010	160.2	175.6	3403.3	2255.2	1284.2	545.3	254.0	545.8
PBE–D3(BJ)ATM	1	−168.488500	1.0688	1.1728	1.2010	160.2	175.6	3403.3	2255.2	1284.2	545.3	254.0	545.8
PBE–D3M(BJ)2B	1	−168.488959	1.0688	1.1728	1.2009	160.2	175.6	3403.2	2255.4	1284.5	545.3	254.4	545.8
PBE–D3M(BJ)ATM	1	−168.488959	1.0688	1.1728	1.2009	160.2	175.6	3403.3	2255.4	1284.4	545.3	254.0	545.8
PBE–D3M(0)2B	1	−168.492765	1.0671	1.1701	1.2004	161.5	175.9	3410.6	2262.5	1286.2	547.5	238.6	548.0
PBE–D3M(0)ATM	1	−168.492765	1.0671	1.1701	1.2004	161.5	175.9	3410.6	2262.5	1286.2	547.5	238.6	548.0
PBE–D3(0)2B	1	−168.487247	1.0689	1.1729	1.2011	160.2	175.6	3401.9	2254.1	1283.3	545.4	254.3	545.9

Table S2 (continued)

Functional	Group	Energy	r_e(H–C)	r_e(C–N)	r_e(N–O)	θ_e(H–C–N)	θ_e(C–N–O)	$\omega_1(a')$	$\omega_2(a')$	$\omega_3(a')$	$\omega_4(a')$	$\omega_5(a')$	$\omega_6(a'')$
PBE–D3(0)ATM	1	−168.487247	1.0689	1.1729	1.2011	160.2	175.6	3401.9	2254.1	1283.3	545.4	254.3	545.9
PBELYP1W	1	−168.713993	1.0660	1.1726	1.2098	160.2	175.5	3397.9	2216.2	1243.9	532.5	260.9	532.6
PBE–NL	1	−168.404293	1.0693	1.1730	1.2007	160.0	175.5	3399.6	2255.6	1285.3	544.8	257.2	545.1
PBEsol	1	−167.876191	1.0717	1.1705	1.1955	163.9	176.6	3396.7	2284.5	1306.7	553.6	204.7	554.0
PBEsol–D3(BJ)2B	1	−167.876683	1.0717	1.1705	1.1955	163.9	176.5	3396.7	2284.5	1306.8	553.6	205.0	554.0
PBEsol–D3(BJ)ATM	1	−167.876683	1.0717	1.1705	1.1955	163.9	176.5	3396.7	2284.5	1306.8	553.6	205.0	554.0
PBEsol–D3(0)2B	1	−167.876384	1.0718	1.1705	1.1956	164.1	176.6	3396.1	2284.0	1306.2	553.7	203.0	554.0
PBEsol–D3(0)ATM	1	−167.876384	1.0718	1.1705	1.1956	164.1	176.6	3396.1	2284.0	1306.2	553.7	203.0	554.0
PKZB	2	−168.362903	1.0755	1.1852	1.2055	149.6	172.8	3351.6	2203.3	1270.2	539.9	385.0	540.6
PKZB–D3(0)2B	2	−168.367521	1.0744	1.1853	1.2040	146.5	172.1	3340.6	2198.6	1268.0	536.6	410.7	537.3
PKZB–D3(0)ATM	2	−168.367521	1.0744	1.1853	1.2040	146.5	172.1	3340.6	2198.6	1268.0	536.6	410.7	537.3
PTPSS	6	−168.563644	1.0598	1.1649	1.1969	163.4	176.3	3490.2	2271.8	1294.1	557.1	215.1	558.5
PTPSS–D3(BJ)2B	6	−168.564496	1.0598	1.1649	1.1969	163.3	176.3	3491.0	2272.2	1294.5	557.6	216.8	559.0
PTPSS–D3(BJ)ATM	6	−168.564496	1.0598	1.1649	1.1969	163.3	176.3	3490.4	2272.0	1294.4	558.0	216.9	558.7
PTPSS–D3(0)2B	6	−168.563852	1.0599	1.1650	1.1970	163.6	176.3	3489.0	2270.9	1293.3	557.6	212.9	559.1
PTPSS–D3(0)ATM	6	−168.563852	1.0599	1.1650	1.1970	163.6	176.3	3489.0	2271.0	1293.1	556.9	211.1	558.1
PW86PBE	1	−168.772821	1.0667	1.1724	1.2050	161.2	175.8	3411.1	2241.3	1267.2	541.3	244.3	541.7
PW91	1	−168.617980	1.0670	1.1708	1.2008	161.3	175.8	3412.9	2257.7	1282.1	545.4	241.4	545.7
PW91–D3(BJ)2B	1	−168.618752	1.0669	1.1708	1.2007	161.3	175.8	3413.0	2257.7	1282.2	545.3	242.2	545.7
PW91–D3(BJ)ATM	1	−168.618752	1.0669	1.1708	1.2007	161.3	175.8	3413.0	2257.7	1282.2	545.3	242.2	545.7
revM06–L	2	−168.595463	1.0564	1.1490	1.1863	170.5	177.8	3533.5	2395.4	1348.0	592.1	113.9	592.1
revPBE	1	−168.626678	1.0702	1.1773	1.2044	156.3	174.5	3387.9	2229.5	1271.5	540.3	305.7	540.9
revPBE–D3(BJ)2B	1	−168.630792	1.0700	1.1773	1.2042	156.0	174.5	3388.5	2230.0	1272.5	540.0	309.8	540.6
revPBE–D3(BJ)ATM	1	−168.630792	1.0700	1.1773	1.2042	156.0	174.5	3388.5	2230.0	1272.5	540.0	309.8	540.6
revPBE–D3(0)2B	1	−168.627574	1.0705	1.1775	1.2049	156.9	174.7	3384.5	2227.2	1268.9	540.5	298.4	541.1
revPBE–D3(0)ATM	1	−168.627574	1.0705	1.1775	1.2049	156.9	174.7	3384.4	2227.2	1268.9	540.5	298.5	541.1
revPBE–NL	1	−168.452391	1.0711	1.1776	1.2035	155.9	174.5	3381.9	2232.3	1275.7	539.7	308.5	539.5
revSCAN	2	−168.579387	1.0620	1.1620	1.1910	161.4	175.2	3474.4	2324.4	1328.8	570.9	225.5	573.4
revTPSS	2	−168.630895	1.0664	1.1721	1.2030	154.8	174.0	3413.4	2240.3	1274.4	549.2	324.5	549.6
revTPSS–D3(BJ)2B	2	−168.632579	1.0663	1.1722	1.2029	154.7	174.0	3413.4	2240.3	1274.8	549.0	326.5	549.5
revTPSS–D3(BJ)ATM	2	−168.632660	1.0663	1.1722	1.2029	154.7	174.0	3413.4	2240.6	1274.8	549.0	325.7	552.7

Table S2 (continued)

Functional	Group	Energy	r_e(H–C)	r_e(C–N)	r_e(N–O)	θ_e(H–C–N)	θ_e(C–N–O)	$\omega_1(a')$	$\omega_2(a')$	$\omega_3(a')$	$\omega_4(a')$	$\omega_5(a')$	$\omega_6(a'')$
revTPSS–D3(0)2B	2	−168.631255	1.0665	1.1722	1.2032	155.0	174.0	3411.9	2239.2	1273.1	549.2	322.2	549.7
revTPSS–D3(0)ATM	2	−168.631255	1.0665	1.1722	1.2032	155.0	174.0	3411.9	2239.1	1273.1	549.2	322.2	549.7
revTPSSh	3	−168.611903	1.0613	1.1597	1.2001	170.0	177.6	3486.1	2307.3	1291.2	563.6	122.0	563.6
revTPSSh–D3(BJ)2B	3	−168.613463	1.0613	1.1598	1.2000	169.7	177.6	3486.1	2307.3	1291.5	563.5	125.0	563.6
revTPSSh–D3(BJ)ATM	3	−168.613463	1.0613	1.1598	1.2000	169.7	177.6	3486.1	2307.3	1291.5	563.5	125.0	563.6
revTPSSh–D3(0)2B	3	−168.612243	1.0614	1.1598	1.2003	170.4	177.7	3484.8	2306.4	1289.9	563.7	117.0	563.7
revTPSSh–D3(0)ATM	3	−168.612243	1.0614	1.1598	1.2003	170.4	177.7	3484.9	2306.5	1289.9	563.7	115.9	563.7
revTPSS–NL	2	−168.528151	1.0669	1.1724	1.2026	154.6	173.9	3409.7	2241.0	1276.1	548.3	327.3	549.0
RPBE	1	−168.674546	1.0705	1.1786	1.2056	155.4	174.3	3384.0	2221.7	1267.4	538.5	316.9	539.1
RPBE–D3(BJ)2B	1	−168.683557	1.0701	1.1784	1.2048	154.7	174.1	3386.5	2225.0	1271.1	537.6	326.0	538.2
RPBE–D3(BJ)ATM	1	−168.683557	1.0701	1.1784	1.2048	154.7	174.1	3386.5	2225.1	1271.1	537.6	326.0	538.2
RPBE–D3(0)2B	1	−168.675833	1.0709	1.1788	1.2061	156.0	174.4	3382.7	2220.4	1266.1	538.8	310.5	539.4
RPBE–D3(0)ATM	1	−168.675833	1.0709	1.1788	1.2061	156.0	174.4	3382.7	2220.4	1266.1	538.8	310.5	539.4
SCAN	2	−168.589989	1.0628	1.1629	1.1942	157.8	174.3	3451.9	2297.5	1307.5	557.6	288.6	559.8
SCAN–D3(BJ)2B	2	−168.590312	1.0628	1.1629	1.1941	157.8	174.3	3454.9	2293.4	1302.7	556.5	272.4	552.2
SCAN–D3(BJ)ATM	2	−168.590312	1.0628	1.1629	1.1941	157.8	174.3	3451.9	2297.4	1307.6	557.6	288.8	559.8
SCAN–D3(0)2B	2	−168.590048	1.0628	1.1629	1.1942	157.8	174.3	3452.0	2297.4	1307.6	557.6	288.8	559.8
SCAN–D3(0)ATM	2	−168.590048	1.0628	1.1629	1.1942	157.8	174.3	3452.0	2297.5	1307.6	557.6	288.8	559.8
SOGGA	1	−167.745680	1.0729	1.1711	1.1942	163.8	176.5	3394.2	2290.6	1313.6	555.8	205.7	556.3
SOGGA11	1	−168.682530	1.0648	1.1621	1.1873	179.6	179.9	3438.6	2304.2	1318.2	560.7	72.1	560.7
TH1	1	−168.610624	1.0688	1.1749	1.1986	154.9	174.1	3392.6	2232.6	1277.0	544.0	324.9	544.5
TH2	1	−168.603916	1.0690	1.1776	1.2045	153.3	173.6	3377.8	2198.9	1251.8	535.6	347.3	535.8
TH3	1	−168.610635	1.0679	1.1745	1.2029	155.9	174.4	3390.2	2225.5	1265.9	539.4	314.3	539.6
TH4	1	−168.605726	1.0601	1.1660	1.1953	160.1	175.5	3452.1	2274.9	1291.8	549.2	263.7	549.4
τ–HCTH	2	−168.610315	1.0619	1.1639	1.1894	162.8	176.0	3458.3	2299.0	1311.7	556.2	223.4	556.6
τ–HCTH–D3(BJ)2B	2	−168.614736	1.0618	1.1639	1.1892	162.4	176.0	3458.8	2299.9	1313.0	555.9	227.8	556.2
τ–HCTH–D3(BJ)ATM	2	−168.614736	1.0618	1.1639	1.1892	162.4	176.0	3458.8	2299.9	1313.0	555.9	227.8	556.2
τ–HCTH–D3(0)2B	2	−168.611020	1.0622	1.1641	1.1898	163.4	176.2	3455.5	2297.2	1309.5	556.4	215.5	556.8
τ–HCTH–D3(0)ATM	2	−168.611021	1.0622	1.1641	1.1898	163.4	176.2	3455.5	2297.2	1309.5	556.4	215.5	556.8
TH–FC	1	−172.669049	1.0694	1.1757	1.2006	153.1	173.5	3379.2	2214.5	1263.0	540.6	354.5	540.8
TH–FC+FO	1	−172.669049	1.0694	1.1757	1.2006	153.1	173.5	3379.2	2214.5	1263.0	540.6	354.5	540.8

Table S2 (continued)

Functional	Group	Energy	r_e(H–C)	r_e(C–N)	r_e(N–O)	θ_e(H–C–N)	θ_e(C–N–O)	$\omega_1(a')$	$\omega_2(a')$	$\omega_3(a')$	$\omega_4(a')$	$\omega_5(a')$	$\omega_6(a'')$
TH–FCO	1	−172.727129	1.0688	1.1724	1.1965	154.8	174.1	3381.5	2229.9	1273.2	545.0	330.4	545.3
TH–FL	1	−172.259283	1.0687	1.1584	1.1830	175.0	179.0	3414.9	2327.7	1331.3	568.4	81.4	568.5
TPSS	2	−168.688258	1.0647	1.1701	1.2029	157.5	174.7	3427.1	2246.0	1272.2	549.1	290.1	549.5
TPSS–D3(BJ)2B	2	−168.690108	1.0646	1.1701	1.2028	157.4	174.7	3427.3	2246.1	1272.5	549.0	291.9	549.4
TPSS–D3(BJ)ATM	2	−168.690109	1.0646	1.1701	1.2028	157.4	174.7	3427.3	2246.1	1272.5	549.0	291.8	549.4
TPSS–D3(0)2B	2	−168.688612	1.0648	1.1702	1.2030	157.6	174.7	3425.7	2244.9	1271.1	549.1	289.1	549.5
TPSS–D3(0)ATM	2	−168.688612	1.0648	1.1702	1.2030	157.6	174.7	3425.7	2244.9	1271.1	549.1	289.1	549.5
TPSSh–D3(BJ)2B	3	−168.662562	1.0601	1.1584	1.1997	175.1	178.9	3495.2	2310.6	1290.0	563.7	85.4	561.7
TPSSLYP1W	1	−168.909296	1.0614	1.1691	1.2120	158.1	174.7	3427.7	2210.3	1229.7	536.3	286.9	536.2
TPSS–NL	2	−168.572241	1.0653	1.1703	1.2024	157.3	174.6	3423.0	2247.0	1274.4	548.6	293.0	549.0
VSXC	2	−168.717425	1.0611	1.1682	1.1953	158.6	175.1	3476.6	2281.5	1299.7	560.3	278.9	560.5
VV10	1	−168.915700	1.0650	1.1711	1.2059	161.6	175.8	3417.3	2237.8	1260.9	538.6	240.2	539.3
XLYP	1	−168.768597	1.0656	1.1730	1.2115	159.5	175.3	3394.7	2206.9	1236.0	529.9	268.5	529.9
XLYP–D3(BJ)2B	1	−168.773049	1.0655	1.1730	1.2112	159.2	175.2	3395.3	2207.8	1237.4	529.6	272.3	529.6
XLYP–D3(BJ)ATM	1	−168.773049	1.0655	1.1730	1.2112	159.2	175.2	3395.3	2207.8	1237.4	529.6	272.3	529.6
XLYP–D3(0)2B	1	−168.769331	1.0659	1.1731	1.2119	160.1	175.4	3391.9	2205.0	1233.5	530.1	260.2	530.1
XLYP–D3(0)ATM	1	−168.769331	1.0659	1.1731	1.2119	160.1	175.4	3391.9	2205.0	1233.5	530.1	260.2	530.1
ZLP	1	−169.151958	1.0576	1.1643	1.1954	158.1	175.0	3457.0	2267.0	1284.4	549.3	295.6	549.5

Table S3. DFT linear minima for HCNO: electronic energies (E_h), geometric structures (Å, °), and harmonic vibrational frequencies (cm^{-1})

Functional	Group	Energy	r_e(H–C)	r_e(C–N)	r_e(N–O)	θ_e(H–C–N)	θ_e(C–N–O)	$\omega_1(\sigma)$	$\omega_2(\sigma)$	$\omega_3(\sigma)$	$\omega_{4a}(\pi)$	$\omega_{4b}(\pi)$	$\omega_{5a}(\pi)$	$\omega_{5b}(\pi)$
B1LYP	3	−168.593491	1.0581	1.1519	1.1987	180	180	3508.0	2330.9	1291.6	568.8	568.8	277.2	277.2
B1LYP–D3(BJ)2B	3	−168.596090	1.0580	1.1519	1.1986	180	180	3508.8	2331.5	1292.1	568.7	568.7	275.9	275.9
B1LYP–D3(BJ)ATM	3	−168.596090	1.0580	1.1519	1.1986	180	180	3508.8	2331.5	1292.1	568.7	568.7	275.9	275.9
B1LYP–D3(0)2B	3	−168.593968	1.0584	1.1522	1.1989	180	180	3504.7	2328.8	1289.9	569.0	569.0	279.2	279.2
B1LYP–D3(0)ATM	3	−168.593968	1.0584	1.1522	1.1989	180	180	3504.7	2328.8	1289.9	569.0	569.0	279.2	279.2
B1PW91	3	−168.610094	1.0595	1.1519	1.1908	180	180	3519.4	2363.2	1330.5	581.1	581.1	269.3	269.3
B1WC	3	−168.213990	1.0637	1.1563	1.1894	180	180	3481.4	2350.8	1334.7	575.8	575.8	210.3	210.3
B2GP–PLYP	6	−168.547176	1.0558	1.1585	1.1957	180	180	3536.7	2302.2	1304.3	567.5	567.1	122.9	122.4
B2GP–PLYP–D3(BJ)2B	6	−168.548007	1.0557	1.1585	1.1957	180	180	3536.6	2302.5	1304.5	566.9	566.9	121.8	121.8
B2GP–PLYP–D3(BJ)ATM	6	−168.548007	1.0557	1.1585	1.1957	180	180	3536.9	2302.3	1304.4	566.6	566.3	118.9	118.3
B2GP–PLYP–D3(0)2B	6	−168.547352	1.0558	1.1586	1.1958	180	180	3534.7	2301.4	1303.6	567.0	567.0	124.7	124.7
B2GP–PLYP–D3(0)ATM	6	−168.547352	1.0558	1.1586	1.1958	180	180	3535.4	2301.3	1303.6	567.2	567.1	125.2	124.4
B2GP–PLYP–NL	6	−168.502086	1.0559	1.1586	1.1956	180	180	3535.7	2302.4	1304.6	566.5	566.3	121.6	121.0
B3LYP	3	−168.659719	1.0591	1.1542	1.1989	180	180	3496.5	2321.3	1290.5	564.8	564.8	239.9	239.9
B3LYP5	3	−168.577743	1.0593	1.1545	1.1992	180	180	3494.1	2319.7	1289.6	564.5	564.5	238.7	238.7
B3LYP–D3(BJ)2B	3	−168.662312	1.0590	1.1542	1.1989	180	180	3497.5	2321.9	1291.0	564.7	564.7	238.3	238.3
B3LYP–D3(BJ)ATM	3	−168.662312	1.0590	1.1542	1.1989	180	180	3497.5	2321.9	1291.0	564.7	564.7	238.3	238.3
B3LYP–D3M(BJ)2B	3	−168.663069	1.0589	1.1541	1.1988	180	180	3497.8	2322.2	1291.3	564.6	564.6	237.8	237.8
B3LYP–D3M(BJ)ATM	3	−168.663070	1.0589	1.1541	1.1988	180	180	3497.8	2322.2	1291.3	564.6	564.6	237.8	237.8
B3LYP–D3M(0)2B	3	−168.660665	1.0594	1.1546	1.1993	180	180	3493.0	2318.4	1288.6	565.1	565.1	243.5	243.5
B3LYP–D3M(0)ATM	3	−168.660666	1.0594	1.1546	1.1993	180	180	3493.0	2318.4	1288.6	565.1	565.1	243.5	243.5
B3LYP–D3(0)2B	3	−168.660172	1.0592	1.1544	1.1991	180	180	3493.9	2319.4	1289.1	564.9	564.9	241.5	241.5
B3LYP–D3(0)ATM	3	−168.660172	1.0592	1.1544	1.1991	180	180	3493.9	2319.4	1289.1	564.9	564.9	241.5	241.5
B3LYP–NL	3	−168.537140	1.0596	1.1544	1.1984	180	180	3493.0	2322.8	1293.2	564.7	564.4	237.0	236.4
B3LYPS	3	−168.569067	1.0608	1.1573	1.2002	180	180	3478.0	2306.9	1285.7	559.4	559.4	193.0	193.0
B3P86	3	−168.685187	1.0614	1.1549	1.1938	180	180	3494.9	2340.7	1316.2	572.5	572.5	228.2	228.2
B3P86–D3(BJ)2B	3	−168.686711	1.0613	1.1549	1.1937	180	180	3495.4	2340.9	1316.4	572.4	572.4	227.3	227.3
B3P86–D3(BJ)ATM	3	−168.686711	1.0613	1.1549	1.1937	180	180	3495.4	2340.9	1316.3	572.4	572.4	227.3	227.3
B3P86–D3(0)2B	3	−168.685546	1.0615	1.1551	1.1939	180	180	3493.2	2339.4	1315.1	572.5	572.5	228.9	228.9
B3P86–D3(0)ATM	3	−168.685546	1.0615	1.1551	1.1939	180	180	3493.2	2339.4	1315.1	572.5	572.5	228.9	228.9
B3PW91	3	−168.589562	1.0604	1.1544	1.1928	180	180	3503.6	2346.2	1320.5	574.3	574.3	232.4	232.4

Table S3 (continued)

Functional	Group	Energy	r_e(H–C)	r_e(C–N)	r_e(N–O)	θ_e(H–C–N)	θ_e(C–N–O)	$\omega_1(\sigma)$	$\omega_2(\sigma)$	$\omega_3(\sigma)$	$\omega_{4\,a}(\pi)$	$\omega_{4\,b}(\pi)$	$\omega_{5\,a}(\pi)$	$\omega_{5\,b}(\pi)$
B3PW91–D3(BJ)2B	3	−168.592121	1.0603	1.1543	1.1927	180	180	3504.5	2346.8	1320.9	574.2	574.2	230.8	230.8
B3PW91–D3(BJ)ATM	3	−168.592121	1.0603	1.1543	1.1927	180	180	3504.5	2346.8	1320.9	574.2	574.2	230.8	230.8
B3PW91–D3(0)2B	3	−168.590054	1.0606	1.1546	1.1929	180	180	3500.9	2344.3	1318.9	574.4	574.4	233.8	233.8
B3PW91–D3(0)ATM	3	−168.590054	1.0606	1.1546	1.1929	180	180	3500.9	2344.3	1318.9	574.4	574.4	233.8	233.8
B3PW91–NL	3	−168.455721	1.0610	1.1546	1.1922	180	180	3499.7	2347.9	1323.1	573.7	573.7	228.8	228.0
B5050LYP	3	−168.525822	1.0530	1.1372	1.1868	180	180	3582.9	2423.2	1341.2	604.8	604.8	434.8	434.8
B86B95	3	−168.642234	1.0581	1.1519	1.1913	180	180	3519.1	2356.9	1325.8	580.5	580.5	273.7	273.7
B88B95	3	−168.597242	1.0580	1.1509	1.1897	180	180	3519.8	2362.5	1331.1	582.7	582.7	278.3	278.3
B88B95–D3(BJ)2B	3	−168.598970	1.0579	1.1509	1.1896	180	180	3520.3	2362.9	1331.3	582.6	582.6	277.5	277.5
B88B95–D3(BJ)ATM	3	−168.598970	1.0579	1.1509	1.1896	180	180	3520.3	2362.9	1331.3	582.6	582.6	277.5	277.5
B88B95–D3(0)2B	3	−168.597658	1.0582	1.1511	1.1899	180	180	3516.5	2360.5	1329.4	582.9	582.9	280.2	280.2
B88B95–D3(0)ATM	3	−168.597658	1.0582	1.1511	1.1899	180	180	3516.5	2360.5	1329.4	582.9	582.9	280.2	280.2
B97–0	3	−168.599404	1.0612	1.1572	1.1995	180	180	3489.6	2320.6	1294.2	563.6	563.6	178.9	178.9
B97–1	3	−168.606971	1.0609	1.1565	1.1999	180	180	3491.7	2322.8	1293.6	564.3	564.3	194.2	194.2
B97–1–D3(BJ)2B	3	−168.608628	1.0609	1.1565	1.1998	180	180	3492.2	2323.2	1294.0	564.2	564.2	193.1	193.1
B97–1–D3(BJ)ATM	3	−168.608628	1.0609	1.1565	1.1998	180	180	3492.2	2323.2	1293.9	564.2	564.2	193.1	193.1
B97–1–D3(0)2B	3	−168.607340	1.0611	1.1567	1.2000	180	180	3488.9	2321.0	1292.1	564.4	564.4	196.6	196.6
B97–1–D3(0)ATM	3	−168.607340	1.0611	1.1567	1.2000	180	180	3488.9	2321.0	1292.1	564.4	564.4	196.6	196.6
B97–1P	3	−168.607979	1.0610	1.1583	1.1970	180	180	3490.7	2321.7	1301.5	563.3	563.3	128.1	128.1
B97–2	3	−168.596168	1.0584	1.1530	1.1898	180	180	3526.1	2359.5	1330.3	578.1	578.1	234.2	234.2
B97–2–D3(BJ)2B	3	−168.600827	1.0582	1.1529	1.1896	180	180	3527.8	2361.1	1331.7	577.7	577.7	231.6	231.6
B97–2–D3(BJ)ATM	3	−168.600827	1.0582	1.1529	1.1896	180	180	3527.8	2361.1	1331.7	577.7	577.7	231.6	231.6
B97–2–D3(0)2B	3	−168.596713	1.0587	1.1533	1.1901	180	180	3521.9	2356.8	1328.1	578.2	578.2	237.1	237.1
B97–2–D3(0)ATM	3	−168.596713	1.0587	1.1533	1.1901	180	180	3521.9	2356.8	1328.1	578.2	578.2	237.1	237.1
B97–3	3	−168.606677	1.0585	1.1518	1.1941	180	180	3521.9	2354.2	1316.9	577.5	577.5	268.8	268.8
B97–K	3	−168.604046	1.0594	1.1499	1.2038	180	180	3527.3	2350.1	1285.5	577.9	577.9	349.5	349.5
BB1K	3	−168.573599	1.0550	1.1430	1.1839	180	180	3561.3	2409.7	1356.6	601.5	601.5	370.0	370.0
BHandH	3	−167.549572	1.0590	1.1372	1.1769	180	180	3550.0	2452.9	1390.2	620.1	620.1	436.5	436.5
BHandHLYP	3	−168.550788	1.0529	1.1377	1.1886	180	180	3581.5	2416.7	1332.9	602.1	602.1	433.0	433.0
BMK	4	−168.563164	1.0623	1.1493	1.2035	180	180	3494.2	2368.1	1304.7	586.2	586.2	316.3	316.3
BMK–D3(BJ)2B	4	−168.564687	1.0623	1.1493	1.2035	180	180	3494.6	2368.3	1305.0	586.1	586.1	315.8	315.8

Table S3 (continued)

Functional	Group	Energy	r_e(H–C)	r_e(C–N)	r_e(N–O)	θ_e(H–C–N)	θ_e(C–N–O)	$\omega_1(\sigma)$	$\omega_2(\sigma)$	$\omega_3(\sigma)$	$\omega_{4a}(\pi)$	$\omega_{4b}(\pi)$	$\omega_{5a}(\pi)$	$\omega_{5b}(\pi)$
BMK–D3(BJ)ATM	4	−168.564687	1.0623	1.1493	1.2035	180	180	3494.6	2368.3	1305.0	586.1	586.1	315.8	315.8
BMK–D3(0)2B	4	−168.563646	1.0626	1.1495	1.2037	180	180	3490.7	2365.7	1302.8	586.4	586.4	318.3	318.3
BMK–D3(0)ATM	4	−168.563646	1.0626	1.1495	1.2037	180	180	3490.7	2365.7	1302.9	586.4	586.4	318.3	318.3
CAM–B3LYP	5	−168.586627	1.0597	1.1475	1.1952	180	180	3504.1	2359.5	1311.4	579.5	579.5	358.1	358.1
CAM–B3LYP–D3(BJ)2B	5	−168.587677	1.0597	1.1475	1.1951	180	180	3504.4	2359.7	1311.6	579.5	579.5	357.7	357.7
CAM–B3LYP–D3(BJ)ATM	5	−168.587677	1.0597	1.1475	1.1951	180	180	3504.4	2359.7	1311.6	579.5	579.5	357.7	357.7
CAM–B3LYP–D3(0)2B	5	−168.586937	1.0599	1.1477	1.1953	180	180	3502.1	2358.2	1310.4	579.6	579.6	359.1	359.1
CAM–B3LYP–D3(0)ATM	5	−168.586938	1.0599	1.1477	1.1953	180	180	3502.1	2358.2	1310.4	579.6	579.6	359.1	359.1
CAM–LDA0	0	−167.864765	1.0626	1.1455	1.1822	180	180	3503.3	2405.0	1366.6	599.0	599.0	366.4	366.4
CAP0	3	−168.371991	1.0617	1.1530	1.1900	180	180	3508.3	2364.8	1335.6	582.4	582.4	261.9	261.9
DLDF	4	−169.687860	1.0522	1.1414	1.2028	180	180	3560.9	2371.1	1270.4	590.4	590.4	460.0	460.0
DLDF+D09	4	−169.687860	1.0522	1.1414	1.2028	180	180	3560.8	2371.0	1270.3	590.4	590.4	460.5	460.2
DLDF+D10	4	−169.687860	1.0522	1.1414	1.2028	180	180	3560.8	2370.9	1270.4	590.3	590.2	460.1	459.8
DSD–PBEB95	6	−168.474785	1.0570	1.1597	1.1926	180	180	3530.8	2309.2	1317.6	570.3	570.3	51.6	51.6
DSD–PBEB95–D3(BJ)	6	−168.449438	1.0575	1.1582	1.1935	180	180	3528.0	2313.6	1315.3	570.3	570.2	114.1	112.5
DSD–PBEB95–NL	6	−168.416176	1.0576	1.1583	1.1935	180	180	3525.9	2311.7	1313.9	570.1	570.0	112.5	111.7
EDF2	3	−168.566184	1.0583	1.1525	1.1925	180	180	3500.5	2339.7	1312.5	570.4	570.4	235.9	235.9
HF	0	−167.713383	1.0526	1.1224	1.1899	180	180	3640.2	2497.9	1310.5	646.8	646.8	605.3	605.3
HF–3c	0	−167.870218	1.0440	1.0893	1.0625	180	180	3646.6	2640.0	1639.1	827.9	827.9	553.9	553.9
HF+D	0	−167.713383	1.0526	1.1224	1.1899	180	180	3640.1	2497.8	1310.4	646.6	646.5	605.7	605.6
HF–D3(BJ)2B	0	−167.729463	1.0512	1.1215	1.1886	180	180	3652.8	2506.6	1318.2	644.5	644.5	599.6	599.6
HF–D3(BJ)ATM	0	−167.729464	1.0512	1.1215	1.1886	180	180	3652.8	2506.6	1318.2	644.5	644.5	599.6	599.6
HF–D3M(BJ)2B	0	−167.741840	1.0505	1.1207	1.1872	180	180	3658.2	2513.5	1323.5	643.1	643.1	597.3	597.3
HF–D3M(BJ)ATM	0	−167.741840	1.0505	1.1207	1.1872	180	180	3658.2	2513.5	1323.5	643.1	643.1	597.3	597.3
HF–D3M(0)2B	0	−167.730703	1.0513	1.1207	1.1886	180	180	3638.5	2502.6	1309.6	640.4	640.4	595.8	595.8
HF–D3M(0)ATM	0	−167.730704	1.0513	1.1207	1.1886	180	180	3638.5	2502.6	1309.6	640.4	640.4	595.8	595.8
HF–D3(0)2B	0	−167.713845	1.0527	1.1225	1.1900	180	180	3637.9	2496.2	1309.0	647.1	647.1	605.3	605.3
HF–D3(0)ATM	0	−167.713845	1.0527	1.1225	1.1900	180	180	3637.9	2496.2	1309.0	647.1	647.1	605.3	605.3
HJS–B97x	5	−167.850958	1.0656	1.1551	1.1865	180	180	3492.5	2376.7	1355.1	587.4	587.4	252.5	252.5
HJS–PBE	5	−168.466427	1.0608	1.1530	1.1902	180	180	3512.9	2364.0	1334.7	581.5	581.5	261.1	261.1
HJS–PBEsol	5	−167.919460	1.0643	1.1533	1.1852	180	180	3497.2	2382.1	1358.3	588.9	588.9	267.7	267.7

Table S3 (continued)

Functional	Group	Energy	r_e(H–C)	r_e(C–N)	r_e(N–O)	θ_e(H–C–N)	θ_e(C–N–O)	$\omega_1(\sigma)$	$\omega_2(\sigma)$	$\omega_3(\sigma)$	$\omega_{4\,a}(\pi)$	$\omega_{4\,b}(\pi)$	$\omega_{5\,a}(\pi)$	$\omega_{5\,b}(\pi)$
hPBEint	3	−168.037051	1.0661	1.1589	1.1912	180	180	3471.8	2345.1	1332.2	574.2	574.2	192.6	192.6
HSE03	5	−168.724479	1.0593	1.1517	1.1897	180	180	3525.0	2370.0	1335.9	582.3	582.3	278.4	278.4
HSE03−D3(BJ)2B	5	−168.725593	1.0592	1.1517	1.1897	180	180	3523.5	2368.6	1336.0	582.5	582.5	272.8	272.8
HSE03−D3(BJ)ATM	5	−168.725593	1.0592	1.1517	1.1897	180	180	3523.5	2368.7	1336.0	582.5	582.5	272.8	272.8
HSE03−D3(0)2B	5	−168.724742	1.0594	1.1518	1.1898	180	180	3521.6	2367.4	1335.0	582.6	582.6	274.4	274.4
HSE03−D3(0)ATM	5	−168.724742	1.0594	1.1518	1.1898	180	180	3521.6	2367.4	1335.0	582.6	582.6	274.4	274.4
HSE06	5	−168.484570	1.0604	1.1525	1.1900	180	180	3512.4	2363.2	1333.2	580.4	580.4	265.7	265.7
HSE06−D3(BJ)2B	5	−168.485435	1.0604	1.1525	1.1900	180	180	3514.1	2364.1	1334.4	582.0	582.0	268.1	268.1
HSE06−D3(BJ)ATM	5	−168.485435	1.0604	1.1525	1.1900	180	180	3514.1	2364.1	1334.4	582.0	582.0	268.1	268.1
HSE06−D3(0)2B	5	−168.484713	1.0604	1.1525	1.1900	180	180	3513.8	2363.8	1334.2	581.9	581.9	267.9	267.9
HSE06−D3(0)ATM	5	−168.484713	1.0604	1.1525	1.1900	180	180	3513.8	2363.8	1334.2	581.9	581.9	267.9	267.9
KMLYP	3	−168.286468	1.0539	1.1327	1.1760	180	180	3594.4	2473.0	1391.6	626.4	626.4	467.2	467.2
KSDT	0	−167.323475	1.0718	1.1638	1.1894	180	180	3403.2	2320.5	1327.1	563.4	563.4	84.9	84.9
LC−BOP	1	−168.251930	1.0616	1.1399	1.1911	180	180	3519.2	2412.1	1343.6	607.1	607.1	472.4	472.4
LC−VV10	1	−168.438400	1.0616	1.1438	1.1899	180	180	3534.7	2413.5	1355.7	604.4	604.4	445.7	445.7
LDA0	0	−167.429212	1.0661	1.1515	1.1851	180	180	3470.3	2373.9	1352.0	588.5	588.5	301.4	301.4
LRC−ωPBE	5	−168.515086	1.0657	1.1553	1.1957	180	180	3472.5	2348.0	1323.3	574.1	574.1	313.1	313.1
LRC−ωPBEh	5	−168.486415	1.0624	1.1518	1.1904	180	180	3504.4	2371.4	1338.8	583.2	583.2	313.9	313.9
M05	4	−168.575011	1.0599	1.1562	1.1866	180	180	3519.2	2376.5	1360.7	594.1	594.1	241.4	241.4
M05−2X	4	−168.628342	1.0582	1.1468	1.1930	180	180	3537.4	2374.4	1327.0	590.4	590.4	388.2	388.2
M05−2X−D3(0)2B	4	−168.628378	1.0582	1.1468	1.1931	180	180	3537.6	2374.6	1327.2	590.2	590.2	388.6	388.6
M05−2X−D3(0)ATM	4	−168.628378	1.0582	1.1468	1.1931	180	180	3537.3	2374.4	1327.0	590.4	590.4	388.4	388.4
M05−D3(0)2B	4	−168.575189	1.0599	1.1563	1.1867	180	180	3518.2	2375.8	1360.2	594.2	594.2	242.2	242.2
M05−D3(0)ATM	4	−168.575189	1.0599	1.1563	1.1867	180	180	3518.2	2375.8	1360.2	594.2	594.2	242.2	242.2
M06	4	−168.567996	1.0586	1.1513	1.1878	180	180	3504.3	2372.5	1348.0	591.7	591.7	323.3	323.3
M06−2X	4	−168.587792	1.0600	1.1477	1.1952	180	180	3511.1	2369.5	1318.1	592.9	592.9	398.6	398.6
M06−2X−D3(0)2B	4	−168.587801	1.0600	1.1477	1.1952	180	180	3510.6	2369.4	1318.1	592.7	592.7	398.7	398.7
M06−2X−D3(0)ATM	4	−168.587801	1.0600	1.1477	1.1952	180	180	3511.0	2369.5	1318.1	592.9	592.9	398.7	398.7
M06−D3(0)2B	4	−168.568054	1.0586	1.1513	1.1878	180	180	3504.5	2372.6	1348.1	591.7	591.7	323.2	323.2
M06−D3(0)ATM	4	−168.568054	1.0586	1.1513	1.1878	180	180	3504.5	2372.6	1348.1	591.7	591.7	323.2	323.2
M06−HF	4	−168.606123	1.0602	1.1381	1.2005	180	180	3522.7	2375.3	1265.8	616.8	616.8	510.2	510.2

Table S3 (continued)

Functional	Group	Energy	r_e(H–C)	r_e(C–N)	r_e(N–O)	θ_e(H–C–N)	θ_e(C–N–O)	$\omega_1(\sigma)$	$\omega_2(\sigma)$	$\omega_3(\sigma)$	$\omega_{4a}(\pi)$	$\omega_{4b}(\pi)$	$\omega_{5a}(\pi)$	$\omega_{5b}(\pi)$
M06–HF–D3(0)2B	4	−168.606153	1.0602	1.1381	1.2005	180	180	3522.6	2375.2	1265.8	616.8	616.8	510.4	510.4
M06–HF–D3(0)ATM	4	−168.606153	1.0602	1.1381	1.2005	180	180	3522.6	2375.2	1265.8	616.8	616.8	510.4	510.4
M08–HX	4	−168.594125	1.0625	1.1476	1.1919	180	180	3506.6	2373.4	1322.6	597.7	597.7	399.4	399.4
M08–HX–D3(0)2B	4	−168.594133	1.0625	1.1476	1.1919	180	180	3506.5	2373.3	1322.5	597.7	597.7	399.5	399.5
M08–HX–D3(0)ATM	4	−168.594133	1.0625	1.1476	1.1919	180	180	3506.5	2373.3	1322.5	597.7	597.7	399.5	399.5
M08–SO	4	−168.536293	1.0661	1.1509	1.1972	180	180	3471.4	2349.2	1310.5	587.8	587.8	422.3	422.3
M11	5	−168.596449	1.0645	1.1492	1.1924	180	180	3474.3	2354.4	1324.5	595.2	595.2	412.1	412.1
M11–D3(BJ)2B	5	−168.596569	1.0645	1.1492	1.1924	180	180	3474.3	2354.4	1324.5	595.2	595.2	412.1	412.1
M11–D3(BJ)ATM	5	−168.596569	1.0645	1.1492	1.1924	180	180	3474.3	2354.4	1324.5	595.2	595.2	412.1	412.1
M11–D3(0)2B	5	−168.596586	1.0645	1.1492	1.1925	180	180	3473.2	2353.8	1324.0	595.3	595.3	412.5	412.5
M11–D3(0)ATM	5	−168.596586	1.0645	1.1492	1.1925	180	180	3473.2	2353.7	1323.9	595.3	595.3	412.5	412.5
mB3LYP–RC04	3	−168.944305	1.0552	1.1512	1.1955	180	180	3528.7	2343.0	1304.7	569.6	569.6	257.1	257.1
MGGA–MVS	2	−168.567106	1.0617	1.1591	1.1836	180	180	3515.4	2392.4	1371.1	603.3	603.3	166.3	166.3
MGGA–MVSh	4	−168.537119	1.0576	1.1471	1.1770	180	180	3567.8	2447.6	1402.0	622.9	622.9	344.1	344.1
MN12–L	2	−168.503507	1.0565	1.1468	1.1885	180	180	3545.2	2375.0	1318.3	583.7	583.7	33.0	33.0
MN12–L–D3(BJ)2B	2	−168.503728	1.0565	1.1468	1.1885	180	180	3545.2	2375.0	1318.3	583.7	583.7	33.1	33.1
MN12–L–D3(BJ)ATM	2	−168.503728	1.0565	1.1468	1.1885	180	180	3545.2	2375.0	1318.3	583.7	583.7	32.9	32.9
MN12–L–D3(0)2B	2	−168.503715	1.0567	1.1469	1.1886	180	180	3543.6	2373.9	1317.3	583.3	583.3	37.5	37.5
MN12–L–D3(0)ATM	2	−168.503715	1.0567	1.1469	1.1886	180	180	3543.6	2374.0	1317.4	583.7	583.7	39.8	39.8
MN12–SX	5	−168.526583	1.0603	1.1471	1.1948	180	180	3523.1	2365.2	1304.4	577.8	577.8	274.6	274.6
MN12–SX–D3(BJ)2B	5	−168.526876	1.0603	1.1471	1.1948	180	180	3523.1	2365.2	1304.4	577.8	577.8	274.5	274.5
MN12–SX–D3(BJ)ATM	5	−168.526876	1.0603	1.1471	1.1948	180	180	3523.1	2365.2	1304.4	577.8	577.8	274.5	274.5
MN12–SX–D3(0)2B	5	−168.526763	1.0604	1.1472	1.1949	180	180	3521.8	2364.3	1303.6	577.9	577.9	275.4	275.4
MN12–SX–D3(0)ATM	5	−168.526763	1.0604	1.1472	1.1949	180	180	3521.7	2364.3	1303.6	577.9	577.9	275.4	275.4
MN15	5	−168.552569	1.0606	1.1530	1.1978	180	180	3520.1	2348.8	1318.7	579.7	579.7	378.0	378.0
MN15–D3(BJ)2B	5	−168.552624	1.0606	1.1530	1.1978	180	180	3520.1	2348.8	1318.7	579.7	579.7	378.0	378.0
MN15–D3(BJ)ATM	5	−168.552573	1.0606	1.1530	1.1978	180	180	3520.0	2348.8	1318.7	579.7	579.7	378.0	378.0
MPW1B95	3	−168.590681	1.0572	1.1490	1.1881	180	180	3529.1	2373.9	1337.8	587.0	587.0	303.1	303.1
MPW1B95–D3(BJ)2B	3	−168.591407	1.0572	1.1489	1.1881	180	180	3529.2	2374.0	1337.8	587.0	587.0	302.9	302.9
MPW1B95–D3(BJ)ATM	3	−168.591407	1.0572	1.1489	1.1881	180	180	3529.2	2374.0	1337.8	587.0	587.0	302.9	302.9
MPW1B95–D3(0)2B	3	−168.590932	1.0573	1.1491	1.1882	180	180	3527.1	2372.7	1336.8	587.1	587.1	304.2	304.2

Table S3 (continued)

Functional	Group	Energy	r_e(H–C)	r_e(C–N)	r_e(N–O)	θ_e(H–C–N)	θ_e(C–N–O)	$\omega_1(\sigma)$	$\omega_2(\sigma)$	$\omega_3(\sigma)$	$\omega_{4\,a}(\pi)$	$\omega_{4\,b}(\pi)$	$\omega_{5\,a}(\pi)$	$\omega_{5\,b}(\pi)$
MPW1B95–D3(0)ATM	3	−168.590932	1.0573	1.1491	1.1882	180	180	3527.1	2372.7	1336.7	587.1	587.1	304.2	304.2
MPW1K	3	−168.579342	1.0557	1.1417	1.1833	180	180	3570.9	2423.3	1363.0	605.1	605.1	387.8	387.8
mPW1LYP	3	−168.592268	1.0580	1.1517	1.1984	180	180	3508.3	2332.1	1292.7	569.1	569.1	280.5	280.5
mPW1LYP–D3(0)2B	3	−168.592700	1.0582	1.1519	1.1986	180	180	3505.0	2329.9	1290.9	569.2	569.2	282.4	282.4
mPW1LYP–D3(0)ATM	3	−168.592700	1.0582	1.1519	1.1986	180	180	3505.0	2329.9	1290.9	569.2	569.2	282.4	282.4
mPW1PBE	3	−168.553137	1.0600	1.1521	1.1902	180	180	3516.7	2365.0	1333.5	581.7	581.7	269.6	269.6
mPW1PW	3	−168.608619	1.0593	1.1517	1.1904	180	180	3519.7	2364.3	1331.4	581.3	581.3	272.8	272.8
mPW1PW–D3(BJ)2B	3	−168.610535	1.0593	1.1517	1.1904	180	180	3520.3	2364.7	1331.8	581.2	581.2	271.8	271.8
mPW1PW–D3(BJ)ATM	3	−168.610535	1.0593	1.1517	1.1904	180	180	3520.3	2364.7	1331.8	581.2	581.2	271.8	271.8
mPW1PW–D3(0)2B	3	−168.609009	1.0595	1.1519	1.1906	180	180	3517.5	2362.8	1330.3	581.4	581.4	274.0	274.0
mPW1PW–D3(0)ATM	3	−168.609009	1.0595	1.1519	1.1906	180	180	3517.5	2362.7	1330.2	581.4	581.4	274.0	274.0
MPW3LYP	3	−168.599421	1.0588	1.1531	1.1980	180	180	3500.1	2327.8	1294.6	567.2	567.2	258.9	258.9
mPW3PW	3	−168.671419	1.0600	1.1539	1.1922	180	180	3506.5	2349.0	1322.5	574.9	574.9	237.9	237.9
MPWB1K	3	−168.569257	1.0545	1.1417	1.1829	180	180	3567.2	2417.0	1360.7	604.3	604.3	382.7	382.7
MPWB1K–D3(BJ)2B	3	−168.569976	1.0545	1.1417	1.1829	180	180	3567.3	2417.1	1360.8	604.3	604.3	382.6	382.6
MPWB1K–D3(BJ)ATM	3	−168.569976	1.0545	1.1417	1.1829	180	180	3567.3	2417.1	1360.8	604.3	604.3	382.6	382.6
MPWB1K–D3(0)2B	3	−168.569486	1.0546	1.1418	1.1830	180	180	3565.4	2415.9	1359.7	604.4	604.4	383.5	383.5
MPWB1K–D3(0)ATM	3	−168.569486	1.0546	1.1418	1.1830	180	180	3565.4	2415.9	1359.7	604.4	604.4	383.5	383.5
N12–SX	5	−168.575955	1.0559	1.1484	1.1881	180	180	3534.6	2362.2	1331.1	581.5	581.5	314.6	314.6
N12–SX–D3(BJ)2B	5	−168.576986	1.0559	1.1484	1.1881	180	180	3534.9	2362.3	1331.2	581.4	581.4	314.3	314.3
N12–SX–D3(BJ)ATM	5	−168.576986	1.0559	1.1484	1.1881	180	180	3534.9	2362.3	1331.2	581.4	581.4	314.3	314.3
N12–SX–D3(0)2B	5	−168.576300	1.0561	1.1486	1.1882	180	180	3532.1	2360.6	1329.7	581.6	581.6	316.1	316.1
N12–SX–D3(0)ATM	5	−168.576300	1.0561	1.1486	1.1882	180	180	3532.1	2360.6	1329.7	581.6	581.6	316.1	316.1
O3LYP	3	−168.608270	1.0606	1.1591	1.1938	180	180	3499.0	2332.2	1315.0	565.4	565.4	93.5	93.5
O3LYP–D3(BJ)2B	3	−168.610375	1.0605	1.1591	1.1937	180	180	3499.6	2332.6	1315.4	565.3	565.3	90.9	90.9
O3LYP–D3(BJ)ATM	3	−168.610375	1.0605	1.1591	1.1937	180	180	3499.6	2332.6	1315.4	565.3	565.3	91.0	91.0
O3LYP–D3(0)2B	3	−168.608713	1.0608	1.1593	1.1940	180	180	3495.9	2330.2	1313.4	565.6	565.6	99.4	99.4
O3LYP–D3(0)ATM	3	−168.608713	1.0608	1.1593	1.1940	180	180	3495.9	2330.2	1313.4	565.6	565.6	99.4	99.4
PBE0	3	−168.468080	1.0609	1.1528	1.1901	180	180	3513.0	2365.7	1335.8	582.1	582.1	269.2	269.2
PBE0–1/3	3	−168.463450	1.0590	1.1479	1.1866	180	180	3537.6	2393.7	1351.2	593.3	593.3	328.3	328.3
PBE0–2	6	−168.467780	1.0557	1.1567	1.1879	180	180	3559.7	2339.6	1342.6	582.0	582.0	163.0	162.4

Table S3 (continued)

Functional	Group	Energy	r_e(H–C)	r_e(C–N)	r_e(N–O)	θ_e(H–C–N)	θ_e(C–N–O)	$\omega_1(\sigma)$	$\omega_2(\sigma)$	$\omega_3(\sigma)$	$\omega_{4\,a}(\pi)$	$\omega_{4\,b}(\pi)$	$\omega_{5\,a}(\pi)$	$\omega_{5\,b}(\pi)$
PBE0–D3(BJ)2B	3	−168.469321	1.0608	1.1527	1.1901	180	180	3513.4	2365.9	1336.0	582.0	582.0	268.6	268.6
PBE0–D3(BJ)ATM	3	−168.469321	1.0608	1.1527	1.1901	180	180	3513.4	2365.9	1336.0	582.0	582.0	268.6	268.6
PBE0–D3M(BJ)2B	3	−168.469823	1.0608	1.1527	1.1900	180	180	3513.5	2366.1	1336.1	582.0	582.0	268.5	268.5
PBE0–D3M(BJ)ATM	3	−168.469823	1.0608	1.1527	1.1900	180	180	3513.5	2366.1	1336.1	582.0	582.0	268.5	268.5
PBE0–D3M(0)2B	3	−168.471431	1.0602	1.1519	1.1897	180	180	3514.9	2367.9	1337.0	582.9	582.9	272.6	272.6
PBE0–D3M(0)ATM	3	−168.471431	1.0602	1.1519	1.1897	180	180	3514.9	2367.9	1337.0	582.9	582.9	272.6	272.6
PBE0–D3(0)2B	3	−168.468352	1.0609	1.1529	1.1902	180	180	3511.7	2364.7	1335.1	582.1	582.1	269.9	269.9
PBE0–D3(0)ATM	3	−168.468352	1.0609	1.1529	1.1902	180	180	3511.7	2364.7	1335.1	582.1	582.1	269.9	269.9
PBE0–DH	6	−168.474041	1.0575	1.1498	1.1870	180	180	3550.8	2379.3	1346.1	590.2	590.2	295.4	295.3
PBE0–DH–D3(BJ)2B	6	−168.475349	1.0575	1.1497	1.1870	180	180	3550.9	2381.2	1348.7	590.2	590.0	294.9	294.5
PBE0–DH–D3(BJ)ATM	6	−168.475349	1.0575	1.1497	1.1870	180	180	3551.3	2379.7	1346.3	590.2	590.1	295.6	295.6
PBE0–DH–D3(0)2B	6	−168.474238	1.0576	1.1499	1.1871	180	180	3549.7	2378.8	1345.5	590.6	590.6	297.5	297.5
PBE0–DH–D3(0)ATM	6	−168.474238	1.0576	1.1499	1.1871	180	180	3549.6	2378.5	1345.4	590.9	590.9	296.8	296.2
PBE0–NL	3	−168.393511	1.0611	1.1529	1.1899	180	180	3511.0	2366.0	1336.8	581.5	581.4	267.9	267.7
PBE50	3	−168.456490	1.0556	1.1389	1.1805	180	180	3584.9	2446.8	1378.0	615.0	615.0	421.7	421.7
PBEh–3c	3	−168.296110	1.0586	1.1428	1.1819	180	180	3551.4	2421.9	1370.1	607.1	607.1	400.4	400.4
PW6B95	4	−168.853572	1.0559	1.1499	1.1912	180	180	3528.4	2357.6	1321.8	579.9	579.9	283.3	283.3
PW6B95–D3(BJ)2B	4	−168.854223	1.0558	1.1499	1.1911	180	180	3528.5	2357.7	1321.9	579.9	579.9	283.1	283.1
PW6B95–D3(BJ)ATM	4	−168.854223	1.0558	1.1499	1.1911	180	180	3528.5	2357.7	1321.9	579.9	579.9	283.1	283.1
PW6B95–D3(0)2B	4	−168.853776	1.0560	1.1500	1.1913	180	180	3526.8	2356.6	1321.0	580.0	580.0	284.4	284.4
PW6B95–D3(0)ATM	4	−168.853776	1.0560	1.1500	1.1913	180	180	3526.8	2356.6	1321.0	580.0	580.0	284.4	284.4
PW86B95	3	−168.715859	1.0570	1.1505	1.1914	180	180	3524.9	2359.0	1324.5	582.1	582.1	296.9	296.9
PWB6K	3	−168.717973	1.0528	1.1400	1.1829	180	180	3578.9	2421.5	1358.8	605.8	605.8	396.7	396.7
PWB6K–D3(BJ)2B	3	−168.718226	1.0528	1.1400	1.1829	180	180	3579.0	2421.5	1358.8	605.8	605.8	396.7	396.7
PWB6K–D3(BJ)ATM	3	−168.718227	1.0528	1.1400	1.1829	180	180	3579.0	2421.5	1358.8	605.8	605.8	396.7	396.7
PWB6K–D3(0)2B	3	−168.718094	1.0529	1.1400	1.1830	180	180	3578.0	2420.9	1358.3	605.9	605.9	397.1	397.1
PWB6K–D3(0)ATM	3	−168.718094	1.0529	1.1400	1.1830	180	180	3578.0	2420.9	1358.3	605.9	605.9	397.1	397.1
PWPB95	6	−168.577679	1.0577	1.1565	1.1946	180	180	3516.6	2314.7	1305.4	567.5	567.0	156.2	155.9
PWPB95–D3(BJ)2B	6	−168.578163	1.0577	1.1565	1.1946	180	180	3514.3	2314.7	1304.5	568.1	567.7	155.5	155.1
PWPB95–D3(BJ)ATM	6	−168.578163	1.0577	1.1565	1.1946	180	180	3516.8	2314.8	1305.5	567.5	567.3	156.1	154.9
PWPB95–D3(0)2B	6	−168.577847	1.0578	1.1566	1.1947	180	180	3516.1	2314.0	1304.8	567.9	567.9	159.4	159.4

Table S3 (continued)

Functional	Group	Energy	r_e(H–C)	r_e(C–N)	r_e(N–O)	θ_e(H–C–N)	θ_e(C–N–O)	$\omega_1(\sigma)$	$\omega_2(\sigma)$	$\omega_3(\sigma)$	$\omega_{4\,a}(\pi)$	$\omega_{4\,b}(\pi)$	$\omega_{5\,a}(\pi)$	$\omega_{5\,b}(\pi)$
PWPB95–D3(0)ATM	6	−168.577847	1.0578	1.1566	1.1947	180	180	3515.4	2314.0	1304.8	568.0	567.9	159.3	158.9
PWPB95–NL	6	−168.539286	1.0578	1.1566	1.1946	180	180	3515.9	2314.9	1305.7	567.2	567.2	156.3	155.3
revB3LYP	3	−168.525101	1.0598	1.1542	1.1977	180	180	3492.4	2325.2	1296.3	566.5	566.5	243.6	243.6
revPBE0	3	−168.573186	1.0610	1.1545	1.1931	180	180	3513.2	2354.4	1324.7	578.2	578.2	251.3	251.3
revPBE0–D3(BJ)2B	3	−168.576687	1.0608	1.1544	1.1930	180	180	3514.9	2355.4	1325.5	578.0	578.0	248.9	248.9
revPBE0–D3(BJ)ATM	3	−168.576687	1.0608	1.1544	1.1930	180	180	3514.9	2355.4	1325.4	578.0	578.0	248.9	248.9
revPBE0–D3(0)2B	3	−168.573843	1.0613	1.1548	1.1934	180	180	3508.9	2351.7	1322.7	578.4	578.4	254.6	254.6
revPBE0–D3(0)ATM	3	−168.573844	1.0613	1.1548	1.1934	180	180	3508.9	2351.7	1322.7	578.4	578.4	254.6	254.6
revPBE0–NL	3	−168.430847	1.0617	1.1547	1.1925	180	180	3509.1	2356.2	1327.8	578.1	577.6	247.1	246.9
revSCAN0	4	−168.538211	1.0566	1.1468	1.1833	180	180	3554.8	2413.3	1367.1	601.3	601.3	269.0	269.0
SB98–1a	3	−168.753728	1.0537	1.1459	1.1875	180	180	3532.5	2362.8	1322.1	579.2	579.2	281.4	281.4
SB98–1b	3	−167.980552	1.0654	1.1589	1.1950	180	180	3480.6	2331.7	1316.7	572.4	572.4	233.3	233.3
SB98–1c	3	−168.600573	1.0612	1.1573	1.1994	180	180	3490.2	2320.7	1294.7	563.7	563.7	179.8	179.8
SB98–2a	3	−168.951307	1.0582	1.1533	1.1988	180	180	3513.9	2334.4	1296.3	567.5	567.5	245.1	245.1
SB98–2b	3	−168.305340	1.0619	1.1544	1.1969	180	180	3487.7	2331.6	1302.2	569.9	569.9	243.8	243.8
SB98–2c	3	−168.590660	1.0604	1.1549	1.1987	180	180	3492.6	2326.4	1294.7	565.7	565.7	209.7	209.7
SCAN0	4	−168.546892	1.0565	1.1457	1.1871	180	180	3546.9	2398.2	1344.0	589.5	589.5	269.2	269.2
SOGGA11–X	3	−168.583400	1.0598	1.1497	1.1931	180	180	3545.4	2385.4	1333.2	589.9	589.9	332.0	332.0
SOGGA11–X–D3(BJ)2B	3	−168.585271	1.0597	1.1497	1.1931	180	180	3545.9	2385.7	1333.5	589.8	589.8	331.3	331.3
SOGGA11–X–D3(BJ)ATM	3	−168.585271	1.0597	1.1497	1.1931	180	180	3545.9	2385.8	1333.5	589.8	589.8	331.3	331.3
SOGGA11–X–D3(0)2B	3	−168.583812	1.0600	1.1499	1.1933	180	180	3542.2	2383.4	1331.5	590.0	590.0	333.6	333.6
SOGGA11–X–D3(0)ATM	3	−168.583812	1.0600	1.1499	1.1933	180	180	3542.2	2383.4	1331.5	590.0	590.0	333.6	333.6
SPW92	0	−167.348574	1.0711	1.1631	1.1886	180	180	3408.1	2324.6	1329.8	564.4	564.4	93.6	93.6
SVWN	0	−167.786290	1.0694	1.1617	1.1872	180	180	3422.0	2333.8	1335.4	566.4	566.4	109.0	109.0
Teter93	0	−167.350731	1.0712	1.1630	1.1884	180	180	3406.7	2324.3	1329.9	564.5	564.5	93.6	93.6
τ–HCTHh	4	−168.617296	1.0611	1.1575	1.1976	180	180	3484.6	2319.9	1297.6	561.9	561.9	141.5	141.5
TH–FL	1	−172.259284	1.0686	1.1581	1.1831	180	180	3417.0	2328.8	1331.2	568.5	568.5	24.2	24.2
TPSSh	3	−168.660903	1.0600	1.1582	1.1998	180	180	3497.2	2311.9	1289.2	563.9	563.9	52.3	52.3
TPSSh–D3(BJ)2B	3	−168.662563	1.0599	1.1581	1.1998	180	180	3497.4	2312.1	1289.6	563.8	563.8	46.3	46.3
TPSSh–D3(BJ)ATM	3	−168.662564	1.0599	1.1581	1.1998	180	180	3497.7	2312.2	1289.5	563.8	563.8	47.6	47.6
TPSSh–D3(0)2B	3	−168.661261	1.0601	1.1583	1.2000	180	180	3495.4	2310.6	1288.2	563.9	563.9	56.1	56.1

Table S3 (continued)

Functional	Group	Energy	r_e(H–C)	r_e(C–N)	r_e(N–O)	θ_e(H–C–N)	θ_e(C–N–O)	$\omega_1(\sigma)$	$\omega_2(\sigma)$	$\omega_3(\sigma)$	$\omega_{4\,a}(\pi)$	$\omega_{4\,b}(\pi)$	$\omega_{5\,a}(\pi)$	$\omega_{5\,b}(\pi)$
TPSSh–D3(0)ATM	3	−168.661261	1.0601	1.1583	1.2000	180	180	3495.4	2310.6	1288.2	563.9	563.9	56.1	56.1
tuned–CAM–B3LYP	5	−168.544510	1.0629	1.1585	1.1997	180	180	3456.2	2303.6	1289.4	556.4	556.4	207.7	207.7
ωB97	1	−168.619152	1.0633	1.1505	1.1973	180	180	3513.3	2373.1	1324.6	586.3	586.3	397.0	397.0
ωB97M–D3(BJ)	5	−168.693870	1.0590	1.1495	1.1992	180	180	3507.6	2346.7	1295.4	571.7	571.7	348.3	348.3
ωB97M–V	5	−168.600838	1.0595	1.1497	1.1990	180	180	3504.5	2346.4	1296.5	571.5	571.5	347.1	347.1
ωB97X	5	−168.604716	1.0611	1.1488	1.1938	180	180	3513.9	2371.4	1325.9	584.7	584.7	371.3	371.3
ωB97X–D	5	−168.593505	1.0598	1.1501	1.1924	180	180	3508.0	2358.9	1321.9	579.6	579.6	314.5	314.5
ωB97X–D3	5	−168.600164	1.0606	1.1495	1.1928	180	180	3513.0	2367.1	1325.7	582.4	582.4	343.0	343.0
ωB97X–D3(BJ)	5	−168.686050	1.0624	1.1505	1.1983	180	180	3507.7	2359.9	1310.7	579.1	579.1	363.8	363.8
ωB97X–D3(0)2B	5	−168.604864	1.0611	1.1488	1.1938	180	180	3513.9	2371.3	1325.8	584.6	584.6	370.9	370.9
ωB97X–D3(0)ATM	5	−168.604864	1.0611	1.1488	1.1938	180	180	3513.9	2371.3	1325.8	584.6	584.6	370.9	370.9
ωB97X–V	5	−168.592356	1.0629	1.1507	1.1982	180	180	3504.6	2359.3	1311.4	578.9	578.9	363.2	363.2
ωPBE	5	−168.521511	1.0629	1.1474	1.1920	180	180	3513.3	2391.6	1344.4	594.5	594.5	411.0	411.0
ωPBE0	5	−168.492069	1.0604	1.1440	1.1868	180	180	3537.4	2414.1	1358.4	601.2	601.2	409.5	409.5
ωPBE–D3(BJ)2B	5	−168.522820	1.0629	1.1474	1.1920	180	180	3513.7	2391.8	1344.5	594.4	594.4	410.5	410.5
ωPBE–D3(BJ)ATM	5	−168.522820	1.0629	1.1474	1.1920	180	180	3513.7	2391.7	1344.5	594.4	594.4	410.5	410.5
ωPBE–D3M(BJ)2B	5	−168.523469	1.0628	1.1473	1.1920	180	180	3514.3	2392.0	1344.7	594.4	594.4	410.0	410.0
ωPBE–D3M(BJ)ATM	5	−168.523469	1.0628	1.1473	1.1920	180	180	3514.3	2392.0	1344.7	594.4	594.4	410.0	410.0
ωPBE–D3M(0)2B	5	−168.521908	1.0631	1.1476	1.1922	180	180	3511.0	2390.0	1343.1	594.6	594.6	412.1	412.1
ωPBE–D3M(0)ATM	5	−168.521908	1.0631	1.1476	1.1922	180	180	3511.0	2390.0	1343.1	594.6	594.6	412.1	412.1
ωPBE–D3(0)2B	5	−168.521840	1.0631	1.1475	1.1922	180	180	3511.3	2390.2	1343.3	594.6	594.6	411.8	411.8
ωPBE–D3(0)ATM	5	−168.521840	1.0631	1.1475	1.1922	180	180	3511.3	2390.2	1343.3	594.6	594.6	411.8	411.8
X3LYP	3	−168.593950	1.0589	1.1532	1.1981	180	180	3499.7	2327.4	1294.3	567.2	567.2	257.1	257.1
X3LYP–D3(BJ)2B	3	−168.596079	1.0589	1.1532	1.1980	180	180	3500.4	2327.8	1294.7	567.1	567.1	256.0	256.0
X3LYP–D3(BJ)ATM	3	−168.596079	1.0589	1.1532	1.1980	180	180	3500.4	2327.8	1294.7	567.1	567.1	256.0	256.0
X3LYP–D3(0)2B	3	−168.594302	1.0591	1.1534	1.1982	180	180	3497.4	2326.0	1293.2	567.2	567.2	258.3	258.3
X3LYP–D3(0)ATM	3	−168.594302	1.0591	1.1534	1.1982	180	180	3497.4	2326.0	1293.2	567.2	567.2	258.3	258.3
XB1K	3	−164.944741	1.1000	1.1842	1.2316	180	180	3268.7	2196.5	1216.6	546.6	546.6	239.0	239.0

Table S4. DFT linear transition states for HCNO: barriers to linearity (cm^{-1}), geometric structures (Å, °), and harmonic vibrational frequencies (cm^{-1})

Functional	Group	Barrier	r_e(H–C)	r_e(C–N)	r_e(N–O)	θ_e(H–C–N)	θ_e(C–N–O)	$\omega_1(\sigma)$	$\omega_2(\sigma)$	$\omega_3(\sigma)$	$\omega_{4\,a}(\pi)$	$\omega_{4\,b}(\pi)$	$\omega_{5\,a}(\pi)$	$\omega_{5\,b}(\pi)$
B2PLYP	6	0.20	1.0572	1.1611	1.1994	180	180	3516.6	2282.9	1286.4	557.6	557.6	53.7 *i*	53.7 *i*
B2PLYP−D3(BJ)2B	6	0.24	1.0571	1.1611	1.1993	180	180	3516.9	2283.1	1286.4	557.2	557.1	57.6 *i*	58.0 *i*
B2PLYP−D3(BJ)ATM	6	0.24	1.0571	1.1611	1.1993	180	180	3516.9	2283.2	1286.5	558.1	557.9	52.6 *i*	52.9 *i*
B2PLYP−D3M(BJ)2B	6	0.29	1.0571	1.1610	1.1993	180	180	3517.0	2283.2	1286.7	557.5	557.5	59.7 *i*	59.7 *i*
B2PLYP−D3M(BJ)ATM	6	0.29	1.0571	1.1610	1.1993	180	180	3517.3	2283.3	1286.7	557.1	557.0	61.5 *i*	62.4 *i*
B2PLYP−D3M(0)2B	6	0.11	1.0573	1.1613	1.1995	180	180	3515.3	2281.4	1285.5	557.7	557.7	46.6 *i*	46.6 *i*
B2PLYP−D3M(0)ATM	6	0.11	1.0573	1.1613	1.1995	180	180	3515.0	2281.3	1285.5	557.4	557.3	48.9 *i*	50.2 *i*
B2PLYP−D3(0)2B	6	0.13	1.0573	1.1612	1.1995	180	180	3514.5	2281.7	1285.4	557.7	557.7	46.6 *i*	46.6 *i*
B2PLYP−D3(0)ATM	6	0.12	1.0573	1.1612	1.1995	180	180	3514.6	2281.5	1285.4	557.7	557.5	52.0 *i*	52.9 *i*
B2PLYP−NL	6	0.31	1.0574	1.1612	1.1992	180	180	3514.9	2283.3	1287.0	557.4	557.1	62.0 *i*	63.0 *i*
B86bPBE	1	26.88	1.0657	1.1682	1.2037	180	180	3438.8	2270.3	1275.0	544.9	544.9	187.6 *i*	187.6 *i*
B97−D	1	23.27	1.0632	1.1651	1.1990	180	180	3448.6	2274.7	1277.6	547.5	547.5	181.3 *i*	181.3 *i*
B97−D3(BJ)	1	32.18	1.0622	1.1645	1.1987	180	180	3460.6	2279.7	1280.8	547.3	547.3	197.0 *i*	197.0 *i*
B97−D3M(BJ)	1	34.85	1.0618	1.1641	1.1982	180	180	3463.8	2283.0	1283.2	546.8	546.8	201.1 *i*	201.1 *i*
B97^{GGA}−1	1	28.67	1.0610	1.1626	1.1902	180	180	3489.4	2319.5	1316.2	559.9	559.9	191.1 *i*	191.1 *i*
B97M−D3(BJ)	1	19.37	1.0521	1.1520	1.1923	180	180	3577.8	2360.6	1316.0	572.5	572.5	169.6 *i*	169.6 *i*
B97M−V	1	19.21	1.0527	1.1523	1.1923	180	180	3573.8	2359.8	1316.2	572.4	572.4	168.9 *i*	168.9 *i*
BLYP	1	27.30	1.0641	1.1683	1.2121	180	180	3426.7	2234.5	1236.4	532.5	532.5	189.4 *i*	189.4 *i*
BLYP−D3(BJ)2B	1	28.84	1.0640	1.1682	1.2120	180	180	3428.0	2235.3	1237.1	532.3	532.3	192.0 *i*	192.0 *i*
BLYP−D3(BJ)ATM	1	28.83	1.0640	1.1682	1.2120	180	180	3428.0	2235.4	1237.1	532.3	532.3	192.0 *i*	192.0 *i*
BLYP−D3M(BJ)2B	1	29.94	1.0638	1.1681	1.2119	180	180	3429.3	2236.2	1237.7	532.2	532.2	193.8 *i*	193.8 *i*
BLYP−D3M(BJ)ATM	1	29.93	1.0638	1.1681	1.2119	180	180	3429.3	2236.2	1237.7	532.2	532.2	193.8 *i*	193.8 *i*
BLYP−D3M(0)2B	1	24.32	1.0646	1.1688	1.2126	180	180	3422.7	2231.0	1234.2	532.9	532.9	184.0 *i*	184.0 *i*
BLYP−D3M(0)ATM	1	24.32	1.0646	1.1688	1.2126	180	180	3422.7	2231.0	1234.2	532.9	532.9	184.0 *i*	184.0 *i*
BLYP−D3(0)2B	1	26.22	1.0643	1.1685	1.2123	180	180	3423.4	2232.2	1234.6	532.6	532.6	187.2 *i*	187.2 *i*
BLYP−D3(0)ATM	1	26.21	1.0643	1.1685	1.2123	180	180	3423.4	2232.2	1234.6	532.6	532.6	187.2 *i*	187.2 *i*
BLYP−NL	1	29.17	1.0649	1.1684	1.2113	180	180	3422.2	2237.2	1240.4	531.4	531.4	191.9 *i*	191.9 *i*
BOP	1	39.06	1.0642	1.1690	1.2123	180	180	3432.4	2234.7	1237.2	533.2	533.2	207.7 *i*	207.7 *i*
BOP−D3(BJ)2B	1	43.07	1.0638	1.1688	1.2120	180	180	3435.6	2236.8	1238.7	532.8	532.8	212.9 *i*	212.9 *i*
BOP−D3(BJ)ATM	1	43.07	1.0638	1.1688	1.2120	180	180	3435.6	2236.8	1238.7	532.8	532.8	212.9 *i*	212.9 *i*
BOP−D3(0)2B	1	34.30	1.0647	1.1696	1.2128	180	180	3425.4	2230.0	1233.6	533.5	533.5	200.8 *i*	200.8 *i*

Table S4 (continued)

Functional	Group	Barrier	r_e(H–C)	r_e(C–N)	r_e(N–O)	θ_e(H–C–N)	θ_e(C–N–O)	$\omega_1(\sigma)$	$\omega_2(\sigma)$	$\omega_3(\sigma)$	$\omega_{4a}(\pi)$	$\omega_{4b}(\pi)$	$\omega_{5a}(\pi)$	$\omega_{5b}(\pi)$
BOP–D3(0)ATM	1	34.30	1.0647	1.1696	1.2128	180	180	3425.4	2230.0	1233.6	533.5	533.5	200.8 *i*	200.8 *i*
BP86	1	33.44	1.0668	1.1687	1.2052	180	180	3426.4	2259.6	1267.9	542.7	542.7	192.1 *i*	192.1 *i*
BP86–D3(BJ)2B	1	34.49	1.0667	1.1687	1.2051	180	180	3427.6	2261.7	1268.3	542.2	542.2	199.5 *i*	199.5 *i*
BP86–D3(BJ)ATM	1	34.48	1.0667	1.1687	1.2051	180	180	3427.6	2261.7	1268.3	542.2	542.2	199.5 *i*	199.5 *i*
BP86–D3M(BJ)2B	1	35.20	1.0667	1.1687	1.2051	180	180	3428.2	2261.9	1268.4	542.2	542.2	200.5 *i*	200.5 *i*
BP86–D3M(BJ)ATM	1	35.20	1.0667	1.1687	1.2051	180	180	3428.1	2261.9	1268.3	542.2	542.2	200.5 *i*	200.5 *i*
BP86–D3M(0)2B	1	32.03	1.0671	1.1690	1.2054	180	180	3423.7	2259.1	1266.2	542.5	542.5	195.6 *i*	195.6 *i*
BP86–D3M(0)ATM	1	32.03	1.0671	1.1690	1.2054	180	180	3423.7	2259.1	1266.2	542.5	542.5	195.6 *i*	195.6 *i*
BP86–D3(0)2B	1	32.39	1.0670	1.1690	1.2053	180	180	3423.9	2259.3	1266.3	542.4	542.4	196.1 *i*	196.1 *i*
BP86–D3(0)ATM	1	32.39	1.0670	1.1690	1.2053	180	180	3423.9	2259.3	1266.3	542.4	542.4	196.1 *i*	196.1 *i*
BP86–NL	1	35.47	1.0675	1.1689	1.2046	180	180	3424.4	2262.2	1270.6	541.7	541.7	198.2 *i*	198.2 *i*
BP86–VWN	1	30.72	1.0661	1.1681	1.2044	180	180	3431.7	2265.4	1270.7	543.3	543.3	194.0 *i*	194.0 *i*
core–DSD–BLYP	6	1.36	1.0563	1.1614	1.1965	180	180	3529.8	2287.1	1301.3	562.6	562.5	88.8 *i*	90.1 *i*
core–DSD–BLYP–D3(BJ)2B	6	1.44	1.0563	1.1614	1.1965	180	180	3530.2	2287.6	1301.6	562.8	562.8	91.6 *i*	91.6 *i*
core–DSD–BLYP–D3(BJ)ATM	6	1.44	1.0563	1.1614	1.1965	180	180	3530.2	2287.5	1301.5	562.7	562.7	90.7 *i*	90.7 *i*
DSD–BLYP	6	0.11	1.0550	1.1608	1.1932	180	180	3545.5	2300.6	1316.2	568.9	568.9	47.0 *i*	47.0 *i*
DSD–BLYP–D3(BJ)	6	0.23	1.0558	1.1606	1.1954	180	180	3536.1	2298.4	1305.9	565.4	565.3	56.2 *i*	57.0 *i*
DSD–BLYP–D3(BJ)2B	6	0.13	1.0550	1.1608	1.1932	180	180	3545.8	2300.8	1316.4	568.8	568.8	50.3 *i*	50.3 *i*
DSD–BLYP–D3(BJ)ATM	6	0.13	1.0550	1.1608	1.1932	180	180	3545.7	2300.8	1316.4	569.5	569.2	50.6 *i*	52.3 *i*
DSD–BLYP–D3(0)2B	6	0.07	1.0551	1.1609	1.1933	180	180	3543.8	2299.7	1315.5	568.9	568.9	43.4 *i*	43.4 *i*
DSD–BLYP–D3(0)ATM	6	0.07	1.0551	1.1609	1.1933	180	180	3544.2	2299.7	1315.6	568.9	568.9	42.6 *i*	42.6 *i*
DSD–BLYP–NL	6	0.22	1.0560	1.1607	1.1955	180	180	3534.4	2293.0	1306.2	564.6	564.5	55.9 *i*	57.4 *i*
DSD–PBEP86	6	2.50	1.0584	1.1623	1.1942	180	180	3531.6	2297.4	1309.3	563.2	563.0	60.4 *i*	60.5 *i*
DSD–PBEP86–D3(BJ)	6	4.32	1.0591	1.1625	1.1959	180	180	3517.7	2291.0	1306.1	563.8	563.8	116.1 *i*	116.1 *i*
DSD–PBEP86–NL	6	4.31	1.0593	1.1626	1.1959	180	180	3516.2	2290.2	1306.0	563.6	563.6	116.3 *i*	116.3 *i*
DSD–PBEPBE	6	3.72	1.0579	1.1620	1.1929	180	180	3530.7	2303.4	1318.8	568.7	568.5	114.2 *i*	114.5 *i*
DSD–PBEPBE–D3(BJ)	6	6.23	1.0588	1.1621	1.1950	180	180	3520.0	2295.6	1309.8	563.8	563.7	130.0 *i*	130.9 *i*
DSD–PBEPBE–NL	6	6.39	1.0590	1.1622	1.1949	180	180	3518.5	2295.5	1309.9	564.1	563.6	130.3 *i*	131.6 *i*
EDF1	1	27.77	1.0622	1.1653	1.2008	180	180	3463.1	2285.7	1282.8	547.6	547.6	190.3 *i*	190.3 *i*
FT97	1	49.17	1.0679	1.1721	1.2064	180	180	3430.7	2259.7	1268.7	542.5	542.5	217.8 *i*	217.8 *i*
GAM	1	1.90	1.0558	1.1594	1.1816	180	180	3532.9	2363.7	1352.1	573.9	573.9	95.9 *i*	95.9 *i*

Table S4 (continued)

Functional	Group	Barrier	r_e(H–C)	r_e(C–N)	r_e(N–O)	θ_e(H–C–N)	θ_e(C–N–O)	$\omega_1(\sigma)$	$\omega_2(\sigma)$	$\omega_3(\sigma)$	$\omega_{4\,a}(\pi)$	$\omega_{4\,b}(\pi)$	$\omega_{5\,a}(\pi)$	$\omega_{5\,b}(\pi)$
HCTH/120	1	23.02	1.0616	1.1633	1.1939	180	180	3474.3	2307.1	1303.6	554.8	554.8	181.1 *i*	181.1 *i*
HCTH/120–D3(BJ)2B	1	24.19	1.0615	1.1632	1.1938	180	180	3475.5	2307.8	1304.2	554.7	554.7	183.4 *i*	183.4 *i*
HCTH/120–D3(BJ)ATM	1	24.19	1.0615	1.1632	1.1938	180	180	3475.5	2307.8	1304.2	554.7	554.7	183.4 *i*	183.4 *i*
HCTH/120–D3(0)2B	1	22.52	1.0617	1.1634	1.1940	180	180	3472.6	2305.8	1302.6	554.8	554.8	180.0 *i*	180.0 *i*
HCTH/120–D3(0)ATM	1	22.52	1.0617	1.1634	1.1940	180	180	3472.6	2305.8	1302.6	554.8	554.8	180.0 *i*	180.0 *i*
HCTH/147	1	21.23	1.0616	1.1631	1.1933	180	180	3476.7	2310.4	1306.4	556.0	556.0	177.5 *i*	177.5 *i*
HCTH/407	1	12.00	1.0610	1.1621	1.1892	180	180	3490.0	2327.2	1322.7	561.8	561.8	153.6 *i*	153.6 *i*
HCTH/407–D3(BJ)2B	1	14.80	1.0605	1.1615	1.1885	180	180	3494.6	2332.1	1326.0	561.0	561.0	161.9 *i*	161.9 *i*
HCTH/407–D3(BJ)ATM	1	14.80	1.0605	1.1615	1.1885	180	180	3494.6	2332.1	1326.0	561.0	561.0	161.9 *i*	161.9 *i*
HCTH/407–D3(0)2B	1	10.53	1.0613	1.1624	1.1894	180	180	3485.2	2324.2	1320.2	562.0	562.0	148.4 *i*	148.4 *i*
HCTH/407–D3(0)ATM	1	10.53	1.0613	1.1624	1.1894	180	180	3485.2	2324.2	1320.2	562.0	562.0	148.4 *i*	148.4 *i*
HCTH/407+	1	27.74	1.0619	1.1640	1.1907	180	180	3487.3	2324.2	1320.1	559.9	559.9	190.0 *i*	190.0 *i*
HCTH/93	1	29.93	1.0618	1.1634	1.1928	180	180	3477.2	2313.2	1309.1	557.1	557.1	194.0 *i*	194.0 *i*
HCTH–p(1/4)	1	0.63	1.0603	1.1599	1.1885	180	180	3492.7	2341.6	1330.6	564.5	564.5	72.9 *i*	72.9 *i*
HCTH–p(7/6)	1	126.20	1.0842	1.1796	1.1996	180	180	3324.9	2244.2	1283.6	548.6	548.6	273.1 *i*	273.1 *i*
KT2	1	123.21	1.0531	1.1628	1.2043	180	180	3497.3	2277.3	1261.8	538.4	538.4	275.1 *i*	275.1 *i*
M06–L	2	11.98	1.0559	1.1573	1.1912	180	180	3536.8	2365.0	1335.8	584.3	584.3	146.9 *i*	146.9 *i*
M06–L–D3(0)2B	2	11.88	1.0560	1.1573	1.1912	180	180	3536.6	2365.0	1335.8	584.3	584.3	146.6 *i*	146.6 *i*
M06–L–D3(0)ATM	2	11.88	1.0560	1.1573	1.1912	180	180	3536.6	2365.0	1335.8	584.3	584.3	146.6 *i*	146.6 *i*
M11–L	2	129.17	1.0595	1.1440	1.1701	180	180	3514.3	2400.3	1368.1	583.4	583.4	285.4 *i*	285.4 *i*
M11–L–D3(BJ)2B	2	129.50	1.0594	1.1440	1.1701	180	180	3514.4	2400.4	1368.2	583.4	583.4	285.6 *i*	285.6 *i*
M11–L–D3(BJ)ATM	2	129.49	1.0594	1.1440	1.1701	180	180	3514.4	2400.4	1368.2	583.4	583.4	285.6 *i*	285.6 *i*
M11–L–D3(0)2B	2	127.73	1.0596	1.1441	1.1701	180	180	3512.5	2399.2	1367.2	583.4	583.4	284.4 *i*	284.4 *i*
M11–L–D3(0)ATM	2	127.73	1.0596	1.1441	1.1701	180	180	3512.5	2399.2	1367.2	583.4	583.4	284.4 *i*	284.4 *i*
MGGA_MS0	2	129.29	1.0557	1.1529	1.2047	180	180	3550.7	2316.4	1257.1	540.3	540.3	285.0 *i*	285.0 *i*
MGGA_MS1	2	141.38	1.0576	1.1558	1.2057	180	180	3534.0	2306.2	1257.2	539.9	539.9	291.4 *i*	291.4 *i*
MGGA_MS2	2	165.44	1.0576	1.1558	1.2053	180	180	3530.1	2306.8	1259.4	537.8	537.8	306.7 *i*	306.7 *i*
MGGA_MS2h	4	39.65	1.0565	1.1512	1.2006	180	180	3548.8	2337.7	1280.2	552.2	552.2	205.3 *i*	205.3 *i*
MN15–L	2	18.77	1.0665	1.1613	1.2008	180	180	3527.9	2360.9	1316.2	576.6	576.6	163.2 *i*	163.2 *i*
MN15–L–D3(0)2B	2	18.77	1.0665	1.1613	1.2008	180	180	3527.9	2360.9	1316.2	576.6	576.6	163.2 *i*	163.2 *i*
MN15–L–D3(0)ATM	2	18.77	1.0665	1.1613	1.2008	180	180	3527.9	2360.9	1316.2	576.6	576.6	163.2 *i*	163.2 *i*

Table S4 (continued)

Functional	Group	Barrier	r_e(H–C)	r_e(C–N)	r_e(N–O)	θ_e(H–C–N)	θ_e(C–N–O)	$\omega_1(\sigma)$	$\omega_2(\sigma)$	$\omega_3(\sigma)$	$\omega_{4\,a}(\pi)$	$\omega_{4\,b}(\pi)$	$\omega_{5\,a}(\pi)$	$\omega_{5\,b}(\pi)$
MOHLYP	1	95.13	1.0734	1.1774	1.2125	180	180	3396.7	2234.0	1251.7	534.9	534.9	257.5 *i*	257.5 *i*
MOHLYP2	1	189.06	1.0685	1.1771	1.2155	180	180	3425.5	2222.6	1234.2	530.7	530.7	310.3 *i*	310.3 *i*
MP2D	0	94.16	1.0560	1.1716	1.1903	180	180	3535.8	2270.3	1329.3	566.4	566.4	271.6 *i*	271.6 *i*
MP2	0	93.69	1.0560	1.1715	1.1902	180	180	3536.2	2271.0	1329.3	563.0	563.0	290.4 *i*	290.6 *i*
MPWLYP1M	3	2.53	1.0627	1.1645	1.2087	180	180	3444.2	2256.4	1250.1	540.4	540.4	102.2 *i*	102.2 *i*
MPWLYP1W	1	23.95	1.0634	1.1677	1.2117	180	180	3431.1	2236.2	1237.0	532.8	532.8	183.3 *i*	183.3 *i*
mPWPW	1	24.46	1.0650	1.1674	1.2031	180	180	3441.5	2272.1	1275.8	545.4	545.4	183.3 *i*	183.3 *i*
N12	1	0.58	1.0525	1.1543	1.1868	180	180	3501.2	2315.8	1304.5	557.0	557.0	71.7 *i*	71.7 *i*
N12–D3(BJ)2B	1	0.68	1.0525	1.1542	1.1868	180	180	3501.7	2316.0	1304.7	556.9	556.9	74.5 *i*	74.5 *i*
N12–D3(BJ)ATM	1	0.68	1.0525	1.1542	1.1868	180	180	3501.7	2316.0	1304.7	556.9	556.9	74.5 *i*	74.5 *i*
N12–D3(0)2B	1	0.33	1.0528	1.1545	1.1870	180	180	3497.3	2313.2	1302.4	557.1	557.1	62.0 *i*	62.0 *i*
N12–D3(0)ATM	1	0.33	1.0528	1.1545	1.1870	180	180	3497.3	2313.1	1302.4	557.1	557.1	62.0 *i*	62.0 *i*
oBLYP–D	1	20.25	1.0647	1.1681	1.2105	180	180	3421.9	2238.8	1241.9	534.6	534.6	170.2 *i*	170.2 *i*
oPBE–D	1	40.41	1.0669	1.1704	1.2071	180	180	3427.0	2254.7	1261.9	540.6	540.6	203.7 *i*	203.7 *i*
PBEOP	1	33.61	1.0651	1.1696	1.2120	180	180	3427.5	2235.8	1240.1	533.6	533.6	199.5 *i*	199.5 *i*
oPWLYP–D	1	21.02	1.0648	1.1682	1.2108	180	180	3420.3	2237.3	1240.7	534.1	534.1	171.9 *i*	171.9 *i*
oTPSS–D	1	119.15	1.0635	1.1630	1.2001	180	180	3461.1	2295.0	1279.3	550.8	550.8	297.7 *i*	297.7 *i*
PBE	1	25.43	1.0668	1.1686	1.2027	180	180	3434.1	2274.2	1280.4	546.3	546.3	184.4 *i*	184.4 *i*
PBE1W	1	26.58	1.0652	1.1682	1.2050	180	180	3439.3	2264.8	1268.9	542.9	542.9	187.4 *i*	187.4 *i*
PBE–D3(BJ)2B	1	26.12	1.0667	1.1686	1.2026	180	180	3434.7	2274.5	1280.6	546.2	546.2	185.7 *i*	185.7 *i*
PBE–D3(BJ)ATM	1	26.12	1.0667	1.1686	1.2026	180	180	3434.7	2274.5	1280.6	546.2	546.2	185.7 *i*	185.7 *i*
PBE–D3M(BJ)2B	1	26.14	1.0667	1.1686	1.2026	180	180	3434.8	2274.7	1280.8	546.2	546.2	185.8 *i*	185.8 *i*
PBE–D3M(BJ)ATM	1	26.14	1.0667	1.1686	1.2026	180	180	3434.8	2274.7	1280.8	546.2	546.2	185.7 *i*	185.7 *i*
PBE–D3M(0)2B	1	18.81	1.0653	1.1665	1.2018	180	180	3438.0	2279.1	1282.9	548.3	548.3	171.1 *i*	171.1 *i*
PBE–D3M(0)ATM	1	18.81	1.0653	1.1665	1.2018	180	180	3438.0	2279.1	1282.9	548.3	548.3	171.1 *i*	171.1 *i*
PBE–D3(0)2B	1	25.27	1.0669	1.1687	1.2027	180	180	3433.2	2273.4	1279.7	546.3	546.3	184.1 *i*	184.1 *i*
PBE–D3(0)ATM	1	25.27	1.0669	1.1687	1.2027	180	180	3433.2	2273.4	1279.7	546.3	546.3	184.1 *i*	184.1 *i*
PBELYP1W	1	24.19	1.0639	1.1683	1.2117	180	180	3430.7	2237.1	1238.8	533.2	533.2	183.7 *i*	183.7 *i*
PBE–NL	1	26.56	1.0671	1.1687	1.2024	180	180	3432.0	2274.9	1281.4	545.6	545.5	186.4 *i*	187.1 *i*
PBEsol	1	10.47	1.0704	1.1678	1.1965	180	180	3416.8	2296.2	1304.8	554.3	554.3	146.1 *i*	146.1 *i*
PBEsol–D3(BJ)2B	1	10.53	1.0704	1.1678	1.1965	180	180	3416.9	2296.2	1304.8	554.2	554.2	146.3 *i*	146.3 *i*

Table S4 (continued)

Functional	Group	Barrier	r_e(H–C)	r_e(C–N)	r_e(N–O)	θ_e(H–C–N)	θ_e(C–N–O)	$\omega_1(\sigma)$	$\omega_2(\sigma)$	$\omega_3(\sigma)$	$\omega_{4\,a}(\pi)$	$\omega_{4\,b}(\pi)$	$\omega_{5\,a}(\pi)$	$\omega_{5\,b}(\pi)$
PBEsol–D3(BJ)ATM	1	10.53	1.0704	1.1678	1.1965	180	180	3416.9	2296.2	1304.8	554.2	554.2	146.3 *i*	146.3 *i*
PBEsol–D3(0)2B	1	10.12	1.0705	1.1679	1.1966	180	180	3415.9	2295.5	1304.3	554.3	554.3	144.9 *i*	144.9 *i*
PBEsol–D3(0)ATM	1	10.12	1.0705	1.1679	1.1966	180	180	3415.9	2295.5	1304.3	554.3	554.3	144.8 *i*	144.8 *i*
PKZB	2	141.75	1.0701	1.1746	1.2096	180	180	3428.0	2253.0	1262.3	541.4	541.4	285.5 *i*	285.5 *i*
PKZB–D3(0)2B	2	199.43	1.0683	1.1727	1.2088	180	180	3429.5	2257.1	1258.5	538.1	538.1	308.2 *i*	308.2 *i*
PKZB–D3(0)ATM	2	199.42	1.0683	1.1727	1.2088	180	180	3429.5	2257.1	1258.5	538.1	538.1	308.2 *i*	308.2 *i*
PTPSS	6	12.15	1.0585	1.1615	1.1984	180	180	3509.7	2286.5	1291.0	559.2	559.1	152.7 *i*	152.8 *i*
PTPSS–D3(BJ)2B	6	12.34	1.0585	1.1615	1.1984	180	180	3509.9	2286.5	1291.1	558.8	558.6	156.4 *i*	156.5 *i*
PTPSS–D3(BJ)ATM	6	12.34	1.0585	1.1615	1.1984	180	180	3509.9	2286.7	1291.1	559.7	559.2	152.9 *i*	154.9 *i*
PTPSS–D3(0)2B	6	11.61	1.0586	1.1616	1.1985	180	180	3507.3	2283.7	1289.0	559.3	559.0	149.1 *i*	149.5 *i*
PTPSS–D3(0)ATM	6	11.61	1.0586	1.1616	1.1985	180	180	3508.0	2285.3	1290.1	558.9	558.7	152.9 *i*	152.9 *i*
PW86PBE	1	20.39	1.0648	1.1686	1.2066	180	180	3440.0	2259.3	1263.4	542.2	542.2	174.8 *i*	174.8 *i*
PW91	1	19.72	1.0651	1.1671	1.2023	180	180	3441.0	2274.9	1278.6	546.2	546.2	173.2 *i*	173.2 *i*
PW91–D3(BJ)2B	1	19.98	1.0651	1.1671	1.2023	180	180	3441.3	2275.0	1278.7	546.1	546.1	173.8 *i*	173.8 *i*
PW91–D3(BJ)ATM	1	19.98	1.0651	1.1671	1.2023	180	180	3441.3	2275.1	1278.7	546.1	546.1	173.8 *i*	173.8 *i*
revM06–L	2	1.05	1.0559	1.1480	1.1868	180	180	3542.0	2400.8	1346.4	592.3	592.3	79.2 *i*	79.2 *i*
revPBE	1	52.18	1.0671	1.1711	1.2069	180	180	3433.8	2258.7	1266.3	541.5	541.5	221.9 *i*	221.9 *i*
revPBE–D3(BJ)2B	1	55.31	1.0668	1.1709	1.2067	180	180	3435.8	2259.9	1267.2	541.2	541.2	225.1 *i*	225.1 *i*
revPBE–D3(BJ)ATM	1	55.31	1.0668	1.1709	1.2067	180	180	3435.8	2259.9	1267.2	541.2	541.2	225.1 *i*	225.1 *i*
revPBE–D3(0)2B	1	47.51	1.0675	1.1715	1.2072	180	180	3428.6	2255.2	1263.8	541.7	541.7	216.6 *i*	216.6 *i*
revPBE–D3(0)ATM	1	47.51	1.0675	1.1715	1.2072	180	180	3428.6	2255.2	1263.8	541.7	541.7	216.6 *i*	216.6 *i*
revPBE–NL	1	54.91	1.0679	1.1712	1.2060	180	180	3428.8	2261.8	1270.5	541.2	541.0	224.4 *i*	224.8 *i*
revSCAN	2	28.29	1.0602	1.1581	1.1925	180	180	3502.7	2342.6	1325.5	574.0	574.0	183.8 *i*	183.8 *i*
revTPSS	2	66.45	1.0628	1.1646	1.2062	180	180	3465.6	2276.1	1265.9	550.2	550.2	235.8 *i*	235.8 *i*
revTPSS–D3(BJ)2B	2	67.66	1.0628	1.1646	1.2062	180	180	3466.2	2276.5	1266.2	550.1	550.1	236.8 *i*	236.8 *i*
revTPSS–D3(BJ)ATM	2	67.71	1.0628	1.1646	1.2062	180	180	3466.3	2276.5	1266.2	550.1	550.1	236.9 *i*	236.9 *i*
revTPSS–D3(0)2B	2	64.69	1.0630	1.1648	1.2064	180	180	3463.3	2274.5	1264.7	550.3	550.3	234.0 *i*	234.0 *i*
revTPSS–D3(0)ATM	2	64.68	1.0630	1.1648	1.2064	180	180	3463.3	2274.5	1264.7	550.3	550.3	234.0 *i*	234.0 *i*
revTPSSh	3	1.37	1.0608	1.1586	1.2007	180	180	3494.3	2313.1	1289.2	563.8	563.8	85.2 *i*	85.2 *i*
revTPSSh–D3(BJ)2B	3	1.52	1.0608	1.1585	1.2007	180	180	3494.8	2313.4	1289.5	563.7	563.7	87.5 *i*	87.5 *i*
revTPSSh–D3(BJ)ATM	3	1.52	1.0608	1.1585	1.2007	180	180	3494.8	2313.4	1289.5	563.7	563.7	87.5 *i*	87.5 *i*

Table S4 (continued)

Functional	Group	Barrier	r_e(H–C)	r_e(C–N)	r_e(N–O)	θ_e(H–C–N)	θ_e(C–N–O)	$\omega_1(\sigma)$	$\omega_2(\sigma)$	$\omega_3(\sigma)$	$\omega_{4a}(\pi)$	$\omega_{4b}(\pi)$	$\omega_{5a}(\pi)$	$\omega_{5b}(\pi)$
revTPSSh–D3(0)2B	3	1.13	1.0610	1.1587	1.2008	180	180	3492.3	2311.7	1288.2	563.8	563.8	81.2 *i*	81.2 *i*
revTPSSh–D3(0)ATM	3	1.13	1.0610	1.1587	1.2008	180	180	3492.3	2311.7	1288.2	563.8	563.8	81.2 *i*	81.2 *i*
revTPSS–NL	2	68.68	1.0633	1.1648	1.2059	180	180	3462.7	2277.2	1267.6	549.1	549.0	237.5 *i*	238.1 *i*
RPBE	1	60.71	1.0671	1.1719	1.2082	180	180	3433.5	2253.6	1261.7	539.7	539.7	230.6 *i*	230.6 *i*
RPBE–D3(BJ)2B	1	68.39	1.0665	1.1713	1.2076	180	180	3439.1	2258.7	1265.2	538.9	538.9	237.7 *i*	237.7 *i*
RPBE–D3(BJ)ATM	1	68.39	1.0665	1.1713	1.2076	180	180	3439.1	2258.7	1265.2	538.9	538.9	237.7 *i*	237.7 *i*
RPBE–D3(0)2B	1	55.47	1.0676	1.1723	1.2087	180	180	3431.4	2251.4	1260.7	540.0	540.0	225.9 *i*	225.9 *i*
RPBE–D3(0)ATM	1	55.47	1.0676	1.1723	1.2087	180	180	3431.4	2251.4	1260.7	540.0	540.0	225.8 *i*	225.8 *i*
SCAN	2	51.79	1.0602	1.1571	1.1966	180	180	3493.1	2327.7	1301.1	561.2	561.2	202.8 *i*	202.8 *i*
SCAN–D3(BJ)2B	2	51.93	1.0601	1.1571	1.1965	180	180	3497.3	2323.7	1296.9	560.7	560.7	225.1 *i*	225.1 *i*
SCAN–D3(BJ)ATM	2	51.93	1.0601	1.1571	1.1965	180	180	3493.2	2327.7	1301.1	561.1	561.1	203.0 *i*	203.0 *i*
SCAN–D3(0)2B	2	51.84	1.0602	1.1571	1.1966	180	180	3493.3	2327.7	1301.1	561.1	561.1	202.9 *i*	202.9 *i*
SCAN–D3(0)ATM	2	51.84	1.0602	1.1571	1.1966	180	180	3493.3	2327.7	1301.2	561.1	561.1	202.9 *i*	202.9 *i*
SOGGA	1	10.82	1.0715	1.1684	1.1952	180	180	3414.5	2302.3	1311.9	556.5	556.5	146.9 *i*	146.9 *i*
SOGGA11	1	0.01	1.0649	1.1621	1.1873	180	180	3440.2	2305.4	1318.1	561.0	561.0	0 *i*	0 *i*
TH1	1	66.44	1.0652	1.1677	1.2014	180	180	3445.3	2266.7	1271.1	545.6	545.6	235.6 *i*	235.6 *i*
TH2	1	86.76	1.0648	1.1692	1.2079	180	180	3438.5	2239.3	1243.9	536.9	536.9	253.9 *i*	253.9 *i*
TH3	1	57.15	1.0646	1.1679	1.2055	180	180	3439.1	2257.1	1259.8	540.6	540.6	228.3 *i*	228.3 *i*
TH4	1	25.72	1.0579	1.1616	1.1971	180	180	3485.2	2296.0	1287.6	550.1	550.1	187.6 *i*	187.6 *i*
τ–HCTH	2	14.20	1.0603	1.1607	1.1907	180	180	3483.2	2314.7	1309.0	557.2	557.2	159.7 *i*	159.7 *i*
τ–HCTH–D3(BJ)2B	2	15.38	1.0601	1.1605	1.1905	180	180	3484.7	2316.2	1310.2	556.9	556.9	163.0 *i*	163.0 *i*
τ–HCTH–D3(BJ)ATM	2	15.38	1.0601	1.1605	1.1905	180	180	3484.7	2316.2	1310.2	556.9	556.9	163.0 *i*	163.0 *i*
τ–HCTH–D3(0)2B	2	12.28	1.0607	1.1610	1.1910	180	180	3478.8	2311.9	1306.9	557.4	557.4	153.9 *i*	153.9 *i*
τ–HCTH–D3(0)ATM	2	12.28	1.0607	1.1610	1.1910	180	180	3478.8	2311.9	1306.9	557.4	557.4	153.9 *i*	153.9 *i*
TH–FC	1	91.98	1.0650	1.1670	1.2039	180	180	3442.6	2256.9	1255.7	542.4	542.4	258.9 *i*	258.9 *i*
TH–FC+FO	1	91.98	1.0650	1.1670	1.2039	180	180	3442.6	2256.9	1255.7	542.4	542.4	258.9 *i*	258.9 *i*
TH–FCO	1	69.18	1.0650	1.1650	1.1994	180	180	3436.3	2265.4	1267.0	546.6	546.6	239.8 *i*	239.8 *i*
TPSS	2	41.54	1.0619	1.1642	1.2054	180	180	3468.6	2274.2	1265.3	550.0	550.0	209.2 *i*	209.2 *i*
TPSS–D3(BJ)2B	2	42.59	1.0618	1.1642	1.2054	180	180	3469.3	2274.6	1265.6	549.9	549.9	210.5 *i*	210.5 *i*
TPSS–D3(BJ)ATM	2	42.58	1.0618	1.1642	1.2054	180	180	3469.3	2274.6	1265.6	549.9	549.9	210.5 *i*	210.5 *i*
TPSS–D3(0)2B	2	41.09	1.0620	1.1644	1.2055	180	180	3466.9	2272.9	1264.2	550.0	550.0	208.5 *i*	208.5 *i*

Table S4 (continued)

Functional	Group	Barrier	r_e(H–C)	r_e(C–N)	r_e(N–O)	θ_e(H–C–N)	θ_e(C–N–O)	$\omega_1(\sigma)$	$\omega_2(\sigma)$	$\omega_3(\sigma)$	$\omega_{4\,a}(\pi)$	$\omega_{4\,b}(\pi)$	$\omega_{5\,a}(\pi)$	$\omega_{5\,b}(\pi)$
TPSS–D3(0)ATM	2	41.09	1.0620	1.1644	1.2055	180	180	3466.9	2272.9	1264.3	550.0	550.0	208.5 *i*	208.5 *i*
TPSSLYP1W	1	38.50	1.0587	1.1633	1.2149	180	180	3468.6	2239.6	1220.6	536.7	536.7	206.4 *i*	206.4 *i*
TPSS–NL	2	43.50	1.0624	1.1644	1.2050	180	180	3465.4	2275.6	1267.6	550.1	549.8	210.3 *i*	211.0 *i*
VSXC	2	34.41	1.0586	1.1631	1.1974	180	180	3515.4	2306.6	1294.6	561.3	561.3	201.1 *i*	201.1 *i*
VV10	1	19.02	1.0631	1.1674	1.2075	180	180	3445.5	2255.5	1256.8	539.7	539.3	171.4 *i*	172.5 *i*
XLYP	1	29.51	1.0633	1.1683	1.2135	180	180	3430.1	2229.7	1230.2	530.6	530.6	193.3 *i*	193.3 *i*
XLYP–D3(BJ)2B	1	31.28	1.0631	1.1681	1.2133	180	180	3431.7	2231.3	1231.5	530.2	530.2	196.2 *i*	196.2 *i*
XLYP–D3(BJ)ATM	1	31.28	1.0631	1.1681	1.2133	180	180	3431.7	2231.2	1231.5	530.2	530.2	196.2 *i*	196.2 *i*
XLYP–D3(0)2B	1	26.66	1.0637	1.1687	1.2139	180	180	3425.4	2226.6	1227.9	530.7	530.7	188.3 *i*	188.3 *i*
XLYP–D3(0)ATM	1	26.65	1.0637	1.1687	1.2139	180	180	3425.4	2226.6	1227.9	530.7	530.7	188.3 *i*	188.3 *i*
ZLP	1	40.38	1.0550	1.1589	1.1976	180	180	3497.9	2293.0	1278.4	550.5	550.5	212.9 *i*	212.9 *i*

Table S5. DFT predictions and percentiles (%–ile) for the HCN + O(^3P) → HCNO reaction energy [ΔE_e(rxn), kcal mol^{-1}], HCNO equilibrium bond distances, and HCNO geometric shifts [Δr_e(H–C), Δr_e(C–N), Å] relative to HCN

Functional	Group	ΔE_e(rxn)	%–ile ΔE_e(rxn)	%–ile r_e(H–C)	%–ile r_e(C–N)	%–ile r_e(N–O)	Δr_e(H–C)	%–ile Δr_e(H–C)	Δr_e(C–N)	%–ile Δr_e(C–N)
B2PLYP	6	–55.6	74.7	68.8	85.8	78.8	–0.0062	84.8	0.0099	43.0
B2PLYP–D3(BJ)2B	6	–55.9	70.6	66.9	85.6	78.5	–0.0062	82.3	0.0099	42.6
B2PLYP–D3(BJ)ATM	6	–55.9	70.4	67.1	85.4	77.9	–0.0062	83.1	0.0100	42.4
B2PLYP–D3M(BJ)2B	6	–56.1	69.1	66.3	85.0	77.3	–0.0062	83.7	0.0100	41.5
B2PLYP–D3M(BJ)ATM	6	–56.1	68.9	65.8	85.2	77.5	–0.0062	83.3	0.0100	41.8
B2PLYP–D3M(0)2B	6	–55.8	72.2	70.2	84.4	81.0	–0.0063	74.1	0.0099	43.8
B2PLYP–D3M(0)ATM	6	–55.8	72.0	70.4	84.2	80.8	–0.0063	74.3	0.0099	43.2
B2PLYP–D3(0)2B	6	–55.7	74.3	69.4	84.8	80.0	–0.0062	77.2	0.0099	44.5
B2PLYP–D3(0)ATM	6	–55.7	74.1	69.6	84.6	79.8	–0.0062	77.5	0.0099	44.1
B2PLYP–NL	6	–56.7	61.8	74.0	83.8	76.9	–0.0061	90.2	0.0100	41.3
B86bPBE	1	–72.3	11.1	12.7	22.3	98.5	–0.0058	91.2	0.0155	18.6
B97–D	1	–62.7	29.9	24.8	38.8	68.8	–0.0062	83.5	0.0148	23.0
B97–D3(BJ)	1	–63.8	26.9	30.6	38.5	66.3	–0.0057	82.0	0.0153	20.5
B97–D3M(BJ)	1	–64.9	25.3	31.9	39.6	65.6	–0.0056	71.2	0.0153	20.7
B97^{GGA}–1	1	–65.8	21.9	36.0	50.6	17.1	–0.0064	66.0	0.0142	26.5
B97M–D3(BJ)	1	–56.7	62.6	34.6	86.9	30.4	–0.0055	66.4	0.0117	35.9
B97M–V	1	–57.5	54.3	37.3	89.8	30.2	–0.0054	61.0	0.0118	35.5
BLYP	1	–64.8	25.5	19.6	21.9	54.4	–0.0052	42.0	0.0162	15.4
BLYP–D3(BJ)2B	1	–65.7	22.5	20.2	20.2	55.6	–0.0052	41.3	0.0163	11.5
BLYP–D3(BJ)ATM	1	–65.7	22.3	20.4	20.4	55.8	–0.0052	41.5	0.0163	11.7
BLYP–D3M(BJ)2B	1	–66.0	20.7	20.8	21.0	57.3	–0.0051	39.0	0.0163	11.1
BLYP–D3M(BJ)ATM	1	–66.0	20.5	20.6	21.3	57.1	–0.0051	38.8	0.0163	11.3
BLYP–D3M(0)2B	1	–65.3	24.0	16.5	16.9	51.0	–0.0054	57.2	0.0164	9.6
BLYP–D3M(0)ATM	1	–65.3	23.8	16.3	17.1	50.8	–0.0054	57.0	0.0164	9.8
BLYP–D3(0)2B	1	–65.0	24.8	18.5	19.2	53.3	–0.0054	58.2	0.0163	12.7
BLYP–D3(0)ATM	1	–65.0	24.6	18.3	19.0	53.1	–0.0054	58.0	0.0163	12.5
BLYP–NL	1	–68.7	14.4	14.4	17.7	62.3	–0.0050	30.3	0.0164	10.0
BOP	1	–62.8	29.6	16.0	14.2	56.0	–0.0048	22.8	0.0169	7.3
BOP–D3(BJ)2B	1	–64.5	26.5	17.5	13.8	59.8	–0.0048	22.1	0.0170	6.1
BOP–D3(BJ)ATM	1	–64.5	26.3	17.3	14.0	59.6	–0.0048	21.9	0.0170	6.3
BOP–D3(0)2B	1	–63.2	28.8	14.2	12.9	52.9	–0.0053	46.3	0.0169	7.5

Table S5 (continued)

Functional	Group	ΔE_e(rxn)	%–ile ΔE_e(rxn)	%–ile r_e(H–C)	%–ile r_e(C–N)	%–ile r_e(N–O)	Δr_e(H–C)	%–ile Δr_e(H–C)	Δr_e(C–N)	%–ile Δr_e(C–N)
BOP–D3(0)ATM	1	−63.2	28.6	14.0	13.1	52.7	−0.0053	46.1	0.0169	7.7
BP86	1	−72.5	10.9	8.1	16.7	95.4	−0.0054	58.9	0.0162	15.7
BP86–D3(BJ)2B	1	−73.1	9.8	8.5	16.0	95.8	−0.0054	61.4	0.0162	14.0
BP86–D3(BJ)ATM	1	−73.1	9.6	8.3	16.3	95.6	−0.0054	61.2	0.0162	14.2
BP86–D3M(BJ)2B	1	−73.2	9.4	9.0	15.6	96.3	−0.0054	61.8	0.0163	13.6
BP86–D3M(BJ)ATM	1	−73.2	9.2	8.8	15.8	96.0	−0.0054	61.6	0.0163	13.8
BP86–D3M(0)2B	1	−72.7	10.2	6.7	15.2	94.2	−0.0057	77.9	0.0162	14.8
BP86–D3M(0)ATM	1	−72.7	10.0	6.5	15.4	94.0	−0.0057	77.7	0.0162	15.0
BP86–D3(0)2B	1	−72.7	10.6	7.5	14.8	94.6	−0.0057	79.3	0.0162	14.4
BP86–D3(0)ATM	1	−72.7	10.4	7.3	15.0	94.4	−0.0057	79.1	0.0162	14.6
BP86–NL	1	−75.8	6.3	5.6	14.4	99.6	−0.0053	48.6	0.0163	11.9
BP86–VWN	1	−70.9	12.3	12.3	19.8	99.4	−0.0055	64.7	0.0160	17.5
core–DSD–BLYP	6	−54.2	82.0	58.8	82.7	65.4	−0.0058	87.5	0.0102	40.1
core–DSD–BLYP–D3(BJ)2B	6	−54.5	80.8	57.9	82.9	65.2	−0.0058	89.8	0.0102	39.9
core–DSD–BLYP–D3(BJ)ATM	6	−54.5	80.6	58.1	82.5	65.0	−0.0058	89.4	0.0102	39.7
DSD–BLYP	6	−56.7	61.6	41.3	89.0	43.5	−0.0061	90.4	0.0095	47.0
DSD–BLYP–D3(BJ)	6	−54.8	79.1	50.0	90.0	60.0	−0.0060	93.9	0.0093	48.0
DSD–BLYP–D3(BJ)2B	6	−57.0	58.5	40.8	89.4	42.9	−0.0061	87.7	0.0095	46.8
DSD–BLYP–D3(BJ)ATM	6	−57.0	58.2	41.0	89.2	43.1	−0.0061	89.1	0.0095	46.6
DSD–BLYP–D3(0)2B	6	−56.8	60.5	41.7	87.3	45.0	−0.0061	86.0	0.0095	46.3
DSD–BLYP–D3(0)ATM	6	−56.8	60.3	41.9	87.1	44.8	−0.0061	86.4	0.0095	46.1
DSD–BLYP–NL	6	−54.8	78.7	51.0	88.1	60.2	−0.0060	96.2	0.0094	47.8
DSD–PBEP86	6	−56.6	64.5	99.4	72.3	46.9	−0.0059	94.2	0.0102	39.5
DSD–PBEP86–D3(BJ)	6	−55.4	76.0	84.0	67.9	57.9	−0.0057	80.0	0.0106	37.6
DSD–PBEP86–NL	6	−55.5	75.4	80.0	67.3	58.1	−0.0057	78.5	0.0106	37.4
DSD–PBEPBE	6	−57.3	56.4	93.1	71.5	36.3	−0.0058	87.3	0.0105	37.8
DSD–PBEPBE–D3(BJ)	6	−55.9	69.9	89.6	67.5	50.0	−0.0056	72.9	0.0109	37.2
DSD–PBEPBE–NL	6	−56.4	65.8	83.8	65.8	49.8	−0.0056	69.3	0.0109	37.0
EDF1	1	−67.3	17.7	32.1	38.1	77.7	−0.0058	92.1	0.0152	21.5
FT97	1	−61.9	31.9	3.8	6.5	90.6	−0.0057	76.8	0.0165	8.8
GAM	1	−68.5	14.6	52.3	92.1	2.3	−0.0082	2.7	0.0104	38.4

Table S5 (continued)

Functional	Group	ΔE_e(rxn)	%−ile ΔE_e(rxn)	%−ile r_e(H−C)	%−ile r_e(C−N)	%−ile r_e(N−O)	Δr_e(H−C)	%−ile Δr_e(H−C)	Δr_e(C−N)	%−ile Δr_e(C−N)
HCTH/120	1	−67.7	17.1	34.0	50.2	38.1	−0.0064	64.5	0.0142	26.3
HCTH/120−D3(BJ)2B	1	−68.4	15.7	34.4	49.6	36.5	−0.0064	68.1	0.0144	23.6
HCTH/120−D3(BJ)ATM	1	−68.4	15.4	34.2	49.4	36.7	−0.0064	68.3	0.0144	23.4
HCTH/120−D3(0)2B	1	−67.8	16.5	33.5	49.8	39.2	−0.0066	52.2	0.0142	24.8
HCTH/120−D3(0)ATM	1	−67.8	16.3	33.3	50.0	39.4	−0.0066	52.4	0.0142	25.1
HCTH/147	1	−68.1	16.1	35.0	51.5	34.4	−0.0065	58.5	0.0140	26.9
HCTH/407	1	−68.2	15.9	43.8	62.1	14.2	−0.0073	18.0	0.0128	32.2
HCTH/407−D3(BJ)2B	1	−70.3	13.4	46.9	65.4	11.3	−0.0073	19.2	0.0127	33.0
HCTH/407−D3(BJ)ATM	1	−70.3	13.2	46.7	65.6	11.5	−0.0073	19.4	0.0127	33.2
HCTH/407−D3(0)2B	1	−68.4	15.2	42.3	60.6	16.7	−0.0077	12.9	0.0128	32.4
HCTH/407−D3(0)ATM	1	−68.4	15.0	42.1	60.8	16.9	−0.0077	13.2	0.0128	32.6
HCTH/407+	1	−67.7	16.9	32.7	42.9	20.0	−0.0069	28.4	0.0138	27.1
HCTH/93	1	−66.5	20.0	32.9	45.2	32.3	−0.0063	71.6	0.0146	23.2
HCTH−p(1/4)	1	−74.5	9.0	66.5	91.9	15.2	−0.0077	11.7	0.0117	35.7
HCTH−p(7/6)	1	−41.6	42.0	0.2	2.5	65.8	−0.0061	87.1	0.0187	1.5
KT2	1	−63.3	28.4	81.5	18.1	86.0	−0.0028	1.0	0.0190	1.0
M06−L	2	−61.8	32.6	75.4	91.3	24.2	−0.0077	11.1	0.0116	36.1
M06−L−D3(0)2B	2	−61.8	32.4	75.6	91.5	24.4	−0.0077	11.3	0.0116	36.3
M06−L−D3(0)ATM	2	−61.8	32.2	75.8	91.7	24.6	−0.0077	11.5	0.0116	36.5
M11−L	2	−56.5	65.6	28.1	71.7	0.6	−0.0070	27.8	0.0142	25.7
M11−L−D3(BJ)2B	2	−56.7	62.2	28.5	72.1	0.2	−0.0070	26.1	0.0142	25.3
M11−L−D3(BJ)ATM	2	−56.7	62.0	28.3	71.9	0.4	−0.0070	26.3	0.0142	25.5
M11−L−D3(0)2B	2	−56.6	63.3	27.7	72.9	0.8	−0.0071	22.3	0.0142	25.9
M11−L−D3(0)ATM	2	−56.6	63.0	27.5	72.7	1.0	−0.0071	22.5	0.0142	26.1
MGGA_MS0	2	−53.6	86.6	61.5	70.6	76.7	−0.0016	0.4	0.0182	2.3
MGGA_MS1	2	−53.7	85.6	37.9	50.4	84.6	−0.0016	0.2	0.0189	1.3
MGGA_MS2	2	−55.9	70.1	34.8	45.8	80.6	−0.0013	0.0	0.0196	0.8
MGGA_MS2h	4	−51.8	96.2	92.3	93.1	68.3	−0.0039	4.6	0.0129	31.3
MN12−L	2	−52.1	94.2	57.3	29.2	16.3	−0.0080	5.6	0.0062	95.8
MN12−L−D3(BJ)2B	2	−52.1	93.3	56.9	29.6	15.8	−0.0080	5.2	0.0062	95.4
MN12−L−D3(BJ)ATM	2	−52.1	93.1	57.1	29.4	16.0	−0.0080	5.4	0.0062	95.6

Table S5 (continued)

Functional	Group	ΔE_e(rxn)	%–ile ΔE_e(rxn)	%–ile r_e(H–C)	%–ile r_e(C–N)	%–ile r_e(N–O)	Δr_e(H–C)	%–ile Δr_e(H–C)	Δr_e(C–N)	%–ile Δr_e(C–N)
MN12–L–D3(0)2B	2	−52.1	92.1	58.3	31.0	17.3	−0.0081	4.2	0.0062	94.8
MN12–L–D3(0)ATM	2	−52.1	91.9	58.5	30.8	17.5	−0.0081	4.4	0.0062	95.0
MN15–L	2	−56.3	66.4	11.7	54.4	72.7	−0.0078	9.6	0.0096	45.3
MN15–L–D3(0)2B	2	−56.3	66.2	11.5	54.2	72.9	−0.0078	9.8	0.0096	45.1
MN15–L–D3(0)ATM	2	−56.3	66.0	11.3	54.6	73.1	−0.0078	10.0	0.0096	45.5
MOHLYP	1	−62.0	31.7	0.4	2.7	62.5	−0.0047	20.7	0.0186	1.7
MOHLYP2	1	−37.2	24.4	0.8	2.3	50.6	−0.0031	1.9	0.0214	0.0
MP2D	0	−174.4	0.2	96.5	5.2	13.1	−0.0036	2.5	0.0184	2.1
MP2	0	−175.7	0.0	96.7	5.4	12.7	−0.0038	4.0	0.0182	2.5
MPWLYP1M	3	−63.6	27.1	35.8	56.5	66.5	−0.0064	66.2	0.0126	33.4
MPWLYP1W	1	−65.9	21.3	24.0	24.6	56.3	−0.0052	45.1	0.0161	17.1
mPWPW	1	−71.9	11.5	14.6	25.6	93.8	−0.0057	82.9	0.0156	18.2
N12	1	−65.3	23.6	26.7	80.6	7.5	−0.0065	59.9	0.0130	30.1
N12–D3(BJ)2B	1	−65.9	21.7	25.6	81.3	6.9	−0.0065	57.4	0.0130	29.4
N12–D3(BJ)ATM	1	−65.9	21.5	25.8	81.0	7.1	−0.0065	57.6	0.0130	29.6
N12–D3(0)2B	1	−65.6	23.0	29.0	81.7	9.0	−0.0067	42.6	0.0130	30.3
N12–D3(0)ATM	1	−65.6	22.8	29.2	81.5	9.2	−0.0067	42.8	0.0130	30.5
oBLYP–D	1	−67.2	18.4	19.2	25.4	64.8	−0.0054	56.2	0.0157	18.0
oPBE–D	1	−67.8	16.7	6.3	12.1	83.1	−0.0053	48.4	0.0164	10.9
PBEOP	1	−66.6	19.8	12.9	13.3	56.9	−0.0051	37.6	0.0165	8.6
oPWLYP–D	1	−67.1	18.8	17.7	24.8	63.3	−0.0053	52.8	0.0158	17.7
oTPSS–D	1	−60.6	40.9	13.1	25.8	66.7	−0.0042	11.9	0.0178	3.1
PBE	1	−74.8	8.1	9.8	20.0	91.9	−0.0060	98.3	0.0154	19.4
PBE1W	1	−71.3	12.1	13.8	22.5	94.8	−0.0057	83.9	0.0156	18.4
PBE–D3(BJ)2B	1	−75.2	7.5	10.4	20.6	91.5	−0.0060	94.4	0.0154	19.6
PBE–D3(BJ)ATM	1	−75.2	7.3	10.2	20.8	91.7	−0.0060	94.6	0.0154	19.8
PBE–D3M(BJ)2B	1	−75.4	7.1	10.0	21.5	90.8	−0.0060	95.6	0.0154	20.0
PBE–D3M(BJ)ATM	1	−75.4	6.9	10.6	21.7	91.0	−0.0060	95.0	0.0154	20.3
PBE–D3M(0)2B	1	−75.6	6.7	15.0	35.4	87.5	−0.0062	78.7	0.0149	22.5
PBE–D3M(0)ATM	1	−75.6	6.5	14.8	35.6	87.7	−0.0062	78.9	0.0149	22.8
PBE–D3(0)2B	1	−74.9	7.9	9.6	19.6	92.5	−0.0061	89.6	0.0154	19.2

Table S5 (continued)

Functional	Group	ΔE_e(rxn)	%−ile ΔE_e(rxn)	%−ile r_e(H−C)	%−ile r_e(C−N)	%−ile r_e(N−O)	Δr_e(H−C)	%−ile Δr_e(H−C)	Δr_e(C−N)	%−ile Δr_e(C−N)
PBE−D3(0)ATM	1	−74.9	7.7	9.4	19.4	92.7	−0.0061	90.0	0.0154	19.0
PBELYP1W	1	−67.1	18.6	21.7	22.1	57.5	−0.0053	47.8	0.0160	17.3
PBE−NL	1	−76.5	5.8	7.9	18.8	89.4	−0.0059	93.7	0.0154	18.8
PBEsol	1	−86.5	5.4	2.5	31.7	62.1	−0.0069	34.2	0.0143	24.2
PBEsol−D3(BJ)2B	1	−86.6	4.8	2.9	31.3	61.7	−0.0069	33.8	0.0143	23.8
PBEsol−D3(BJ)ATM	1	−86.6	4.6	2.7	31.5	61.9	−0.0069	34.0	0.0143	24.0
PBEsol−D3(0)2B	1	−86.6	5.2	2.3	30.4	62.7	−0.0069	29.4	0.0143	24.4
PBEsol−D3(0)ATM	1	−86.6	5.0	2.1	30.6	62.9	−0.0069	29.6	0.0143	24.6
PKZB	2	−61.5	35.7	0.6	3.3	79.6	−0.0035	2.3	0.0204	0.6
PKZB−D3(0)2B	2	−63.4	28.0	1.3	2.9	90.4	−0.0027	0.8	0.0211	0.2
PKZB−D3(0)ATM	2	−63.4	27.8	1.0	3.1	90.2	−0.0027	0.6	0.0211	0.4
PTPSS	6	−55.0	77.9	85.2	63.1	67.5	−0.0053	49.1	0.0125	34.0
PTPSS−D3(BJ)2B	6	−55.2	76.6	85.4	62.7	67.1	−0.0053	50.5	0.0125	33.6
PTPSS−D3(BJ)ATM	6	−55.2	76.4	85.6	62.9	67.3	−0.0053	50.7	0.0125	33.8
PTPSS−D3(0)2B	6	−55.1	77.5	83.5	62.3	67.9	−0.0054	56.8	0.0125	34.2
PTPSS−D3(0)ATM	6	−55.1	77.2	83.3	62.5	68.1	−0.0054	56.6	0.0125	34.4
PW86PBE	1	−69.0	14.0	16.7	22.7	82.1	−0.0059	97.7	0.0151	22.1
PW91	1	−74.6	8.8	15.2	28.3	90.0	−0.0060	98.5	0.0152	21.3
PW91−D3(BJ)2B	1	−74.8	8.6	15.6	27.3	89.6	−0.0060	97.9	0.0152	20.9
PW91−D3(BJ)ATM	1	−74.8	8.4	15.4	27.5	89.8	−0.0060	98.1	0.0152	21.1
revM06−L	2	−58.8	49.7	52.9	41.7	6.7	−0.0080	6.1	0.0096	45.7
revPBE	1	−66.4	20.3	5.0	9.6	87.3	−0.0052	43.2	0.0169	7.9
revPBE−D3(BJ)2B	1	−67.5	17.5	6.0	9.2	88.8	−0.0052	43.6	0.0169	6.9
revPBE−D3(BJ)ATM	1	−67.5	17.3	5.8	9.4	88.5	−0.0052	43.4	0.0169	7.1
revPBE−D3(0)2B	1	−66.7	19.6	4.8	9.0	83.8	−0.0056	70.8	0.0168	8.4
revPBE−D3(0)ATM	1	−66.7	19.4	4.6	8.8	84.0	−0.0056	69.7	0.0168	8.1
revPBE−NL	1	−70.8	12.5	3.3	8.5	92.9	−0.0051	34.4	0.0170	6.5
revSCAN	2	−61.6	34.7	48.1	83.5	32.1	−0.0071	23.6	0.0138	27.3
revTPSS	2	−60.1	43.8	19.4	24.4	96.5	−0.0041	8.8	0.0174	4.4
revTPSS−D3(BJ)2B	2	−60.5	42.2	19.8	24.2	97.1	−0.0041	9.2	0.0175	3.8
revTPSS−D3(BJ)ATM	2	−60.6	41.8	20.0	24.0	97.3	−0.0041	9.4	0.0175	3.5

Table S5 (continued)

Functional	Group	ΔE_e(rxn)	%−ile ΔE_e(rxn)	%−ile r_e(H−C)	%−ile r_e(C−N)	%−ile r_e(N−O)	Δr_e(H−C)	%−ile Δr_e(H−C)	Δr_e(C−N)	%−ile Δr_e(C−N)
revTPSS−D3(0)2B	2	−60.2	42.8	19.0	23.3	95.2	−0.0042	12.3	0.0174	4.0
revTPSS−D3(0)ATM	2	−60.2	42.6	18.8	23.5	95.0	−0.0042	12.1	0.0174	4.2
revTPSSh	3	−55.0	78.1	54.8	94.2	86.3	−0.0066	52.6	0.0099	42.8
revTPSSh−D3(BJ)2B	3	−55.4	75.8	56.0	93.8	85.6	−0.0066	50.1	0.0100	42.0
revTPSSh−D3(BJ)ATM	3	−55.4	75.6	55.8	94.0	85.8	−0.0066	50.3	0.0100	42.2
revTPSSh−D3(0)2B	3	−55.1	77.0	53.1	93.3	86.9	−0.0067	42.4	0.0099	43.6
revTPSSh−D3(0)ATM	3	−55.1	76.8	53.5	93.5	87.1	−0.0067	42.2	0.0099	44.3
revTPSS−NL	2	−62.3	31.3	15.8	23.1	99.2	−0.0040	6.9	0.0175	3.3
RPBE	1	−65.0	24.2	4.4	6.3	79.2	−0.0050	31.5	0.0172	4.8
RPBE−D3(BJ)2B	1	−67.3	18.2	5.4	6.7	85.0	−0.0049	25.3	0.0172	5.0
RPBE−D3(BJ)ATM	1	−67.3	18.0	5.2	6.9	84.8	−0.0049	25.1	0.0172	5.2
RPBE−D3(0)2B	1	−65.5	23.4	4.2	5.6	74.2	−0.0053	49.5	0.0172	5.6
RPBE−D3(0)ATM	1	−65.5	23.2	4.0	5.8	74.0	−0.0053	49.3	0.0172	5.8
SCAN	2	−60.7	40.7	39.8	79.8	52.3	−0.0044	15.4	0.0163	13.4
SCAN−D3(BJ)2B	2	−60.8	39.2	40.2	79.0	51.5	−0.0044	15.9	0.0163	12.1
SCAN−D3(BJ)ATM	2	−60.8	39.0	40.0	79.2	51.7	−0.0044	15.7	0.0163	12.3
SCAN−D3(0)2B	2	−60.7	39.9	39.6	79.4	51.9	−0.0044	16.3	0.0163	12.9
SCAN−D3(0)ATM	2	−60.7	39.7	39.4	79.6	52.1	−0.0044	16.1	0.0163	13.2
SOGGA	1	−88.2	4.0	1.7	26.3	52.5	−0.0071	24.8	0.0141	26.7
SOGGA11	1	−76.1	6.1	29.4	83.3	11.7	−0.0061	92.3	0.0161	15.9
TH1	1	−68.5	14.8	11.0	12.3	73.3	−0.0049	25.5	0.0172	5.4
TH2	1	−57.3	55.7	9.2	8.3	86.7	−0.0039	6.3	0.0186	1.9
TH3	1	−61.3	36.7	12.5	13.5	97.5	−0.0048	24.2	0.0170	6.7
TH4	1	−62.9	29.4	78.8	55.4	61.0	−0.0056	71.0	0.0150	22.3
τ−HCTH	2	−65.8	22.1	49.8	70.8	21.0	−0.0066	51.4	0.0132	28.4
τ−HCTH−D3(BJ)2B	2	−66.9	19.2	50.8	71.0	20.2	−0.0066	49.7	0.0132	28.6
τ−HCTH−D3(BJ)ATM	2	−66.9	19.0	50.6	71.3	20.4	−0.0066	49.9	0.0132	28.8
τ−HCTH−D3(0)2B	2	−66.0	21.1	46.3	70.2	23.3	−0.0069	31.1	0.0132	29.0
τ−HCTH−D3(0)ATM	2	−66.0	20.9	46.0	70.4	23.5	−0.0069	31.3	0.0132	29.2
TH−FC	1	−24.2	5.6	7.1	11.9	89.0	−0.0046	19.0	0.0180	2.9
TH−FC+FO	1	−50.8	97.5	6.9	11.7	89.2	−0.0046	18.8	0.0180	2.7

Table S5 (continued)

Functional	Group	ΔE_e(rxn)	%−ile ΔE_e(rxn)	%−ile r_e(H−C)	%−ile r_e(C−N)	%−ile r_e(N−O)	Δr_e(H−C)	%−ile Δr_e(H−C)	Δr_e(C−N)	%−ile Δr_e(C−N)
TH−FCO	1	−56.1	68.5	10.8	22.9	66.0	−0.0050	30.9	0.0173	4.6
TH−FL	1	−88.1	4.4	11.9	96.9	4.2	−0.0078	10.9	0.0130	29.9
TPSS	2	−61.3	37.0	30.0	36.3	97.7	−0.0049	25.9	0.0161	16.9
TPSS−D3(BJ)2B	2	−61.8	33.4	30.4	35.0	99.0	−0.0049	27.6	0.0161	16.1
TPSS−D3(BJ)ATM	2	−61.8	33.2	30.2	35.2	98.8	−0.0049	27.3	0.0161	16.3
TPSS−D3(0)2B	2	−61.4	36.5	29.8	33.5	96.9	−0.0051	36.5	0.0161	16.5
TPSS−D3(0)ATM	2	−61.4	36.3	29.6	33.8	96.7	−0.0051	36.3	0.0161	16.7
TPSSh−D3(BJ)2B	3	−56.6	64.1	79.0	97.1	81.7	−0.0071	23.4	0.0093	48.2
TPSSLYP1W	1	−54.6	79.7	53.3	39.8	41.0	−0.0043	14.0	0.0164	10.2
TPSS−NL	2	−63.9	26.7	25.0	32.3	99.8	−0.0048	23.8	0.0162	15.2
VSXC	2	−58.6	51.1	59.0	44.2	60.8	−0.0060	99.8	0.0151	21.9
VV10	1	−68.8	14.2	27.9	26.0	75.8	−0.0056	76.6	0.0152	21.7
XLYP	1	−63.3	28.2	23.1	17.9	45.4	−0.0050	29.0	0.0164	9.4
XLYP−D3(BJ)2B	1	−64.5	26.1	23.8	18.3	47.3	−0.0050	30.1	0.0164	9.0
XLYP−D3(BJ)ATM	1	−64.5	25.9	23.5	18.5	47.1	−0.0050	29.9	0.0164	9.2
XLYP−D3(0)2B	1	−63.6	27.6	22.1	17.5	41.9	−0.0053	51.1	0.0164	10.6
XLYP−D3(0)ATM	1	−63.6	27.3	21.9	17.3	41.7	−0.0053	50.9	0.0164	10.4
ZLP	1	nc[a]	nc	74.2	70.0	61.3	nc	nc	nc	nc
B1LYP	3	−50.3	95.4	86.0	58.5	74.4	−0.0062	80.2	0.0084	67.6
B1LYP−D3(BJ)2B	3	−51.0	98.7	84.2	57.9	73.5	−0.0063	75.8	0.0084	67.8
B1LYP−D3(BJ)ATM	3	−51.0	99.0	84.2	57.9	73.5	−0.0063	75.8	0.0084	67.8
B1LYP−D3(0)2B	3	−50.5	96.7	90.2	60.0	76.0	−0.0063	73.1	0.0086	64.9
B1LYP−D3(0)ATM	3	−50.5	96.9	90.2	60.0	76.0	−0.0063	73.1	0.0086	64.9
B1PW91	3	−55.5	74.9	91.0	58.5	31.9	−0.0067	45.3	0.0082	74.7
B1WC	3	−71.6	11.7	33.8	86.7	20.8	−0.0071	23.0	0.0098	44.9
B2GP−PLYP	6	−53.5	86.8	46.5	97.9	64.0	−0.0062	85.8	0.0087	58.7
B2GP−PLYP−D3(BJ)2B	6	−53.7	85.4	45.6	97.3	63.5	−0.0062	84.1	0.0087	59.1
B2GP−PLYP−D3(BJ)ATM	6	−53.7	85.2	45.8	97.3	63.5	−0.0062	85.4	0.0087	59.1
B2GP−PLYP−D3(0)2B	6	−53.6	86.4	47.5	99.2	64.4	−0.0062	81.4	0.0088	58.2
B2GP−PLYP−D3(0)ATM	6	−53.6	86.2	47.5	99.2	64.4	−0.0062	81.4	0.0088	58.2
B2GP−PLYP−NL	6	−54.2	82.3	48.3	98.5	63.1	−0.0061	86.6	0.0087	58.9

[a]nc = not computed

Table S5 (continued)

Functional	Group	ΔE_e(rxn)	%−ile ΔE_e(rxn)	%−ile r_e(H−C)	%−ile r_e(C−N)	%−ile r_e(N−O)	Δr_e(H−C)	%−ile Δr_e(H−C)	Δr_e(C−N)	%−ile Δr_e(C−N)
B3LYP	3	−55.8	71.4	97.1	74.4	76.5	−0.0064	64.9	0.0092	49.7
B3LYP5	3	−55.2	76.2	93.3	76.5	79.0	−0.0064	64.1	0.0092	49.5
B3LYP−D3(BJ)2B	3	−56.5	64.9	99.6	73.8	75.4	−0.0065	60.1	0.0091	50.1
B3LYP−D3(BJ)ATM	3	−56.5	64.7	99.6	73.8	75.4	−0.0065	60.1	0.0091	50.1
B3LYP−D3M(BJ)2B	3	−56.7	61.4	98.3	73.3	75.0	−0.0065	59.1	0.0091	50.5
B3LYP−D3M(BJ)ATM	3	−56.7	61.2	98.3	73.3	75.0	−0.0065	59.1	0.0091	50.5
B3LYP−D3M(0)2B	3	−56.2	67.8	91.7	77.7	80.2	−0.0066	54.7	0.0094	47.4
B3LYP−D3M(0)ATM	3	−56.2	67.6	91.7	77.7	80.2	−0.0066	54.7	0.0094	47.4
B3LYP−D3(0)2B	3	−56.0	69.5	96.0	75.8	78.1	−0.0066	54.3	0.0093	48.6
B3LYP−D3(0)ATM	3	−56.0	69.3	96.0	75.8	78.1	−0.0066	54.3	0.0093	48.6
B3LYP−NL	3	−58.6	50.5	90.0	75.4	72.1	−0.0064	68.5	0.0092	49.3
B3LYPS	3	−60.9	38.2	64.4	92.5	86.5	−0.0067	47.0	0.0099	43.4
B3P86	3	−62.2	31.5	54.0	80.8	48.3	−0.0068	37.8	0.0089	55.5
B3P86−D3(BJ)2B	3	−62.6	30.3	55.4	80.0	47.5	−0.0068	35.3	0.0089	55.7
B3P86−D3(BJ)ATM	3	−62.6	30.1	55.4	80.0	47.5	−0.0068	35.3	0.0089	55.7
B3P86−D3(0)2B	3	−62.4	31.1	52.5	81.9	49.4	−0.0069	28.6	0.0090	54.3
B3P86−D3(0)ATM	3	−62.4	30.9	52.5	81.9	49.4	−0.0069	28.6	0.0090	54.3
B3PW91	3	−59.5	47.2	70.8	75.6	41.3	−0.0068	37.8	0.0089	55.3
B3PW91−D3(BJ)2B	3	−60.2	43.2	73.5	74.6	40.6	−0.0069	32.2	0.0089	56.4
B3PW91−D3(BJ)ATM	3	−60.2	43.0	73.5	74.6	40.6	−0.0069	32.2	0.0089	56.4
B3PW91−D3(0)2B	3	−59.7	46.1	67.7	77.3	42.1	−0.0070	26.9	0.0090	52.4
B3PW91−D3(0)ATM	3	−59.7	45.9	67.7	77.3	42.1	−0.0070	26.9	0.0090	52.4
B3PW91−NL	3	−62.6	30.5	60.6	76.9	37.9	−0.0068	40.9	0.0089	54.7
B5050LYP	3	−39.8	30.7	28.8	4.0	8.5	−0.0054	55.3	0.0055	97.9
B86B95	3	−57.4	54.9	85.8	58.3	34.0	−0.0067	47.0	0.0083	70.4
B88B95	3	−58.1	52.8	82.1	52.5	22.5	−0.0065	55.1	0.0085	66.0
B88B95−D3(BJ)2B	3	−58.6	50.9	80.4	51.9	21.7	−0.0066	51.6	0.0085	66.4
B88B95−D3(BJ)ATM	3	−58.6	50.7	80.4	51.9	21.7	−0.0066	51.6	0.0085	66.4
B88B95−D3(0)2B	3	−58.3	52.0	87.7	52.7	25.0	−0.0067	45.3	0.0086	62.0
B88B95−D3(0)ATM	3	−58.3	51.8	87.7	52.7	25.0	−0.0067	45.3	0.0086	62.0
B97−0	3	−58.1	52.6	57.5	92.3	81.5	−0.0068	40.9	0.0088	57.2

Table S5 (continued)

Functional	Group	ΔE_e(rxn)	%–ile ΔE_e(rxn)	%–ile r_e(H–C)	%–ile r_e(C–N)	%–ile r_e(N–O)	Δr_e(H–C)	%–ile Δr_e(H–C)	Δr_e(C–N)	%–ile Δr_e(C–N)
B97–1	3	–58.7	50.3	62.1	87.9	83.5	–0.0066	52.0	0.0087	59.7
B97–1–D3(BJ)2B	3	–59.1	49.3	62.3	87.5	82.3	–0.0066	48.0	0.0087	60.5
B97–1–D3(BJ)ATM	3	–59.1	49.1	62.3	87.5	82.3	–0.0066	48.0	0.0087	60.5
B97–1–D3(0)2B	3	–58.8	50.1	59.4	90.6	85.2	–0.0067	44.1	0.0088	57.6
B97–1–D3(0)ATM	3	–58.8	49.9	59.4	90.6	85.2	–0.0067	44.1	0.0088	57.6
B97–1P	3	–60.7	40.5	61.0	95.8	67.7	–0.0072	20.9	0.0090	52.2
B97–2	3	–59.7	46.3	90.2	67.1	24.8	–0.0071	24.0	0.0080	78.7
B97–2–D3(BJ)2B	3	–60.9	38.6	87.3	64.6	21.3	–0.0072	21.1	0.0079	80.6
B97–2–D3(BJ)ATM	3	–60.9	38.4	87.3	64.6	21.3	–0.0072	21.1	0.0079	80.6
B97–2–D3(0)2B	3	–59.9	45.1	95.2	69.2	28.8	–0.0072	20.0	0.0081	76.4
B97–2–D3(0)ATM	3	–59.9	44.9	95.2	69.2	28.8	–0.0072	20.0	0.0081	76.4
B97–3	3	–56.6	62.8	92.1	56.9	51.3	–0.0065	62.6	0.0080	78.5
B97–K	3	–49.2	89.4	92.9	48.5	91.3	–0.0056	75.6	0.0061	97.1
BB1K	3	–50.9	98.1	38.5	12.7	6.0	–0.0060	94.8	0.0068	88.5
BHandH	3	–61.2	37.2	99.0	4.0	1.7	–0.0057	79.7	0.0065	90.6
BHandHLYP	3	–37.5	25.7	27.3	4.4	18.8	–0.0053	53.7	0.0053	96.5
BMK	4	–53.6	85.8	44.6	44.0	93.1	–0.0052	44.9	0.0086	64.5
BMK–D3(BJ)2B	4	–54.0	83.3	44.8	43.5	93.3	–0.0053	46.6	0.0086	64.9
BMK–D3(BJ)ATM	4	–54.0	83.1	44.8	43.5	93.3	–0.0053	46.6	0.0086	64.9
BMK–D3(0)2B	4	–53.8	85.0	42.5	44.6	92.1	–0.0053	53.9	0.0087	59.9
BMK–D3(0)ATM	4	–53.8	84.8	42.5	44.6	92.1	–0.0053	53.9	0.0087	59.9
CAM–B3LYP	5	–54.3	81.4	87.1	34.8	58.8	–0.0058	87.9	0.0079	80.0
CAM–B3LYP–D3(BJ)2B	5	–54.6	79.5	88.3	34.0	58.3	–0.0058	90.6	0.0079	80.2
CAM–B3LYP–D3(BJ)ATM	5	–54.6	79.3	88.3	34.0	58.3	–0.0058	90.6	0.0079	80.2
CAM–B3LYP–D3(0)2B	5	–54.5	81.2	82.9	37.1	60.4	–0.0059	92.9	0.0079	79.3
CAM–B3LYP–D3(0)ATM	5	–54.5	81.0	82.9	37.1	60.4	–0.0059	92.9	0.0079	79.3
CAM–LDA0	0	–70.6	12.9	42.9	23.8	2.7	–0.0062	82.5	0.0087	60.3
CAP0	3	–56.5	65.3	51.3	66.0	26.7	–0.0068	39.2	0.0082	75.4
DLDF	4	–8.8	3.3	22.3	9.8	97.9	–0.0057	80.8	0.0031	67.0
DLDF+D09	4	–8.8	3.5	22.5	9.8	97.9	–0.0057	80.8	0.0031	67.0
DLDF+D10	4	–8.8	3.8	22.5	9.8	97.9	–0.0057	80.8	0.0031	67.0

Table S5 (continued)

Functional	Group	ΔE_e(rxn)	%−ile ΔE_e(rxn)	%−ile r_e(H−C)	%−ile r_e(C−N)	%−ile r_e(N−O)	Δr_e(H−C)	%−ile Δr_e(H−C)	Δr_e(C−N)	%−ile Δr_e(C−N)
DSD−PBEB95	6	−57.4	55.1	62.7	94.4	40.4	−0.0064	69.5	0.0086	64.5
DSD−PBEB95−D3(BJ)	6	−54.8	78.9	71.7	95.4	46.5	−0.0063	73.1	0.0082	75.4
DSD−PBEB95−NL	6	−55.0	77.7	75.2	95.6	46.5	−0.0063	75.2	0.0081	75.8
EDF2	3	−63.2	29.0	89.8	60.4	40.0	−0.0065	56.4	0.0099	44.7
HF	0	14.4	0.4	24.4	1.5	25.4	−0.0043	13.8	−0.0010	34.7
HF−3c	0	−49.5	90.6	1.5	0.0	0.0	−0.0063	73.1	0.0086	62.8
HF+D	0	14.4	0.6	24.6	1.7	25.6	−0.0043	13.6	−0.0010	34.9
HF−D3(BJ)2B	0	10.5	1.3	16.9	1.0	18.3	−0.0044	14.6	−0.0014	30.9
HF−D3(BJ)ATM	0	10.5	1.5	16.9	1.0	18.3	−0.0044	14.6	−0.0015	30.7
HF−D3M(BJ)2B	0	8.1	2.1	13.3	0.2	10.8	−0.0043	14.2	−0.0019	28.0
HF−D3M(BJ)ATM	0	8.1	2.3	13.3	0.2	10.8	−0.0043	14.2	−0.0019	28.0
HF−D3M(0)2B	0	10.0	1.7	17.9	0.6	19.6	−0.0044	15.0	−0.0022	27.6
HF−D3M(0)ATM	0	10.0	1.9	17.9	0.6	19.6	−0.0044	15.0	−0.0022	27.6
HF−D3(0)2B	0	14.2	0.8	25.2	1.9	26.3	−0.0045	16.7	−0.0009	35.1
HF−D3(0)ATM	0	14.2	1.0	25.2	1.9	26.3	−0.0045	16.7	−0.0009	35.1
HJS−B97x	5	−65.0	25.1	23.3	81.9	7.3	−0.0073	18.2	0.0082	73.9
HJS−PBE	5	−59.8	45.3	65.0	66.3	30.0	−0.0069	34.7	0.0083	71.8
HJS−PBEsol	5	−69.1	13.8	32.3	68.8	6.5	−0.0071	23.0	0.0086	64.9
hPBEint	3	−70.1	13.6	21.0	99.8	33.3	−0.0075	16.5	0.0092	49.1
HSE03	5	−59.2	48.6	94.6	56.0	22.5	−0.0068	41.8	0.0083	70.4
HSE03−D3(BJ)2B	5	−59.5	47.0	95.6	55.6	22.1	−0.0068	39.5	0.0083	70.8
HSE03−D3(BJ)ATM	5	−59.5	46.8	95.6	55.6	22.1	−0.0068	39.5	0.0083	70.8
HSE03−D3(0)2B	5	−59.3	48.0	92.5	56.9	23.8	−0.0068	37.8	0.0084	68.3
HSE03−D3(0)ATM	5	−59.3	47.8	92.5	56.9	23.8	−0.0068	37.8	0.0084	68.3
HSE06	5	−59.1	48.9	71.5	61.5	27.7	−0.0068	38.6	0.0083	68.9
HSE06−D3(BJ)2B	5	−59.4	47.6	71.9	61.0	26.7	−0.0068	35.9	0.0083	69.1
HSE06−D3(BJ)ATM	5	−59.4	47.4	71.9	61.0	26.7	−0.0068	35.9	0.0083	69.1
HSE06−D3(0)2B	5	−59.2	48.4	71.9	61.7	27.3	−0.0069	31.7	0.0083	69.1
HSE06−D3(0)ATM	5	−59.2	48.2	71.9	61.7	27.3	−0.0069	31.7	0.0083	69.1
KMLYP	3	−53.1	89.1	33.1	3.5	1.5	−0.0054	55.9	0.0055	98.7
KSDT	0	−103.2	2.9	1.9	72.5	20.6	−0.0078	10.2	0.0129	31.7

Table S5 (continued)

Functional	Group	ΔE_e(rxn)	%−ile ΔE_e(rxn)	%−ile r_e(H−C)	%−ile r_e(C−N)	%−ile r_e(N−O)	Δr_e(H−C)	%−ile Δr_e(H−C)	Δr_e(C−N)	%−ile Δr_e(C−N)
LC−BOP	1	−56.9	59.7	52.1	7.1	32.5	−0.0052	43.8	0.0071	87.3
LC−VV10	1	−56.0	69.7	51.9	14.6	26.0	−0.0056	71.4	0.0067	89.1
LDA0	0	−72.0	11.3	21.5	54.0	6.3	−0.0067	45.3	0.0095	45.9
LRC−ωPBE	5	−63.2	29.2	22.9	83.1	64.2	−0.0065	57.8	0.0082	72.7
LRC−ωPBEh	5	−58.9	49.5	44.0	56.7	31.0	−0.0066	48.9	0.0075	84.1
M05	4	−61.7	34.4	84.6	86.0	7.9	−0.0080	5.8	0.0079	79.7
M05−2X	4	−51.1	99.6	86.3	26.7	43.3	−0.0050	28.2	0.0077	82.0
M05−2X−D3(0)2B	4	−51.1	99.4	86.7	26.7	43.8	−0.0049	26.5	0.0077	82.0
M05−2X−D3(0)ATM	4	−51.1	99.2	86.7	26.7	43.8	−0.0049	26.5	0.0077	82.0
M05−D3(0)2B	4	−61.7	33.8	81.7	86.3	8.1	−0.0080	4.8	0.0080	78.9
M05−D3(0)ATM	4	−61.7	33.6	81.7	86.3	8.1	−0.0080	4.8	0.0080	78.9
M06	4	−60.6	41.5	93.8	53.3	12.1	−0.0074	17.7	0.0080	77.9
M06−2X	4	−52.8	90.2	79.8	37.5	59.0	−0.0058	85.6	0.0066	90.0
M06−2X−D3(0)2B	4	−52.8	90.0	79.2	37.5	59.0	−0.0057	85.0	0.0066	90.0
M06−2X−D3(0)ATM	4	−52.8	89.8	79.2	37.5	59.0	−0.0057	85.0	0.0066	90.0
M06−D3(0)2B	4	−60.6	41.3	93.8	53.3	12.1	−0.0074	17.1	0.0080	77.9
M06−D3(0)ATM	4	−60.6	41.1	93.8	53.3	12.1	−0.0074	17.1	0.0080	77.9
M06−HF	4	−41.5	39.5	77.5	4.6	87.9	−0.0030	1.7	0.0054	96.9
M06−HF−D3(0)2B	4	−41.5	40.1	76.5	4.8	88.1	−0.0030	1.3	0.0054	97.3
M06−HF−D3(0)ATM	4	−41.5	40.3	76.5	4.8	88.1	−0.0030	1.3	0.0054	97.3
M08−HX	4	−53.8	84.6	43.5	36.5	34.6	−0.0062	79.5	0.0075	83.5
M08−HX−D3(0)2B	4	−53.8	84.3	43.1	36.5	34.6	−0.0062	80.2	0.0075	83.5
M08−HX−D3(0)ATM	4	−53.8	84.1	43.1	36.5	34.6	−0.0062	80.2	0.0075	83.5
M08−SO	4	−52.9	89.6	21.3	52.3	68.5	−0.0055	63.9	0.0070	87.9
M11	5	−55.7	73.7	31.3	42.3	38.5	−0.0053	53.0	0.0101	40.7
M11−D3(BJ)2B	5	−55.8	73.1	31.3	42.3	38.5	−0.0053	53.0	0.0101	40.7
M11−D3(BJ)ATM	5	−55.8	72.9	31.3	42.3	38.5	−0.0053	53.0	0.0101	40.7
M11−D3(0)2B	5	−55.8	71.8	30.8	43.1	39.6	−0.0054	55.3	0.0101	40.3
M11−D3(0)ATM	5	−55.8	71.6	30.8	43.1	39.6	−0.0054	55.3	0.0101	40.3
mB3LYP−RC04	3	−61.6	34.9	41.5	53.1	61.5	−0.0063	77.0	0.0092	49.7
MGGA−MVS	2	−70.7	12.7	51.5	98.1	5.8	−0.0084	2.1	0.0111	36.7

Table S5 (continued)

Functional	Group	ΔE_e(rxn)	%−ile ΔE_e(rxn)	%−ile r_e(H−C)	%−ile r_e(C−N)	%−ile r_e(N−O)	Δr_e(H−C)	%−ile Δr_e(H−C)	Δr_e(C−N)	%−ile Δr_e(C−N)
MGGA−MVSh	4	−57.0	58.0	74.4	29.0	1.9	−0.0070	25.7	0.0085	66.4
MN12−L	2	−52.1	93.9	54.2	27.7	17.7	−0.0081	3.3	0.0059	99.0
MN12−L−D3(BJ)2B	2	−52.1	92.9	54.2	27.7	17.7	−0.0081	3.3	0.0059	99.0
MN12−L−D3(BJ)ATM	2	−52.1	92.7	54.2	27.7	17.7	−0.0081	3.3	0.0059	99.0
MN12−L−D3(0)2B	2	−52.1	91.6	56.5	28.5	19.2	−0.0082	2.9	0.0060	98.3
MN12−L−D3(0)ATM	2	−52.1	91.4	56.5	28.5	19.2	−0.0082	2.9	0.0060	98.3
MN12−SX	5	−52.1	94.8	74.6	29.8	55.0	−0.0079	8.1	0.0053	94.2
MN12−SX−D3(BJ)2B	5	−52.1	92.5	74.6	29.8	55.0	−0.0079	8.1	0.0053	94.2
MN12−SX−D3(BJ)ATM	5	−52.1	92.3	74.6	29.8	55.0	−0.0079	8.1	0.0053	94.2
MN12−SX−D3(0)2B	5	−52.1	93.7	72.7	31.9	56.5	−0.0079	7.1	0.0053	96.0
MN12−SX−D3(0)ATM	5	−52.1	93.5	72.7	31.9	56.5	−0.0079	7.1	0.0053	96.0
MN15	5	−57.0	59.3	67.7	66.5	69.6	−0.0054	62.0	0.0080	77.2
MN15−D3(BJ)2B	5	−57.0	58.7	67.7	66.5	69.6	−0.0054	62.0	0.0080	77.2
MN15−D3(BJ)ATM	5	−57.0	59.1	67.7	66.5	69.6	−0.0054	62.0	0.0080	77.2
MPW1B95	3	−58.0	53.4	65.6	41.5	14.4	−0.0064	64.3	0.0082	73.9
MPW1B95−D3(BJ)2B	3	−58.2	52.4	65.2	41.0	13.3	−0.0064	63.5	0.0082	72.7
MPW1B95−D3(BJ)ATM	3	−58.2	52.2	65.2	41.0	13.3	−0.0064	63.5	0.0082	72.7
MPW1B95−D3(0)2B	3	−58.1	53.2	67.3	41.9	14.8	−0.0065	59.1	0.0083	71.4
MPW1B95−D3(0)ATM	3	−58.1	53.0	67.3	41.9	14.8	−0.0065	59.1	0.0083	71.4
MPW1K	3	−47.6	80.0	45.4	11.0	5.4	−0.0060	96.5	0.0061	96.7
mPW1LYP	3	−51.8	95.8	82.7	56.0	71.9	−0.0062	81.8	0.0085	66.0
mPW1LYP−D3(0)2B	3	−52.0	95.2	88.8	59.0	72.3	−0.0063	71.8	0.0086	62.0
mPW1LYP−D3(0)ATM	3	−52.0	95.0	88.8	59.0	72.3	−0.0063	71.8	0.0086	62.0
mPW1PBE	3	−57.7	53.7	79.2	59.8	29.8	−0.0067	43.0	0.0082	72.7
mPW1PW	3	−57.1	57.8	93.3	55.2	31.3	−0.0066	47.4	0.0083	72.0
mPW1PW−D3(BJ)2B	3	−57.6	54.1	94.8	54.8	30.6	−0.0067	44.1	0.0082	72.2
mPW1PW−D3(BJ)ATM	3	−57.6	53.9	94.8	54.8	30.6	−0.0067	44.1	0.0082	72.2
mPW1PW−D3(0)2B	3	−57.2	56.8	91.0	57.5	31.5	−0.0068	40.1	0.0083	69.1
mPW1PW−D3(0)ATM	3	−57.2	56.6	91.0	57.5	31.5	−0.0068	40.1	0.0083	69.1
MPW3LYP	3	−56.3	67.0	96.9	67.7	70.2	−0.0063	69.9	0.0090	51.8
mPW3PW	3	−61.5	35.5	80.8	73.1	36.9	−0.0068	40.1	0.0090	53.7

Table S5 (continued)

Functional	Group	ΔE_e(rxn)	%–ile ΔE_e(rxn)	%–ile r_e(H–C)	%–ile r_e(C–N)	%–ile r_e(N–O)	Δr_e(H–C)	%–ile Δr_e(H–C)	Δr_e(C–N)	%–ile Δr_e(C–N)
MPWB1K	3	–51.1	99.8	35.6	10.8	3.3	–0.0060	99.6	0.0067	89.1
MPWB1K–D3(BJ)2B	3	–51.3	97.9	35.2	10.4	2.9	–0.0060	99.0	0.0067	89.6
MPWB1K–D3(BJ)ATM	3	–51.3	97.7	35.2	10.4	2.9	–0.0060	99.0	0.0067	89.6
MPWB1K–D3(0)2B	3	–51.2	98.5	36.3	11.3	4.8	–0.0060	95.2	0.0067	88.7
MPWB1K–D3(0)ATM	3	–51.2	98.3	36.3	11.3	4.8	–0.0060	95.2	0.0067	88.7
N12–SX	5	–61.5	35.9	49.0	39.4	14.4	–0.0058	86.2	0.0103	38.6
N12–SX–D3(BJ)2B	5	–61.8	33.0	48.3	39.0	13.8	–0.0058	87.9	0.0103	38.8
N12–SX–D3(BJ)ATM	5	–61.8	32.8	48.3	39.0	13.8	–0.0058	87.9	0.0103	38.8
N12–SX–D3(0)2B	5	–61.7	34.2	50.2	40.0	15.4	–0.0058	92.5	0.0104	38.0
N12–SX–D3(0)ATM	5	–61.7	34.0	50.2	40.0	15.4	–0.0058	92.5	0.0104	38.0
O3LYP	3	–61.0	38.0	66.7	98.3	48.5	–0.0078	9.0	0.0089	56.2
O3LYP–D3(BJ)2B	3	–61.6	35.3	69.0	98.8	47.9	–0.0079	7.5	0.0089	56.8
O3LYP–D3(BJ)ATM	3	–61.6	35.1	69.0	98.8	47.9	–0.0079	7.5	0.0089	56.8
O3LYP–D3(0)2B	3	–61.2	37.6	63.1	96.5	50.2	–0.0080	6.5	0.0090	52.8
O3LYP–D3(0)ATM	3	–61.2	37.4	63.1	96.5	50.2	–0.0080	6.5	0.0090	52.8
PBE0	3	–59.6	46.6	62.9	64.2	29.2	–0.0068	35.7	0.0082	74.9
PBE0–1/3	3	–54.9	78.3	98.1	38.3	7.7	–0.0065	58.7	0.0072	85.4
PBE0–2	6	–55.8	71.2	45.2	91.0	12.9	–0.0063	75.8	0.0078	81.4
PBE0–D3(BJ)2B	3	–59.9	44.7	64.0	63.8	28.3	–0.0069	32.2	0.0081	76.0
PBE0–D3(BJ)ATM	3	–59.9	44.5	64.0	63.8	28.3	–0.0069	32.2	0.0081	76.0
PBE0–D3M(BJ)2B	3	–60.1	44.3	64.4	63.3	27.7	–0.0069	32.2	0.0081	76.4
PBE0–D3M(BJ)ATM	3	–60.1	44.1	64.4	63.3	27.7	–0.0069	32.2	0.0081	76.4
PBE0–D3M(0)2B	3	–60.2	43.6	76.5	59.4	22.9	–0.0069	32.2	0.0082	74.3
PBE0–D3M(0)ATM	3	–60.2	43.4	76.5	59.4	22.9	–0.0069	32.2	0.0082	74.3
PBE0–D3(0)2B	3	–59.7	45.7	61.7	64.6	29.4	–0.0069	30.5	0.0082	72.7
PBE0–D3(0)ATM	3	–59.7	45.5	61.7	64.6	29.4	–0.0069	30.5	0.0082	72.7
PBE0–DH	6	–54.2	82.5	71.3	46.9	9.8	–0.0064	68.7	0.0071	86.6
PBE0–DH–D3(BJ)2B	6	–54.5	80.4	69.8	46.3	9.4	–0.0064	66.8	0.0071	87.5
PBE0–DH–D3(BJ)ATM	6	–54.5	80.2	69.8	46.3	9.4	–0.0064	66.8	0.0071	87.5
PBE0–DH–D3(0)2B	6	–54.3	81.8	73.1	47.1	10.0	–0.0064	66.8	0.0071	85.8
PBE0–DH–D3(0)ATM	6	–54.3	81.6	73.1	47.1	10.0	–0.0064	65.3	0.0071	85.8

Table S5 (continued)

Functional	Group	ΔE_e(rxn)	%−ile ΔE_e(rxn)	%−ile r_e(H−C)	%−ile r_e(C−N)	%−ile r_e(N−O)	Δr_e(H−C)	%−ile Δr_e(H−C)	Δr_e(C−N)	%−ile Δr_e(C−N)
PBE0−NL	3	−61.0	37.8	59.2	64.4	25.8	−0.0068	37.0	0.0082	75.2
PBE50	3	−45.9	66.6	44.4	6.0	2.1	−0.0059	97.5	0.0053	95.2
PBEh−3c	3	−53.2	87.5	94.4	12.5	2.5	−0.0059	99.4	0.0069	88.3
PW6B95	4	−56.7	62.4	47.9	47.1	33.1	−0.0063	69.9	0.0086	63.9
PW6B95−D3(BJ)2B	4	−56.9	60.1	47.1	47.1	32.5	−0.0064	68.7	0.0086	63.9
PW6B95−D3(BJ)ATM	4	−56.9	59.9	47.1	47.1	32.5	−0.0064	68.7	0.0086	63.9
PW6B95−D3(0)2B	4	−56.8	61.0	49.2	48.8	33.5	−0.0064	67.4	0.0086	61.6
PW6B95−D3(0)ATM	4	−56.8	60.8	49.2	48.8	33.5	−0.0064	67.4	0.0086	61.6
PW86B95	3	−56.6	63.5	61.3	50.8	34.2	−0.0065	62.8	0.0084	68.7
PWB6K	3	−49.0	87.7	26.5	7.3	3.5	−0.0058	87.9	0.0064	92.5
PWB6K−D3(BJ)2B	3	−49.1	88.7	26.0	7.3	3.5	−0.0058	88.7	0.0064	92.5
PWB6K−D3(BJ)ATM	3	−49.1	88.9	26.0	7.3	3.5	−0.0058	88.7	0.0064	92.5
PWB6K−D3(0)2B	3	−49.0	88.3	26.9	7.9	4.4	−0.0058	91.4	0.0064	91.9
PWB6K−D3(0)ATM	3	−49.0	88.5	26.9	7.9	4.4	−0.0058	91.4	0.0064	91.9
PWPB95	6	−55.7	73.9	77.3	88.8	54.2	−0.0063	74.5	0.0086	63.0
PWPB95−D3(BJ)2B	6	−55.9	71.0	76.0	88.3	53.8	−0.0063	74.5	0.0086	63.3
PWPB95−D3(BJ)ATM	6	−55.9	70.8	76.0	88.3	53.8	−0.0063	74.5	0.0086	63.3
PWPB95−D3(0)2B	6	−55.8	72.7	77.7	90.2	54.6	−0.0063	72.4	0.0086	61.0
PWPB95−D3(0)ATM	6	−55.8	72.4	77.7	90.2	54.6	−0.0063	72.4	0.0086	61.0
PWPB95−NL	6	−56.3	67.4	78.5	89.6	53.5	−0.0063	75.2	0.0086	63.3
revB3LYP	3	−58.3	51.4	84.8	74.2	69.4	−0.0064	63.3	0.0093	48.4
revPBE0	3	−53.1	88.1	60.8	76.5	44.6	−0.0070	28.0	0.0077	81.8
revPBE0−D3(BJ)2B	3	−54.1	82.9	63.1	75.0	42.5	−0.0071	24.4	0.0077	82.9
revPBE0−D3(BJ)ATM	3	−54.1	82.7	63.1	75.0	42.5	−0.0071	24.4	0.0077	82.9
revPBE0−D3(0)2B	3	−53.4	87.3	55.0	78.5	46.0	−0.0072	21.1	0.0078	81.0
revPBE0−D3(0)ATM	3	−53.4	87.1	55.0	78.5	46.0	−0.0072	21.1	0.0078	81.0
revPBE0−NL	3	−56.6	64.3	51.7	78.1	40.0	−0.0069	29.2	0.0077	81.6
revSCAN0	4	−50.6	97.1	56.3	26.5	5.6	−0.0077	12.5	0.0073	84.3
SB98−1a	3	−57.4	55.5	32.5	25.2	11.9	−0.0061	86.6	0.0090	54.1
SB98−1b	3	−60.2	42.4	24.2	99.6	57.7	−0.0074	17.1	0.0083	71.2
SB98−1c	3	−58.3	51.6	57.7	92.7	81.3	−0.0068	39.5	0.0088	57.4

Table S5 (continued)

Functional	Group	ΔE_e(rxn)	%−ile ΔE_e(rxn)	%−ile r_e(H−C)	%−ile r_e(C−N)	%−ile r_e(N−O)	Δr_e(H−C)	%−ile Δr_e(H−C)	Δr_e(C−N)	%−ile Δr_e(C−N)
SB98−2a	3	−56.5	65.1	88.1	68.8	74.8	−0.0062	82.5	0.0087	59.5
SB98−2b	3	−57.4	55.3	49.6	76.3	66.9	−0.0065	63.0	0.0086	61.0
SB98−2c	3	−57.0	58.9	70.6	80.4	74.6	−0.0064	64.9	0.0088	58.0
SCAN0	4	−49.4	90.4	53.8	25.0	10.4	−0.0063	75.8	0.0076	83.3
SOGGA11−X	3	−51.9	95.6	86.5	46.0	45.2	−0.0068	40.7	0.0051	92.3
SOGGA11−X−D3(BJ)2B	3	−52.4	91.2	89.2	45.4	44.2	−0.0068	37.0	0.0050	91.4
SOGGA11−X−D3(BJ)ATM	3	−52.4	91.0	89.2	45.4	44.2	−0.0068	37.0	0.0050	91.4
SOGGA11−X−D3(0)2B	3	−52.1	94.6	81.0	48.1	45.6	−0.0069	34.7	0.0052	93.5
SOGGA11−X−D3(0)ATM	3	−52.1	94.4	81.0	48.1	45.6	−0.0069	34.7	0.0052	93.5
SPW92	0	−103.2	2.7	3.5	77.1	18.8	−0.0078	10.6	0.0129	31.5
SVWN	0	−106.3	2.5	7.7	84.0	10.6	−0.0077	12.7	0.0129	31.9
Teter93	0	−102.6	3.1	3.1	78.1	16.5	−0.0078	10.4	0.0129	31.1
τ−HCTHh	4	−60.8	38.8	59.4	92.9	69.2	−0.0068	36.7	0.0094	47.2
TH−FL	1	−88.1	4.2	12.1	94.6	5.2	−0.0079	7.9	0.0128	32.8
TPSSh	3	−56.1	68.7	80.2	95.2	83.3	−0.0072	20.5	0.0090	52.0
TPSSh−D3(BJ)2B	3	−56.6	63.9	82.3	94.8	82.3	−0.0072	19.6	0.0090	52.8
TPSSh−D3(BJ)ATM	3	−56.6	63.7	82.3	94.8	82.3	−0.0072	19.6	0.0090	52.8
TPSSh−D3(0)2B	3	−56.3	67.2	78.1	95.8	84.2	−0.0073	18.2	0.0091	50.9
TPSSh−D3(0)ATM	3	−56.3	66.8	78.1	95.8	84.2	−0.0073	18.2	0.0091	50.9
tuned−CAM−B3LYP	5	−71.4	11.9	38.3	97.3	81.9	−0.0066	47.4	0.0102	39.2
ωB97	1	−53.2	87.9	36.7	51.0	69.0	−0.0064	67.8	0.0058	99.8
ωB97M−D3(BJ)	5	−50.6	97.3	98.8	45.0	79.4	−0.0056	73.9	0.0063	93.9
ωB97M−V	5	−51.8	96.5	90.8	46.3	77.1	−0.0055	66.6	0.0063	93.3
ωB97X	5	−53.9	83.9	60.0	40.4	48.5	−0.0062	84.1	0.0065	90.8
ωB97X−D	5	−55.5	75.2	85.0	49.2	38.3	−0.0063	69.9	0.0077	82.0
ωB97X−D3	5	−54.8	78.5	66.0	44.4	41.5	−0.0063	71.8	0.0069	88.1
ωB97X−D3(BJ)	5	−52.6	90.8	44.2	51.3	71.7	−0.0062	84.1	0.0059	99.6
ωB97X−D3(0)2B	5	−54.0	83.7	60.2	40.6	48.5	−0.0062	78.1	0.0065	91.0
ωB97X−D3(0)ATM	5	−54.0	83.5	60.2	40.6	48.5	−0.0062	78.1	0.0065	91.0
ωB97X−V	5	−53.6	86.0	39.2	51.7	71.0	−0.0061	91.0	0.0060	98.1
ωPBE	5	−57.0	59.5	38.1	33.3	36.0	−0.0058	91.4	0.0071	85.6

Table S5 (continued)

Functional	Group	ΔE_e(rxn)	%−ile ΔE_e(rxn)	%−ile r_e(H−C)	%−ile r_e(C−N)	%−ile r_e(N−O)	Δr_e(H−C)	%−ile Δr_e(H−C)	Δr_e(C−N)	%−ile Δr_e(C−N)
ωPBE0	5	−51.8	96.0	71.0	16.5	8.8	−0.0059	98.7	0.0061	97.7
ωPBE−D3(BJ)2B	5	−57.3	56.2	38.8	32.9	35.6	−0.0059	93.3	0.0071	86.2
ωPBE−D3(BJ)ATM	5	−57.3	55.9	38.8	32.9	35.6	−0.0059	93.3	0.0071	86.2
ωPBE−D3M(BJ)2B	5	−57.5	54.7	40.4	32.5	35.2	−0.0059	95.8	0.0071	86.6
ωPBE−D3M(BJ)ATM	5	−57.5	54.5	40.4	32.5	35.2	−0.0059	95.8	0.0071	86.6
ωPBE−D3M(0)2B	5	−57.1	57.2	36.9	35.8	37.5	−0.0059	96.7	0.0072	84.6
ωPBE−D3M(0)ATM	5	−57.1	57.0	36.9	35.8	37.5	−0.0059	96.7	0.0072	84.6
ωPBE−D3(0)2B	5	−57.1	57.6	37.5	34.0	36.9	−0.0059	96.7	0.0072	85.0
ωPBE−D3(0)ATM	5	−57.1	57.4	37.5	34.0	36.9	−0.0059	96.7	0.0072	85.0
X3LYP	3	−55.6	74.5	99.2	68.5	70.8	−0.0063	69.9	0.0090	53.9
X3LYP−D3(BJ)2B	3	−56.2	68.3	97.7	68.1	70.4	−0.0064	65.3	0.0089	54.7
X3LYP−D3(BJ)ATM	3	−56.2	68.1	97.7	68.1	70.4	−0.0064	65.3	0.0089	54.7
X3LYP−D3(0)2B	3	−55.8	73.5	97.1	69.6	71.0	−0.0065	60.1	0.0090	51.4
X3LYP−D3(0)ATM	3	−55.8	73.3	97.1	69.6	71.0	−0.0065	60.1	0.0090	51.4
XB1K	3	−40.7	36.1	0.0	3.8	1.3	−0.0077	13.4	0.0063	93.1

Table S6. DFT percentiles for predicting HCNO ω_1–ω_4 vibrational frequencies and shifts [$\Delta\omega$(H–C), $\Delta\omega$(C–N)] relative to HCN

Functional	Group	%–ile ω_1	%–ile ω_2	%–ile ω_3	%–ile ω_4	$\Delta\omega$(H–C)	%–ile $\Delta\omega$(H–C)	$\Delta\omega$(C–N)	%–ile $\Delta\omega$(C–N)
B2PLYP	6	68.8	97.7	81.9	80.0	52.9	96.9	148.1	97.9
B2PLYP–D3(BJ)2B	6	69.4	97.5	81.0	78.8	50.6	80.0	146.5	95.2
B2PLYP–D3(BJ)ATM	6	67.1	96.9	81.3	79.6	53.0	97.5	148.2	97.5
B2PLYP–D3M(BJ)2B	6	68.5	97.1	80.4	79.4	52.2	91.4	145.9	94.2
B2PLYP–D3M(BJ)ATM	6	66.3	96.7	80.6	78.3	52.8	95.6	148.1	97.7
B2PLYP–D3M(0)2B	6	70.0	97.9	82.9	77.3	52.8	94.8	146.6	95.4
B2PLYP–D3M(0)ATM	6	71.7	98.5	83.1	78.5	54.2	92.5	147.8	99.4
B2PLYP–D3(0)2B	6	70.6	98.1	83.3	77.5	53.5	97.7	146.7	95.8
B2PLYP–D3(0)ATM	6	72.5	98.3	83.5	75.2	53.8	94.2	148.2	97.3
B2PLYP–NL	6	73.3	97.3	80.2	81.9	52.2	91.2	148.4	96.2
B86bPBE	1	19.0	83.8	87.9	92.1	41.7	35.3	130.0	53.2
B97–D	1	23.8	87.3	86.9	96.0	43.2	39.0	130.1	53.7
B97–D3(BJ)	1	25.2	88.8	84.4	95.8	38.0	30.1	127.8	48.9
B97–D3M(BJ)	1	25.8	90.8	80.0	95.4	36.5	25.9	129.3	51.1
B97^{GGA}–1	1	40.8	85.2	37.5	75.0	46.6	52.8	156.2	77.2
B97M–D3(BJ)	1	36.0	50.6	34.6	45.8	43.1	38.8	128.0	49.3
B97M–V	1	40.6	51.0	34.4	45.6	42.5	37.2	128.3	50.3
BLYP	1	13.1	51.7	72.9	63.8	34.3	20.7	101.4	10.4
BLYP–D3(BJ)2B	1	12.7	51.3	74.6	61.9	33.5	19.6	101.0	9.6
BLYP–D3(BJ)ATM	1	12.9	51.5	74.4	61.7	33.5	19.8	101.0	9.8
BLYP–D3M(BJ)2B	1	13.5	51.9	76.3	60.4	32.9	19.4	101.0	10.0
BLYP–D3M(BJ)ATM	1	13.3	52.1	75.8	60.6	32.9	19.2	101.0	10.2
BLYP–D3M(0)2B	1	9.4	48.5	68.1	65.2	35.6	24.0	99.2	6.7
BLYP–D3M(0)ATM	1	9.2	48.8	68.3	65.0	35.6	23.8	99.2	6.9
BLYP–D3(0)2B	1	10.8	50.0	70.2	64.4	35.9	24.8	100.8	9.2
BLYP–D3(0)ATM	1	11.0	50.2	70.0	64.6	35.9	25.1	100.8	9.4
BLYP–NL	1	7.1	53.3	81.5	61.5	32.2	16.9	103.3	13.2
BOP	1	11.7	47.3	76.7	66.0	28.9	10.9	100.2	8.8
BOP–D3(BJ)2B	1	12.5	48.1	79.8	63.1	27.9	10.2	100.2	8.4
BOP–D3(BJ)ATM	1	12.3	48.3	79.4	63.3	27.9	10.0	100.2	8.6
BOP–D3(0)2B	1	6.7	45.6	68.8	68.8	32.9	18.8	99.6	7.1

Table S6 (continued)

Functional	Group	%-ile ω_1	%-ile ω_2	%-ile ω_3	%-ile ω_4	$\Delta\omega$(H-C)	%-ile $\Delta\omega$(H-C)	$\Delta\omega$(C-N)	%-ile $\Delta\omega$(C-N)
BOP-D3(0)ATM	1	6.9	45.8	68.5	68.5	32.9	19.0	99.6	7.7
BP86	1	9.0	72.7	93.1	86.7	34.9	21.9	122.9	33.6
BP86-D3(BJ)2B	1	10.2	73.5	92.3	88.1	35.7	24.6	123.3	36.3
BP86-D3(BJ)ATM	1	10.0	73.3	92.1	88.3	35.7	24.4	123.3	36.5
BP86-D3M(BJ)2B	1	10.6	74.0	91.5	87.7	35.5	23.6	123.2	35.9
BP86-D3M(BJ)ATM	1	10.4	73.8	91.7	87.5	35.5	23.4	123.2	35.5
BP86-D3M(0)2B	1	7.9	70.8	95.6	89.0	37.8	29.2	123.1	34.0
BP86-D3M(0)ATM	1	7.7	70.6	95.8	89.2	37.8	28.8	123.1	34.2
BP86-D3(0)2B	1	8.3	71.3	95.2	88.5	37.7	28.2	123.1	34.7
BP86-D3(0)ATM	1	8.1	71.0	95.4	88.5	37.7	28.0	123.1	34.4
BP86-NL	1	4.8	76.0	89.2	85.4	23.2	5.8	107.1	15.9
BP86-VWN	1	15.2	78.5	89.4	90.0	36.9	26.3	124.9	39.9
core-DSD-BLYP	6	53.1	96.0	62.3	69.8	48.4	65.1	157.6	71.4
core-DSD-BLYP-D3(BJ)2B	6	52.5	95.4	61.9	71.5	51.1	81.0	158.7	68.7
core-DSD-BLYP-D3(BJ)ATM	6	52.9	95.6	62.1	71.0	48.4	64.7	157.7	71.2
DSD-BLYP	6	36.7	84.2	43.1	50.2	52.5	92.9	171.8	37.0
DSD-BLYP-D3(BJ)	6	45.8	90.0	53.5	59.4	51.0	80.8	161.9	60.1
DSD-BLYP-D3(BJ)2B	6	36.3	83.5	42.7	50.0	50.2	77.0	171.3	38.2
DSD-BLYP-D3(BJ)ATM	6	36.5	84.0	42.9	50.8	52.6	93.3	172.0	36.7
DSD-BLYP-D3(0)2B	6	38.8	84.6	44.2	49.2	54.6	88.9	173.3	32.4
DSD-BLYP-D3(0)ATM	6	39.2	84.8	44.4	49.8	52.8	94.6	171.6	37.4
DSD-BLYP-NL	6	46.5	91.0	54.6	59.2	51.5	86.4	161.8	60.3
DSD-PBEP86	6	63.3	91.7	45.8	57.1	48.8	68.5	164.3	56.6
DSD-PBEP86-D3(BJ)	6	79.6	95.8	51.9	70.0	49.9	75.8	171.6	37.6
DSD-PBEP86-NL	6	82.1	96.3	52.1	70.8	48.4	64.9	170.3	40.3
DSD-PBEPBE	6	57.5	86.7	37.3	52.5	47.5	60.5	171.0	38.6
DSD-PBEPBE-D3(BJ)	6	78.5	93.5	47.9	69.6	47.4	59.3	162.4	59.5
DSD-PBEPBE-NL	6	81.0	93.8	47.7	70.6	44.2	43.2	160.7	61.4
EDF1	1	28.5	94.0	81.7	96.5	41.4	34.9	132.6	58.9
FT97	1	5.8	66.9	90.6	86.9	36.7	26.1	131.6	57.6
GAM	1	52.7	34.2	6.9	44.2	77.9	10.6	193.1	11.9

Table S6 (continued)

Functional	Group	%−ile ω_1	%−ile ω_2	%−ile ω_3	%−ile ω_4	$\Delta\omega$(H−C)	%−ile $\Delta\omega$(H−C)	$\Delta\omega$(C−N)	%−ile $\Delta\omega$(C−N)
HCTH/120	1	32.9	92.1	53.8	90.2	47.5	59.9	148.4	96.5
HCTH/120−D3(BJ)2B	1	32.3	92.3	53.3	91.7	46.5	52.2	147.9	99.0
HCTH/120−D3(BJ)ATM	1	32.1	92.5	53.1	91.5	46.5	51.8	147.8	99.2
HCTH/120−D3(0)2B	1	31.7	93.1	55.4	89.8	48.9	69.5	148.2	96.9
HCTH/120−D3(0)ATM	1	31.5	93.3	55.2	89.6	48.9	69.3	148.2	97.1
HCTH/147	1	34.4	90.2	51.3	87.1	48.9	68.9	150.7	90.4
HCTH/407	1	50.4	70.4	29.8	72.3	60.0	51.1	166.2	50.9
HCTH/407−D3(BJ)2B	1	51.9	68.8	26.5	73.1	58.3	63.7	168.1	47.2
HCTH/407−D3(BJ)ATM	1	52.1	68.5	26.3	73.3	58.3	63.5	168.1	47.0
HCTH/407−D3(0)2B	1	47.3	74.4	34.8	72.1	62.6	42.0	165.9	52.4
HCTH/407−D3(0)ATM	1	47.5	74.2	35.0	71.9	62.6	41.8	166.0	52.0
HCTH/407+	1	40.2	81.0	32.9	74.4	52.6	93.7	165.8	52.8
HCTH/93	1	31.9	90.6	47.5	84.8	45.5	46.3	151.6	88.9
HCTH−p(1/4)	1	79.8	50.4	22.9	63.5	68.8	28.4	171.4	37.8
HCTH−p(7/6)	1	0.0	41.3	82.3	95.6	30.7	12.7	149.3	94.8
KT2	1	25.6	61.9	92.5	76.0	4.1	1.3	95.1	3.5
M06−L	2	75.0	43.3	12.1	22.9	70.1	25.3	167.6	48.4
M06−L−D3(0)2B	2	75.4	43.1	12.5	22.5	70.1	25.7	167.6	48.2
M06−L−D3(0)ATM	2	75.2	43.5	12.3	22.7	70.1	25.5	167.6	48.6
M11−L	2	30.6	39.4	1.5	33.1	43.6	40.9	158.8	67.4
M11−L−D3(BJ)2B	2	31.0	39.0	1.0	33.3	43.6	41.3	158.8	67.2
M11−L−D3(BJ)ATM	2	30.8	39.2	1.3	33.5	43.6	41.1	158.8	67.6
M11−L−D3(0)2B	2	30.2	40.0	1.9	32.9	44.6	44.9	158.8	68.1
M11−L−D3(0)ATM	2	30.0	40.2	1.7	32.7	44.6	44.7	158.8	68.3
MGGA_MS0	2	57.7	90.4	88.1	87.3	−14.4	0.4	53.8	0.6
MGGA_MS1	2	40.4	81.5	88.3	85.8	−14.7	0.2	57.7	1.0
MGGA_MS2	2	34.2	79.2	87.7	80.6	−19.2	0.0	56.2	0.8
MGGA_MS2h	4	82.9	80.0	67.9	91.9	17.1	2.7	85.9	1.9
MN12−L	2	37.3	17.1	38.5	23.1	79.6	9.8	137.0	69.5
MN12−L−D3(BJ)2B	2	37.5	16.5	38.3	23.5	79.6	9.4	137.1	70.6
MN12−L−D3(BJ)ATM	2	37.7	16.7	38.1	23.3	79.6	9.6	137.1	70.4

Table S6 (continued)

Functional	Group	%−ile ω_1	%−ile ω_2	%−ile ω_3	%−ile ω_4	$\Delta\omega$(H−C)	%−ile $\Delta\omega$(H−C)	$\Delta\omega$(C−N)	%−ile $\Delta\omega$(C−N)
MN12−L−D3(0)2B	2	40.0	17.9	41.3	22.3	80.1	8.6	137.0	70.1
MN12−L−D3(0)ATM	2	39.8	18.5	41.5	22.1	80.3	8.4	137.0	69.3
MN15−L	2	91.3	52.5	32.3	41.9	56.9	73.5	140.6	81.0
MN15−L−D3(0)2B	2	91.0	52.3	32.7	41.9	56.9	73.3	140.6	81.2
MN15−L−D3(0)ATM	2	91.5	52.7	32.5	41.7	57.0	73.1	140.6	80.8
MOHLYP	1	0.4	36.5	89.6	70.4	21.8	4.6	117.1	26.1
MOHLYP2	1	0.6	10.0	77.1	54.8	0.8	1.0	92.5	2.9
MP2D	0	90.8	91.5	26.0	75.8	22.3	5.2	211.2	1.7
MP2	0	90.0	91.9	25.8	75.4	23.3	6.1	212.3	1.5
MPWLYP1M	3	29.0	82.9	87.3	84.2	53.2	99.6	114.1	22.5
MPWLYP1W	1	16.3	53.8	74.0	64.8	35.5	23.2	101.6	10.6
mPWPW	1	19.8	85.0	87.5	92.5	40.7	33.4	129.1	50.7
N12	1	97.1	71.9	56.7	81.0	57.5	70.1	147.3	98.5
N12−D3(BJ)2B	1	97.5	71.7	55.8	81.5	57.6	68.3	147.3	98.3
N12−D3(BJ)ATM	1	97.3	71.5	56.0	81.3	57.6	68.7	147.3	98.1
N12−D3(0)2B	1	92.1	74.6	60.8	80.2	60.3	50.5	147.3	98.7
N12−D3(0)ATM	1	90.6	75.2	61.0	80.4	59.9	53.4	147.1	96.7
oBLYP−D	1	13.8	57.9	82.1	71.3	37.8	29.0	107.3	16.1
oPBE−D	1	7.3	65.8	99.8	82.1	33.9	20.0	121.7	31.1
PBEOP	1	9.6	50.8	80.8	69.2	32.8	18.2	104.4	14.0
oPWLYP−D	1	11.9	56.3	79.6	70.2	37.1	26.7	106.4	15.7
oTPSS−D	1	16.9	89.2	79.2	93.1	18.4	3.5	113.8	22.3
PBE	1	17.5	87.5	85.6	95.2	43.1	38.6	134.8	62.6
PBE1W	1	19.2	79.0	91.9	89.4	40.7	33.6	125.4	40.9
PBE−D3(BJ)2B	1	18.3	87.7	85.4	94.4	43.4	40.5	135.1	63.0
PBE−D3(BJ)ATM	1	18.1	87.9	85.2	94.2	43.4	40.3	135.1	63.3
PBE−D3M(BJ)2B	1	17.9	88.3	84.6	93.3	43.3	39.7	135.3	64.5
PBE−D3M(BJ)ATM	1	18.5	88.1	84.8	93.5	43.4	40.1	135.2	64.3
PBE−D3M(0)2B	1	20.4	92.9	82.7	99.2	46.9	55.7	136.9	68.9
PBE−D3M(0)ATM	1	20.2	92.7	82.5	99.4	46.9	55.5	136.9	69.1
PBE−D3(0)2B	1	17.1	86.9	86.0	94.8	44.0	42.6	134.7	61.8

Table S6 (continued)

Functional	Group	%−ile ω_1	%−ile ω_2	%−ile ω_3	%−ile ω_4	$\Delta\omega$(H−C)	%−ile $\Delta\omega$(H−C)	$\Delta\omega$(C−N)	%−ile $\Delta\omega$(C−N)
PBE−D3(0)ATM	1	17.3	87.1	85.8	94.8	44.0	42.4	134.7	61.6
PBELYP1W	1	16.0	54.0	78.1	67.7	35.7	24.2	103.2	12.9
PBE−NL	1	16.5	88.5	84.2	92.7	41.9	36.3	135.9	65.8
PBEsol	1	15.8	94.6	54.4	90.8	53.6	97.1	156.3	76.4
PBEsol−D3(BJ)2B	1	15.6	94.4	54.0	91.3	53.6	96.5	156.3	76.6
PBEsol−D3(BJ)ATM	1	15.4	94.2	54.2	91.0	53.6	96.7	156.3	76.2
PBEsol−D3(0)2B	1	14.8	95.0	55.0	90.6	54.3	90.6	156.2	77.0
PBEsol−D3(0)ATM	1	15.0	94.8	54.8	90.4	54.4	90.4	156.2	76.8
PKZB	2	2.3	44.4	96.7	83.5	7.8	1.5	107.4	16.3
PKZB−D3(0)2B	2	1.3	40.6	97.9	74.0	−4.1	0.8	105.1	14.6
PKZB−D3(0)ATM	2	1.5	40.4	97.7	73.8	−4.1	0.6	105.1	15.0
PTPSS	6	84.6	99.2	67.1	79.8	39.9	31.7	138.8	75.4
PTPSS−D3(BJ)2B	6	87.1	99.6	66.3	77.9	40.5	33.2	139.2	77.5
PTPSS−D3(BJ)ATM	6	86.0	99.4	66.5	75.6	40.0	31.9	139.0	76.0
PTPSS−D3(0)2B	6	81.7	98.8	69.0	76.3	40.1	32.2	138.3	73.3
PTPSS−D3(0)ATM	6	81.9	99.0	69.8	80.8	40.5	33.0	138.6	74.3
PW86PBE	1	20.6	77.3	98.3	87.9	44.1	42.8	123.2	35.3
PW91	1	21.5	89.6	86.7	94.6	44.2	43.0	132.3	58.7
PW91−D3(BJ)2B	1	21.9	89.8	86.3	93.8	44.2	43.6	132.2	58.5
PW91−D3(BJ)ATM	1	21.7	89.4	86.5	93.8	44.2	43.4	132.2	58.2
revM06−L	2	46.0	7.5	8.1	13.1	79.9	8.8	144.1	89.1
revPBE	1	7.5	65.4	94.2	85.0	31.7	15.4	123.2	36.1
revPBE−D3(BJ)2B	1	8.5	66.3	92.9	83.8	31.2	13.4	123.1	34.9
revPBE−D3(BJ)ATM	1	8.8	66.5	93.5	84.0	31.2	13.6	123.2	35.1
revPBE−D3(0)2B	1	6.0	62.9	97.5	85.6	34.8	21.7	122.7	33.4
revPBE−D3(0)ATM	1	5.6	62.7	97.3	86.0	34.7	21.1	122.7	33.2
revPBE−NL	1	4.6	68.1	89.0	83.1	30.1	12.1	126.0	44.3
revSCAN	2	59.0	61.5	25.6	46.5	52.7	93.9	145.7	93.7
revTPSS	2	22.3	76.3	90.2	97.5	18.1	3.3	102.1	11.5
revTPSS−D3(BJ)2B	2	22.7	76.5	90.0	98.5	18.0	3.1	102.0	11.3
revTPSS−D3(BJ)ATM	2	22.5	76.7	89.8	98.3	17.9	2.9	102.2	11.7

Table S6 (continued)

Functional	Group	%−ile ω_1	%−ile ω_2	%−ile ω_3	%−ile ω_4	$\Delta\omega$(H−C)	%−ile $\Delta\omega$(H−C)	$\Delta\omega$(C−N)	%−ile $\Delta\omega$(C−N)
revTPSS−D3(0)2B	2	21.0	75.0	91.0	97.1	19.6	4.2	101.9	11.1
revTPSS−D3(0)ATM	2	21.3	74.8	91.3	96.9	19.6	4.4	101.9	10.9
revTPSSh	3	77.5	77.9	72.7	67.5	56.2	77.9	130.3	54.5
revTPSSh−D3(BJ)2B	3	77.9	77.7	71.9	68.3	56.1	78.1	130.2	53.9
revTPSSh−D3(BJ)ATM	3	77.7	77.5	72.1	68.1	56.1	78.3	130.2	54.1
revTPSSh−D3(0)2B	3	76.0	78.8	75.0	66.9	57.6	69.1	130.3	54.3
revTPSSh−D3(0)ATM	3	76.3	78.3	75.2	66.7	57.7	67.8	130.3	54.7
revTPSS−NL	2	19.6	77.1	88.8	99.8	16.9	2.5	103.0	12.7
RPBE	1	5.4	58.5	98.1	76.5	29.2	11.1	119.7	28.6
RPBE−D3(BJ)2B	1	6.3	60.6	94.6	74.6	26.8	9.0	121.2	30.7
RPBE−D3(BJ)ATM	1	6.5	60.8	94.4	74.8	26.8	9.2	121.2	30.9
RPBE−D3(0)2B	1	5.0	57.5	99.6	79.0	31.0	12.9	118.4	26.5
RPBE−D3(0)ATM	1	5.2	57.3	99.4	79.2	31.0	13.2	118.4	26.3
SCAN	2	37.9	85.4	52.9	77.1	22.2	4.8	112.5	19.8
SCAN−D3(BJ)2B	2	41.0	89.0	61.7	82.3	25.2	7.7	108.4	16.9
SCAN−D3(BJ)ATM	2	38.1	85.8	52.7	77.7	22.2	5.0	112.5	19.6
SCAN−D3(0)2B	2	38.5	86.0	52.3	76.9	22.4	5.6	112.5	20.0
SCAN−D3(0)ATM	2	38.3	85.4	52.5	76.7	22.4	5.4	112.5	20.3
SOGGA	1	14.0	91.3	46.3	85.2	55.5	80.6	163.2	58.0
SOGGA11	1	30.4	79.8	40.4	72.5	42.0	37.0	142.0	82.9
TH1	1	12.1	68.3	88.5	92.3	25.6	7.9	121.1	30.5
TH2	1	3.8	40.8	87.1	72.7	14.5	2.3	98.9	6.5
TH3	1	9.8	61.3	99.2	81.7	26.1	8.1	112.8	21.1
TH4	1	39.0	99.8	71.5	97.3	38.5	30.5	128.6	50.5
τ−HCTH	2	43.8	84.4	48.1	83.3	51.5	86.2	152.6	85.6
τ−HCTH−D3(BJ)2B	2	44.0	83.3	47.1	84.6	51.1	82.0	153.1	83.5
τ−HCTH−D3(BJ)ATM	2	44.2	83.1	46.9	84.4	51.1	82.3	153.1	83.3
τ−HCTH−D3(0)2B	2	41.5	86.3	50.8	82.5	54.2	92.1	152.5	86.2
τ−HCTH−D3(0)ATM	2	41.3	86.5	51.0	82.7	54.2	92.3	152.5	86.4
TH−FC	1	4.0	53.1	96.9	86.3	18.5	3.8	107.9	16.5
TH−FC+FO	1	4.2	52.9	97.1	86.5	18.5	4.0	107.9	16.7

Table S6 (continued)

Functional	Group	%−ile ω_1	%−ile ω_2	%−ile ω_3	%−ile ω_4	$\Delta\omega$(H−C)	%−ile $\Delta\omega$(H−C)	$\Delta\omega$(C−N)	%−ile $\Delta\omega$(C−N)
TH−FCO	1	4.4	66.0	90.8	92.9	24.7	6.9	116.2	25.7
TH−FL	1	22.9	59.4	21.7	50.6	66.8	31.3	169.5	43.0
TPSS	2	26.7	80.2	93.8	97.7	29.9	11.9	109.1	18.2
TPSS−D3(BJ)2B	2	26.9	80.4	93.3	99.0	29.9	11.5	109.1	18.0
TPSS−D3(BJ)ATM	2	27.1	80.6	92.7	98.8	29.9	11.7	109.1	17.7
TPSS−D3(0)2B	2	26.3	79.4	94.8	98.1	31.3	14.0	109.0	17.3
TPSS−D3(0)ATM	2	26.0	79.6	95.0	97.9	31.3	13.8	109.0	17.5
TPSSh−D3(BJ)2B	3	95.4	75.8	74.8	67.3	63.2	39.9	134.3	60.8
TPSSLYP1W	1	27.9	49.2	60.6	73.5	23.4	6.3	77.3	1.3
TPSS−NL	2	25.0	81.3	90.4	99.6	28.6	10.4	110.4	18.6
VSXC	2	61.9	96.5	62.9	72.9	41.0	34.0	126.2	45.7
VV10	1	24.6	72.3	94.0	78.1	41.9	36.7	116.5	25.9
XLYP	1	14.2	46.5	63.8	56.3	32.0	16.7	96.3	4.2
XLYP−D3(BJ)2B	1	14.4	46.9	64.8	55.2	31.7	15.0	96.8	4.6
XLYP−D3(BJ)ATM	1	14.6	47.1	64.6	55.4	31.7	15.2	96.8	5.0
XLYP−D3(0)2B	1	11.3	45.2	63.3	56.7	35.1	22.8	96.2	4.0
XLYP−D3(0)ATM	1	11.5	45.4	63.1	56.5	35.1	22.5	96.2	3.8
ZLP	1	41.9	95.2	85.0	96.7	nc	nc	nc	nc
B1LYP	3	79.0	56.0	71.7	49.0	52.9	96.2	115.3	24.4
B1LYP−D3(BJ)2B	3	77.1	55.8	71.0	49.6	53.5	98.3	115.7	25.1
B1LYP−D3(BJ)ATM	3	77.3	55.6	71.3	49.4	53.5	98.7	115.7	25.5
B1LYP−D3(0)2B	3	83.1	58.3	75.6	48.8	53.7	95.2	114.5	23.6
B1LYP−D3(0)ATM	3	83.3	58.1	75.4	48.5	53.7	95.4	114.5	23.8
B1PW91	3	60.6	29.0	23.5	32.5	57.7	68.1	140.6	80.6
B1WC	3	70.8	41.0	17.7	41.5	62.1	44.1	154.3	82.7
B2GP−PLYP	6	43.3	82.3	59.6	52.3	53.3	99.2	155.4	78.7
B2GP−PLYP−D3(BJ)2B	6	43.5	81.7	58.3	54.6	51.8	88.3	155.4	79.1
B2GP−PLYP−D3(BJ)ATM	6	43.1	82.1	58.8	55.0	53.5	98.5	155.5	78.3
B2GP−PLYP−D3(0)2B	6	45.2	82.5	59.8	54.4	54.6	88.5	156.6	74.7
B2GP−PLYP−D3(0)ATM	6	44.6	82.7	60.4	53.8	53.5	97.3	155.1	80.0
B2GP−PLYP−NL	6	44.4	81.9	58.1	55.8	53.2	99.8	155.8	77.9

Table S6 (continued)

Functional	Group	%-ile ω_1	%-ile ω_2	%-ile ω_3	%-ile ω_4	$\Delta\omega$(H-C)	%-ile $\Delta\omega$(H-C)	$\Delta\omega$(C-N)	%-ile $\Delta\omega$(C-N)
B3LYP	3	97.7	64.4	74.2	59.6	55.3	82.5	120.2	29.4
B3LYP5	3	92.7	67.1	76.5	62.1	55.4	81.6	120.0	28.8
B3LYP-D3(BJ)2B	3	98.8	64.0	73.3	60.2	55.9	79.3	120.6	29.6
B3LYP-D3(BJ)ATM	3	99.0	63.8	73.1	60.0	55.9	79.5	120.6	29.9
B3LYP-D3M(BJ)2B	3	97.9	63.5	72.3	61.0	56.0	78.9	120.8	30.1
B3LYP-D3M(BJ)ATM	3	98.1	63.3	72.5	60.8	56.0	78.9	120.8	30.3
B3LYP-D3M(0)2B	3	89.6	67.9	78.5	58.5	56.3	76.6	118.5	26.7
B3LYP-D3M(0)ATM	3	89.4	67.7	78.3	58.3	56.3	76.8	118.5	26.9
B3LYP-D3(0)2B	3	92.5	67.3	77.9	59.0	56.4	76.2	119.6	28.2
B3LYP-D3(0)ATM	3	92.3	67.5	77.7	58.8	56.4	76.4	119.6	28.0
B3LYP-NL	3	89.8	63.1	69.6	59.8	55.0	86.0	122.1	32.2
B3LYPS	3	62.7	78.1	83.8	74.2	58.0	65.3	124.3	38.4
B3P86	3	94.4	47.9	44.0	44.8	58.9	61.0	138.6	74.1
B3P86-D3(BJ)2B	3	95.8	47.7	43.3	45.4	59.3	57.8	138.8	74.9
B3P86-D3(BJ)ATM	3	95.6	47.5	43.5	45.2	59.3	58.2	138.8	75.2
B3P86-D3(0)2B	3	90.4	49.8	45.4	44.6	60.0	52.0	138.3	72.9
B3P86-D3(0)ATM	3	90.2	49.6	45.2	44.4	59.9	52.4	138.3	73.1
B3PW91	3	87.3	44.8	37.1	42.9	59.3	58.5	140.6	80.4
B3PW91-D3(BJ)2B	3	84.2	43.8	36.7	43.5	60.0	51.6	141.0	82.5
B3PW91-D3(BJ)ATM	3	84.0	44.0	36.9	43.3	60.0	51.4	141.0	82.3
B3PW91-D3(0)2B	3	91.7	46.0	37.9	42.7	60.6	49.1	140.1	79.7
B3PW91-D3(0)ATM	3	91.9	46.3	37.7	42.5	60.6	49.3	140.1	79.3
B3PW91-NL	3	94.6	42.9	32.1	44.0	58.9	61.2	142.6	84.6
B5050LYP	3	20.8	3.3	11.9	5.4	42.6	37.6	112.2	19.4
B86B95	3	61.3	35.4	28.5	33.8	57.9	66.4	135.1	63.5
B88B95	3	60.2	30.4	23.1	25.0	56.7	75.4	135.8	65.3
B88B95-D3(BJ)2B	3	59.2	29.6	21.9	25.4	57.1	71.8	136.1	66.2
B88B95-D3(BJ)ATM	3	59.4	29.4	22.1	25.2	57.1	72.0	136.1	66.4
B88B95-D3(0)2B	3	63.8	32.1	25.2	24.8	57.2	71.4	135.1	63.9
B88B95-D3(0)ATM	3	63.5	32.3	25.4	24.6	57.2	71.2	135.1	63.7
B97-0	3	82.3	65.2	66.9	67.9	58.9	60.8	132.9	59.1

Table S6 (continued)

Functional	Group	%−ile ω_1	%−ile ω_2	%−ile ω_3	%−ile ω_4	$\Delta\omega$(H−C)	%−ile $\Delta\omega$(H−C)	$\Delta\omega$(C−N)	%−ile $\Delta\omega$(C−N)
B97−1	3	87.7	62.5	67.7	62.9	57.2	71.6	130.1	53.4
B97−1−D3(BJ)2B	3	87.9	62.3	67.3	64.2	57.5	70.6	130.3	54.9
B97−1−D3(BJ)ATM	3	88.1	62.1	67.5	64.0	57.5	70.8	130.3	55.1
B97−1−D3(0)2B	3	81.5	64.8	70.6	62.5	57.7	67.4	129.4	51.6
B97−1−D3(0)ATM	3	81.3	64.6	70.8	62.3	57.7	67.6	129.4	51.8
B97−1P	3	86.9	64.2	62.7	69.4	63.5	38.0	143.4	87.3
B97−2	3	51.0	33.3	23.8	38.8	62.6	42.2	148.7	95.6
B97−2−D3(BJ)2B	3	49.4	31.3	20.8	41.0	63.2	39.2	149.9	93.3
B97−2−D3(BJ)ATM	3	49.6	31.5	20.6	40.8	63.2	39.5	149.9	93.5
B97−2−D3(0)2B	3	55.6	35.8	26.7	38.5	63.5	38.2	147.7	99.8
B97−2−D3(0)ATM	3	55.8	35.6	26.9	38.3	63.5	38.4	147.7	99.6
B97−3	3	56.0	38.1	42.3	41.3	55.1	84.1	129.6	53.0
B97−K	3	49.8	41.5	84.0	39.6	47.0	56.2	106.1	15.2
BB1K	3	28.8	6.9	6.3	7.3	50.4	78.5	131.5	57.4
BHandH	3	33.5	2.5	0.6	2.7	43.8	41.5	133.6	60.5
BHandHLYP	3	22.1	5.4	19.8	7.1	42.6	37.4	109.2	18.4
BMK	4	93.1	22.5	57.7	20.0	45.3	45.9	115.3	24.2
BMK−D3(BJ)2B	4	94.0	22.1	57.1	20.4	45.6	47.0	115.5	24.8
BMK−D3(BJ)ATM	4	93.8	22.3	56.9	20.2	45.6	46.8	115.5	24.6
BMK−D3(0)2B	4	86.5	25.0	61.5	19.6	46.0	49.7	114.5	23.2
BMK−D3(0)ATM	4	86.7	24.8	61.3	19.4	46.0	49.9	114.5	23.4
CAM−B3LYP	5	86.3	33.1	48.8	36.5	46.3	50.7	113.1	21.7
CAM−B3LYP−D3(BJ)2B	5	85.2	32.7	48.5	36.9	46.6	53.2	113.3	22.1
CAM−B3LYP−D3(BJ)ATM	5	85.4	32.9	48.3	36.7	46.6	53.0	113.3	21.9
CAM−B3LYP−D3(0)2B	5	88.8	34.4	50.0	36.0	46.8	55.3	112.7	20.7
CAM−B3LYP−D3(0)ATM	5	89.0	34.6	50.2	35.8	46.8	55.1	112.7	20.5
CAM−LDA0	0	87.5	7.1	2.7	7.9	48.6	67.2	139.8	78.1
CAP0	3	78.1	26.3	16.3	26.5	58.4	62.8	146.0	94.6
DLDF	4	29.2	20.2	96.3	14.6	52.8	96.0	97.3	6.3
DLDF+D09	4	29.4	20.4	96.5	14.8	52.8	95.8	97.2	6.1
DLDF+D10	4	29.6	20.6	96.0	15.2	52.8	95.0	97.2	5.8

Table S6 (continued)

Functional	Group	%−ile ω_1	%−ile ω_2	%−ile ω_3	%−ile ω_4	$\Delta\omega$(H−C)	%−ile $\Delta\omega$(H−C)	$\Delta\omega$(C−N)	%−ile $\Delta\omega$(C−N)
DSD−PBEB95	6	47.1	76.9	41.7	47.1	56.0	78.7	167.8	47.4
DSD−PBEB95−D3(BJ)	6	49.2	70.2	45.0	46.9	54.4	89.6	159.0	66.6
DSD−PBEB95−NL	6	51.3	73.1	46.0	47.3	55.0	86.6	158.8	67.0
EDF2	3	92.9	49.0	47.3	46.7	56.4	76.0	131.0	56.4
HF	0	2.5	1.5	49.6	0.6	30.7	12.5	89.7	2.7
HF−3c	0	2.1	0.0	0.0	0.0	38.4	30.3	155.3	79.5
HF+D	0	2.7	1.7	49.8	0.8	30.7	12.3	89.7	2.5
HF−D3(BJ)2B	0	1.7	0.6	40.0	1.0	31.5	14.2	94.9	3.3
HF−D3(BJ)ATM	0	1.9	0.8	40.2	1.3	31.5	14.4	94.8	3.1
HF−D3M(BJ)2B	0	0.8	0.2	31.5	1.7	31.9	15.9	99.8	8.1
HF−D3M(BJ)ATM	0	1.0	0.4	31.7	1.5	31.9	15.7	99.8	7.9
HF−D3M(0)2B	0	2.9	1.0	50.4	2.1	32.2	17.3	99.6	7.5
HF−D3M(0)ATM	0	3.1	1.3	50.6	1.9	32.2	17.1	99.6	7.3
HF−D3(0)2B	0	3.3	1.9	51.5	0.2	32.0	16.5	89.3	2.1
HF−D3(0)ATM	0	3.5	2.1	51.7	0.2	32.0	16.3	89.3	2.3
HJS−B97x	5	88.5	12.5	6.7	18.1	63.0	40.7	163.6	57.8
HJS−PBE	5	72.3	28.3	17.5	30.4	59.4	56.8	145.0	91.9
HJS−PBEsol	5	99.2	11.3	5.6	17.5	60.8	47.6	160.2	64.1
hPBEint	3	54.6	45.0	20.0	43.1	65.0	35.1	158.9	66.8
HSE03	5	51.5	20.8	15.8	26.9	60.3	50.3	144.8	90.8
HSE03−D3(BJ)2B	5	53.3	21.9	15.0	26.3	58.8	61.8	143.5	87.5
HSE03−D3(BJ)ATM	5	53.5	21.7	15.2	26.0	58.8	61.6	143.5	87.7
HSE03−D3(0)2B	5	56.7	23.1	17.3	25.8	59.0	60.1	143.0	86.8
HSE03−D3(0)ATM	5	56.9	23.3	17.1	25.6	59.0	60.3	143.0	86.6
HSE06	5	72.7	29.2	19.4	34.0	57.5	69.7	142.7	84.8
HSE06−D3(BJ)2B	5	66.5	28.1	17.9	29.2	59.2	58.7	143.6	87.9
HSE06−D3(BJ)ATM	5	66.7	27.9	18.1	29.0	59.2	59.1	143.7	88.1
HSE06−D3(0)2B	5	68.1	28.5	18.3	29.6	59.7	54.5	143.7	88.3
HSE06−D3(0)ATM	5	68.3	28.8	18.5	29.4	59.7	54.7	143.7	88.5
KMLYP	3	16.7	2.3	0.4	2.3	41.3	34.7	127.6	48.0
KSDT	0	17.7	65.6	27.5	69.0	67.5	30.7	170.2	40.7

Table S6 (continued)

Functional	Group	%-ile ω_1	%-ile ω_2	%-ile ω_3	%-ile ω_4	$\Delta\omega$(H-C)	%-ile $\Delta\omega$(H-C)	$\Delta\omega$(C-N)	%-ile $\Delta\omega$(C-N)
LC-BOP	1	61.0	6.7	10.6	3.8	37.0	26.5	106.4	15.4
LC-VV10	1	45.4	6.3	6.5	6.0	41.7	35.5	122.2	32.8
LDA0	0	52.3	16.0	7.1	17.7	54.3	90.8	146.2	95.0
LRC-ωPBE	5	55.4	42.7	31.9	43.8	53.3	99.4	137.6	71.0
LRC-ωPBEh	5	85.6	19.4	12.7	24.0	55.5	80.4	143.2	87.1
M05	4	60.8	12.7	3.5	12.1	74.5	16.1	192.8	12.1
M05-2X	4	42.7	14.8	27.9	15.0	34.2	20.5	103.9	13.6
M05-2X-D3(0)2B	4	42.3	14.6	27.3	16.0	34.5	20.9	104.1	13.8
M05-2X-D3(0)ATM	4	42.9	15.0	27.7	14.4	34.2	20.3	103.8	13.4
M05-D3(0)2B	4	61.5	13.1	4.0	11.9	74.8	14.6	192.5	12.5
M05-D3(0)ATM	4	61.7	12.9	4.2	11.7	74.8	14.8	192.5	12.3
M06	4	85.8	19.2	8.3	13.3	68.7	28.6	164.0	57.2
M06-2X	4	74.0	21.0	40.6	12.7	44.9	45.7	112.8	21.5
M06-2X-D3(0)2B	4	75.6	21.5	40.8	12.9	44.4	43.8	112.7	20.9
M06-2X-D3(0)ATM	4	74.4	21.3	41.0	12.5	44.8	45.1	112.8	21.3
M06-D3(0)2B	4	85.0	19.0	7.9	13.8	69.0	27.1	164.0	57.0
M06-D3(0)ATM	4	84.8	18.8	7.7	13.5	69.0	26.9	164.0	56.8
M06-HF	4	54.8	13.3	99.0	3.3	12.3	2.1	48.8	0.4
M06-HF-D3(0)2B	4	55.2	13.5	98.5	3.1	12.2	1.7	48.7	0.2
M06-HF-D3(0)ATM	4	55.0	13.8	98.5	2.9	12.2	1.9	48.7	0.0
M08-HX	4	80.2	16.9	33.5	8.5	46.7	54.9	105.1	14.8
M08-HX-D3(0)2B	4	80.4	17.5	34.0	8.3	46.6	53.9	105.1	14.2
M08-HX-D3(0)ATM	4	80.6	17.3	33.8	8.1	46.6	53.7	105.1	14.4
M08-SO	4	53.8	41.7	49.4	17.9	42.8	37.8	111.4	19.2
M11	5	58.3	37.3	30.2	9.2	32.7	17.5	97.0	5.4
M11-D3(BJ)2B	5	58.1	37.7	30.8	9.6	32.7	18.0	97.0	5.2
M11-D3(BJ)ATM	5	57.9	37.5	30.4	9.2	32.7	17.7	97.0	5.6
M11-D3(0)2B	5	57.3	38.3	31.0	9.0	32.9	18.6	96.8	4.8
M11-D3(0)ATM	5	57.1	38.5	31.3	8.8	32.9	18.4	96.8	4.4
mB3LYP-RC04	3	48.3	46.7	57.9	47.7	54.3	91.6	123.6	37.2
MGGA-MVS	2	64.4	7.9	2.1	6.9	76.9	11.3	194.9	9.0

Table S6 (continued)

Functional	Group	%−ile ω_1	%−ile ω_2	%−ile ω_3	%−ile ω_4	$\Delta\omega$(H−C)	%−ile $\Delta\omega$(H−C)	$\Delta\omega$(C−N)	%−ile $\Delta\omega$(C−N)
MGGA−MVSh	4	26.5	2.7	0.2	2.5	60.7	47.8	176.0	27.6
MN12−L	2	35.4	14.0	39.6	21.5	81.8	7.3	138.6	73.7
MN12−L−D3(BJ)2B	2	35.4	14.0	39.6	21.5	81.8	7.1	138.6	73.9
MN12−L−D3(BJ)ATM	2	35.8	14.4	39.4	21.5	81.7	7.5	138.6	73.5
MN12−L−D3(0)2B	2	37.1	16.3	42.1	23.8	82.0	6.7	138.2	72.4
MN12−L−D3(0)ATM	2	36.9	15.6	41.9	21.3	82.0	6.5	138.3	72.7
MN12−SX	5	54.4	25.8	59.0	40.2	68.6	29.9	126.1	44.5
MN12−SX−D3(BJ)2B	5	54.0	25.4	59.2	40.6	68.6	29.4	126.1	45.3
MN12−SX−D3(BJ)ATM	5	54.2	25.6	59.4	40.4	68.6	29.6	126.1	44.7
MN12−SX−D3(0)2B	5	56.3	27.7	60.0	40.0	68.9	27.3	125.7	41.8
MN12−SX−D3(0)ATM	5	56.5	27.3	60.2	39.8	68.9	27.6	125.8	42.0
MN15	5	59.8	42.5	38.8	35.4	41.8	35.9	122.0	31.5
MN15−D3(BJ)2B	5	59.6	42.3	39.0	35.6	41.8	36.1	122.0	31.7
MN15−D3(BJ)ATM	5	60.0	42.1	39.2	35.2	41.8	35.7	122.1	31.9
MPW1B95	3	48.1	15.8	13.3	18.8	55.1	84.3	134.8	62.0
MPW1B95−D3(BJ)2B	3	47.7	15.4	12.9	19.2	55.2	83.1	134.8	62.2
MPW1B95−D3(BJ)ATM	3	47.9	15.2	13.1	19.0	55.2	83.3	134.8	62.4
MPW1B95−D3(0)2B	3	50.2	18.3	14.0	18.5	55.4	81.8	134.3	61.0
MPW1B95−D3(0)ATM	3	50.0	18.1	14.4	18.3	55.4	81.4	134.3	61.2
MPW1K	3	25.4	3.1	2.9	5.2	49.7	74.7	134.9	62.8
mPW1LYP	3	78.3	55.0	70.4	48.3	52.8	94.4	115.2	24.0
mPW1LYP−D3(0)2B	3	82.5	57.1	73.8	48.1	53.5	97.9	114.4	23.0
mPW1LYP−D3(0)ATM	3	82.7	56.9	73.5	47.9	53.5	98.1	114.4	22.8
mPW1PBE	3	62.9	26.0	19.0	29.8	58.1	64.5	142.8	85.4
mPW1PW	3	60.4	27.5	21.5	31.9	57.5	69.9	140.5	80.2
mPW1PW−D3(BJ)2B	3	58.8	26.9	20.4	32.3	58.0	65.8	140.7	82.0
mPW1PW−D3(BJ)ATM	3	58.5	27.1	20.2	32.1	58.0	65.6	140.7	81.6
mPW1PW−D3(0)2B	3	62.1	30.0	24.0	31.7	58.5	62.2	140.0	78.9
mPW1PW−D3(0)ATM	3	62.3	30.2	24.2	31.5	58.4	62.4	140.0	78.5
MPW3LYP	3	94.2	59.2	66.0	52.7	54.3	91.0	119.5	27.8
mPW3PW	3	80.8	41.9	34.2	42.3	59.1	59.7	140.7	81.8

Table S6 (continued)

Functional	Group	%−ile ω_1	%−ile ω_2	%−ile ω_3	%−ile ω_4	$\Delta\omega$(H−C)	%−ile $\Delta\omega$(H−C)	$\Delta\omega$(C−N)	%−ile $\Delta\omega$(C−N)
MPWB1K	3	27.7	5.2	3.8	6.3	49.5	72.9	130.8	55.7
MPWB1K−D3(BJ)2B	3	27.5	4.8	3.1	6.7	49.6	74.3	130.9	56.2
MPWB1K−D3(BJ)ATM	3	27.3	5.0	3.3	6.5	49.6	74.5	130.9	55.9
MPWB1K−D3(0)2B	3	28.1	5.8	4.4	5.8	49.8	75.2	130.4	55.5
MPWB1K−D3(0)ATM	3	28.3	5.6	4.6	5.6	49.8	74.9	130.4	55.3
N12−SX	5	45.6	31.0	23.3	30.8	49.2	71.0	126.6	45.9
N12−SX−D3(BJ)2B	5	44.8	30.6	22.7	31.3	49.5	72.4	126.7	46.3
N12−SX−D3(BJ)ATM	5	44.8	30.8	22.5	31.0	49.5	72.7	126.7	46.6
N12−SX−D3(0)2B	5	46.7	31.7	25.0	30.2	49.6	74.1	126.0	44.1
N12−SX−D3(0)ATM	5	46.9	31.9	24.8	30.0	49.6	73.9	126.0	43.8
O3LYP	3	96.5	54.8	45.6	57.7	71.1	23.0	158.0	70.8
O3LYP−D3(BJ)2B	3	95.0	54.6	44.8	58.1	71.5	22.1	158.4	69.7
O3LYP−D3(BJ)ATM	3	95.2	54.4	44.6	57.9	71.5	22.3	158.4	69.9
O3LYP−D3(0)2B	3	96.9	56.5	46.5	57.5	71.7	21.3	157.3	72.0
O3LYP−D3(0)ATM	3	96.7	56.7	46.7	57.3	71.7	21.5	157.3	72.2
PBE0	3	71.9	25.2	16.0	27.7	59.1	59.5	145.1	92.1
PBE0−1/3	3	42.1	7.7	7.3	12.3	55.1	85.6	142.1	83.1
PBE0−2	6	29.8	49.4	11.7	28.8	54.4	89.8	179.7	25.3
PBE0−D3(BJ)2B	3	70.4	24.4	15.4	28.1	59.4	57.2	145.3	92.7
PBE0−D3(BJ)ATM	3	70.2	24.6	15.6	27.9	59.4	57.0	145.3	92.3
PBE0−D3M(BJ)2B	3	69.6	23.8	14.6	28.5	59.4	56.6	145.4	93.1
PBE0−D3M(BJ)ATM	3	69.6	24.0	14.8	28.3	59.4	56.4	145.4	92.9
PBE0−D3M(0)2B	3	64.8	22.7	13.8	24.4	59.4	57.4	145.0	91.6
PBE0−D3M(0)ATM	3	65.0	22.9	13.5	24.2	59.4	57.6	145.0	91.4
PBE0−D3(0)2B	3	72.9	26.5	16.7	27.3	59.8	54.1	144.8	91.0
PBE0−D3(0)ATM	3	73.1	26.7	16.9	27.1	59.8	54.3	144.8	90.6
PBE0−DH	6	33.3	11.9	8.8	15.4	54.2	91.9	152.8	84.3
PBE0−DH−D3(BJ)2B	6	33.1	11.5	7.5	15.8	54.1	92.7	154.7	81.4
PBE0−DH−D3(BJ)ATM	6	32.7	11.7	8.5	15.6	54.5	89.1	153.1	83.7
PBE0−DH−D3(0)2B	6	33.8	12.1	9.0	14.2	55.6	80.2	152.9	83.9
PBE0−DH−D3(0)ATM	6	34.0	12.3	9.2	14.0	54.6	88.7	152.6	85.8

Table S6 (continued)

Functional	Group	%−ile ω_1	%−ile ω_2	%−ile ω_3	%−ile ω_4	$\Delta\omega$(H−C)	%−ile $\Delta\omega$(H−C)	$\Delta\omega$(C−N)	%−ile $\Delta\omega$(C−N)
PBE0−NL	3	74.8	24.2	14.2	30.6	58.8	61.4	145.9	93.9
PBE50	3	20.0	2.9	0.8	3.5	48.2	63.9	136.7	68.5
PBEh−3c	3	32.5	3.5	2.3	4.0	48.6	66.6	136.6	67.8
PW6B95	4	49.0	35.2	36.0	34.6	54.9	86.8	128.0	49.5
PW6B95−D3(BJ)2B	4	48.5	35.0	35.6	35.0	55.1	85.2	128.1	49.9
PW6B95−D3(BJ)ATM	4	48.8	34.8	35.8	34.8	55.1	85.0	128.1	50.1
PW6B95−D3(0)2B	4	50.6	36.0	36.3	34.4	55.1	83.9	127.6	47.8
PW6B95−D3(0)ATM	4	50.8	36.3	36.5	34.2	55.1	84.6	127.6	47.6
PW86B95	3	51.7	33.8	30.6	27.5	56.2	77.7	129.4	52.2
PWB6K	3	23.5	4.2	5.2	4.6	48.1	62.6	126.1	44.9
PWB6K−D3(BJ)2B	3	23.1	3.8	4.8	5.0	48.1	63.3	126.1	45.5
PWB6K−D3(BJ)ATM	3	23.3	4.0	5.0	4.8	48.1	63.0	126.1	45.1
PWB6K−D3(0)2B	3	24.0	4.6	5.8	4.4	48.2	64.3	125.9	42.8
PWB6K−D3(0)ATM	3	24.2	4.4	6.0	4.2	48.2	64.1	125.9	43.2
PWPB95	6	63.1	69.4	56.5	52.1	54.9	87.1	144.5	90.0
PWPB95−D3(BJ)2B	6	65.6	69.6	58.5	51.0	55.1	83.7	145.3	92.5
PWPB95−D3(BJ)ATM	6	62.5	69.2	56.3	51.9	55.0	85.8	144.6	90.2
PWPB95−D3(0)2B	6	64.0	69.8	57.3	51.5	54.8	88.1	142.9	86.0
PWPB95−D3(0)ATM	6	64.6	70.0	57.5	51.3	55.1	84.8	144.3	89.6
PWPB95−NL	6	64.2	69.0	55.6	53.5	54.8	87.9	144.9	91.2
revB3LYP	3	88.3	60.4	64.4	55.6	55.4	81.2	122.9	33.8
revPBE0	3	71.5	37.9	29.6	38.1	60.9	46.6	143.7	88.7
revPBE0−D3(BJ)2B	3	65.2	37.1	29.2	39.4	61.7	45.3	144.3	89.4
revPBE0−D3(BJ)ATM	3	65.4	36.9	29.4	39.2	61.7	45.5	144.3	89.8
revPBE0−D3(0)2B	3	76.7	39.6	33.3	37.9	62.1	44.3	142.8	85.2
revPBE0−D3(0)ATM	3	76.9	39.8	33.1	37.7	62.1	44.5	142.7	85.0
revPBE0−NL	3	76.5	36.7	27.1	39.0	60.5	50.1	145.9	94.4
revSCAN0	4	31.3	6.5	2.5	7.5	66.1	32.8	162.4	59.3
SB98−1a	3	46.3	29.8	35.2	37.1	52.0	89.4	124.0	38.0
SB98−1b	3	66.9	55.2	42.5	45.0	64.6	36.5	146.8	96.0
SB98−1c	3	83.8	65.0	65.6	67.1	59.2	58.9	133.3	59.9

Table S6 (continued)

Functional	Group	%−ile ω_1	%−ile ω_2	%−ile ω_3	%−ile ω_4	$\Delta\omega$(H−C)	%−ile $\Delta\omega$(H−C)	$\Delta\omega$(C−N)	%−ile $\Delta\omega$(C−N)
SB98−2a	3	67.7	53.5	64.2	51.7	52.6	93.1	122.2	32.6
SB98−2b	3	80.0	55.4	62.5	47.5	54.4	90.0	127.1	46.8
SB98−2c	3	89.2	59.8	65.8	56.9	54.9	87.5	126.6	46.1
SCAN0	4	34.6	7.3	10.4	17.3	52.6	93.5	142.5	84.1
SOGGA11−X	3	35.2	10.6	19.6	16.7	57.5	70.4	138.7	74.5
SOGGA11−X−D3(BJ)2B	3	34.8	10.4	19.2	17.1	57.9	66.8	139.0	75.6
SOGGA11−X−D3(BJ)ATM	3	35.0	10.2	18.8	16.9	57.9	67.0	139.0	75.8
SOGGA11−X−D3(0)2B	3	39.6	10.8	21.0	16.5	58.0	66.2	138.0	71.8
SOGGA11−X−D3(0)ATM	3	39.4	11.0	21.3	16.3	58.0	66.0	138.0	71.6
SPW92	0	19.4	61.0	24.6	62.7	67.3	30.9	170.8	38.8
SVWN	0	24.8	54.2	16.5	56.0	66.7	31.5	172.2	35.7
Teter93	0	18.8	61.7	24.4	61.3	67.2	31.1	170.7	39.0
τ−HCTHh	4	75.8	66.7	63.5	71.7	60.1	50.9	139.4	77.7
TH−FL	1	24.4	57.7	22.3	50.4	68.8	27.8	170.6	39.2
TPSSh	3	98.5	72.9	77.5	65.8	65.2	34.4	135.7	65.1
TPSSh−D3(BJ)2B	3	99.8	72.5	76.0	66.5	65.3	34.2	135.9	65.6
TPSSh−D3(BJ)ATM	3	98.3	72.1	76.9	66.3	65.7	33.8	135.9	66.0
TPSSh−D3(0)2B	3	96.3	75.4	78.8	65.6	66.2	32.4	135.3	64.9
TPSSh−D3(0)ATM	3	96.0	75.6	79.0	65.4	66.2	32.6	135.3	64.7
tuned−CAM−B3LYP	5	41.7	80.8	77.3	82.9	57.0	72.2	128.0	49.7
ωB97	1	71.3	17.7	30.0	19.8	51.3	83.5	129.3	51.4
ωB97M−D3(BJ)	5	79.4	44.2	65.0	46.0	46.5	52.6	111.0	19.0
ωB97M−V	5	84.4	44.6	64.0	46.3	47.8	62.0	110.9	18.8
ωB97X	5	67.9	19.6	28.1	20.6	49.6	73.7	125.9	42.6
ωB97X−D	5	78.8	34.0	35.4	36.3	51.4	85.4	127.9	49.1
ωB97X−D3	5	72.1	23.5	29.0	26.7	53.0	99.0	129.5	52.6
ωB97X−D3(BJ)	5	79.2	32.5	49.2	37.3	49.9	75.6	122.3	33.0
ωB97X−D3(0)2B	5	67.5	20.0	28.8	20.8	50.3	77.2	126.0	43.4
ωB97X−D3(0)ATM	5	67.3	19.8	28.3	20.8	50.3	77.5	126.0	43.6
ωB97X−V	5	83.5	33.5	49.0	37.5	50.6	79.7	122.0	31.3
ωPBE	5	71.0	9.0	10.2	10.6	45.3	46.1	125.6	41.1

Table S6 (continued)

Functional	Group	%−ile ω_1	%−ile ω_2	%−ile ω_3	%−ile ω_4	$\Delta\omega$(H−C)	%−ile $\Delta\omega$(H−C)	$\Delta\omega$(C−N)	%−ile $\Delta\omega$(C−N)
ωPBE0	5	42.5	6.0	5.4	7.7	47.1	58.0	133.0	59.7
ωPBE−D3(BJ)2B	5	69.0	8.5	10.0	11.0	45.6	47.4	125.7	41.5
ωPBE−D3(BJ)ATM	5	69.2	8.8	9.8	10.8	45.6	47.2	125.7	41.3
ωPBE−D3M(BJ)2B	5	66.0	8.3	9.4	11.5	45.8	48.0	125.8	42.2
ωPBE−D3M(BJ)ATM	5	65.8	8.1	9.6	11.3	45.8	48.2	125.8	42.4
ωPBE−D3M(0)2B	5	74.2	9.6	11.5	10.0	45.9	49.5	124.9	39.5
ωPBE−D3M(0)ATM	5	74.6	9.8	11.3	9.8	45.9	48.9	124.9	39.7
ωPBE−D3(0)2B	5	73.8	9.2	11.0	10.4	45.9	48.4	125.1	40.5
ωPBE−D3(0)ATM	5	73.5	9.4	10.8	10.2	45.9	48.6	125.1	40.1
X3LYP	3	94.8	59.6	66.7	53.3	54.4	90.2	119.6	28.4
X3LYP−D3(BJ)2B	3	93.3	59.0	65.4	54.2	54.9	87.3	120.0	29.0
X3LYP−D3(BJ)ATM	3	93.5	58.8	65.2	54.0	54.9	87.7	120.0	29.2
X3LYP−D3(0)2B	3	99.6	60.2	69.2	53.1	55.3	82.9	119.3	27.1
X3LYP−D3(0)ATM	3	99.4	60.0	69.4	52.9	55.3	82.7	119.3	27.3
XB1K	3	0.2	38.8	43.8	96.3	59.5	55.9	108.7	17.1

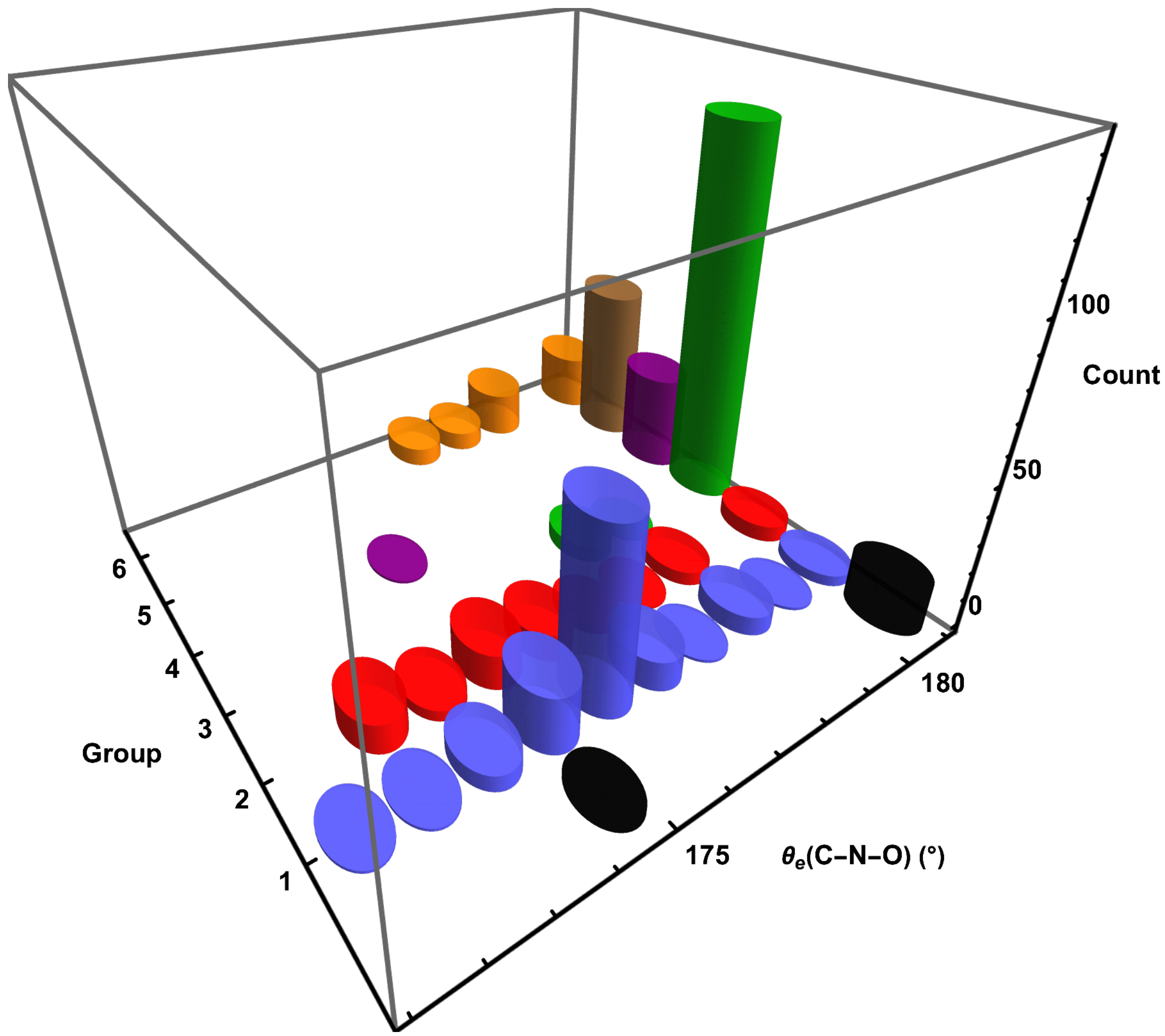

Figure S1. Histogram of fulminic acid θ_e(C–N–O) values predicted by each group of DFAs.

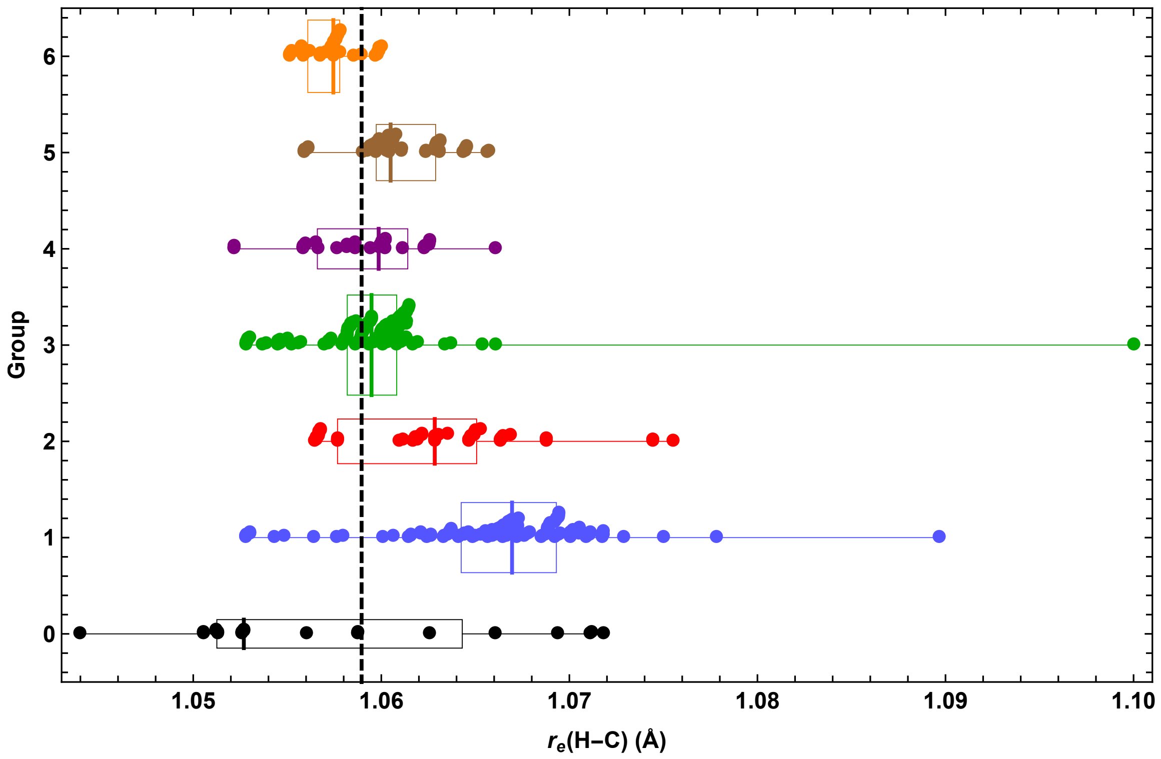

Figure S2. Scatter plot of DFA predictions for r_e(H–C) sorted by group hierarchy. For each group, a box and whisker plot shows the maximum, minimum, quartiles, and median of the data set. The vertical dotted line identifies the essentially exact r_e value (ref. 74).

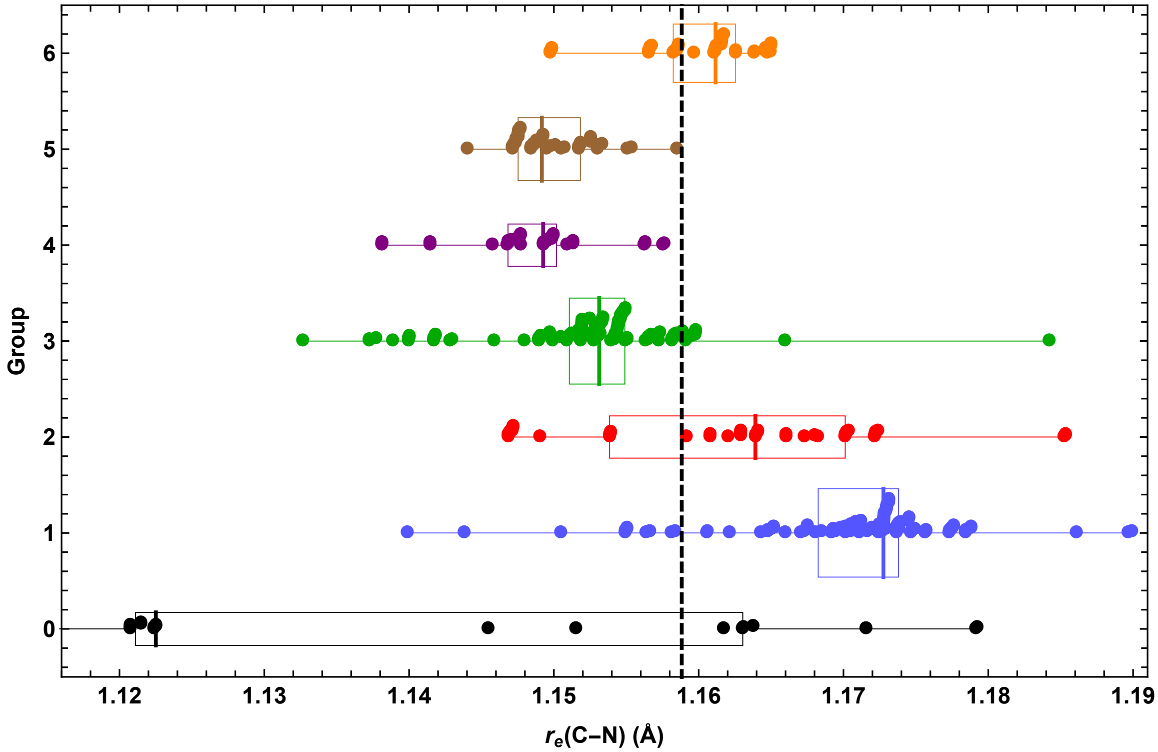

Figure S3. Scatter plot of fulminic acid r_e(C–N) predictions for each DFA group, overlaid with box and whisker representations of the data. See the caption of Figure S2 for further explanation.

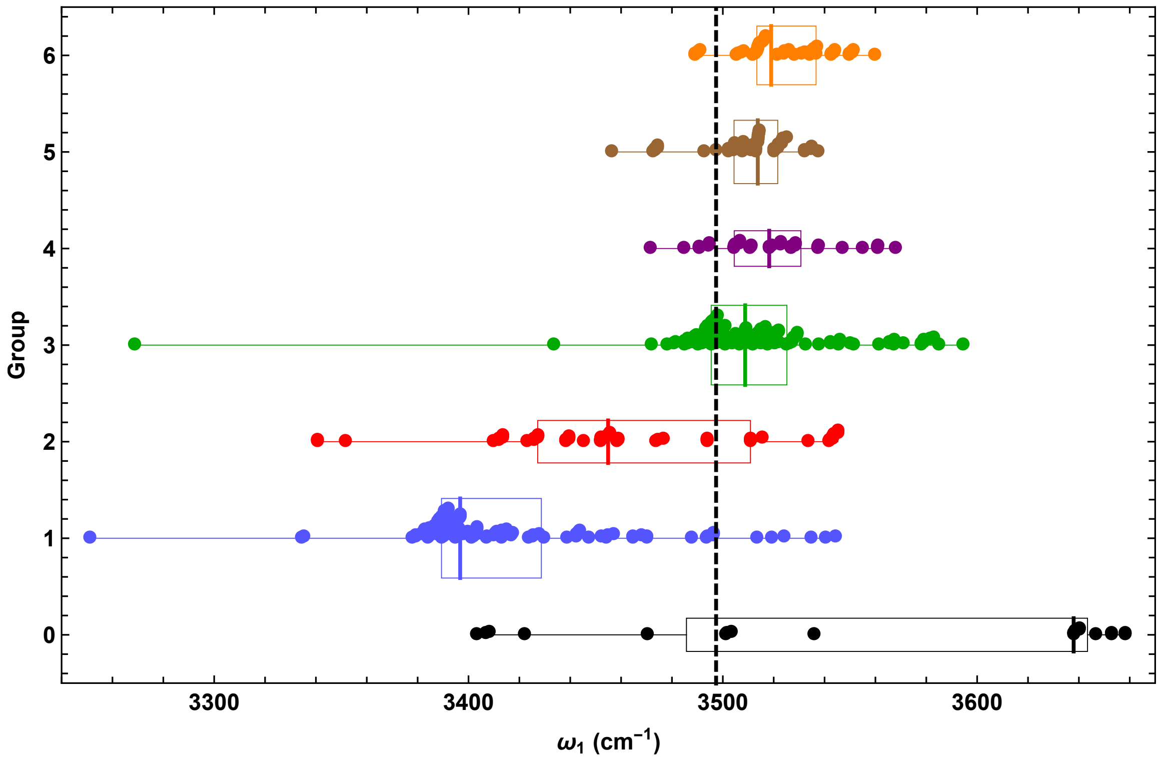

Figure S4. Scatter plot of fulminic acid ω_1 predictions for each DFA group, overlaid with box and whisker representations of the data. See the caption of Figure S2 for further explanation.

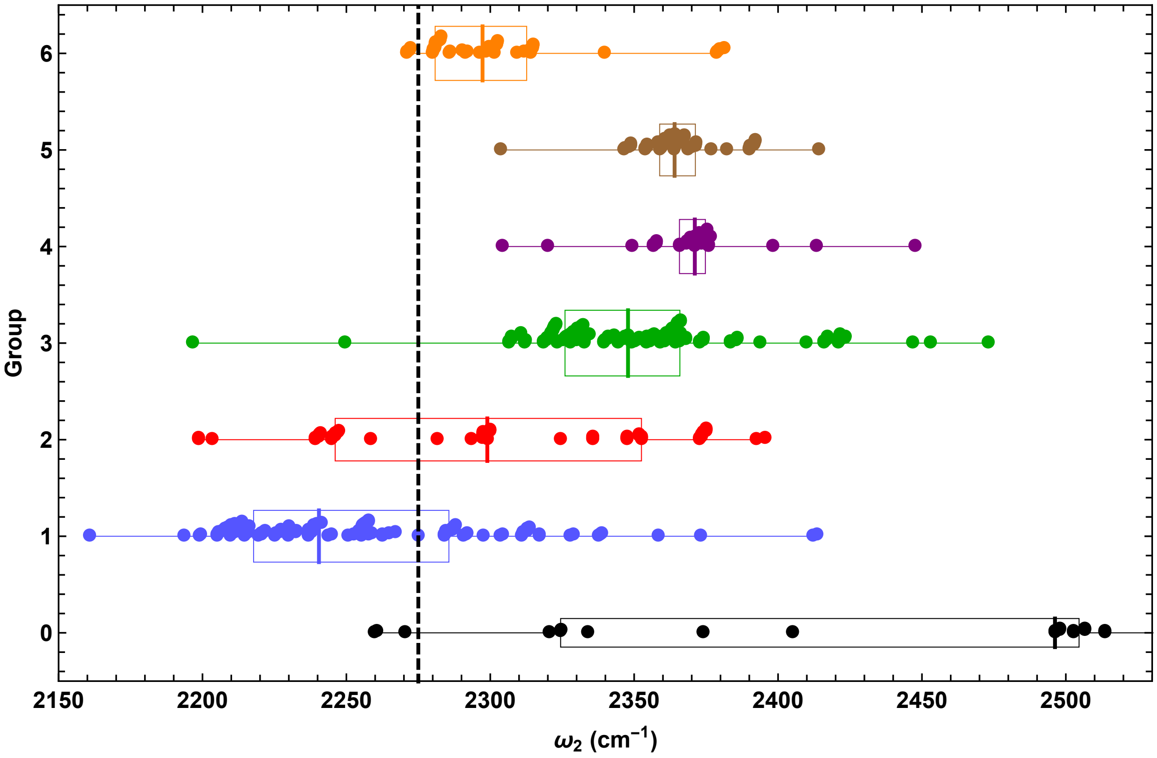

Figure S5. **Scatter plot of fulminic acid ω_2 predictions for each DFA group, overlaid with box and whisker representations of the data. See the caption of Figure S2 for further explanation.**

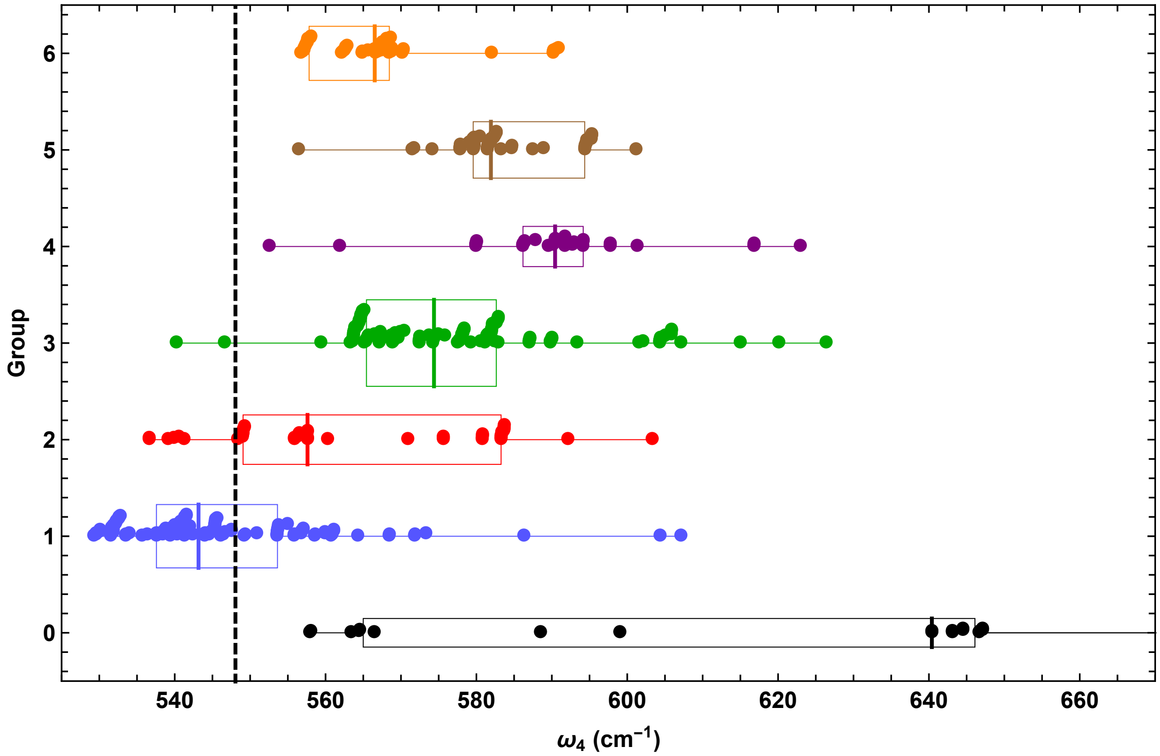

Figure S6. Scatter plot of (linear, bent) HCNO [$\omega_{4a}(\pi)$, $\omega_4(a^{'})$] predictions for each DFA group, overlaid with box and whisker representations of the data. See the caption of Figure S2 for further explanation.

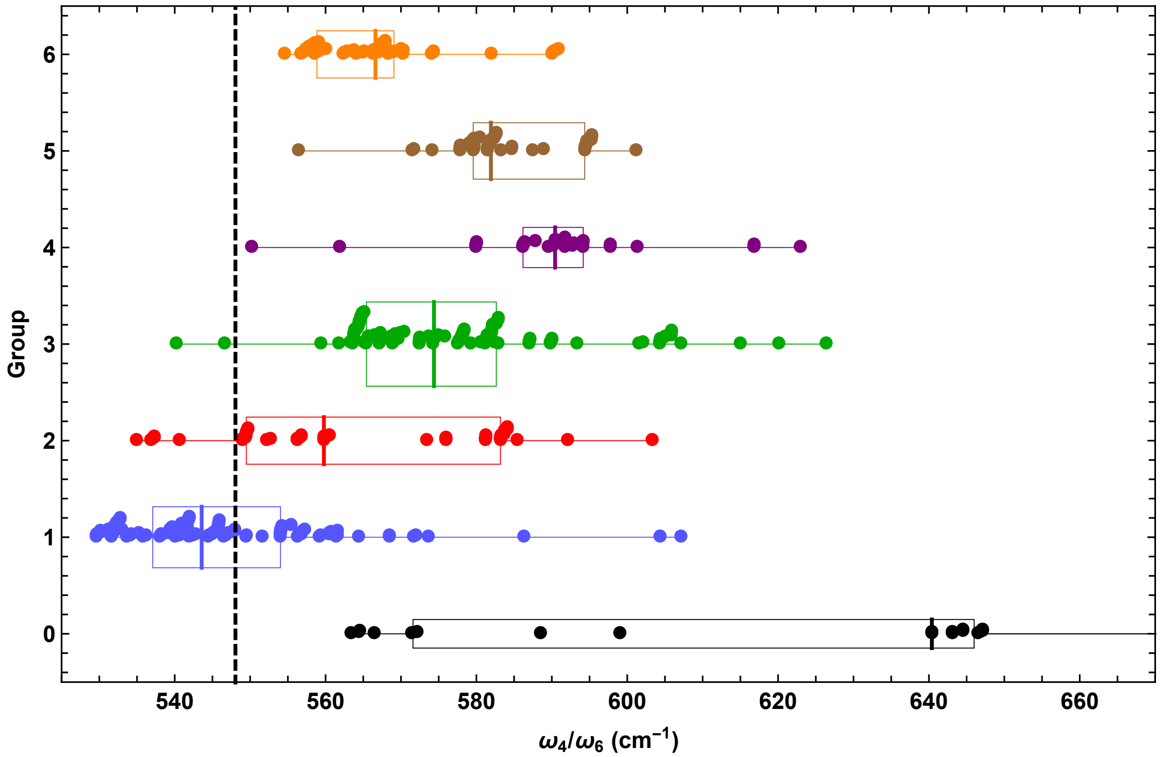

Figure S7. Scatter plot of (linear, bent) HCNO $[\omega_{4b}(\pi),\ \omega_6(a'')]$ predictions for each DFA group, overlaid with box and whisker representations of the data. See the caption of Figure S2 for further explanation.

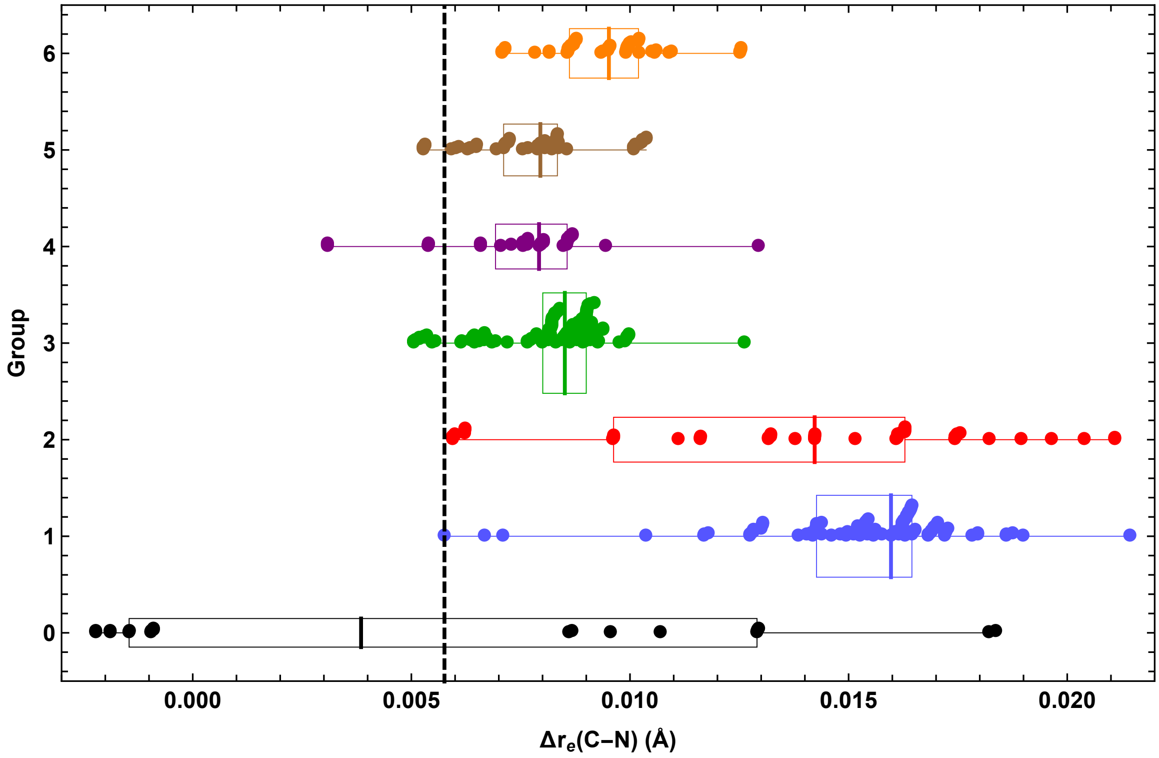

Figure S8. Scatter plot of HCN → HCNO Δr_e(C–N) predictions for each DFA group, overlaid with box and whisker representations of the data. See the caption of Figure S2 for further explanation.

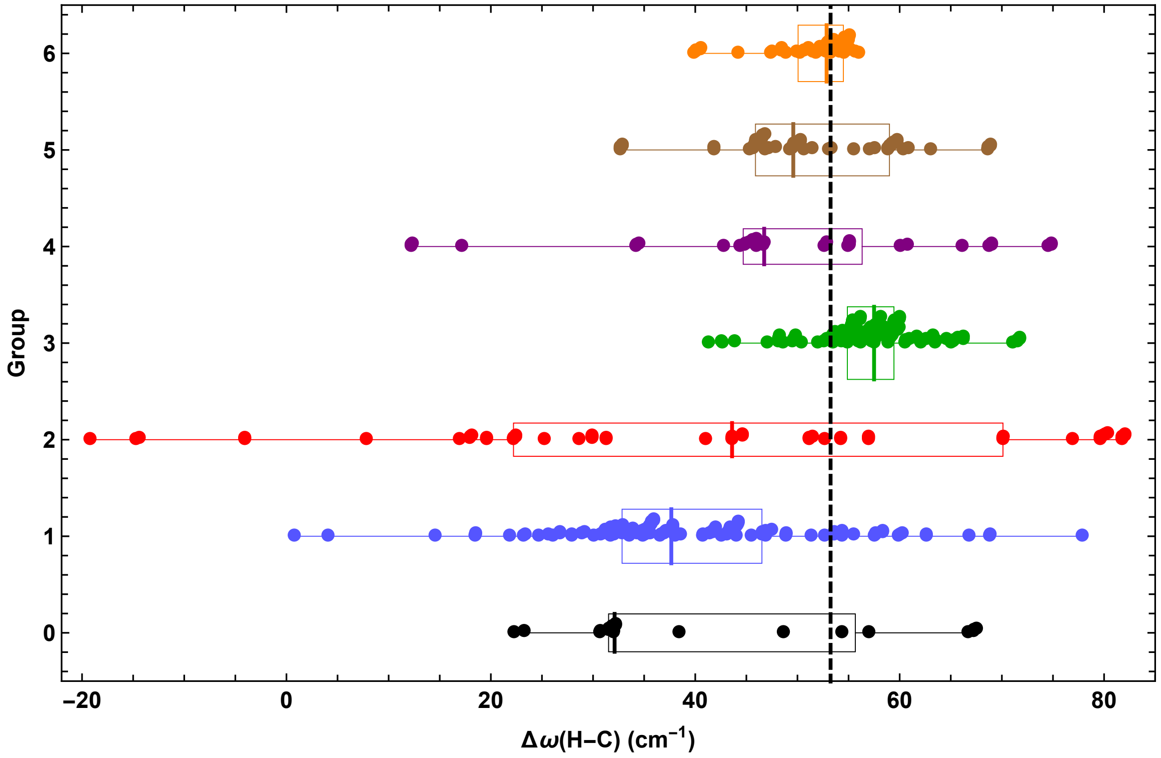

Figure S9. Scatter plot of HCN → HCNO Δω(H–C str.) predictions for each DFA group, overlaid with box and whisker representations of the data. See the caption of Figure S2 for further explanation.

Table S7. Bond distance shifts (Å) in fulminic acid from dispersion (D) corrections

Distance	−D correction	Count	Range	Mean	MAS	Std. Dev.	% minus
r_e(H–C)	−D	1	[−0.00126, −0.00126]	−0.00126	0.00126	none	100.0
r_e(H–C)	−D3	1	[−0.00045, −0.00045]	−0.00045	0.00045	none	100.0
r_e(H–C)	−D3(BJ)	5	[0.00048, 0.00127]	0.00094	0.00094	0.00031	0.0
r_e(H–C)	−D3(BJ)2B	55	[−0.00137, 0.00007]	−0.00009	0.00009	0.00019	92.7
r_e(H–C)	−D3(BJ)ATM	54	[−0.00137, 0.00000]	−0.00009	0.00009	0.00019	94.4
r_e(H–C)	−D3(0)2B	62	[−0.00108, 0.00039]	0.00011	0.00014	0.00018	4.8
r_e(H–C)	−D3(0)ATM	62	[−0.00108, 0.00039]	0.00011	0.00014	0.00018	4.8
r_e(H–C)	−D3M(BJ)2B	8	[−0.00204, −0.00006]	−0.00034	0.00034	0.00069	100.0
r_e(H–C)	−D3M(BJ)ATM	8	[−0.00204, −0.00006]	−0.00034	0.00034	0.00069	100.0
r_e(H–C)	−D3M(0)2B	8	[−0.00181, 0.00042]	−0.00032	0.00062	0.00084	37.5
r_e(H–C)	−D3M(0)ATM	8	[−0.00181, 0.00042]	−0.00032	0.00062	0.00084	37.5
r_e(C–N)	−D	1	[0.00130, 0.00130]	0.00130	0.00130	none	0.0
r_e(C–N)	−D3	1	[0.00070, 0.00070]	0.00070	0.00070	none	0.0
r_e(C–N)	−D3(BJ)	5	[−0.00143, 0.00170]	0.00034	0.00094	0.00117	40.0
r_e(C–N)	−D3(BJ)2B	55	[−0.00090, 0.00027]	−0.00003	0.00006	0.00014	60.0
r_e(C–N)	−D3(BJ)ATM	54	[−0.00090, 0.00014]	−0.00003	0.00005	0.00013	59.3
r_e(C–N)	−D3(0)2B	62	[−0.00001, 0.00030]	0.00011	0.00012	0.00008	1.6
r_e(C–N)	−D3(0)ATM	62	[−0.00001, 0.00030]	0.00011	0.00012	0.00008	3.2
r_e(C–N)	−D3M(BJ)2B	8	[−0.00164, 0.00010]	−0.00021	0.00026	0.00058	62.5
r_e(C–N)	−D3M(BJ)ATM	8	[−0.00164, 0.00010]	−0.00021	0.00026	0.00058	62.5
r_e(C–N)	−D3M(0)2B	8	[−0.00269, 0.00045]	−0.00049	0.00080	0.00114	37.5
r_e(C–N)	−D3M(0)ATM	8	[−0.00269, 0.00045]	−0.00049	0.00080	0.00114	37.5
r_e(N–O)	−D	1	[−0.00136, −0.00136]	−0.00136	0.00136	none	100.0
r_e(N–O)	−D3	1	[−0.00103, −0.00103]	−0.00103	0.00103	none	100.0
r_e(N–O)	−D3(BJ)	5	[0.00088, 0.00451]	0.00217	0.00217	0.00139	0.0
r_e(N–O)	−D3(BJ)2B	55	[−0.00132, 0.00000]	−0.00012	0.00012	0.00023	92.7
r_e(N–O)	−D3(BJ)ATM	54	[−0.00132, 0.00000]	−0.00012	0.00012	0.00023	92.6
r_e(N–O)	−D3(0)2B	62	[−0.00154, 0.00063]	0.00012	0.00017	0.00025	4.8
r_e(N–O)	−D3(0)ATM	62	[−0.00154, 0.00063]	0.00012	0.00017	0.00025	4.8
r_e(N–O)	−D3M(BJ)2B	8	[−0.00265, −0.00006]	−0.00045	0.00045	0.00089	100.0
r_e(N–O)	−D3M(BJ)ATM	8	[−0.00265, −0.00006]	−0.00045	0.00045	0.00090	100.0
r_e(N–O)	−D3M(0)2B	8	[−0.00123, 0.00051]	−0.00008	0.00047	0.00060	37.5
r_e(N–O)	−D3M(0)ATM	8	[−0.00123, 0.00051]	−0.00008	0.00047	0.00060	37.5

MAS = mean absolute shift

Table S8. Bond angle shifts (deg) in fulminic acid from dispersion (D) corrections

Angle	–D correction	Count	Range	Mean	MAS	Std. Dev.	% minus
θ_e(H–C–N)	–D3(BJ)	3	[–1.72, –1.00]	–1.47	1.47	0.41	100.0
θ_e(H–C–N)	–D3(BJ)2B	24	[–4.90, –0.01]	–0.45	0.45	0.98	100.0
θ_e(H–C–N)	–D3(BJ)ATM	23	[–0.89, –0.01]	–0.26	0.26	0.24	100.0
θ_e(H–C–N)	–D3(0)2B	24	[–3.17, 1.02]	0.16	0.42	0.76	12.5
θ_e(H–C–N)	–D3(0)ATM	24	[–3.17, 1.02]	0.15	0.42	0.76	8.3
θ_e(H–C–N)	–D3M(BJ)2B	4	[–0.65, –0.04]	–0.40	0.40	0.30	100.0
θ_e(H–C–N)	–D3M(BJ)ATM	4	[–0.65, –0.03]	–0.39	0.39	0.30	100.0
θ_e(H–C–N)	–D3M(0)2B	4	[0.14, 1.33]	0.64	0.64	0.57	0.0
θ_e(H–C–N)	–D3M(0)ATM	4	[0.14, 1.33]	0.62	0.62	0.56	0.0
θ_e(C–N–O)	–D3(BJ)	3	[–0.41, –0.23]	–0.34	0.34	0.10	100.0
θ_e(C–N–O)	–D3(BJ)2B	24	[–1.15, 0.00]	–0.10	0.10	0.23	100.0
θ_e(C–N–O)	–D3(BJ)ATM	23	[–0.20, 0.00]	–0.06	0.06	0.05	100.0
θ_e(C–N–O)	–D3(0)2B	24	[–0.68, 0.22]	0.04	0.10	0.17	12.5
θ_e(C–N–O)	–D3(0)ATM	24	[–0.68, 0.22]	0.04	0.10	0.17	8.3
θ_e(C–N–O)	–D3M(BJ)2B	4	[–0.14, 0.00]	–0.08	0.08	0.07	100.0
θ_e(C–N–O)	–D3M(BJ)ATM	4	[–0.14, 0.00]	–0.08	0.08	0.07	100.0
θ_e(C–N–O)	–D3M(0)2B	4	[0.04, 0.34]	0.16	0.16	0.14	0.0
θ_e(C–N–O)	–D3M(0)ATM	4	[0.04, 0.34]	0.15	0.15	0.14	0.0

MAS = mean absolute shift

Table S9. Stretching frequency shifts (cm^{-1}) in fulminic acid from dispersion (D) corrections

Frequency	–D correction	Count	Range	Mean	MAS	Std. Dev.	% minus
ω_1	–D	1	[−5.87, −5.87]	−5.87	5.87	none	100.00
ω_1	–D3	1	[−0.91, −0.91]	−0.91	0.91	none	100.00
ω_1	–D3(BJ)	5	[−13.09, −2.83]	−8.20	8.20	3.87	100.0
ω_1	–D3(BJ)2B	55	[−2.26, 12.61]	0.62	0.86	1.86	18.2
ω_1	–D3(BJ)ATM	54	[−1.49, 12.61]	0.67	0.74	1.77	13.0
ω_1	–D3(0)2B	62	[−10.99, 1.41]	−1.90	1.97	1.73	91.9
ω_1	–D3(0)ATM	62	[−10.99, 1.40]	−1.92	1.98	1.72	91.9
ω_1	–D3M(BJ)2B	8	[0.03, 17.98]	2.84	2.84	6.13	0.0
ω_1	–D3M(BJ)ATM	8	[0.32, 17.97]	2.90	2.90	6.10	0.0
ω_1	–D3M(0)2B	8	[−4.08, 7.87]	−0.46	2.90	3.84	75.0
ω_1	–D3M(0)ATM	8	[−4.09, 7.87]	−0.51	2.96	3.84	75.0
ω_2	–D	1	[−12.55, −12.55]	−12.55	12.55	none	100.00
ω_2	–D3	1	[−4.28, −4.28]	−4.28	4.28	none	100.00
ω_2	–D3(BJ)	5	[−11.48, 4.45]	−6.42	8.20	6.33	80.0
ω_2	–D3(BJ)2B	55	[−4.09, 8.68]	0.46	0.72	1.50	12.7
ω_2	–D3(BJ)ATM	54	[−1.30, 8.68]	0.54	0.60	1.34	11.1
ω_2	–D3(0)2B	62	[−4.70, 0.66]	−1.26	1.28	0.94	91.9
ω_2	–D3(0)ATM	62	[−4.70, 0.66]	−1.27	1.29	0.93	95.2
ω_2	–D3M(BJ)2B	8	[0.13, 15.66]	2.39	2.39	5.37	0.0
ω_2	–D3M(BJ)ATM	8	[0.25, 15.66]	2.40	2.40	5.36	0.0
ω_2	–D3M(0)2B	8	[−3.48, 7.62]	0.60	3.05	3.92	62.5
ω_2	–D3M(0)ATM	8	[−3.46, 7.62]	0.57	3.08	3.93	62.5
ω_3	–D	1	[−3.98, −3.98]	−3.98	3.98	none	100.00
ω_3	–D3	1	[−0.25, −0.25]	−0.25	0.25	none	100.00
ω_3	–D3(BJ)	5	[−15.24, −2.26]	−8.30	8.30	4.74	100.0
ω_3	–D3(BJ)2B	55	[−4.86, 7.78]	0.58	0.79	1.46	9.1
ω_3	–D3(BJ)ATM	54	[−0.04, 7.78]	0.63	0.63	1.23	5.6
ω_3	–D3(0)2B	62	[−3.81, 0.96]	−1.11	1.16	0.84	93.5
ω_3	–D3(0)ATM	62	[−3.82, 0.95]	−1.13	1.16	0.83	93.5
ω_3	–D3M(BJ)2B	8	[0.33, 13.06]	2.21	2.21	4.40	0.0
ω_3	–D3M(BJ)ATM	8	[0.33, 13.06]	2.19	2.19	4.41	0.0
ω_3	–D3M(0)2B	8	[−2.28, 2.22]	−0.70	1.56	1.60	75.0
ω_3	–D3M(0)ATM	8	[−2.25, 2.22]	−0.69	1.55	1.60	75.0

MAS = mean absolute shift

Table S10. Bending frequency shifts (cm^{-1}) in fulminic acid from dispersion (D) corrections

Frequency	−D correction	Count	Range	Mean	MAS	Std. Dev.	% minus
ω(H−C−N)	−D	1	[−56.75, −56.75]	−56.75	56.75	none	100.00
ω(H−C−N)	−D3	1	[−28.24, −28.24]	−28.24	28.24	none	100.00
ω(H−C−N)	−D3(BJ)	5	[−7.44, 61.66]	23.10	26.08	25.11	20.0
ω(H−C−N)	−D3(BJ)2B	55	[−16.22, 33.01]	1.11	3.22	6.03	60.0
ω(H−C−N)	−D3(BJ)ATM	54	[−5.66, 11.67]	0.97	2.51	3.73	53.7
ω(H−C−N)	−D3(0)2B	62	[−13.79, 25.73]	0.01	2.71	4.83	38.7
ω(H−C−N)	−D3(0)ATM	62	[−13.79, 25.73]	0.01	2.72	4.86	37.1
ω(H−C−N)	−D3M(BJ)2B	8	[−7.95, 12.74]	2.19	5.15	6.89	62.5
ω(H−C−N)	−D3M(BJ)ATM	8	[−7.95, 14.06]	2.31	5.36	7.21	62.5
ω(H−C−N)	−D3M(0)2B	8	[−15.86, 3.60]	−2.76	5.24	7.09	37.5
ω(H−C−N)	−D3M(0)ATM	8	[−15.86, 3.60]	−2.55	5.03	6.99	37.5
ω(C−N−O)	−D	1	[−5.13, −5.13]	−5.13	5.13	none	100.00
ω(C−N−O)	−D3	1	[−2.35, −2.35]	−2.35	2.35	none	100.00
ω(C−N−O)	−D3(BJ)	5	[−5.63, 2.41]	−1.84	2.80	3.16	80.0
ω(C−N−O)	−D3(BJ)2B	55	[−4.35, 2.77]	−0.12	0.42	0.87	85.5
ω(C−N−O)	−D3(BJ)ATM	54	[−2.29, 1.55]	−0.08	0.28	0.52	83.3
ω(C−N−O)	−D3(0)2B	62	[−3.29, 2.71]	0.14	0.28	0.62	14.5
ω(C−N−O)	−D3(0)ATM	62	[−3.29, 2.82]	0.16	0.28	0.63	11.3
ω(C−N−O)	−D3M(BJ)2B	8	[−3.68, 0.96]	−0.37	0.78	1.42	75.0
ω(C−N−O)	−D3M(BJ)ATM	8	[−3.68, 0.69]	−0.43	0.71	1.36	75.0
ω(C−N−O)	−D3M(0)2B	8	[−6.39, 2.07]	−0.15	1.45	2.59	12.5
ω(C−N−O)	−D3M(0)ATM	8	[−6.39, 2.07]	−0.14	1.45	2.60	12.5

MAS = mean absolute shift

Table S11. Shifts in ΔE_e[HCN + O(^3P) → HCNO] (kcal mol^{-1}) from dispersion (D) corrections

−D correction	Count	Range	Mean	MAS	Std. Dev.	% minus
−D	1	[−1.58, −1.58]	−1.58	1.58	none	100.0
−D3	1	[−0.93, −0.93]	−0.93	0.93	none	100.0
−D3(BJ)	5	[1.20, 2.61]	1.68	1.68	0.59	0.0
−D3(BJ)2B	55	[−3.92, −0.02]	−0.59	0.59	0.66	100.0
−D3(BJ)ATM	54	[−3.92, 0.00]	−0.59	0.59	0.66	100.0
−D3(0)2B	62	[−1.83, 0.00]	−0.17	0.17	0.23	100.0
−D3(0)ATM	62	[−1.83, 0.00]	−0.17	0.17	0.23	100.0
−D3M(BJ)2B	8	[−6.26, −0.48]	−1.39	1.39	1.98	100.0
−D3M(BJ)ATM	8	[−6.26, −0.48]	−1.39	1.39	1.98	100.0
−D3M(0)2B	8	[−4.35, −0.17]	−0.90	0.90	1.41	100.0
−D3M(0)ATM	8	[−4.35, −0.17]	−0.90	0.90	1.41	100.0

MAS = mean absolute shift

Table S12. Overall rankings (*n*) of DFAs applied to HCNO

n	Functional	Group	Mean rank	*n*	Functional	Group	Mean rank
1	B2GP−PLYP−NL	6	111.00	41	X3LYP−D3(BJ)ATM	3	124.60
2	B2GP−PLYP−D3(0)ATM	6	111.53	42	MPW3LYP	3	124.80
3	B2GP−PLYP	6	112.00	43	DSD−PBEB95−NL	6	125.20
4	B2GP−PLYP−D3(BJ)ATM	6	112.13	44	B3LYP−NL	3	125.27
5	B3LYP5	3	112.40	45	B97−1	3	126.73
6	PWPB95	6	113.47	46	B97−1−D3(BJ)2B	3	127.53
7	PWPB95−NL	6	114.00	46	B97−1−D3(BJ)ATM	3	127.53
8	B3LYP	3	114.47	48	DSD−PBEB95−D3(BJ)	6	127.87
9	PWPB95−D3(BJ)2B	6	114.53	49	SB98−2a	3	129.13
10	PWPB95−D3(0)ATM	6	115.27	50	B97−0	3	131.47
11	B1LYP−D3(0)2B	3	115.33	51	B97−1−D3(0)2B	3	131.53
11	B1LYP−D3(0)ATM	3	115.33	52	B97−1−D3(0)ATM	3	131.67
13	B2GP−PLYP−D3(0)2B	6	115.47	53	SB98−1c	3	132.60
14	PWPB95−D3(0)2B	6	115.60	54	ωB97M−D3(BJ)	5	133.13
15	B2GP−PLYP−D3(BJ)2B	6	115.87	55	ωB97M−V	5	134.20
16	PWPB95−D3(BJ)ATM	6	116.13	56	B3LYPS	3	134.33
17	B1LYP	3	118.00	57	revB3LYP	3	136.80
18	B3LYP−D3(BJ)2B	3	118.13	58	B97−K	3	139.33
19	B3LYP−D3(BJ)ATM	3	118.27	59	τ−HCTHh	4	142.40
20	B1LYP−D3(BJ)ATM	3	118.47	60	B97−1P	3	143.67
21	B1LYP−D3(BJ)2B	3	118.73	61	SB98−2b	3	144.73
22	X3LYP	3	119.73	62	B2PLYP	6	145.00
23	mPW1LYP−D3(0)2B	3	120.20	63	B2PLYP−D3(0)2B	6	146.80
24	B3LYP−D3(0)2B	3	120.27	64	B2PLYP−D3(0)ATM	6	147.47
25	B3LYP−D3M(BJ)2B	3	120.33	65	B2PLYP−D3M(0)ATM	6	148.07
26	B3LYP−D3M(BJ)ATM	3	120.40	66	ωB97X−V	5	148.60
27	mPW1LYP−D3(0)ATM	3	120.47	67	B2PLYP−NL	6	148.93
28	B3LYP−D3(0)ATM	3	120.53	68	B2PLYP−D3(BJ)ATM	6	149.27
29	B3LYP−D3M(0)2B	3	122.27	69	B2PLYP−D3M(0)2B	6	149.47
30	B3LYP−D3M(0)ATM	3	122.53	70	ωB97X−D3(BJ)	5	149.73
30	TPSSh	3	122.53	71	tuned−CAM−B3LYP	5	151.27
32	mPW1LYP	3	122.67	72	B2PLYP−D3M(BJ)ATM	6	152.00
33	TPSSh−D3(BJ)2B	3	123.00	73	B2PLYP−D3M(BJ)2B	6	153.47
34	TPSSh−D3(BJ)ATM	3	123.53	74	DSD−PBEB95	6	154.60
35	TPSSh−D3(0)2B	3	124.13	75	B2PLYP−D3(BJ)2B	6	155.00
36	X3LYP−D3(0)2B	3	124.27	76	B97−3	3	157.87
37	TPSSh−D3(0)ATM	3	124.33	77	ωB97X−D	5	159.13
38	SB98−2c	3	124.40	78	CAM−B3LYP−D3(0)2B	5	159.33
39	X3LYP−D3(BJ)2B	3	124.53	79	CAM−B3LYP−D3(0)ATM	5	159.47
39	X3LYP−D3(0)ATM	3	124.53	80	CAM−B3LYP−D3(BJ)2B	5	161.93

Table S12 (continued)

n	Functional	Group	Mean rank	*n*	Functional	Group	Mean rank
81	CAM−B3LYP−D3(BJ)ATM	5	162.20	121	revTPSSh	3	184.73
82	CAM−B3LYP	5	163.20	122	M06−2X	4	185.00
83	EDF2	3	166.33	123	core−DSD−BLYP	6	185.20
84	revPBE0	3	167.60	123	M06−2X−D3(0)2B	4	185.20
85	revPBE0−D3(0)2B	3	168.87	125	M06−2X−D3(0)ATM	4	185.40
86	revPBE0−D3(0)ATM	3	169.00	126	B86B95	3	185.53
87	ωB97X−D3	5	170.13	127	core−DSD−BLYP−D3(BJ)ATM	6	185.87
88	B3PW91	3	170.27	128	mPW1PW−D3(BJ)2B	3	186.40
89	LRC−ωPBE	5	171.20	129	revTPSSh−D3(BJ)ATM	3	186.47
90	B3P86	3	171.27	130	revTPSSh−D3(BJ)2B	3	186.67
91	O3LYP	3	171.47	131	mPW1PW−D3(BJ)ATM	3	186.87
92	revPBE0−D3(BJ)ATM	3	171.87	132	B97−2−D3(0)2B	3	187.00
93	revPBE0−D3(BJ)2B	3	171.93	132	mPW1PW−D3(0)2B	3	187.00
94	B3P86−D3(BJ)2B	3	172.53	134	mPW1PW−D3(0)ATM	3	187.07
95	B3P86−D3(BJ)ATM	3	172.60	135	B97−2−D3(0)ATM	3	187.13
96	O3LYP−D3(BJ)2B	3	172.67	136	BMK−D3(BJ)2B	4	187.27
97	O3LYP−D3(BJ)ATM	3	172.80	136	BMK−D3(0)2B	4	187.27
98	O3LYP−D3(0)2B	3	173.47	138	BMK−D3(0)ATM	4	187.40
98	O3LYP−D3(0)ATM	3	173.47	139	BMK−D3(BJ)ATM	4	187.67
100	revPBE0−NL	3	173.80	140	DSD−BLYP−NL	6	188.20
101	ωB97	1	173.87	141	BMK	4	188.33
102	B3PW91−D3(BJ)2B	3	174.73	142	HSE06	5	189.60
103	B3PW91−D3(BJ)ATM	3	175.00	143	mPW1PBE	3	190.73
104	B1PW91	3	175.67	144	CAP0	3	190.80
105	B3PW91−NL	3	175.73	145	revTPSSh−D3(0)ATM	3	190.87
106	B3PW91−D3(0)2B	3	176.13	146	revTPSSh−D3(0)2B	3	190.93
107	B3PW91−D3(0)ATM	3	176.33	147	PTPSS−D3(0)ATM	6	191.27
107	mPW3PW	3	176.33	148	DSD−BLYP−D3(BJ)	6	191.40
109	SB98−1b	3	177.60	149	B97−2	3	191.73
110	B3P86−D3(0)2B	3	177.93	150	SOGGA11−X	3	191.80
111	B3P86−D3(0)ATM	3	178.13	151	PW6B95	4	191.87
112	mB3LYP−RC04	3	178.87	152	DSD−PBEP86−D3(BJ)	6	192.40
113	core−DSD−BLYP−D3(BJ)2B	6	180.87	153	MN12−SX	5	192.47
114	ωB97X−D3(0)2B	5	181.33	154	PTPSS−D3(BJ)2B	6	192.80
115	ωB97X	5	181.40	154	MN12−SX−D3(BJ)2B	5	192.80
116	ωB97X−D3(0)ATM	5	181.47	156	MN12−SX−D3(0)2B	5	192.87
117	MN15	5	182.00	157	MN12−SX−D3(BJ)ATM	5	192.93
117	MN15−D3(BJ)2B	5	182.00	157	MN12−SX−D3(0)ATM	5	192.93
119	MN15−D3(BJ)ATM	5	182.07	159	LRC−ωPBEh	5	193.40
120	mPW1PW	3	183.13	160	PTPSS	6	193.60

Table S12 (continued)

n	Functional	Group	Mean rank	*n*	Functional	Group	Mean rank
161	PW6B95−D3(0)ATM	4	193.67	201	HSE03−D3(BJ)2B	5	201.33
162	PTPSS−D3(0)2B	6	193.80	202	HSE03−D3(BJ)ATM	5	201.47
162	PW6B95−D3(BJ)2B	4	193.80	203	PBE0−D3(0)2B	3	201.60
162	PW6B95−D3(0)2B	4	193.80	204	PBE0−D3(0)ATM	3	201.67
165	PW6B95−D3(BJ)ATM	4	193.93	205	PBE0−1/3	3	202.07
166	PBE0−2	6	194.00	206	PBE0−DH−D3(0)2B	6	202.60
167	PTPSS−D3(BJ)ATM	6	194.27	207	HSE03	5	203.33
168	SOGGA11−X−D3(BJ)2B	3	194.47	208	SCAN0	4	205.13
169	SOGGA11−X−D3(BJ)ATM	3	194.67	209	PBE0−D3M(0)2B	3	205.47
170	SOGGA11−X−D3(0)2B	3	194.87	210	PBE0−D3M(0)ATM	3	205.60
171	PW86B95	3	195.00	211	DSD−PBEPBE−NL	6	207.07
172	SOGGA11−X−D3(0)ATM	3	195.13	212	M08−HX	4	213.13
173	HJS−PBE	5	195.67	213	M08−HX−D3(0)2B	4	213.27
174	DSD−PBEP86−NL	6	196.07	214	M08−HX−D3(0)ATM	4	213.53
175	DSD−PBEP86	6	196.87	215	SB98−1a	3	214.53
176	B88B95	3	197.20	216	ωPBE0	5	215.00
177	PBE0	3	198.07	217	MPW1B95	3	215.07
178	B88B95−D3(0)2B	3	198.27	218	MPW1B95−D3(0)2B	3	216.33
178	PBE0−DH	6	198.27	219	MPW1B95−D3(0)ATM	3	216.60
180	DSD−PBEPBE−D3(BJ)	6	198.53	220	MPW1B95−D3(BJ)2B	3	216.67
180	B88B95−D3(0)ATM	3	198.53	220	MPW1B95−D3(BJ)ATM	3	216.67
180	HSE06−D3(BJ)ATM	5	198.53	222	PBEh−3c	3	217.47
183	HSE06−D3(BJ)2B	5	198.60	223	DLDF+D09	4	219.33
184	B97−2−D3(BJ)2B	3	199.13	223	DLDF+D10	4	219.33
184	B97−2−D3(BJ)ATM	3	199.13	225	DLDF	4	219.80
186	PBE0−NL	3	199.20	226	DSD−BLYP−D3(0)ATM	6	220.07
187	HSE06−D3(0)ATM	5	199.27	227	DSD−BLYP	6	220.33
188	HSE06−D3(0)2B	5	199.40	228	B1WC	3	221.40
189	PBE0−DH−D3(0)ATM	6	199.73	228	ωPBE−D3M(0)2B	5	221.40
190	PBE0−D3(BJ)2B	3	200.00	230	ωPBE−D3M(0)ATM	5	221.60
191	PBE0−D3(BJ)ATM	3	200.33	231	ωPBE−D3(0)2B	5	221.67
192	PBE0−D3M(BJ)2B	3	200.40	232	ωPBE−D3(0)ATM	5	222.00
193	PBE0−D3M(BJ)ATM	3	200.60	233	DSD−BLYP−D3(BJ)ATM	6	222.07
193	PBE0−DH−D3(BJ)2B	6	200.60	234	N12−SX−D3(0)2B	5	222.67
195	M08−SO	4	200.87	235	N12−SX−D3(0)ATM	5	222.93
196	HSE03−D3(0)2B	5	201.07	236	DSD−PBEPBE	6	223.47
196	PBE0−DH−D3(BJ)ATM	6	201.07	237	DSD−BLYP−D3(0)2B	6	223.80
198	B88B95−D3(BJ)2B	3	201.13	238	ωPBE−D3M(BJ)2B	5	224.87
198	B88B95−D3(BJ)ATM	3	201.13	239	ωPBE	5	224.93
200	HSE03−D3(0)ATM	5	201.20	240	ωPBE−D3M(BJ)ATM	5	225.00

Table S12 (continued)

n	Functional	Group	Mean rank	*n*	Functional	Group	Mean rank
241	ωPBE−D3(BJ)2B	5	225.13	281	τ−HCTH−D3(0)ATM	2	242.00
242	ωPBE−D3(BJ)ATM	5	225.40	282	τ−HCTH−D3(0)2B	2	242.47
243	N12	1	226.20	283	PW91	1	243.33
244	N12−SX	5	226.27	284	M06−HF−D3(0)2B	4	243.53
245	N12−SX−D3(BJ)2B	5	226.33	285	M06−HF−D3(0)ATM	4	243.60
246	N12−SX−D3(BJ)ATM	5	226.40	286	CAM−LDA0	0	243.67
247	hPBEint	3	227.53	287	M06−HF	4	243.80
248	DSD−BLYP−D3(BJ)2B	6	227.87	288	PW91−D3(BJ)ATM	1	244.27
249	BB1K	3	228.27	289	PW91−D3(BJ)2B	1	244.33
250	N12−D3(BJ)ATM	1	228.87	290	LDA0	0	244.80
251	N12−D3(BJ)2B	1	229.00	291	HCTH/120	1	245.20
252	TH4	1	230.40	292	B97−D	1	245.80
253	MPWLYP1M	3	230.87	293	HCTH/120−D3(0)ATM	1	245.87
253	VSXC	2	230.87	294	HCTH/120−D3(0)2B	1	245.93
255	M06	4	231.47	294	PBE50	3	245.93
256	M06−D3(0)2B	4	232.33	296	HJS−B97x	5	247.07
257	MN12−L−D3(0)2B	2	232.80	297	HJS−PBEsol	5	247.13
258	MGGA_MS2h	4	232.87	298	HCTH/147	1	248.20
258	M06−D3(0)ATM	4	232.87	299	HCTH/120−D3(BJ)2B	1	248.40
260	MPWB1K	3	232.93	300	HCTH/120−D3(BJ)ATM	1	248.60
260	MPWB1K−D3(0)2B	3	232.93	301	PWB6K−D3(0)2B	3	249.87
262	MPWB1K−D3(0)ATM	3	233.20	301	PWB6K−D3(0)ATM	3	249.87
263	MPWB1K−D3(BJ)2B	3	233.27	303	LC−VV10	1	250.73
264	MPWB1K−D3(BJ)ATM	3	233.40	304	PWB6K−D3(BJ)2B	3	250.93
265	MPW1K	3	233.67	305	PWB6K−D3(BJ)ATM	3	251.00
266	M05−2X−D3(0)2B	4	233.73	306	PBE	1	251.47
267	MN12−L−D3(0)ATM	2	233.93	307	PWB6K	3	251.60
268	M05−2X	4	234.00	308	BHandH	3	252.07
269	M05−2X−D3(0)ATM	4	234.60	309	PBE−D3M(BJ)ATM	1	252.20
269	MN12−L	2	234.60	310	XB1K	3	252.33
271	MN12−L−D3(BJ)2B	2	234.80	311	PBE−D3(BJ)2B	1	252.47
272	MN12−L−D3(BJ)ATM	2	234.87	312	PBE−D3(BJ)ATM	1	252.60
273	N12−D3(0)ATM	1	235.47	312	PBE−D3M(BJ)2B	1	252.60
274	N12−D3(0)2B	1	235.93	314	B97−D3(BJ)	1	253.00
275	τ−HCTH	2	238.27	315	PBE−D3(0)2B	1	253.47
276	PBE−D3M(0)ATM	1	240.53	316	PBE−D3(0)ATM	1	253.60
277	PBE−D3M(0)2B	1	240.60	317	M11−D3(0)2B	5	254.87
278	EDF1	1	240.67	318	M11−D3(BJ)2B	5	254.93
279	τ−HCTH−D3(BJ)ATM	2	241.53	319	M11−D3(0)ATM	5	255.20
280	τ−HCTH−D3(BJ)2B	2	241.60	320	B86bPBE	1	255.27

Table S12 (continued)

n	Functional	Group	Mean rank	*n*	Functional	Group	Mean rank
320	M11	5	255.27	361	MGGA–MVSh	4	281.00
322	M11–D3(BJ)ATM	5	255.33	362	BP86–D3(0)2B	1	281.60
323	KSDT	0	255.53	363	BP86–D3(0)ATM	1	281.87
324	PW86PBE	1	256.13	364	BP86–D3M(0)2B	1	282.13
325	revSCAN	2	256.80	365	BP86–D3M(0)ATM	1	282.27
326	MN15–L	2	257.40	366	FT97	1	282.33
326	MN15–L–D3(0)ATM	2	257.40	367	HCTH/407+	1	282.53
328	MN15–L–D3(0)2B	2	257.73	368	MGGA_MS1	2	283.40
329	mPWPW	1	257.80	369	TPSS–NL	2	284.07
330	PBE–NL	1	257.87	370	HCTH/407–D3(BJ)ATM	1	286.13
331	B97–D3M(BJ)	1	258.80	371	HCTH/407–D3(BJ)2B	1	286.20
332	SPW92	0	259.20	372	HCTH–p(1/4)	1	286.40
333	HCTH/93	1	260.27	373	BP86–D3(BJ)2B	1	287.47
334	Teter93	0	260.33	374	BP86–D3(BJ)ATM	1	287.67
335	revSCAN0	4	260.53	375	BP86–D3M(BJ)2B	1	288.13
336	M05	4	261.60	376	BHandHLYP	3	288.20
337	MGGA_MS0	2	262.60	377	SOGGA	1	288.47
338	M05–D3(0)2B	4	262.67	378	BP86–D3M(BJ)ATM	1	288.53
338	TH–FL	1	262.67	379	HCTH/407	1	288.67
340	M05–D3(0)ATM	4	262.80	380	SCAN–D3(BJ)2B	2	289.60
341	SOGGA11	1	263.07	381	BP86	1	290.40
342	PBE1W	1	263.67	382	HCTH/407–D3(0)2B	1	291.20
343	PBEsol	1	264.20	382	HCTH/407–D3(0)ATM	1	291.20
344	SVWN	0	264.80	384	B5050LYP	3	292.33
345	PBEsol–D3(BJ)2B	1	265.20	385	SCAN	2	293.27
345	PBEsol–D3(BJ)ATM	1	265.20	386	SCAN–D3(0)2B	2	293.53
347	LC–BOP	1	266.80	386	SCAN–D3(0)ATM	2	293.53
348	PBEsol–D3(0)2B	1	267.60	388	HCTH–p(7/6)	1	293.67
349	PBEsol–D3(0)ATM	1	267.67	389	SCAN–D3(BJ)ATM	2	294.33
350	VV10	1	269.47	390	M06–L–D3(0)ATM	2	294.40
351	$B97^{GGA}$–1	1	269.80	390	HF–3c	0	294.40
352	TPSS–D3(0)2B	2	273.33	392	M06–L–D3(0)2B	2	294.87
353	TPSS–D3(0)ATM	2	273.40	393	M06–L	2	295.33
354	B97M–D3(BJ)	1	273.53	394	MGGA_MS2	2	296.60
355	KMLYP	3	273.60	395	MGGA–MVS	2	296.87
356	B97M–V	1	275.13	396	revPBE–D3(0)2B	1	297.60
357	BP86–VWN	1	275.67	397	revPBE–D3(0)ATM	1	298.47
358	TPSS	2	275.87	398	revTPSS–D3(BJ)2B	2	300.67
359	TPSS–D3(BJ)2B	2	276.73	399	revTPSS	2	300.80
360	TPSS–D3(BJ)ATM	2	276.93	400	revTPSS–D3(BJ)ATM	2	301.13

Table S12 (continued)

n	Functional	Group	Mean rank	*n*	Functional	Group	Mean rank
401	revTPSS–D3(0)2B	2	301.40	441	BLYP–D3M(BJ)ATM	1	327.53
402	revTPSS–D3(0)ATM	2	301.47	442	revM06–L	2	329.80
403	oBLYP–D	1	301.73	443	PBEOP	1	331.47
404	TH–FCO	1	302.67	444	BLYP–NL	1	333.33
405	oPBE–D	1	303.13	445	TH–FC	1	333.67
406	TH–FC+FO	1	304.60	446	GAM	1	334.00
407	revPBE	1	306.13	447	BOP–D3(0)ATM	1	335.53
408	revTPSS–NL	2	306.80	448	BOP–D3(0)2B	1	335.73
409	oPWLYP–D	1	306.87	448	XLYP–D3(0)2B	1	335.73
410	revPBE–D3(BJ)ATM	1	307.67	450	XLYP–D3(0)ATM	1	336.07
411	TH3	1	307.87	451	TH2	1	338.27
412	revPBE–D3(BJ)2B	1	308.13	452	HF–D3(0)ATM	0	339.00
413	KT2	1	309.13	453	HF–D3(0)2B	0	339.27
414	BP86–NL	1	309.33	454	BOP–D3(BJ)2B	1	339.87
415	oTPSS–D	1	310.73	455	BOP	1	339.93
416	TPSSLYP1W	1	311.93	456	BOP–D3(BJ)ATM	1	340.00
417	revPBE–NL	1	312.40	457	XLYP–D3(BJ)ATM	1	342.00
418	PBELYP1W	1	313.93	458	XLYP–D3(BJ)2B	1	342.40
419	MPWLYP1W	1	315.87	459	XLYP	1	342.47
420	RPBE–D3(0)2B	1	317.13	460	HF+D	0	342.93
421	RPBE–D3(0)ATM	1	317.20	461	PKZB	2	343.20
422	TH1	1	318.13	462	HF	0	343.40
423	BLYP–D3(0)ATM	1	321.93	463	HF–D3M(0)2B	0	344.40
424	BLYP–D3(0)2B	1	322.07	463	HF–D3M(0)ATM	0	344.40
425	M11–L	2	322.60	465	PKZB–D3(0)2B	2	348.07
426	RPBE	1	322.73	465	PKZB–D3(0)ATM	2	348.07
427	M11–L–D3(0)2B	2	322.80	467	MOHLYP	1	349.47
428	M11–L–D3(0)ATM	2	322.87	468	HF–D3(BJ)ATM	0	350.73
429	BLYP	1	323.80	469	HF–D3(BJ)2B	0	351.07
430	M11–L–D3(BJ)ATM	2	324.00	470	HF–D3M(BJ)2B	0	355.67
431	M11–L–D3(BJ)2B	2	324.27	470	HF–D3M(BJ)ATM	0	355.67
432	MP2	0	325.07	472	MOHLYP2	1	387.67
432	RPBE–D3(BJ)ATM	1	325.07				
434	MP2D	0	325.40				
434	RPBE–D3(BJ)2B	1	325.40				
436	BLYP–D3M(0)2B	1	326.60				
437	BLYP–D3(BJ)ATM	1	326.67				
437	BLYP–D3M(0)ATM	1	326.67				
439	BLYP–D3(BJ)2B	1	327.13				
440	BLYP–D3M(BJ)2B	1	327.40				

Property Comparison: Q-Chem vs. Psi4

To test the validity of the Psi4 results, all properties in Tables 1 and 2 of the main text were re-computed with Q-Chem for those DFAs in the pruned list that are directly supported by Q-Chem. The functionals DSD-BLYP-D3(BJ), HSE06, LDA0, and ωB97X-D3 are supported by Psi4 but not Q-Chem. Statistics on the Q-Chem – Psi4 differences are given in Table S13 for the following properties: HCNO and HCN + O(^3P) total energies, the HCNO → HCN + O(^3P) dissociation energy [D_e(HCN–O)], r_e(H–C), r_e(C–N), r_e(N–O), θ_e(H–C–N), θ_e(C–N–O), and ω_1-ω_6. Two DFAs [B97-D3(BJ) and MN12-L] were excluded as outliers from the statistical analysis and will be further discussed below, giving a sample size of 29 for Table S13.

For the HCNO total energy, all differences are substantially smaller in magnitude than 1 mE_h with (median, IQR) = (0.038, 0.048) mE_h. The HCN + O(^3P) total energies have comparable agreement with (median, IQR) = (0.029, 0.031) mE_h. The largest total energy discrepancy observed for either HCNO or HCN + O(^3P) is 2.6 mE_h for B3LYP-D3(BJ)2B. The median D_e(HCN–O) difference is essentially zero with an interquartile range (IQR) of only 0.006 kcal mol^{-1}. However, the largest D_e(HCN–O) difference is –1.62 kcal mol^{-1} for B3LYP-D3(BJ)2B, consistent with the deviations in total energies. All other dissociation energy absolute differences are well below 1 kcal mol^{-1}.

By default in DFT computations, Psi4 employs a density fitting (DF) algorithm to approximate electron repulsion integrals (ERIs). To ensure that this choice does not influence the total energy results, we also report difference statistics for HCNO energies between the DF algorithm and exact treatment of ERIs, denoted as PK (Table S13). For this metric, the median energy difference is only –0.029 mE_h with an IQR of 0.036 mE_h. No absolute differences exceed 0.3 mE_h, as (min, max) = (–0.28, 0.10) mE_h for [MN15-L, DSD-PBEP86-D3(BJ)].

With the confirmation that no alarming energy differences exist in the pruned dataset of Table S13, we expect the geometric parameters and harmonic vibrational frequencies to also exhibit good agreement between Q-Chem and Psi4. The medians for differences in [r_e(H–C), r_e(C–N), r_e(N–O)] are all on the order of 10^{-5} Å. For r_e(H–C) and r_e(C–N), the (min, max) pairs for the differences are both on the order of 10^{-4} Å; while the max for r_e(N–O) also meets this threshold, the min is –0.0019 Å for SCAN-D3(BJ)2B. The bond angles for the bent minima also exhibit only minor discrepancies, as the (median, IQR) for θ_e(H–C–N) and θ_e(C–N–O) are (0.06, 0.20)° and (0.02, 0.05)°, respectively. A min θ_e(H–C–N) discrepancy of –1.97° is obtained for SOGGA11-X-D3(BJ)2B, whereas M11 produces the max deviation of 1.37°. No θ_e(C–N–O) absolute difference exceeds 0.33°. Thus, the geometric parameters exhibit only minor discrepancies between Psi4 and Q-Chem.

As a final metric, the differences in the harmonic vibrational frequencies between Psi4 and Q-Chem are generally minimal. The (ω_1, ω_2, ω_3) median differences are (–0.12, –0.02, –0.19) cm^{-1} with IQRs of (0.54, 0.20, 0.40) cm^{-1}. Of these stretching frequencies, SCAN-D3(BJ)2B gives the largest discrepancies with the differences in (ω_1, ω_2, ω_3) being (7.37, 4.16, 7.30) cm^{-1}. Differences between the programs for the (linear, bent) ω_4(C–N–O) bending frequencies are even smaller than the stretching frequencies, as no absolute value exceeds 1.6 cm^{-1} with the (median, IQR) = [(0.08, 0.16), (0.19, 0.16)] cm^{-1}. For the bent HCNO ω_6(C–N–O) frequency, the (median, IQR) results of (0.20, 0.38) cm^{-1} are comparable to those of the ω_4 frequency, albeit with a 10.47 cm^{-1} maximum difference for SOGGA11-X-D3(BJ)2B. Statistics for the linear HCNO ω_5(H–C–N) bending frequency are on par with the other bending frequencies, with no differences larger than 5 cm^{-1} and a (median, IQR) = (–0.07, 1.20) cm^{-1}. However, the bent HCNO ω_5 bending frequencies show the largest discrepancies with (Q1, median, Q3) = (–8.44, –2.13, –0.19) cm^{-1}. The (min, max) pair

for this property is (–37.62, 35.56) cm^{-1} produced by [B2GP-PLYP-D3(BJ)2B, SOGGA11-X-D3(BJ)2B]. Such differences are not unreasonable given the vast span of values for the flat ω_5 mode.

Now we discuss the two special cases cited above. In Psi4, B97-D3(BJ) is a GGA functional of Group 1, whereas the corresponding options of this class in Q-Chem are B97-D3(0) and B97-D. Adding D3(BJ) dispersion manually to the B97 keyword in Q-Chem calls up a Group 3 global hybrid functional that does not correspond to the Psi4 B97-D3(BJ) functional, as shown by energy differences larger than 40 mE_h for HCNO. We thus abandoned attempts to confirm the Psi4 results with Q-Chem in this case.

For MN12-L, optimizations of the linear structure produced very similar results between Q-Chem and Psi4 with a total energy difference of merely 0.13 mE_h, bond distance agreement to 10^{-4} Å, and ω_1-ω_4 frequency differences less than 0.5 cm^{-1}. Psi4 yields ω_5 = 33 cm^{-1} whereas Q-Chem predicts the linear structure to be a transition state with ω_5 = 19i cm^{-1}. In the case of Psi4, there is a second minimum within 0.0005 mE_h with [θ_e(H–C–N), θ_e(C–N–O)] = (175.3, 178.8)°. Optimization of a bent structure in Q-Chem yields a result with [θ_e(H–C–N), θ_e(C–N–O)] = (178.9, 179.8)° within 10^{-7} E_h of its truly linear analog that differs from the Psi4 bent minimum by only 0.13 mE_h. However, Q-Chem has the anomalous prediction of an imaginary frequency for the mode breaking the plane of symmetry. We do not deem these disparities to be of significance given the tiny energy differences.

Table S13. Statistics for Q-Chem – Psi4 differences for properties reported in Tables 1 and 2 within a pruned subset of DFAs (N = 29).[a]

	Min	Q1	Median	Q3	Max
HCNO total energies	–0.201	0.013	0.038	0.061	2.612
HCN + O(^3P) total energies	–0.164	0.014	0.029	0.045	0.278
D_e (HCN–O)	–1.623	–0.002	–0.00008	0.004	0.024
r_e(C–H)	–0.0009	–0.000003	0.00002	0.00003	0.0003
r_e(C–N)	–0.0007	–0.00004	–0.00002	–0.000003	0.0005
r_e(N–O)	–0.0019	–0.00002	0.00004	0.0001	0.0003
bent θ_e(H–C–N)	–1.97	–0.08	0.06	0.12	1.37
bent θ_e(C–N–O)	–0.25	–0.006	0.02	0.04	0.33
ω_1	–3.52	–0.37	–0.12	0.17	7.37
ω_2	–2.38	–0.14	–0.02	0.06	4.16
ω_3	–1.06	–0.40	–0.19	0.001	7.30
linear ω_4	–0.37	0.005	0.08	0.17	0.37
bent ω_4	–1.23	0.05	0.19	0.21	1.56
bent ω_6	–0.36	0.05	0.20	0.43	10.47
linear ω_5	–3.04	–0.50	–0.07	0.70	4.37
bent ω_5	–37.62	–8.44	–2.13	–0.19	35.56
HCNO total energies: Psi4(DF) – Psi4(DF)	–0.275	–0.044	–0.029	–0.008	0.101

[a]Total energies in mE_h, D_e in kcal mol^{-1}, distances in Å, angles in deg, and frequencies in cm^{-1}.

Influence of the Quadratic Bending Force Constant on the Potential Curves along the Minimum-Energy Path for H–C–N Bending in Fulminic Acid

In ref 74 we showed why the coupling of complementary coordinates with the H–C–N bend is a critical aspect of the potential energy surface (*nota bene*, bullet point 8 of the Summary). In particular, force constants of the linear HCNO structure that couple the H–C–N and C–N–O bends can give rise to lower-energy bent minima even though unrelaxed bending of these coordinates always raises the energy. Using our previously published anharmonic force fields, we have now ascertained the fully relaxed minimum-energy path (MEP) for H–C–N bending at the indisputable AE-CCSDTQ(P)/CBS + MVD1 level of theory. Figure 1 below plots the correct AE-CCSDTQ(P)/CBS + MVD1 energy profile along this path and compares it to the *ω*B97M-V, MP2, CCSD, and CCSD(T) curves computed with the def2-QZVP basis set. It is clear from the plots that *ω*B97M-V, MP2, and CCSD *all* misrepresent the correct curve in a serious way, and CCSD(T) is still not converged to the final answer. The deficiencies of MP2, CCSD, and CCSD(T) were exposed in ref 74, and now we see that *ω*B97M-V performs significantly more poorly than CCSD.

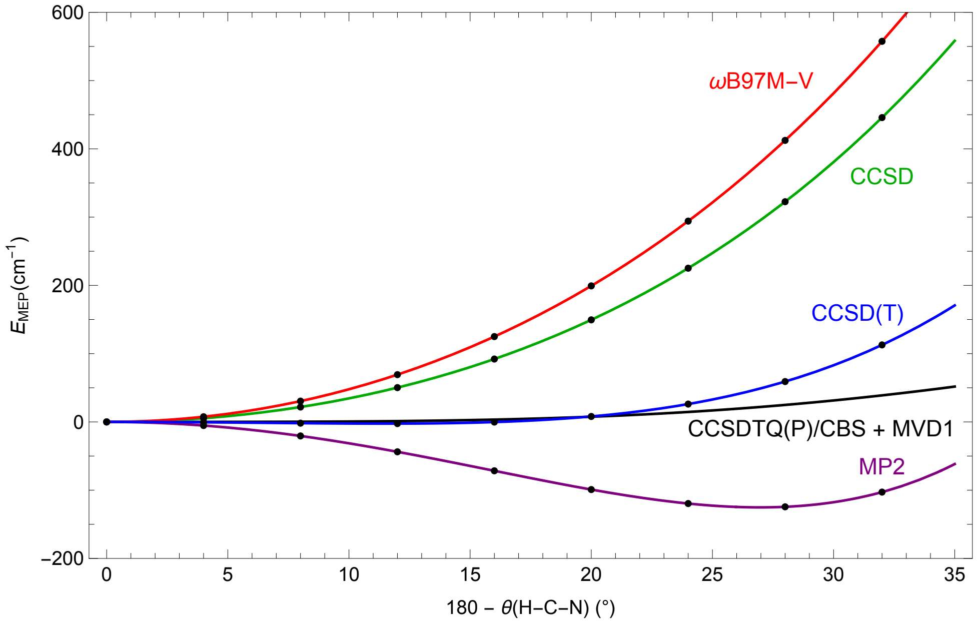

Figure S10. The *ω*B97M-V (red), MP2 (purple), CCSD (green), and CCSD(T) (blue) relative energy curves computed with the def2-QZVP basis set along the AE-CCSDTQ(P)/CBS + MVD1 minimum-energy path (MEP) for H–C–N bending in fulminic acid. The AE-CCSDTQ(P)/CBS + MVD1 (black) curve comprises the benchmark for comparison.

At several levels of theory we tested, the full quartic force field at the linear HCNO structure provides a good representation of the MEP curve until the H–C–N angle is displaced more than 35°. We can use this fact in the following mathematical experiment to *disprove* the notion that the harmonic frequency ω_5 is not a good measure of DFT performance because the potential energy surface is highly anharmonic. After mapping out the MEP curve for a given DFA, a quadratic correction (QC) function

$$E_{\mathrm{QC}}(\theta) = \tfrac{1}{2}(\delta\theta)^2[f_{\theta\theta}(\mathrm{exact}) - f_{\theta\theta}(\mathrm{DFA})]\ ,$$

can be appended, where $f_{\theta\theta}$ denotes a quadratic force constant for bending from linearity and "exact" refers to AE-CCSDTQ(P)/CBS + MVD1. The resulting model potential no longer has a bad quadratic force constant but retains the higher-order anharmonic terms of the original DFA curve. Figure 2 shows the original and quadratically corrected bending curves for HCNO given by ωB97M-V and SCAN-D3(BJ)2B with the def2-QZVP basis set. It is striking that the model potentials now lie very close to the benchmark curve. The inescapable conclusion is that a bad quadratic force constant is the essential determining factor when the MEP profile for a DFA is incorrect. Because the exact $f_{\theta\theta}$ is very close to zero, any deviation of this quantity from zero is a measure of the deficiency of the MEP profile for a DFA. Therefore, the harmonic frequencies given by the quadratic force fields are indeed a valid means of assessing DFA performance! If the quadratic potential is wrong, then the PES in this region is going to be wrong also, even if the anharmonic terms are correct.

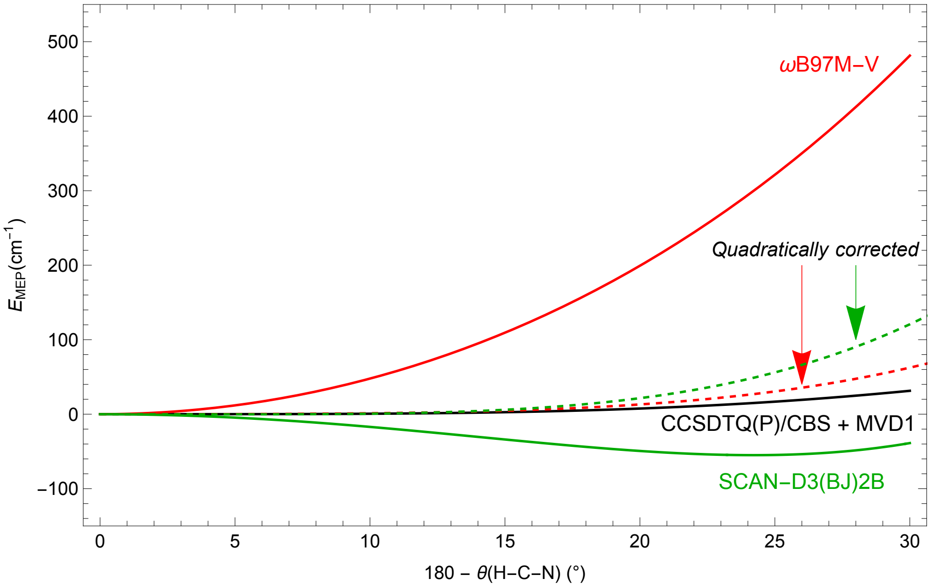

Figure S11. The effect of quadratic correction (QC) on the ωB97M-V (red), and SCAN-D3(BJ)2B (green) potential curves computed with the def2-QZVP basis set along the AE-CCSDTQ(P)/CBS + MVD1 minimum-energy path (MEP) for H–C–N bending in fulminic acid. After QC, the erroneous solid curves turn into the dashed curves that closely follow the AE-CCSDTQ(P)/CBS + MVD1 (black) benchmark, at least until the distortion from linearity exceeds 25°.

References

(1) Adamo, C.; Barone, V. Toward Reliable Adiabatic Connection Models Free from Adjustable Parameters. *Chem. Phys. Lett.* **1997**, *274*, 242–250, DOI: 10.1016/S0009-2614(97)00651-9.

(2) Grimme, S.; Ehrlich, S.; Goerigk, L. Effect of the damping function in dispersion corrected density functional theory. *J. Comput. Chem.* **2011**, *32*, 1456–1465, DOI: 10.1002/jcc.21759.

(3) Goerigk, L.; Hansen, A.; Bauer, C.; Ehrlich, S.; Najibi, A.; Grimme, S. A look at the density functional theory zoo with the advanced GMTKN55 database for general main group thermochemistry, kinetics and noncovalent interactions. *Phys. Chem. Chem. Phys.* **2017**, *19*, 32184–32215, DOI: 10.1039/C7CP04913G.

(4) Axilrod, B. M.; Teller, E. Interaction of the van der Waals Type Between Three Atoms. *J. Chem. Phys.* **1943**, *11*, 299–300, DOI: 10.1063/1.1723844.

(5) Muto, Y. Force between nonpolar molecules. *J. Phys. Math. Soc. Jpn* **1943**, *17*, 629–631.

(6) Grimme, S.; Antony, J.; Ehrlich, S.; Krieg, H. A consistent and accurate *ab initio* parametrization of density functional dispersion correction (DFT-D) for the 94 elements H-Pu. *J. Chem. Phys.* **2010**, *132*, 154104, DOI: 10.1063/1.3382344.

(7) Bilc, D. I.; Orlando, R.; Shaltaf, R.; Rignanese, G.-M.; Íñiguez, J.; Ghosez, P. Hybrid exchange-correlation functional for accurate prediction of the electronic and structural properties of ferroelectric oxides. *Phys. Rev. B* **2008**, *77*, 165107, DOI: 10.1103/PhysRevB.77.165107.

(8) Karton, A.; Tarnopolsky, A.; Lamére, J.-F.; Schatz, G. C.; Martin, J. M. L. Highly Accurate First-Principles Benchmark Data Sets for the Parametrization and Validation

of Density Functional and Other Approximate Methods. Derivation of a Robust, Generally Applicable, Double-Hybrid Functional for Thermochemistry and Thermochemical Kinetics. *J. Phys. Chem. A* **2008**, *112*, 12868–12886, DOI: 10.1021/jp801805p.

(9) Goerigk, L.; Grimme, S. A thorough benchmark of density functional methods for general main group thermochemistry, kinetics, and noncovalent interactions. *Phys. Chem. Chem. Phys.* **2011**, *13*, 6670–6688, DOI: 10.1039/C0CP02984J.

(10) Goerigk, L.; Grimme, S. Efficient and Accurate Double-Hybrid-Meta-GGA Density Functionals—Evaluation with the Extended GMTKN30 Database for General Main Group Thermochemistry, Kinetics, and Noncovalent Interactions. *J. Chem. Theory Comput.* **2011**, *7*, 291–309, DOI: 10.1021/ct100466k.

(11) Hujo, W.; Grimme, S. Performance of the van der Waals Density Functional VV10 and (hybrid)GGA Variants for Thermochemistry and Noncovalent Interactions. *J. Chem. Theory Comput.* **2011**, *7*, 3866–3871, DOI: 10.1021/ct200644w.

(12) Kesharwani, M. K.; Karton, A.; Martin, J. M. L. Benchmark *ab Initio* Conformational Energies for the Proteinogenic Amino Acids through Explicitly Correlated Methods. Assessment of Density Functional Methods. *J. Chem. Theory Comput.* **2016**, *12*, 444–454, DOI: 10.1021/acs.jctc.5b01066.

(13) Grimme, S. Semiempirical hybrid density functional with perturbative second-order correlation. *J. Chem. Phys.* **2006**, *124*, 034108, DOI: 10.1063/1.2148954.

(14) Smith, D. G. A.; Burns, L. A.; Patkowski, K.; Sherrill, C. D. Revised Damping Parameters for the D3 Dispersion Correction to Density Functional Theory. *J. Phys. Chem. Lett.* **2016**, *7*, 2197–2203, DOI: 10.1021/acs.jpclett.6b00780.

(15) Calbo, J.; Ortí, E.; Sancho-García, J. C.; Aragó, J. Accurate Treatment of Large Supramolecular Complexes by Double-Hybrid Density Functionals Coupled with Non-

local van der Waals Corrections. *J. Chem. Theory Comput.* **2015**, *11*, 932–939, DOI: 10.1021/acs.jctc.5b00002.

(16) Lee, C.; Yang, W.; Parr, R. G. Development of the Colle-Salvetti correlation-energy formula into a functional of the electron density. *Phys. Rev. B* **1988**, *37*, 785–789, DOI: 10.1103/PhysRevB.37.785.

(17) Becke, A. D. Density-functional thermochemistry. III. The role of exact exchange. *J. Chem. Phys.* **1993**, *98*, 5648–5652, DOI: 10.1063/1.464913.

(18) Becke, A. D. Density-functional exchange-energy approximation with correct asymptotic behavior. *Phys. Rev. A* **1988**, *38*, 3098–3100, DOI: 10.1103/PhysRevA.38.3098.

(19) Vosko, S. H.; Wilk, L.; Nusair, M. Accurate spin-dependent electron liquid correlation energies for local spin density calculations: a critical analysis. *Can. J. Chem.* **1980**, *58*, 1200–1211, DOI: 10.1139/p80-159.

(20) Reiher, M.; Salomon, O.; Artur Hess, B. Reparameterization of hybrid functionals based on energy differences of states of different multiplicity. *Theor. Chem. Acc.* **2001**, *107*, 48–55, DOI: 10.1007/s00214-001-0300-3.

(21) Perdew, J. P. Density-functional approximation for the correlation energy of the inhomogeneous electron gas. *Phys. Rev. B* **1986**, *33*, 8822–8824, DOI: 10.1103/PhysRevB.33.8822.

(22) Perdew, J. P.; Wang, Y. Accurate and simple analytic representation of the electron-gas correlation energy. *Phys. Rev. B* **1992**, *45*, 13244–13249, DOI: 10.1103/PhysRevB.45.13244.

(23) Shao, Y.; Head-Gordon, M.; Krylov, A. I. The spin–flip approach within time-dependent density functional theory: Theory and applications to diradicals. *J. Chem. Phys.* **2003**, *118*, 4807–4818, DOI: 10.1063/1.1545679.

(24) Becke, A. D. Density-functional thermochemistry. IV. A new dynamical correlation functional and implications for exact-exchange mixing. *J. Chem. Phys.* **1996**, *104*, 1040–1046, DOI: 10.1063/1.470829.

(25) Becke, A. D. On the large-gradient behavior of the density functional exchange energy. *J. Chem. Phys.* **1986**, *85*, 7184–7187, DOI: 10.1063/1.451353.

(26) Becke, A. D. Density-functional thermochemistry. V. Systematic optimization of exchange-correlation functionals. *J. Chem. Phys.* **1997**, *107*, 8554–8560, DOI: 10.1063/1.475007.

(27) Hamprecht, F. A.; Cohen, A. J.; Tozer, D. J.; Handy, N. C. Development and assessment of new exchange-correlation functionals. *J. Chem. Phys.* **1998**, *109*, 6264–6271, DOI: 10.1063/1.477267.

(28) Cohen, A. J.; Handy, N. C. Assessment of exchange correlation functionals. *Chem. Phys. Lett.* **2000**, *316*, 160–166, DOI: 10.1016/S0009-2614(99)01273-7.

(29) Wilson, P. J.; Bradley, T. J.; Tozer, D. J. Hybrid exchange-correlation functional determined from thermochemical data and *ab initio* potentials. *J. Chem. Phys.* **2001**, *115*, 9233–9242, DOI: 10.1063/1.1412605.

(30) Keal, T. W.; Tozer, D. J. Semiempirical hybrid functional with improved performance in an extensive chemical assessment. *J. Chem. Phys.* **2005**, *123*, 121103, DOI: 10.1063/1.2061227.

(31) Grimme, S. Semiempirical GGA-type density functional constructed with a long-range dispersion correction. *J. Comput. Chem.* **2006**, *27*, 1787–1799, DOI: 10.1002/jcc.20495.

(32) Boese, A. D.; Martin, J. M. L. Development of density functionals for thermochemical kinetics. *J. Chem. Phys.* **2004**, *121*, 3405–3416, DOI: 10.1063/1.1774975.

(33) Najibi, A.; Goerigk, L. The Nonlocal Kernel in van der Waals Density Functionals as an Additive Correction: An Extensive Analysis with Special Emphasis on the B97M-V and ωB97M-V Approaches. *J. Chem. Theory Comput.* **2018**, *14*, 5725–5738, DOI: 10.1021/acs.jctc.8b00842.

(34) Mardirossian, N.; Head-Gordon, M. Mapping the genome of meta-generalized gradient approximation density functionals: The search for B97M-V. *J. Chem. Phys.* **2015**, *142*, 074111, DOI: 10.1063/1.4907719.

(35) Zhao, Y.; Lynch, B. J.; Truhlar, D. G. Development and Assessment of a New Hybrid Density Functional Model for Thermochemical Kinetics. *J. Phys. Chem. A* **2004**, *108*, 2715–2719, DOI: 10.1021/jp049908s.

(36) Becke, A. D. A new mixing of Hartree–Fock and local density-functional theories. *J. Chem. Phys.* **1993**, *98*, 1372–1377, DOI: 10.1063/1.464304.

(37) Tsuneda, T.; Suzumura, T.; Hirao, K. A new one-parameter progressive Colle–Salvetti-type correlation functional. *J. Chem. Phys.* **1999**, *110*, 10664–10678.

(38) Yanai, T.; Tew, D. P.; Handy, N. C. A new hybrid exchange–correlation functional using the Coulomb-attenuating method (CAM-B3LYP). *Chem. Phys. Lett.* **2004**, *393*, 51–57, DOI: 10.1016/j.cplett.2004.06.011.

(39) Mosquera, M. A.; Borca, C. H.; Ratner, M. A.; Schatz, G. C. Connection between Hybrid Functionals and Importance of the Local Density Approximation. *J. Phys. Chem. A* **2016**, *120*, 1605–1612, DOI: 10.1021/acs.jpca.5b10864.

(40) Carmona-Espíndola, J.; Gázquez, J. L.; Vela, A.; Trickey, S. B. Global hybrid exchange energy functional with correct asymptotic behavior of the corresponding potential. *Theor. Chem. Acc.* **2016**, *135*, 120, DOI: 10.1007/s00214-016-1864-2.

(41) Kozuch, S.; Gruzman, D.; Martin, J. M. L. DSD-BLYP: A General Purpose Double Hybrid Density Functional Including Spin Component Scaling and Dispersion Correction. *J. Phys. Chem. C* **2010**, *114*, 20801–20808, DOI: 10.1021/jp1070852.

(42) Yu, F. Spin-Component-Scaled Double-Hybrid Density Functionals with Nonlocal van der Waals Correlations for Noncovalent Interactions. *J. Chem. Theory Comput.* **2014**, *10*, 4400–4407, DOI: 10.1021/ct500642x.

(43) Pernal, K.; Podeszwa, R.; Patkowski, K.; Szalewicz, K. Dispersionless Density Functional Theory. *Phys. Rev. Lett.* **2009**, *103*, 263201, DOI: 10.1103/PhysRevLett.103.263201.

(44) Podeszwa, R.; Pernal, K.; Patkowski, K.; Szalewicz, K. Extension of the Hartree–Fock Plus Dispersion Method by First-Order Correlation Effects. *J. Phys. Chem. Lett.* **2010**, *1*, 550–555, DOI: 10.1021/jz9002444.

(45) Kozuch, S.; Martin, J. M. L. Spin-component-scaled double hybrids: An extensive search for the best fifth-rung functionals blending DFT and perturbation theory. *J. Comput. Chem.* **2013**, *34*, 2327–2344, DOI: 10.1002/jcc.23391.

(46) Kozuch, S.; Martin, J. M. L. DSD-PBEP86: in search of the best double-hybrid DFT with spin-component scaled MP2 and dispersion corrections. *Phys. Chem. Chem. Phys.* **2011**, *13*, 20104–20107, DOI: 10.1039/C1CP22592H.

(47) Adamson, R. D.; Gill, P. M.; Pople, J. A. Empirical density functionals. *Chem. Phys. Lett.* **1998**, *284*, 6–11, DOI: 10.1016/S0009-2614(97)01282-7.

(48) Lin, C. Y.; George, M. W.; Gill, P. M. W. EDF2: A Density Functional for Predicting Molecular Vibrational Frequencies. *Aust. J. Chem.* **2004**, *57*, 365–370, DOI: 10.1071/CH03263.

(49) Filatov, M.; Thiel, W. A nonlocal correlation energy density functional from a Coulomb hole model. *Int. J. Quantum Chem.* **1997**, *62*, 603–616, DOI: 10.1002/(SICI)1097-461X(1997)62:6<603::AID-QUA4>3.0.CO;2-\#.

(50) Yu, H. S.; Zhang, W.; Verma, P.; He, X.; Truhlar, D. G. Nonseparable exchange–correlation functional for molecules, including homogeneous catalysis involving transition metals. *Phys. Chem. Chem. Phys.* **2015**, *17*, 12146–12160, DOI: 10.1039/C5CP01425E.

(51) Boese, A. D.; Doltsinis, N. L.; Handy, N. C.; Sprik, M. New generalized gradient approximation functionals. *J. Chem. Phys.* **2000**, *112*, 1670–1678, DOI: 10.1063/1.480732.

(52) Boese, A. D.; Handy, N. C. A new parametrization of exchange–correlation generalized gradient approximation functionals. *J. Chem. Phys.* **2001**, *114*, 5497–5503, DOI: 10.1063/1.1347371.

(53) Boese, A. D.; Chandra, A.; Martin, J. M. L.; Marx, D. From *ab initio* quantum chemistry to molecular dynamics: The delicate case of hydrogen bonding in ammonia. *J. Chem. Phys.* **2003**, *119*, 5965–5980, DOI: 10.1063/1.1599338.

(54) Hamprecht, F. A.; Cohen, A. J.; Tozer, D. J.; Handy, N. C. Development and assessment of new exchange-correlation functionals. *J. Chem. Phys.* **1998**, *109*, 626–4–6271, DOI: 10.1063/1.477267.

(55) Menconi, G.; Wilson, P. J.; Tozer, D. J. Emphasizing the exchange-correlation potential in functional development. *J. Chem. Phys.* **2001**, *114*, 3958–3967, DOI: 10.1063/1.1342776.

(56) Fock, V. Näherungsmethode zur Lösung des quantenmechanischen Mehrkörperproblems. *Z. Physik* **1930**, *61*, 126–148, DOI: 10.1007/BF01340294.

(57) Henderson, T. M.; Janesko, B. G.; Scuseria, G. E. Generalized gradient approximation model exchange holes for range-separated hybrids. *J. Chem. Phys.* **2008**, *128*, 194105, DOI: 10.1063/1.2921797.

(58) Schimka, L.; Harl, J.; Kresse, G. Improved hybrid functional for solids: The HSEsol functional. *J. Chem. Phys.* **2011**, *134*, 024116, DOI: 10.1063/1.3524336.

(59) Fabiano, E.; Constantin, L. A.; Della Sala, F. Testing the broad applicability of the PBEint GGA functional and its one-parameter hybrid form. *Int. J. Quantum Chem.* **2013**, *113*, 673–682, DOI: 10.1002/qua.24042.

(60) Heyd, J.; Scuseria, G. E.; Ernzerhof, M. Hybrid functionals based on a screened Coulomb potential. *J. Chem. Phys.* **2003**, *118*, 8207–8215, DOI: 10.1063/1.1564060.

(61) Krukau, A. V.; Vydrov, O. A.; Izmaylov, A. F.; Scuseria, G. E. Influence of the exchange screening parameter on the performance of screened hybrid functionals. *J. Chem. Phys.* **2006**, *125*, 224106, DOI: 10.1063/1.2404663.

(62) Moellmann, J.; Grimme, S. DFT-D3 Study of Some Molecular Crystals. *J. Phys. Chem. C* **2014**, *118*, 7615–7621, DOI: 10.1021/jp501237c.

(63) Kang, J. K.; Musgrave, C. B. Prediction of transition state barriers and enthalpies of reaction by a new hybrid density-functional approximation. *J. Chem. Phys.* **2001**, *115*, 11040–11051, DOI: 10.1063/1.1415079.

(64) Karasiev, V. V.; Sjostrom, T.; Dufty, J.; Trickey, S. B. Accurate Homogeneous Electron Gas Exchange-Correlation Free Energy for Local Spin-Density Calculations. *Phys. Rev. Lett.* **2014**, *112*, 076403, DOI: 10.1103/PhysRevLett.112.076403.

(65) Keal, T. W.; Tozer, D. J. The exchange-correlation potential in Kohn–Sham nuclear magnetic resonance shielding calculations. *J. Chem. Phys.* **2003**, *119*, 3015–3024, DOI: 10.1063/1.1590634.

(66) Song, J.-W.; Hirosawa, T.; Tsuneda, T.; Hirao, K. Long-range corrected density functional calculations of chemical reactions: Redetermination of parameter. *J. Chem. Phys.* **2007**, *126*, 154105, DOI: 10.1063/1.2721532.

(67) Iikura, H.; Tsuneda, T.; Yanai, T.; Hirao, K. A long-range correction scheme for generalized-gradient-approximation exchange functionals. *J. Chem. Phys.* **2001**, *115*, 3540–3544, DOI: 10.1063/1.1383587.

(68) Tawada, Y.; Tsuneda, T.; Yanagisawa, S.; Yanai, T.; Hirao, K. A long-range-corrected time-dependent density functional theory. *J. Chem. Phys.* **2004**, *120*, 8425–8433, DOI: 10.1063/1.1688752.

(69) Vydrov, O. A.; Van Voorhis, T. Nonlocal van der Waals density functional: The simpler the better. *J. Chem. Phys.* **2010**, *133*, 244103, DOI: 10.1063/1.3521275.

(70) Rinke, P.; Schleife, A.; Kioupakis, E.; Janotti, A.; Rödl, C.; Bechstedt, F.; Scheffler, M.; Van de Walle, C. G. First-Principles Optical Spectra for F Centers in MgO. *Phys. Rev. Lett.* **2012**, *108*, 126404, DOI: 10.1103/PhysRevLett.108.126404.

(71) Rohrdanz, M. A.; Herbert, J. M. Simultaneous benchmarking of ground- and excited-state properties with long-range-corrected density functional theory. *J. Chem. Phys.* **2008**, *129*, 034107, DOI: 10.1063/1.2954017.

(72) Rohrdanz, M. A.; Martins, K. M.; Herbert, J. M. A long-range-corrected density functional that performs well for both ground-state properties and time-dependent density functional theory excitation energies, including charge-transfer excited states. *J. Chem. Phys.* **2009**, *130*, 054112, DOI: 10.1063/1.3073302.

(73) Zhao, Y.; Schultz, N. E.; Truhlar, D. G. Exchange-correlation functional with broad accuracy for metallic and nonmetallic compounds, kinetics, and noncovalent interactions. *J. Chem. Phys.* **2005**, *123*, 161103, DOI: 10.1063/1.2126975.

(74) Zhao, Y.; Schultz, N. E.; Truhlar, D. G. Design of Density Functionals by Combining the Method of Constraint Satisfaction with Parametrization for Thermochemistry, Thermochemical Kinetics, and Noncovalent Interactions. *J. Chem. Theory Comput.* **2006**, *2*, 364–382, DOI: 10.1021/ct0502763.

(75) Zhao, Y.; Truhlar, D. G. The M06 suite of density functionals for main group thermochemistry, thermochemical kinetics, noncovalent interactions, excited states, and transition elements: two new functionals and systematic testing of four M06-class functionals and 12 other functionals. *Theor. Chem. Acc.* **2008**, *120*, 215–241, DOI: 10.1007/s00214-007-0310-x.

(76) Zhao, Y.; Truhlar, D. G. Density Functional for Spectroscopy: No Long-Range Self-Interaction Error, Good Performance for Rydberg and Charge-Transfer States, and Better Performance on Average than B3LYP for Ground States. *J. Phys. Chem. A* **2006**, *110*, 13126–13130, DOI: 10.1021/jp066479k.

(77) Zhao, Y.; Truhlar, D. G. A new local density functional for main-group thermochemistry, transition metal bonding, thermochemical kinetics, and noncovalent interactions. *J. Chem. Phys.* **2006**, *125*, 194101, DOI: 10.1063/1.2370993.

(78) Zhao, Y.; Truhlar, D. G. Exploring the Limit of Accuracy of the Global Hybrid Meta Density Functional for Main-Group Thermochemistry, Kinetics, and Noncovalent Interactions. *J. Chem. Theory Comput.* **2008**, *4*, 1849–1868, DOI: 10.1021/ct800246v.

(79) Peverati, R.; Truhlar, D. G. Improving the Accuracy of Hybrid Meta-GGA Density Functionals by Range Separation. *J. Phys. Chem. Lett.* **2011**, *2*, 2810–2817, DOI: 10.1021/jz201170d.

(80) Goerigk, L. Treating London-Dispersion Effects with the Latest Minnesota Density Functionals: Problems and Possible Solutions. *J. Phys. Chem. Lett.* **2015**, *6*, 3891–3896, DOI: 10.1021/acs.jpclett.5b01591.

(81) Peverati, R.; Truhlar, D. G. M11-L: A Local Density Functional That Provides Improved Accuracy for Electronic Structure Calculations in Chemistry and Physics. *J. Phys. Chem. Lett.* **2012**, *3*, 117–124, DOI: 10.1021/jz201525m.

(82) Tognetti, V.; Cortona, P.; Adamo, C. The performances of a parameter-free local correlation functional: The Ragot–Cortona model. *Chem. Phys. Lett.* **2007**, *439*, 381–385, DOI: 10.1016/j.cplett.2007.03.081.

(83) Sun, J.; Xiao, B.; Ruzsinszky, A. Communication: Effect of the orbital-overlap dependence in the meta generalized gradient approximation. *J. Chem. Phys.* **2012**, *137*, 051101, DOI: 10.1063/1.4742312.

(84) Sun, J.; Haunschild, R.; Xiao, B.; Bulik, I. W.; Scuseria, G. E.; Perdew, J. P. Semilocal and hybrid meta-generalized gradient approximations based on the understanding of the kinetic-energy-density dependence. *J. Chem. Phys.* **2013**, *138*, 044113, DOI: 10.1063/1.4789414.

(85) Sun, J.; Perdew, J. P.; Ruzsinszky, A. Semilocal density functional obeying a strongly tightened bound for exchange. *Proc. Nat. Acad. Sci. U.S.A.* **2015**, *112*, 685–689, DOI: 10.1073/pnas.1423145112.

(86) Peverati, R.; Truhlar, D. G. An improved and broadly accurate local approximation to the exchange–correlation density functional: The MN12-L functional for electronic structure calculations in chemistry and physics. *Phys. Chem. Chem. Phys.* **2012**, *14*, 13171–13174, DOI: 10.1039/C2CP42025B.

(87) Peverati, R.; Truhlar, D. G. Screened-exchange density functionals with broad accuracy for chemistry and solid-state physics. *Phys. Chem. Chem. Phys.* **2012**, *14*, 16187–16191, DOI: 10.1039/C2CP42576A.

(88) Yu, H. S.; He, X.; Li, S. L.; Truhlar, D. G. MN15: A Kohn–Sham global-hybrid exchange–correlation density functional with broad accuracy for multi-reference and

single-reference systems and noncovalent interactions. *Chem. Sci.* **2016**, *7*, 5032–5051, DOI: 10.1039/C6SC00705H.

(89) Yu, H. S.; He, X.; Truhlar, D. G. MN15-L: A New Local Exchange-Correlation Functional for Kohn–Sham Density Functional Theory with Broad Accuracy for Atoms, Molecules, and Solids. *J. Chem. Theory Comput.* **2016**, *12*, 1280–1293, DOI: 10.1021/acs.jctc.5b01082.

(90) Schultz, N. E.; Zhao, Y.; Truhlar, D. G. Density Functionals for Inorganometallic and Organometallic Chemistry. *J. Phys. Chem. A* **2005**, *109*, 11127–11143, DOI: 10.1021/jp0539223.

(91) Zheng, J.; Zhao, Y.; Truhlar, D. G. The DBH24/08 Database and Its Use to Assess Electronic Structure Model Chemistries for Chemical Reaction Barrier Heights. *J. Chem. Theory Comput.* **2009**, *5*, 808–821, DOI: 10.1021/ct800568m.

(92) Řezáč, J.; Greenwell, C.; Beran, G. J. O. Accurate Noncovalent Interactions via Dispersion-Corrected Second-Order Møller–Plesset Perturbation Theory. *J. Chem. Theory Comput.* **2018**, *14*, 4711–4721, DOI: 10.1021/acs.jctc.8b00548.

(93) Møller, C.; Plesset, M. S. Note on an Approximation Treatment for Many-Electron Systems. *Phys. Rev.* **1934**, *46*, 618–622, DOI: 10.1103/PhysRev.46.618.

(94) Zhao, Y.; Truhlar, D. G. Hybrid Meta Density Functional Theory Methods for Thermochemistry, Thermochemical Kinetics, and Noncovalent Interactions: The MPW1B95 and MPWB1K Models and Comparative Assessments for Hydrogen Bonding and van der Waals Interactions. *J. Phys. Chem. A* **2004**, *108*, 6908–6918, DOI: 10.1021/jp048147q.

(95) Lynch, B. J.; Fast, P. L.; Harris, M.; Truhlar, D. G. Adiabatic Connection for Kinetics. *J. Phys. Chem. A* **2000**, *104*, 4811–4815, DOI: 10.1021/jp000497z.

(96) Adamo, C.; Barone, V. Exchange functionals with improved long-range behavior and adiabatic connection methods without adjustable parameters: The mPW and mPW1PW models. *J. Chem. Phys.* **1998**, *108*, 664–675, DOI: 10.1063/1.475428.

(97) Perdew, J. P.; Burke, K.; Ernzerhof, M. Generalized Gradient Approximation Made Simple. *Phys. Rev. Lett.* **1996**, *77*, 3865–3868, DOI: 10.1103/PhysRevLett.77.3865.

(98) Dahlke, E. E.; Truhlar, D. G. Improved Density Functionals for Water. *J. Phys. Chem. B* **2005**, *109*, 15677–15683, DOI: 10.1021/jp052436c.

(99) Peverati, R.; Truhlar, D. G. Exchange–Correlation Functional with Good Accuracy for Both Structural and Energetic Properties while Depending Only on the Density and Its Gradient. *J. Chem. Theory Comput.* **2012**, *8*, 2310–2319, DOI: 10.1021/ct3002656.

(100) Hoe, W.-M.; Cohen, A. J.; Handy, N. C. Assessment of a new local exchange functional OPTX. *Chem. Phys. Lett.* **2001**, *341*, 319–328, DOI: 10.1016/S0009-2614(01)00581-4.

(101) Cohen, A. J.; Handy, N. C. Dynamic correlation. *Mol. Phys.* **2001**, *99*, 607–615, DOI: 10.1080/00268970010023435.

(102) Goerigk, L.; Grimme, S. A General Database for Main Group Thermochemistry, Kinetics, and Noncovalent Interactions - Assessment of Common and Reparameterized (meta-)GGA Density Functionals. *J. Chem. Theory Comput.* **2010**, *6*, 107–126, DOI: 10.1021/ct900489g.

(103) Tsuneda, T.; Suzumura, T.; Hirao, K. A new one-parameter progressive Colle–Salvetti-type correlation functional. *J. Chem. Phys.* **1999**, *110*, 10664–10678, DOI: 10.1063/1.479012.

(104) Tsuneda, T.; Suzumura, T.; Hirao, K. A reexamination of exchange energy functionals. *J. Chem. Phys.* **1999**, *111*, 5656–5667, DOI: 10.1063/1.479954.

(105) Perdew, J. P.; Ruzsinszky, A.; Csonka, G. I.; Vydrov, O. A.; Scuseria, G. E.; Constantin, L. A.; Zhou, X.; Burke, K. Restoring the Density-Gradient Expansion for Exchange in Solids and Surfaces. *Phys. Rev. Lett.* **2008**, *100*, 136406, DOI: 10.1103/PhysRevLett.100.136406.

(106) Adamo, C.; Barone, V. Toward reliable density functional methods without adjustable parameters: The PBE0 model. *J. Chem. Phys.* **1999**, *110*, 6158–6170, DOI: 10.1063/1.478522.

(107) Ernzerhof, M.; Scuseria, G. E. Assessment of the Perdew–Burke–Ernzerhof exchange-correlation functional. *J. Chem. Phys.* **1999**, *110*, 5029–5036, DOI: 10.1063/1.478401.

(108) Cortona, P. Note: Theoretical mixing coefficients for hybrid functionals. *J. Chem. Phys.* **2012**, *136*, 086101, DOI: 10.1063/1.3690462.

(109) Chai, J.-D.; Mao, S.-P. Seeking for reliable double-hybrid density functionals without fitting parameters: The PBE0-2 functional. *Chem. Phys. Lett.* **2012**, *538*, 121–125, DOI: 10.1016/j.cplett.2012.04.045.

(110) Brémond, E.; Adamo, C. Seeking for parameter-free double-hybrid functionals: The PBE0-DH model. *J. Chem. Phys.* **2011**, *135*, 024106, DOI: 10.1063/1.3604569.

(111) Bousquet, D.; Brémond, E.; Sancho-García, J. C.; Ciofini, I.; Adamo, C. Non-parametrized functionals with empirical dispersion corrections: A happy match? *Theor. Chem. Acc. 134*, 1602, DOI: 10.1007/s00214-014-1602-6.

(112) Grimme, S.; Brandenburg, J. G.; Bannwarth, C.; Hansen, A. Consistent structures and interactions by density functional theory with small atomic orbital basis sets. *J. Chem. Phys.* **2015**, *143*, 054107, DOI: 10.1063/1.4927476.

(113) Perdew, J. P.; Kurth, S.; Zupan, A. c. v.; Blaha, P. Accurate Density Functional with

Correct Formal Properties: A Step Beyond the Generalized Gradient Approximation. *Phys. Rev. Lett.* **1999**, *82*, 2544–2547, DOI: 10.1103/PhysRevLett.82.2544.

(114) Goerigk, L.; Grimme, S. Efficient and Accurate Double-Hybrid-Meta-GGA Density Functionals—Evaluation with the Extended GMTKN30 Database for General Main Group Thermochemistry, Kinetics, and Noncovalent Interactions. *J. Chem. Theory Comput.* **2011**, *7*, 291–309, DOI: 10.1021/ct100466k.

(115) Zhao, Y.; Truhlar, D. G. Design of Density Functionals That Are Broadly Accurate for Thermochemistry, Thermochemical Kinetics, and Nonbonded Interactions. *J. Phys. Chem. A* **2005**, *109*, 5656–5667, DOI: 10.1021/jp050536c.

(116) Perdew, J. P.; Yue, W. Accurate and simple density functional for the electronic exchange energy: Generalized gradient approximation. *Phys. Rev. B* **1986**, *33*, 8800–8802, DOI: 10.1103/PhysRevB.33.8800.

(117) Perdew, J. P.; Chevary, J. A.; Vosko, S. H.; Jackson, K. A.; Pederson, M. R.; Singh, D. J.; Fiolhais, C. Atoms, molecules, solids, and surfaces: Applications of the generalized gradient approximation for exchange and correlation. *Phys. Rev. B* **1992**, *46*, 6671–6687, DOI: 10.1103/PhysRevB.46.6671.

(118) Reimers, J. R.; Panduwinata, D.; Visser, J.; Chin, Y.; Tang, C.; Goerigk, L.; Ford, M. J.; Sintic, M.; Sum, T.-J.; Coenen, M. J. J. et al. A priori calculations of the free energy of formation from solution of polymorphic self-assembled monolayers. *Proc. Nat. Acad. Sci. U.S.A.* **2015**, *112*, E6101–E6110, DOI: 10.1073/pnas.1516984112.

(119) Yu, F. Spin-Component-Scaled Double-Hybrid Density Functionals with Nonlocal van der Waals Correlations for Noncovalent Interactions. *J. Chem. Theory Comput.* **2014**, *10*, 4400–4407, DOI: 10.1021/ct500642x.

(120) Lu, L.; Hu, H.; Hou, H.; Wang, B. An improved B3LYP method in the calculation of

organic thermochemistry and reactivity. *Comput. Theor. Chem.* **2013**, *1015*, 64–71, DOI: 10.1016/j.comptc.2013.04.009.

(121) Wang, Y.; Jin, X.; Yu, H. S.; Truhlar, D. G.; He, X. Revised M06-L functional for improved accuracy on chemical reaction barrier heights, noncovalent interactions, and solid-state physics. *Proc. Nat. Acad. Sci. U.S.A.* **2017**, *114*, 8487–8492, DOI: 10.1073/pnas.1705670114.

(122) Zhang, Y.; Yang, W. Comment on "Generalized Gradient Approximation Made Simple". *Phys. Rev. Lett.* **1998**, *80*, 890–890, DOI: 10.1103/PhysRevLett.80.890.

(123) Mezei, P. D.; Csonka, G. I.; Kállay, M. Simple Modifications of the SCAN Meta-Generalized Gradient Approximation Functional. *J. Chem. Theory Comput.* **2018**, *14*, 2469–2479, DOI: 10.1021/acs.jctc.8b00072.

(124) Sun, J.; Marsman, M.; Csonka, G. I.; Ruzsinszky, A.; Hao, P.; Kim, Y.-S.; Kresse, G.; Perdew, J. P. Self-consistent meta-generalized gradient approximation within the projector-augmented-wave method. *Phys. Rev. B* **2011**, *84*, 035117, DOI: 10.1103/PhysRevB.84.035117.

(125) Kruse, H.; Banáš, P.; Šponer, J. Investigations of Stacked DNA Base-Pair Steps: Highly Accurate Stacking Interaction Energies, Energy Decomposition, and Many-Body Stacking Effects. *J. Chem. Theory Comput.* **2019**, *15*, 95–115, DOI: 10.1021/acs.jctc.8b00643.

(126) Csonka, G. I.; Perdew, J. P.; Ruzsinszky, A. Global Hybrid Functionals: A Look at the Engine under the Hood. *J. Chem. Theory Comput.* **2010**, *6*, 3688–3703, DOI: 10.1021/ct100488v.

(127) Hammer, B.; Hansen, L. B.; Nørskov, J. K. Improved adsorption energetics within density-functional theory using revised Perdew-Burke-Ernzerhof functionals. *Phys. Rev. B* **1999**, *59*, 7413–7421, DOI: 10.1103/PhysRevB.59.7413.

(128) Schmider, H. L.; Becke, A. D. Optimized density functionals from the extended G2 test set. *J. Chem. Phys.* **1998**, *108*, 9624–9631, DOI: 10.1063/1.476438.

(129) Sun, J.; Ruzsinszky, A.; Perdew, J. P. Strongly Constrained and Appropriately Normed Semilocal Density Functional. *Phys. Rev. Lett.* **2015**, *115*, 036402, DOI: 10.1103/PhysRevLett.115.036402.

(130) Brandenburg, J. G.; Bates, J. E.; Sun, J.; Perdew, J. P. Benchmark tests of a strongly constrained semilocal functional with a long-range dispersion correction. *Phys. Rev. B* **2016**, *94*, 115144, DOI: 10.1103/PhysRevB.94.115144.

(131) Hui, K.; Chai, J.-D. SCAN-based hybrid and double-hybrid density functionals from models without fitted parameters. *J. Chem. Phys.* **2016**, *144*, 044114, DOI: 10.1063/1.4940734.

(132) Zhao, Y.; Truhlar, D. G. Construction of a generalized gradient approximation by restoring the density-gradient expansion and enforcing a tight Lieb–Oxford bound. *J. Chem. Phys.* **2008**, *128*, 184109, DOI: 10.1063/1.2912068.

(133) Peverati, R.; Zhao, Y.; Truhlar, D. G. Generalized Gradient Approximation That Recovers the Second-Order Density-Gradient Expansion with Optimized Across-the-Board Performance. *J. Phys. Chem. Lett.* **2011**, *2*, 1991–1997, DOI: 10.1021/jz200616w.

(134) Peverati, R.; Truhlar, D. G. Communication: A global hybrid generalized gradient approximation to the exchange-correlation functional that satisfies the second-order density-gradient constraint and has broad applicability in chemistry. *J. Chem. Phys.* **2011**, *135*, 191102, DOI: 10.1063/1.3663871.

(135) Dirac, P. A. M. Note on Exchange Phenomena in the Thomas Atom. *Math. Proc. Cambridge Philos. Soc.* **1930**, *26*, 376—385, DOI: 10.1017/S0305004100016108.

(136) Bloch, F. Bemerkung zur Elektronentheorie des Ferromagnetismus und der elektrischen Leitfähigkeit. *Zeitschrift für Physik* **1929**, *57*, 545–555, DOI: 10.1007/BF01340281.

(137) Boese, A. D.; Handy, N. C. New exchange-correlation density functionals: The role of the kinetic-energy density. *J. Chem. Phys.* **2002**, *116*, 9559–9569.

(138) Goedecker, S.; Teter, M.; Hutter, J. Separable dual-space Gaussian pseudopotentials. *Phys. Rev. B* **1996**, *54*, 1703–1710, DOI: 10.1103/PhysRevB.54.1703.

(139) Tozer, D. J.; Handy, N. C.; Green, W. H. Exchange-correlation functionals from ab initio electron densities. *Chem. Phys. Lett.* **1997**, *273*, 183–194, DOI: 10.1016/S0009-2614(97)00586-1.

(140) Tozer, D. J.; Handy, N. C. The development of new exchange-correlation functionals. *J. Chem. Phys.* **1998**, *108*, 2545–2555, DOI: 10.1063/1.475638.

(141) Tozer, D. J.; Handy, N. C. Development of New Exchange-Correlation Functionals. 2. *J. Phys. Chem. A* **1998**, *102*, 3162–3168, DOI: 10.1021/jp980259s.

(142) Handy, N. C.; Tozer, D. J. The development of new exchange-correlation functionals: 3. *Mol. Phys.* **1998**, *94*, 707–715, DOI: 10.1080/002689798167863.

(143) Tao, J.; Perdew, J. P.; Staroverov, V. N.; Scuseria, G. E. Climbing the Density Functional Ladder: Nonempirical Meta–Generalized Gradient Approximation Designed for Molecules and Solids. *Phys. Rev. Lett.* **2003**, *91*, 146401, DOI: 10.1103/PhysRevLett.91.146401.

(144) Hujo, W.; Grimme, S. Performance of Non-Local and Atom-Pairwise Dispersion Corrections to DFT for Structural Parameters of Molecules with Noncovalent Interactions. *J. Chem. Theory Comput.* **2013**, *9*, 308–315, DOI: 10.1021/ct300813c.

(145) Staroverov, V. N.; Scuseria, G. E.; Tao, J.; Perdew, J. P. Comparative assessment of a

new nonempirical density functional: Molecules and hydrogen-bonded complexes. *J. Chem. Phys.* **2003**, *119*, 12129–12137, DOI: 10.1063/1.1626543.

(146) Okuno, K.; Shigeta, Y.; Kishi, R.; Miyasaka, H.; Nakano, M. Tuned CAM-B3LYP functional in the time-dependent density functional theory scheme for excitation energies and properties of diarylethene derivatives. *J. Photochem. Photobiol. A* **2012**, *235*, 29–34, DOI: 10.1016/j.jphotochem.2012.03.003.

(147) Van Voorhis, T.; Scuseria, G. E. A novel form for the exchange-correlation energy functional. *J. Chem. Phys.* **1998**, *109*, 400–410, DOI: 10.1063/1.476577.

(148) Chai, J.-D.; Head-Gordon, M. Systematic optimization of long-range corrected hybrid density functionals. *J. Chem. Phys.* **2008**, *128*, 084106, DOI: 10.1063/1.2834918.

(149) Najibi, A.; Goerigk, L. The Nonlocal Kernel in van der Waals Density Functionals as an Additive Correction: An Extensive Analysis with Special Emphasis on the B97M-V and ωB97M-V Approaches. *J. Chem. Theory Comput.* **2018**, *14*, 5725–5738, DOI: 10.1021/acs.jctc.8b00842.

(150) Mardirossian, N.; Head-Gordon, M. ωB97M-V: A combinatorially optimized, range-separated hybrid, meta-GGA density functional with VV10 nonlocal correlation. *J. Chem. Phys.* **2016**, *144*, 214110, DOI: 10.1063/1.4952647.

(151) Chai, J.-D.; Head-Gordon, M. Long-range corrected hybrid density functionals with damped atom–atom dispersion corrections. *Phys. Chem. Chem. Phys.* **2008**, *10*, 6615–6620, DOI: 10.1039/B810189B.

(152) Lin, Y.-S.; Li, G.-D.; Mao, S.-P.; Chai, J.-D. Long-Range Corrected Hybrid Density Functionals with Improved Dispersion Corrections. *J. Chem. Theory Comput.* **2013**, *9*, 263–272, DOI: 10.1021/ct300715s.

(153) Mardirossian, N.; Head-Gordon, M. ωB97X-V: A 10-parameter, range-separated hybrid, generalized gradient approximation density functional with nonlocal correlation, designed by a survival-of-the-fittest strategy. *Phys. Chem. Chem. Phys.* **2014**, *16*, 9904–9924, DOI: 10.1039/C3CP54374A.

(154) Weintraub, E.; Henderson, T. M.; Scuseria, G. E. Long-Range-Corrected Hybrids Based on a New Model Exchange Hole. *J. Chem. Theory Comput.* **2009**, *5*, 754–762, DOI: 10.1021/ct800530u.

(155) Xu, X.; Goddard, W. A. The X3LYP extended density functional for accurate descriptions of nonbond interactions, spin states, and thermochemical properties. *Proc. Nat. Acad. Sci. U.S.A.* **2004**, *101*, 2673–2677, DOI: 10.1073/pnas.0308730100.

(156) Zhao, Q.; Levy, M.; Parr, R. G. Applications of coordinate-scaling procedures to the exchange-correlation energy. *Phys. Rev. A* **1993**, *47*, 918–922, DOI: 10.1103/PhysRevA.47.918.
